# Methyl Salicylate Level Increase in Flax after *Fusarium oxysporum* Infection Is Associated with Phenylpropanoid Pathway Activation

**DOI:** 10.3389/fpls.2016.01951

**Published:** 2017-01-20

**Authors:** Aleksandra Boba, Kamil Kostyn, Anna Kostyn, Wioleta Wojtasik, Mariusz Dziadas, Marta Preisner, Jan Szopa, Anna Kulma

**Affiliations:** ^1^Faculty of Biotechnology, University of WrocławWrocław, Poland; ^2^Department of Genetics, Institute of Genetics and Microbiology, University of WroclawWroclaw, Poland; ^3^Department of Genetics, Plant Breeding and Seed Production, Faculty of Life Sciences and Technology, Wroclaw University of Environmental and Plant SciencesWroclaw, Poland; ^4^Department of Food Science and Dietetics, Medical University of WroclawWroclaw, Poland

**Keywords:** flax, *Fusarium oxysporum*, benzoate, salicylic acid, phenylpropanoids

## Abstract

Flax (*Linum usitatissimum*) is a crop plant valued for its oil and fiber. Unfortunately, large losses in cultivation of this plant are caused by fungal infections, with *Fusarium oxysporum* being one of its most dangerous pathogens. Among the plant's defense strategies, changes in the expression of genes of the shikimate/phenylpropanoid/benzoate pathway and thus in phenolic contents occur. Among the benzoates, salicylic acid, and its methylated form methyl salicylate play an important role in regulating plants' response to stress conditions. Upon treatment of flax plants with the fungus we found that methyl salicylate content increased (4.8-fold of the control) and the expression profiles of the analyzed genes suggest that it is produced most likely from cinnamic acid, through the β-oxidative route. At the same time activation of some genes involved in lignin and flavonoid biosynthesis was observed. We suggest that increased methyl salicylate biosynthesis during flax response to *F. oxysporum* infection may be associated with phenylpropanoid pathway activation.

## Introduction

Flax (*Linum usitatissimum*) is a crop plant utilized in many branches of industry as it is a source of oil and fiber, as well as the secondary products—seedcakes and shives. The flax raw products are applied in the food, medical, clothing, cosmetic, chemical, and building industries to name only the most important. Among the advantages of flax is virtually complete utilization of the plant, thus rendering it a zero-waste crop. Unfortunately, the cultivation of flax is stymied because of pathogens, with *Fusarium* causing the most losses. It is estimated that the flax crop loss caused by this genus of pathogenic fungi reaches 20% (Muir and Westcott, 2003; Heller et al., 2006). Although *Fusarium* fungi are mostly soil saprophytes, some species/strains are belligerent toward plants. *Fusarium oxysporum f*.sp. *lini* is a flax-specific pathogen and is considered the most dangerous species for flax. It invades the plant through roots to spread inside the vascular bundles, where it develops microconidia that after germination block the water and nutrient flow, leading to plant wilt, yellowing of lower parts and death (Olivain et al., 2003; Michielse and Rep, 2009), which is why it is included among necrotrophic pathogens. Sometimes the fungus is called a hemibiotroph, because infection initially resembles that of a pathogen that relies on a living host (biotrophic), but eventually transition to killing and consuming host cells (necrotrophic) occurs (Krol et al., 2015). The fungus produces mycotoxins and enzymes hydrolysing cell wall components (cellulases, pectinases, glucuronidases, etc.) that facilitate host tissue penetration.

**Figure 1:**
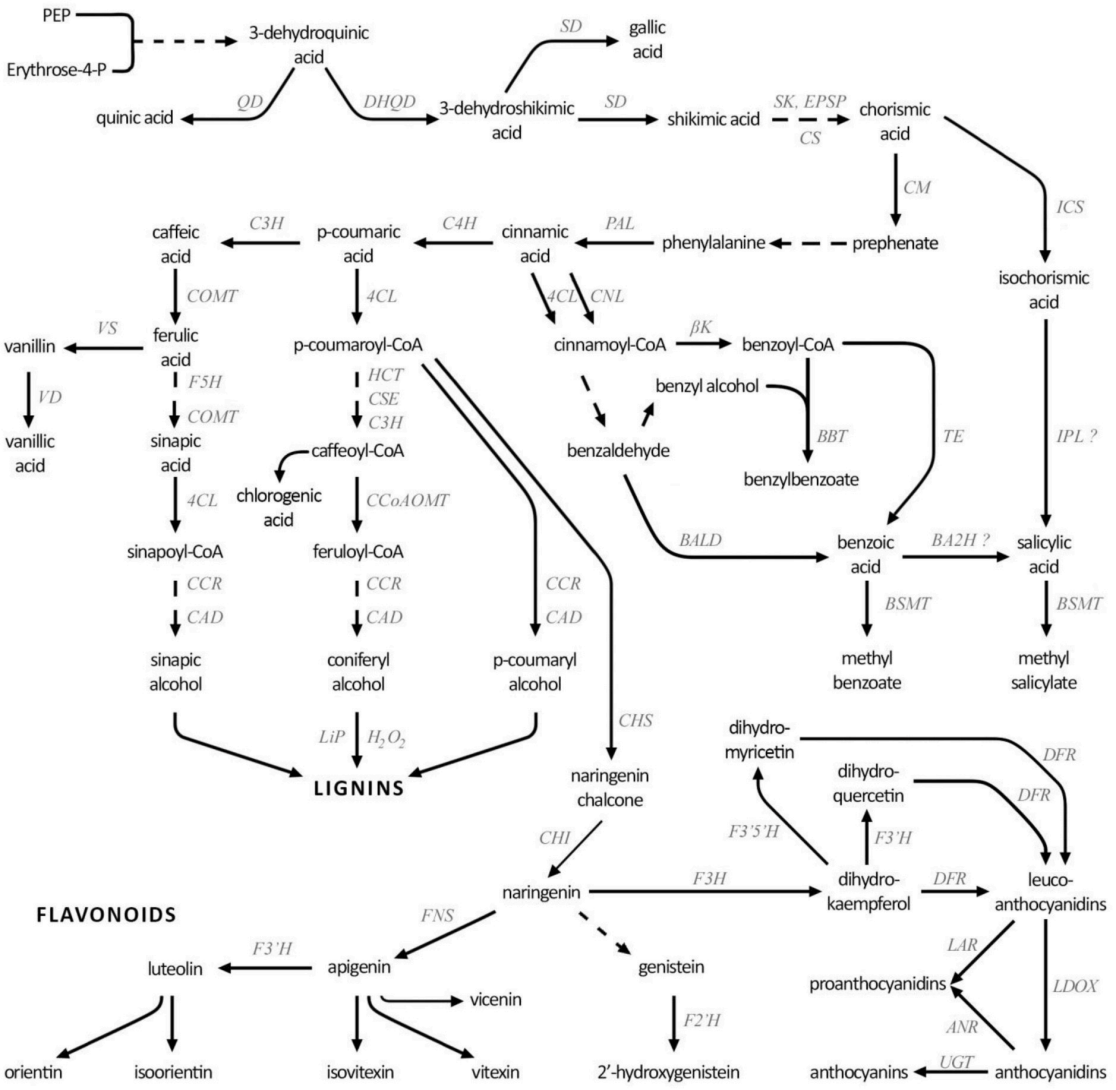
**Simplified phenolic compound biosynthesis pathway**. DHQD, 3-dehydroquinate dehydratase; QD, quinate dehydrogenase; SD, shikimate dehydrogenase; EPSP, 3-phosphoshikimate 1-carboxyvinyltransferase; SK, shikimate kinase; CS, chorismate synthase; CM, chorismate mutase; ICS, isochorismate synthase; IPL, isochorismate pyruvate lyase; PAL, phenylalanine ammonia lyase; C4H, cinnamic acid 4-hydroxylase; 4CL, 4-coumarate-CoA ligase; CNL, cinnamate-CoA ligase; βk, β-ketothiolase; TE, thioesterase; BALD, benzaldehyde dehydrogenase; BBT, benzyl alcohol O-benzoyltransferase; BA2H, benzoic acid 2-hydroxylase; BSMT, benzoic acid/salicylic acid methyltransferase; COMT, caffeic acid 3-O-methyltransferase; VS, vanillin synthase; VD, vanillin dehydrogenase; HCT, hydroxycinnamoyl-CoA:quinate/shikimate hydroxycinnamoyl transferase; CSE, caffeoyl shikimate esterase; C3H, 4-coumarate 3-hydroxylase; CCoAOMT, caffeoyl-CoA O-methyltransferase; F5H, ferulate 5-hydroxylase; CCR, cinnamoyl-CoA reductase; CAD, cinnamyl-alcohol dehydrogenase; LiP, lignin peroxidase; CHS, chalcone synthase; CHI, chalcone isomerase; F3H, flavanone 3-dioxygenase; FNS, flavone synthase; F3′H, flavonoid-3′-hydroxylase; F3′5′H, flavonoid 3′,5′-hydroxylase; DFR, dihydroflavonol reductase; LAR, leucoanthocyanidin reductase; LDOX, eucoanthocyanidin dioxygenase; ANR, anthocyanidin reductase; UGT, UDP-glucuronosyltransferase.

Plants have developed a number of mechanisms to counteract fungal attack, including passive mechanical barriers (cuticle, cell wall, stomatal apertures, lenticels) and chemical compounds (defensins, phytoanticipins), as well as active defenses (oxidative burst, cell wall reinforcement, antioxidants, phytoalexins, pathogen-related proteins). Upon infection, the plant employs a number of secondary metabolites in the defense against the pathogen. Among them phenolic compounds (phenolic acids, flavonoids, lignin, catecholamines, and benzoic derivatives) are known to play a role in many aspects of the plant's antifungal response (Lattanzio et al., 2006; Kostyn et al., 2012). Their biosynthesis starts in the shikimate pathway, which leads to production of phenylalanine—the first compound on the phenylpropanoid biosynthesis pathway (Figure 1). Non-oxidative deamination of phenylalanine catalyzed by phenylalanine ammonia lyase (PAL), the key-enzyme of this pathway, leads to cinnamic acid, which can be further transformed to *p*-coumaric acid by cinnamic acid 4-hydroxylase (C4H). The above-described core route is branched at several points, and each of the branches leads to a different group of compounds.

The benzoic acid derivatives, including salicylic acid, are produced in two different ways (Chen et al., 2009; Widhalm and Dudareva, 2015). In the first one, chorismic acid is transformed by isochorismate synthase (ICS) to isochorismate and then to salicylic acid (Garcion et al., 2008; Dempsey et al., 2011). It is likely that similar to the bacterial pathway, plants may convert isochorismate to salicylic acid via an isochorismate pyruvate lyase (IPL) enzyme. In *Arabidopsis thaliana ics* mutants salicylic acid synthesis induced by pathogen treatment was only 5–10% of that in the control with minimal induced SA made in a double *ics1ics2* mutant (Garcion et al., 2008). In the second way, cinnamic acid is converted to cinnamoyl-CoA by 4-coumarate-CoA ligase (4CL) in a non-oxidative route (Klempien et al., 2012) or by cinnamate-CoA ligase (CNL) in a β-oxidative route. The latter, which occurs in peroxisomes is followed by production of benzyl-CoA, catalyzed by β-ketothiolase, and further its conversion to benzoic acid. However, the gene or enzyme responsible for conversion of benzoic acid to salicylic acid has not been definitively identified. In a cytoplasmic non-oxidative route, cinnamoyl-CoA can also be converted to benzaldehyde, and then by benzaldehyde dehydrogenase (BALD) to benzoic acid, which is a precursor of salicylic acid. Methyl salicylate is a transport form of salicylate produced by benzoic acid/salicylic acid methyltransferase (BSMT) (Bonnemain et al., 2013; Russell et al., 2016).

*p*-Coumaric acid or its conjugate with coenzyme A, which is formed by 4-coumarate-CoA ligase, can be transformed to other phenolic acids or the phenolic acid-CoA conjugates, respectively. Hydroxycinnamoyl-CoA:quinate/shikimate hydroxycinnamoyl transferase (HCT) is considered the key enzyme in subsequent phenolic acid biosynthesis. They may serve in biosynthesis of many different compounds, including lignins—one of the cell wall polymers—with assistance of H_2_O_2_, peroxidases and possibly dirigent proteins (Hatfield, 2001; Mandal et al., 2011). Though ferulic acid is involved in lignin biosynthesis, it can also be a substrate in vanillin and further vanillic acid synthesis. The lignin biosynthesis route within the phenylpropanoid pathway diverges from other main route of flavonoid biosynthesis. Flavonoids are a large and very diversified group of phenolic derivatives of many various functions, including participation in plant reaction to abiotic and biotic stresses. Many flavonoids exists in the cell in form of glycosides, which have increased solubility and stability.

Benzenoid compounds participate in the plant's response to environmental stress either through their direct anti-microbial activity (Kocacaliskan et al., 2006), antioxidative properties (Dai and Mumper, 2010; Velika and Kron, 2012) or, in the case of salicylic acid, involvement in signaling pathways. After penetration of plant tissue by microbial pathogens, pathogen-associated molecular patterns (PAMPs), which generally are highly conserved molecules within a class of microbes that have an essential function in microbial fitness or survival [e.g., chitin, β-(1,3)-glucan], are recognized by specific receptors, which activate a number of defenses (PAMP-triggered immunity—PTI) (Zipfel and Robatzek, 2010). PTI can be suppressed by pathogen effectors, thus leading to successful colonization of the host. However, this can be repressed if the plant expresses a resistance protein (R) that recognizes the effector to induce effector-triggered immunity (ETI). During PTI and ETI various defense responses are activated. Salicylic acid (SA) is an important endogenous plant hormone signal in delivering the extracellular PAMP message into the plant cell to initiate the transcription of defense genes, including production of reactive oxygen species (ROS), and increased expression of pathogenesis-related (*PR*) genes (Tsuda et al., 2008; Vlot et al., 2009; Muthamilarasan and Prasad, 2013), as well as long-lasting, ample resistance to subsequent pathogen infection known as systemic acquired resistance (SAR) (Ali and Reddy, 2008). Salicylic acid (SA) is recognized a key signal for the activation of disease resistance in many plant species. Infection signal spreading, both to the healthy tissues of the infected plant or other neighboring plants occurs (in part) via a biologically inactive form of SA—methyl salicylate. After reaching its destination, this volatile molecule is converted back to SA thanks to the esterase activity of salicylic acid-binding protein 2 (SABP2) (Park et al., 2007).

Phenylpropanoid secondary metabolites also greatly contribute to plant pathogen resistance (Daayf et al., 2012). Several phenolic acids possess high antifungal activity. Ferulic acid was shown to suppress growth of *Fusarium verticillioides* and *Fusarium proliferatum* (Ferrochio et al., 2013) and inhibit the biosynthesis of *Fusarium* mycotoxins (Boutigny et al., 2009). Accumulation of phenolic compounds at the infection site also reinforces the cell wall, which is accompanied by localized production of ROS driving cell wall cross linking (Field et al., 2006). Biosynthesis of lignin, which contribute to the natural barrier against pathogens, is based on phenolic acids. In addition, phenolic acids and other phenylpropanoids (flavonoids) possess significant antioxidant properties (Khanam et al., 2012), and they significantly contribute to quenching of free radicals produced during the oxidative burst. This “extinguishing” of the initial plant response is important for example in the cases when some necrothrophs induce ROS production in the infected tissue to induce cell death that facilitates subsequent infection (Govrin and Levine, 2000). Flavonoids are involved in the inhibition of pathogen enzymes. It was shown that the anti-pathogenic effect of flavonoids depends on their structure. It was reported that the strongest antifungal activity is demonstrated by unsubstituted flavones and unsubstituted flavanones (Mierziak et al., 2014a).

The aim of this study was to evaluate the engagement of different branches of the shikimic acid/phenylpropanoid pathway in the early response of flax to *F. oxysporum* attack. As the shikimate/phenylpropanoid pathway produces a variety of compound species (benzoates, phenolic acids, lignin, flavonoids, etc.), their mutual relationships, ratios and interactions are of the highest relevance to the process of plant early stages of antifungal response. Our investigation of the transcript levels of genes involved in phenolic biosynthesis as well as the changes of phenolic compound levels has broadened the knowledge of its involvement in flax pathogen resistance.

## Materials and methods

### Plant material

Flax seeds (*L. usitatissimum* cv. Nike) were obtained from the Flax and Hemp Collection of the Institute of Natural Fibres in Poland. The seeds were germinated on Petri dishes containing Murashige and Skoog medium (Sigma-Aldrich) supplemented with 1% sucrose and solidified with 0.9% agar, under a 16 h light (21°C), 8 h darkness (16°C) regime.

### Infection tests

*F. oxysporum* was grown for 4 days at 18°C on potato/dextrose/agar (PDA) medium (Sigma- Aldrich). Fourteen day-old flax seedlings grown on MS medium were transferred, together with the medium, onto a PDA medium with *F. oxysporum*. PDA without fungus was used for a control. The flax seedlings were then collected after 6, 12, 24, 36, and 48 h and immediately frozen in liquid nitrogen and stored at −76°C before further experiments. The experiment was performed in three biological repeats. The whole experimental approach was then repeated and gave similar results.

### Identification of cDNA sequences

Unknown cDNA sequences of the flax genes of interest were identified based on homology alignments with the known gene sequences from other plant species(Clustal Omega, http://www.ebi.ac.uk/Tools/msa/clustalo/). The sequences were amplified in PCR reaction, where cDNA reverse transcribed from mRNA isolated from 14 day-old flax seedlings was used as a template. The primers were designed for the most homologous regions. The reaction product was analyzed via gel electrophoresis and after extraction from the gel using a DNA Gel-out Kit, it was cloned with a TOPO TA Cloning Kit (Invitrogen) and sequenced (Genomed SA, Poland). For verification the obtained DNA sequences were compared with the flax genome sequence (*L. usitatissimum* cv. Bethune) and aligned with corresponding genes from other plants in the GenBank database (http://www.ncbi.nlm.nih.gov/blast/).

### Analysis of gene transcript levels

Total RNA was isolated with the Trizol (Invitrogen) method according to the producer's protocol. The co-isolated DNA was removed by treatment with DNase I. The RNA was then used for genetranscript level analysis. It was transcribed to cDNA with a High Capacity cDNA Reverse Transcription Kit (Applied Biosystems), which was used in real time PCR (RT-PCR) technique using a DyNAmo SYBR Green qPCR Kit (Thermo Scientific) on the StepOnePlus™ Real-Time PCR System (Applied Biosystems) in triplicates. The conditions for the reactions were chosen in accordance with the producer's instructions. The primers were designed and are presented in Supplementary Table S1. For reference the actin gene was used. The differences in levels of transcripts were presented as relative quantification (RQ) to the reference gene and are presented in Supplementary Figure S1.

The isolated RNAs from tissue samples collected at 24 and 48 h after infection were also submitted to sequencing. Necessary sample preparations, sequencing and data processing was performed by an outsourcing company (Genomed SA, Poland). For both qRT-PCR and RNA_Seq, data from three biological replicates was analyzed.

After sequencing, the obtained transcript sequences were then aligned with the identified flax gene sequences of interest (Clustal Omega http://www.ebi.ac.uk/Tools/msa/clustalo/) and only transcripts with open reading frame, of which expression was higher than 2-fold or less than 0.5-fold (statistically significant at *p*-value ≤ 0.001) were further considered. Such sequences were used for phylogenetic tree preparation using the online ClustalW2 Phylogeny software (http://www.ebi.ac.uk/Tools/phylogeny/clustalw2_phylogeny/, Dist. Corr = off, Excl. Gaps = off, Clust. Meth. = Neighbour-joining, P.I.M. = off) and are presented in Supplementary Table S2. Sequences of the transcripts can be found in Supplementary Table S3.

### Determination of phenolic compound contents

Plant tissue collected after *F. oxysporum* infection tests was used for the determination of phenolic contents. 50 mg of the frozen tissue was ground in liquid nitrogen and extracted with 0.1% HCl in methanol followed by 15 min in ultrasonic bath incubation. The samples were centrifuged (12,000 g, 4°C, 10 min) and the supernatant was collected. The procedure was repeated twice, the supernatants were combined and then dried under nitrogen flow. The resulting pellet was re-suspended in 200 μl methanol and used in analysis of free metabolite contents. The remaining tissue after methanol extraction was used subjected to alkaline hydrolysis (2M NaOH at 37°C, overnight). After adjusting pH to 3 with concentrated HCl, two volumes of ethyl acetate were added and mixed. The mixture was then centrifuged at 12,000 × g, 4°C for 15 min. The ethyl acetate phase was collected. The extraction to ethyl acetate was repeated twice. The collected ethyl acetate volumes were combined and dried under nitrogen flow and re-suspended in 200 μl of methanol and then the samples were used for the determination of cell wall bound metabolite contents.

The methanol extracted and alkali-hydrolyzed samples were next analyzed with a Waters Acquity UPLC system with a 2996 PDA QTOF mass detector on an Acquity UPLC BEH C18 (2.1 × 100 mm, 1.7 μm) column. The mobile phase was passed through the column at a flow rate of 0.4 ml/min. The mobile phase consisted of the following components: Solvent A, 0.1% formic acid; and solvent B, 100% acetonitrile. For the first minute, isocratic elution was carried out using 95% of A in B. From 2 to 12 min, a linear gradient was applied using 95 to 70% of A in B. From 13 to 17 min, a linear gradient was applied using 70 to 0% of A in B. In the final minute concentration of A returned to 95%. The column was kept at 25°C. A photodiode array (PDA) was used to detect absorption between 210 and 500 nm. The MS spectra were recorded in ESI positive mode for 17 min in the 50–800 Da range. The parameters were: Nitrogen flow: 800 L/h, source temperature: 70°C, desolvation temperature cone: 400°C, capillary voltage: 3.50, sampling cone: 30, cone voltage: Ramp 40–60 V, scan time: 0.2 s. The identities of components were determined based on either their retention times, UV and mass spectra comparison to authentic standards (Sigma-Aldrich, USA) or for compound derivatives (including glycosides)—based on UV and MS spectra.

### Determination of salicylic acid and methyl salicylate contents

5 g of frozen tissue was ground in liquid nitrogen and extracted with methanol chloroform (8:2) for 1 h at room temperature (on shaker), 50 ng of mandelic acid was added as an internal standard to each sample, then centrifuged (10 min, 12,000 g). The supernatants were collected and evaporated under nitrogen flow. The residues were supplemented with 200 μl of BSTFA:TMCS 9:1, vortexed for 1 min and incubated at 120°C for 60 min. (Huang et al., 2015). After derivatization, sample was vigorously mixed and injected directly to GC/MS. A Shimadzu QP-2020 gas chromatograph coupled with single quadrupole mass spectrometer system (Shimadzu, Japan) was used in electron impact (EI) with electron energy 70 eV. Samples were separated by use of a 30 m × 0.25 mm × 0.25 μm film thickness ZB-5MSi capillary column from Phenomenex, USA. Samples (1 μl) was inject in split mode (1:10). Oven program was set to 40°C for 0 min hold then increased at 20°C/min rate to 300°C for 5 min hold (total time 18.00). Helium (99,9999%) was used as mobile phase with linear velocity of 36.1 cm/s, total flow 14 ml/min. Mass spectrometer was used in SIM mode monitoring 5 channels: 267, 209, 179, 147, 253 from 5.00 min to 18.00 min at 10,000 scans/s. Temperature of transfer line was 300°C and ion source 220°C. Contents of salicylic acid and methyl salicylate were determined based on original standards (Sigma-Aldrich, USA). We were unable to detect salicylic acid glucosides (TransMIT GmbH, Germany) in the studied samples.

### Determination of lignin content

Total lignin content was determined by the acetyl bromide method (Iiyama and Wallis, 1990). Briefly, 100 mg of flax seedling tissue was heated at 100°C for 2 h. Then, 10 ml of H_2_O was added and the samples were heated at 65°C for an additional hour with mixing every 10 min. The samples were filtered through GF/A 24 mm filters (Whatman) and the filtrates were washed three times with H_2_O, ethanol, acetonitrile, and diethyl ether in that order. The filters were then placed in glass vials and heated at 70°C overnight. Subsequently, 2.5 ml of 25% (v/v) acetyl bromide in 80% acetic acid were added and the samples were incubated at 50°C for 2 h. The samples were then cooled down and 10 ml of 2M NaOH and 12 ml of 80% acetic acid were added. After overnight incubation, the lignin content was determined spectrophotometrically at λ = 280 nm. Coniferyl alcohol was used for standard curve preparation. The assays were prepared in three biological repetitions.

### Determination of cellulose content

Cellulose content was determined with the anthrone method described by Ververis et al. (2004). Plant tissue (100 mg) was ground in liquid nitrogen and incubated with a mixture of 65% nitric acid and 80% acetic acid (1:8 vol.) with heating at 100°C for 1 h. After that the samples were centrifuged [5 min, 12,000 × g, room temperature (RT)] and the pellet was washed with water twice, dissolved in 1 ml of 67% H_2_SO_4_ (v/v) and incubated at RT for 1 h with shaking. Then the samples were diluted and 100 μl were added to 900 μl of cooled 0.2% anthrone in 67% H_2_SO_4_, mixed and heated at 100°C for 15 min. Next the samples were cooled down and the level of cellulose was detected spectrophotometrically at 620 nm. Commercial cellulose (Sigma-Aldrich, USA) was used for standard curve preparation. The assays were prepared in three biological repetitions.

### Assessment of influence of selected phenolic compounds on *Fusarium oxysporum* growth

In order to determine the direct influence of selected phenolic compounds on the growth of *F. oxysporum*, PDA media supplemented with standard solutions of salicylic acid, vanillin, vanillic acid, *p*-coumaric acid, caffeic acid, ferulic acid, orientin, isoorientin, vitexin, and isovitexin in three concentrations (100, 50, 10 μM) were prepared. PDA medium with appropriate amounts of the solvent, in which the standard compounds were dissolved, were used for the control. *F. oxysporum* mycelium fragments were grown for 48 h on Petri dishes with the media prepared in this way. Next the mycelia were photographed and their surfaces were measured.

### Statistical analysis

Statistical analyses were performed using Statistica 10 software (StatSoft, USA). The significance of differences between the means was determined using Student's *t*-test for independent samples. The significance of differences in transcript level after RNA sequencing were determined with Fisher's exact test.

## Results

### Transcript levels of selected genes of shikimate/phenylpropanoid/benzoate pathway in flax after *Fusarium oxysporum* treatment

The initial screening of the shikimate/phenylpropanoid gene expression in 2 week old flax seedlings treated with *F. oxysporum* by means of qRT-PCR method revealed substantial alterations in the expression pattern (Supplementary Figure S1). As several of those genes have several isoforms, we performed transcriptome sequencing to obtain a broader and more detailed image of the changes observed in our initial experiment. Several transcript sequences with homology to the genes connected with phenolic compound synthesis were found after whole transcriptome sequencing of RNA isolated from flax plants after 24 and 48 h post infection with *F. oxysporum* (hpi). The results were obtained based on three biological replicates. The genes were divided into groups according to the route they participate in. Whole transcriptome data analysis allowed for the determination of transcript levels. Only transcripts with open reading frame, homologous to the investigated genes, whose expression was more than 2-times higher or less than 2-times lower (statistically significant at *p*-value ≤ 0.001) were considered (Table 1). Transcripts of genes involved in the core route of the shikimate/phenylpropanoid pathway were generally more abundant at 24 hpi, with the highest increase measured for *PAL* (up to 8.21-fold of the control). At 48 hpi the activation of these genes receded. Only for shikimate dehydrogenase gene (*SD*), transcript levels were considerably lower at 48 hpi compared to the control. Increase of transcript levels of three genes of benzoate route: 3-ketoacyl-CoA thiolase 2 (β-ketothiolase), benzoate/salicylate carboxymethyltransferase (*BSMT*), and benzyl alcohol O-benzoyltransferase (*BBT*), was measured. The *BSMT* gene activation is especially worthy of note. Abundance of transcripts of this gene reached 9.1- and 18.8-fold of the control at 24 and 48 hpi, respectively. Genes of lignin biosynthesis route were up-regulated at 24 hpi, but this activation subsided and even decreases in transcript levels compared to the control were observed at 48 hpi. Increased transcription of genes involved in flavonoid biosynthesis was observed both at 24 and 48 hpi. Considerable activation of the key gene of this pathway—chalcone synthase (*CHS*) (up to 75-fold of the control) was noted at 48 hpi. Activation of leucoanthocyanidin dioxygenase (*LDOX*) gene at 24 hpi (76.6-fold of the control) and at 48 hpi (up to 13.5-fold of the control) was observed, while expression of *ANR* gene, involved in flavanol and non-hydrolysable tannin biosynthesis was down-regulated both at 24 and 48 hpi.

**Table 1:** **Transcript levels of genes of shikimate/phenylpropanoid/benzoate pathway measured in 24 and 48 h after infection with *F. oxysporum* presented as fold of the non-treated control; “–” symbol stands for no change in the transcript expression or change in the range between 0.5 and 2-fold of the control**.

**Pathway**	**Gene**	**Acc. number**	**X-fold of the control**	
			**24 h**	**48 h**
CORE ROUTE	Shikimate dehydrogenase (*SD*)	AFSQ01026985	−	0.08
		AFSQ01002620	−	0.36
	Chorismate synthase (*CS*)	AFSQ01008633	2.23	−
	Chorismate mutase (*CM*)	AFSQ01018652	2.04	−
	Phenylalanine ammonia lyase (*PAL*)	AFSQ01008390/91	8.21	−
		AFSQ01000737	5.45	−
	Trans-cinnamate 4-monooxygenase (*C4H*)	AFSQ01009050	2.23	−
		AFSQ01011791	4.62	−
BENZOATE ROUTE	3-ketoacyl-CoA thiolase 2 (β-ketothiolase)		3.19	3.1
	Benzoate/salicylate carboxymethyltransferase (*BSMT*)	AFSQ01004321	7.97	18.83
		AFSQ01007680	3.43	9.94
		AFSQ01004321	9.1	−
		AFSQ01026389	6.27	−
		AFSQ01007680	2.83	−
	Benzyl alcohol O-benzoyltransferase (*BBT*)	AFSQ01022016	5.33	−
LIGNIN ROUTE	4-coumarate-CoA ligase (*4CL*)	AFSQ01020997	3.8	−
		AFSQ01023785	2.8	−
		AFSQ01020997	4.41	−
		AFSQ01013215	0.04	0.09
		AFSQ01014596	−	0.45
		AFSQ01012878	−	2.03
	Hydroxycinnamoyl-CoA:quinate/shikimate Hydroxycinnamoyl transferase (*HCT*)	AFSQ01015754	2.97	3.27
		AFSQ01006651	2.17	−
	Caffeoyl shikimate esterase (*CSE*)	AFSQ01017841	2.77	0.12
		AFSQ01011162	0.29	0.41
	Caffeic acid 3-O-methyltransferase (*COMT*)	AFSQ01002471	2.99	−
		AFSQ01027305	−	0.08
		AFSQ01024646	−	0.18
	Caffeoyl-CoA O-methyltransferase (*CCoAOMT*)	AFSQ01027261	3.9	−
	Cinnamoyl-CoA reductase (*CCR*)	AFSQ01007861	2.1	−
		AFSQ01010549	2.72	−
		AFSQ01011029	10.2	−
	Cinnamyl alcohol dehydrogenase (*CAD*)	AFSQ01005963	3.46	0.44
FLAVONOID ROUTE	chalcone synthase (*CHS*)	AFSQ01005163	4.7	−
		AFSQ01007984	4.25	74.94
		AFSQ01007984	−	75.33
		AFSQ01007986	−	0.01
	Flavanone 3-hydroxylase (*F3H*)	AFSQ01016891	6.02	2.57
		AFSQ01009941	0.02	−
		AFSQ01025118	−	2.49
		AFSQ01022073	−	10.29
	Isoflavone 2′-hydroxylase (*F2′H*)	AFSQ01000408	20.57	3.7
		AFSQ01007109	0.23	0.4
	Flavonoid 3′-hydroxylase/monooxygenase (*F3′H*)	AFSQ01028139	3.99	−
		AFSQ01019455	−	36.51
		AFSQ01008368/69	−	0.34
	Flavonoid 3′,5′-hydroxylase (*F3',5′H*)	AFSQ01012300	2.34	−
	leucoanthocyanidin dioxygenase (*LDOX*)	AFSQ01021358	76.64	13.54
		AFSQ01012389	−	5.11
	Anthocyanidin reductase (*ANR*)	AFSQ01023196	0.36	0.27
	Udp-glucosyltransferase (*UGT*)	AGD95009	38.39	−

*Presented results are statistically significant at p < 0.001 and based on three biological replicates. Accession numbers of the sequences of isoforms are provided*.

### Metabolite changes in flax after *Fusarium oxysporum* treatment

We investigated whether the changes in gene transcript levels after *F. oxysporum* treatment were reflected in corresponding metabolite contents. Therefore, methanol extracts (free metabolites) and extracts of cell wall bound compounds (released after alkaline hydrolysis) from plants at 24 and 48 h after treatment with *F. oxysporum* were analyzed with the UPLC-MS technique. For salicylic acid and methyl salicylate contents GC-MS method was employed (Table 2).

**Table 2:** **Contents of free and cell wall bound metabolites in extracts prepared from plants at 48 h after *F. oxysporum* treatment (mean values of three biological repeats ± standard deviations)**.

**Metabolite**		**Metabolite content**			
		**24 h**		**48 h**	
		**mean (μg/g FW)**	***SD***	**mean (μg/g FW)**	***SD***
**FREE COMPOUNDS**					
Salicylic acid	Control	0.199	0.062	0.19	0.045
	*F. oxysporum*	0.177	0.049	0.156	0.084
Methyl salicylate	Control	0.138	0.071	0.177	0.065
	*F. oxysporum*	0.075	0.081	0.744^***^	0.051
3-O-caffeoylquinic acid (chlorogenic acid)	Control	156.6	21.3	134.9	8.1
	*F. oxysporum*	114.1^*^	18.8	68.9^*^	21.3
O-caffeoylquinic acid (chlorogenic acid isomer)^$^	Control	131.2	3.2	146.4	6.8
	*F. oxysporum*	144.8	15.4	81.3^***^	16.5
Caffeic acid	Control	271.2	8.1	254.5	7.3
	*F. oxysporum*	288.7	21.6	315.12	44.8
Caffeic acid derivative^$^	Control	1413.2	27.1	1501.4	49.1
	*F. oxysporum*	1193.6^***^	38.2	1175.5^***^	14.6
Ferulic acid	Control	107.7	4.7	111.6	5.8
	*F. oxysporum*	132.2^**^	3.6	178.4^***^	3.8
Ferulic acid derivative^$^	Control	31.8	1.4	17.6	4.1
	*F. oxysporum*	25.5^*^	3.5	28.0	11.5
Vicenin	Control	866.3	81.9	761.4	18.2
	*F. oxysporum*	1462.4^***^	32.8	1683.0^***^	60.6
Isoorientin	Control	271.6	9.6	255.4	8.0
	*F. oxysporum*	258.2	12.7	268.2	12.4
Vitexin	Control	38.0	1.2	29.0	27.0
	*F. oxysporum*	36.7	13.2	37.0	7.0
Isovitexin	Control	101.7	9.7	102.3	6.7
	*F. oxysporum*	95.0	21.7	156.9^***^	13.4
Quercetin diglycoside^$^	Control	21.4	1.0	25.8	0.8
	*F. oxysporum*	19.4^**^	1.8	20.2^*^	2.0
Apigenin diglycoside^$^	Control	27.2	1.5	27.1	0.6
	*F. oxysporum*	21.6	2.1	24.4^***^	2.2
Apigenin diglycoside 2^$^	Control	216.3	12.5	164.4	16.4
	*F. oxysporum*	207.1	9.1	227.8^**^	11.1
**CELL WALL BOUND COMPOUNDS**					
Coumaric acid derivative^$^	Control	7.2	2.6	9.1	2.9
	*F. oxysporum*	4.6	1.0	16.6^*^	6.7
Vanillic acid	Control	8.3	0.7	9.7	1.6
	*F. oxysporum*	8.3	0.6	9.4	0.4
4-hydroxy-benzaldehyde	Control	9.5	1.0	9.4	0.3
	*F. oxysporum*	7.3^*^	0.6	9.5	0.5
Vanillin	Control	35.1	6.6	38.8	1.7
	*F. oxysporum*	30.5	5.4	38.6	3.4
*p*-coumaric acid	Control	22.6	2.3	19.2	3.4
	*F. oxysporum*	13.4^***^	1.0	15.5	1.3
Ferulic acid	Control	101.5	5.8	85.7	10.6
	*F. oxysporum*	64.2^***^	3.0	68.4^*^	4.1
LIGNIN	Control	20.9	1.3	21.3	0.9
	*F. oxysporum*	18.9	2.9	17.4^*^	2.4
CELLULOSE	Control	17.5	1.4	17.9	1.3
	*F. oxysporum*	18.7	0.8	21.7^*^	2.7

Statistically significant differences are marked (

* *for p < 0.05*,

** for p < 0.01 and

*** *for p < 0.001). Compounds marked with $ are derivatives, whose concentrations are presented in equivalents of corresponding metabolites*.

Among the free metabolites a significant increase in methyl salicylate content (4.8-fold of the control) was observed at 48 hpi. No significant changes in the level of its precursor, salicylic acid were measured. The remaining metabolites in the soluble metabolite extracts could be divided into two groups: Phenolic acids and flavonoids. Content of caffeic acid derivative—the most abundantly represented phenolic acid decreased by 15.5 and 21.7% at 24 and 48 hpi, respectively. Also, levels of chlorogenic acids dropped down at both studied time points after infection (by 27.1 to 48.9%). Ferulic acid content increased both at 24 and 48 hpi, by 22.7 and 59.86%, respectively. Total flavonoid content increase was observed, by 36.2 and 77.1% at 24 and 48 hpi, respectively. Vicenin was found to be the most abundant compound of this type, and it increased by 68.8 and 121% at 24 and 48 hpi, respectively. Contents of isovitexin and one of apigenin diglycosides increased at 48 hpi by 53.4 and 38.6%. Decrease in the contents of quercetin diglycoside and one of apigenin diglycosides ranged from 9.3 to 21.7% below the control. Among cell wall bound compounds p-coumaric acid content was shown to be dropped down by 40.7% at 24 hpi compared to the control, while its derivative level increased by 82.4% at 48 hpi. Content of ferulic acid decreased by 36.7 and 20.2% below the control, at 24 and 48 hpi, respectively. A 23.2% decrease in 4-hydroxybenzaldehyde at 24 hpi was measured. An independent experimental approach gave similar results (data not included).

Lignin and cellulose contents in flax after *Fusarium* treatment were measured. For lignin, a decrease by 17% and for cellulose an increase by 21% was observed in plants at 48 hpi relatively to the control.

### Influence of selected phenolic compounds on *Fusarium oxysporum* growth

The influence of selected phenolics on growth of *F. oxysporum* was investigated. The results are presented in Table 3. Most of the used compounds showed an inhibitory effect on fungal growth, though the influence differed according to the concentrations that was used. Among benzoate derivatives the most profound effect was observed for vanillin at 0.1 mM concentration (0.5-fold of the control). Vanillic acid in concentration of 0.01 mM led to inhibition of *F. oxysporum* growth to 0.64-fold of the control. Also, phenolic acids turned to be efficient inhibitors of *Fusarium* growth, even at low concentrations. Strong growth inhibition was observed for flavonoids, vitexin, and isovitexin, though low concentrations of these compounds did not exert an inhibiting effect on *Fusarium* growth. Interestingly, 0.05 mM orientin impeded the growth of the fungus, while higher concentration did not exert such an effect.

**Table 3:** **Area of *Fusarium oxysporum* growth on PDA medium supplemented with standard solutions in 0.1, 0.05, and 0.01 M concentrations, measured at 48 h after medium inoculation, presented as fold of the control growth (means of three biological repeats ± standard deviations)**.

**Compound**	**Concentration**		
	**0.1 mM**	**0.05 mM**	**0.01 mM**
Salicylic acid	1.06 ± 0.03	1.05 ± 0.12	0.98 ± 0.05
Vanillin	0.5 ± 0.01*	0.84 ± 0.11	0.67 ± 0.08*
Vanillic acid	0.58 ± 0.05*	0.79 ± 0.08	0.64 ± 0.04*
*p*-coumaric acid	0.61 ± 0.04*	0.76 ± 0.08	0.6 ± 0.09*
Caffeic acid	0.5 ± 0.04*	0.76 ± 0.1	0.66 ± 0.05*
Ferulic acid	0.45 ± 0.1*	0.68 ± 0.06	0.67 ± 0.05*
Orientin	0.86 ± 0.15	0.63 ± 0.07*	0.82 ± 0.15
Isoorientin	0.94 ± 0.07	0.8 ± 0.09	0.84 ± 0.16
Vitexin	0.38 ± 0.02*	0.92 ± 0.14	0.97 ± 0.09
Isovitexin	0.32 ± 0.05*	0.86 ± 0.23	0.91 ± 0.16

*Statistically significant differences are marked with asterisks (p < 0.05)*.

## Discussion

**Figure 2:**
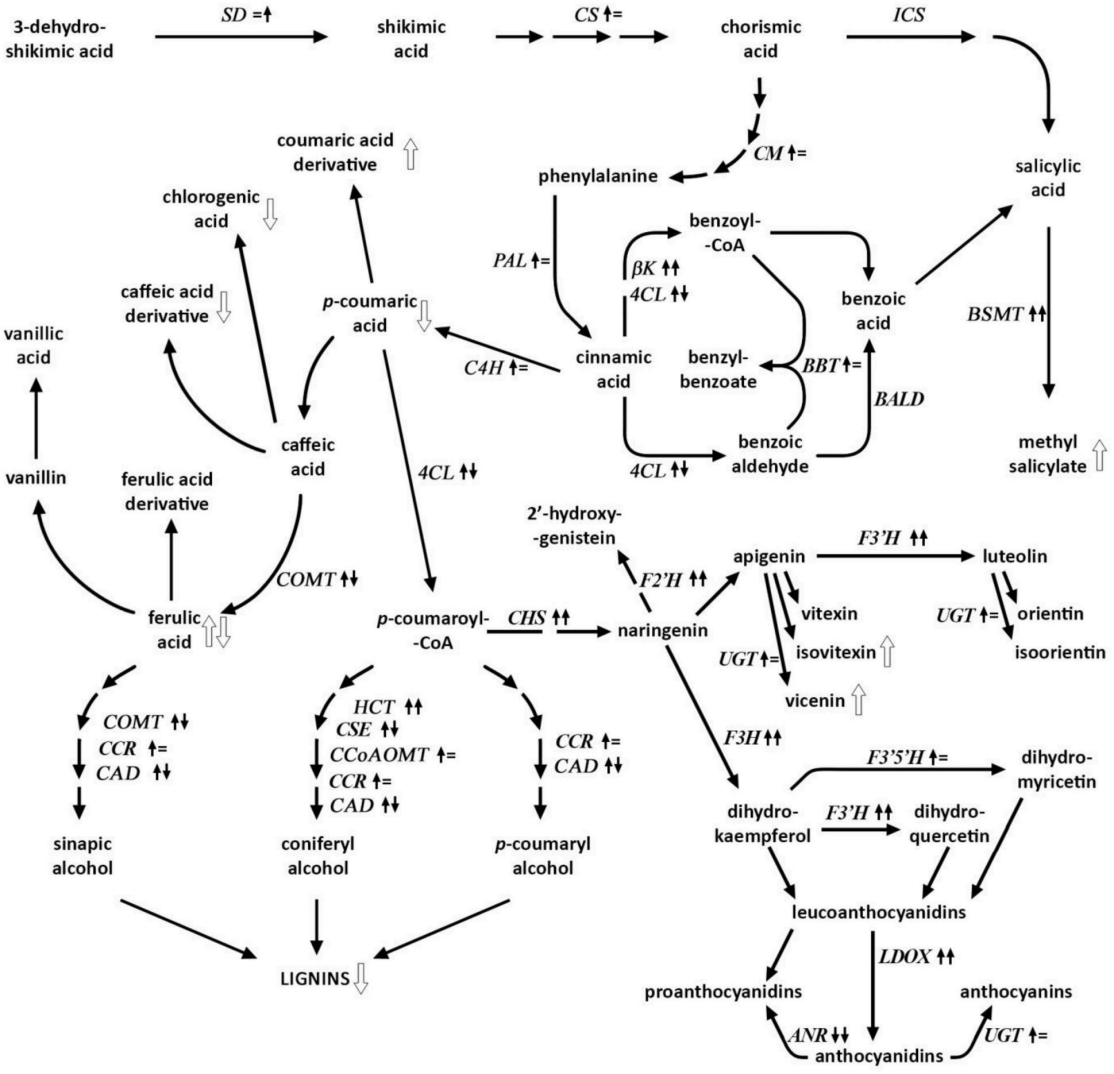
**A scheme of phenylpropanoid pathway**. Genes for which transcript levels changed are marked with up/down arrows or equal sign when no change or change in the range between 0.5 and 2-fold occurred (for 24 hpi and 48 hpi). Changes in metabolite contents are marked with white up/down arrows.

Flax is an appreciated source of oil and fiber used in many branches of industry, ranging from food, textiles and medicine to chemicals, cosmetics, and construction, but unfortunately it is the target for fungal attacks, with *F. oxysporum* being one of the most dangerous flax-specific pathogens causing the most crop loss. Plants have evolved numerous defense mechanisms against pathogen infections, and even though they have been extensively studied, we still lack knowledge on several aspects of plant resistance. Initiation of a resistance response requires perception of signal molecules, either synthesized by the invading pathogen or released from damaged plant cell walls, which turns on the activation of pre-existing components, as well as generation of active agents, such as ROS (Lehmann et al., 2015). Whether ROS would function as signaling molecules or could cause oxidative damage to the tissues depends on the subtle balance between production and scavenging of ROS (Sharma et al., 2012). One of the molecules important for providing this balance is salicylic acid (SA), involved in the regulation of both ROS production and synthesis of antioxidants, mostly derived from the phenylpropanoid pathway (Mandal et al., 2009). Phenylpropanoids possess high antioxidant capacity (Korkina, 2007) and their production occurs relatively early during the plant response to the pathogen (Kostyn et al., 2012). The phenolic derivatives originate from the shikimate pathway. The early genes of this pathway are known to be up-regulated in response to pathogen infection in concert with *PAL*, the key gene in phenolic compound (lignin, benzoates, flavonoids) synthesis (Lazar, 2005). In flax treated with *F. oxysporum* we have measured high induction of *PAL* and trans-cinnamate 4-monooxygenase (*C4H*), the following gene on the pathway, at 24 hpi. This in conjunction with the unchanged transcript level of the *ICS* gene and elevated transcript level of the β-ketothiolase (β*K*) gene along with unchanged *BALD* gene transcript level led us to the suggestion that in flax, at least at early stages of infection activation of oxidative transformation of the cinnamic acid controls SA production manifested as MeSA. These results are in opposition to data from *Arabidopsis*, where *ICS* was shown to be a key gene responsible for SA biosynthesis (Wildermuth et al., 2001; Garcion et al., 2008) and from other plants in which the isochorismate pathway is the dominant pathway for induced SA synthesis (Dempsey et al., 2011). However, no increase in SA content was observed in our experiments. This may be due to its immediate processing to its methylated form—methyl salicylate (MeSA). We have observed almost 5-fold increase in this compound content in the infected seedlings. This correlated well with a considerable activation of benzoate/salicylate carboxymethyltransferase (*BSMT*) gene, which catalyses the transformation of SA to MeSA (Chen et al., 2003). It was shown before in a number of reports that a reduced *PAL* gene transcript level correlates with decreased SA levels and increased plant susceptibility to pathogen infection (Huang et al., 2010). It is known that *PAL* and other genes of the phenylpropanoid pathway (e.g., *4CL, CHS*) are activated upon induction with both fungal and cell wall elicitors (Davis and Hahlbrock, 1987; Lawton and Lamb, 1987; Xue et al., 2014). In elicited flax up-regulation of *PAL*, cinnamoyl-CoA reductase (*CCR*), and cinnamyl alcohol dehydrogenase (*CAD*) was reported (Hano et al., 2006; Kostyn et al., 2012). In our study we also observed an increase in the transcript level of genes connected to lignin biosynthesis. Transcript level of chalcone synthase (*CHS*) gene was elevated at 24 hpi and considerably higher at 48 hpi. Flavonoids can be converted into glucosylated forms by either C-glucosyltransferase or O-glucosyltransferase. Both types of glycosides, which can be derived from apigenin and luteolin, were measured in higher amounts in flax after fungal infection, which corresponds to the increased glucosyltransferase gene transcript level. It is well established that flavonoids have high anti-pathogenic properties (Mierziak et al., 2014b). Overexpression of glucosyltransferase in flax and *Arabidopsis* was shown to protect plants against infection (Lorenc-Kukula et al., 2009; Shin et al., 2012). In our experiments, the elevation of glucosylated forms of flavonoids was correlated with the transcript level of *CHS* gene. The observed discrepancy between activation of transcription of lignin route associated genes and the lignin level could be explained by the competition for substrates. It is known that lignin biosynthesis is inversely related to flavonoid production (Laffont et al., 2010). Zuk et al. (2011) reported on decreased lignin content in transgenic flax plants with overproduction of flavonoids. The concept that the flavonoid and lignin routes could be organized as enzyme complexes was first proposed by Stafford (1974) as an explanation of mutual competition for common intermediates. Silencing of hydroxycinnamoyl-CoA:quinate/shikimate hydroxycinnamoyl transferase (*HCT*) gene expression led to the redirection of the metabolic flux into flavonoids through CHS activity in *Arabidopsis* (Besseau et al., 2007) and in alfalfa (Gallego-Giraldo et al., 2011a). A similar effect was obtained in plants with a silenced 4-coumarate 3-hydroxylase (*C3H*) gene (Li et al., 2010). Formation of specific enzyme complexes might force metabolite flux toward flavonoids even in the presence of excess lignin biosynthesis enzymes. In our experiments transcript levels of genes of lignin synthesis were elevated, while we observed decreased levels of lignin accompanied by higher cellulose contents. The other possibility for the lower lignin content may be in fact connected with the pathogen attack itself and digestion of the cell wall (Rodriguez et al., 1996). In such a scenario, release of elicitors from the cell wall would induce SA synthesis directly (Gallego-Giraldo et al., 2011b) or through ROS. It has been reported that the level of salicylic acid (SA) increased after apoplastic H_2_O_2_ bursts mediated by NADPH oxidases and extracellular peroxidases (O'Brien et al., 2012; Mammarella et al., 2015), though in this case a biosynthesis route involving ICS was implicated (Herrera-Vásquez et al., 2015), however, we did not see ICS induction at any time point examined, but cannot rule out its participation in the flax response. In consequence, SA and/or its methylated form—MeSA would lead to the induction of lignin biosynthesis as PAL-dependent lignification was shown to be induced by SA and MeSA (He and Wolyn, 2005; Mandal, 2010), which is involved in plant defense signaling (Park et al., 2007), leading to SAR and among other things to induction of lignin formation (Mandal, 2010). A simplified scheme of the phenylpropanoid pathway with marked changes in the metabolite contents and transcript levels is presented in Figure 2.

In accordance with the results, a metabolite flux between different routes of the phenylpropanoid pathway occurs at the early stages of *F. oxysporum* infection of flax. The destabilization of equilibrium in phenolic biosynthesis leads to the induction of the plant response, which is most important in the earliest stages of the infection—defense signal preparation (SA, MeSA), redirection of substrates and activation/deactivation of particular metabolic routes, production of antioxidants, which are important regulators of ROS level. Restitution of cell wall integrity (lignin) occurs probably at subsequent steps of the plant's response. Induction of *PAL* and β-ketothiolase genes suggests that in *Fusarium*-infected flax the biosynthesis of salicylic acid (SA) is performed mainly via the PAL-dependent route. As we observed no statistically significant increase in *ICS* gene transcript level at 48 h after infection (Supplementary Figure S1), it is possible that this route is activated at the later stages of the plant response. Confirmation of this suggestion requires, however, further studies involving gene knock-out experiments or specific inhibitors. Moreover, future studies should be focused on determining whether and to what extent manipulation of a redundant or adverse metabolic route within a pathway will favor other, advantageous ones.

## Author contributions

AB performed infection tests and gene expression analysis, participated in RNAseq analysis. KK performed metabolite analysis and wrote the manuscript. AKo investigated the influence of phenolics to fungal growth, participated in writing the manuscript. WW performed gene expression analysis. MD performed GC-MS analysis. MP participated in RNAseq analysis. JS coordinated the study, participated in data analysis. AKu planned the experiments and participated in writing the manuscript.

### Conflict of interest statement

The authors declare that the research was conducted in the absence of any commercial or financial relationships that could be construed as a potential conflict of interest.
