# Supplementary material for: Methyl Salicylate Level Increase in Flax after Fusarium oxysporum Infection Is Associated with Phenylpropanoid Pathway Activation
Source: Front Plant Sci. 2017 Jan 20;7:1951. doi: 10.3389/fpls.2016.01951 (PMC5247452; doi:10.3389/fpls.2016.01951)
Supplement: Supplementary file 1 [file Table1.DOCX]

Supplementary Table S1. Sequences of primers used in RT-PCR analysis.

| **name** | **forward primer** | **reverse primer** |
| --- | --- | --- |
| shikimate dehydrogenase (*SD*) | TGGTAGCAGTCAATCTGGTT | CCTCCAATTATGTCAGCAAG |
| chorismate synthase (*CS*) | CGCCAATCCATGTGTTCGTA | TTTCTCTTGCAGAAGATCGG |
| phenylammonia lyase (*PAL*) | GTTCTGTTTGAAGCCAATGT | TGTAAGCACTCCCGTCG |
| trans-cinnamate 4-monooxygenase (*C4H*) | CTTTCATCAACAAAGTAGTCCTTG | TGGTGAACACTGGAGGAAAATG |
| isochorismate synthase (*ICS*) | ATCGCTTGGGATAATGACC | CACAAGGCTTAATAGGAGAGTG |
| β-ketothiolase (*βK*) | CCCATTCTTGGCGTGTTCAGG | CATTATAGAGCTCGTCGACACA |
| benzaldehyde dehydrogenase (*BALD*) | CTCGAGACATGGGACAA | CTCCAATCGGTTCGTGTAAT |
| benzoic/salicylic acid methyl transferase (*BSMT*) | CGGCAACATTTATATGGCTAGTA | GATCGCTTCTTCTCCCG |
| vanillin synthase (*VS*) | ACCAATAGAAAGGGATTGCCTTA | CGGAAGGATAGCATCGG |
| 4-coumarate-CoA ligase (*4CL*) | GCAGAAATGAAGATCGTCG | GTATGTAACCACCCTTGCT |
| 4-coumarate 3-hydroxylase (*C3H*) | CCATTGGAGTTCAAACCAGA | AGCAAGTGTCCCAACATAG |
| hydroxycinnamoyl-CoA:quinate/shikimate hydroxycinnamoyl transferase (*HCT*) | GTCGATATTCAAGCTGACCC | GTGGCGATGTACAGTTTAGT |
| caffeoyl-CoA O-methyltransferase (*CCoAOMT*) | CGGACAAGGACAACTACAT | ACGAAGTCCCTGTAGTACCTAA |
| caffeic acid 3-O-methyltransferase (*COMT*) | CTCTTGGCTTCTTACTCTGTT | TGAGGACTTTGTCCTGGTT |
| cinnamyl alcohol dehydrogenase (*CAD*) | GGAGCATGAAGGAAACAGAG | CAACATCAACCACGAACCTA |
| chalcone synthase (*CHS*) | AAATGGGGAGAATGGAAGGA | CGCACGATTCAAATAGTGAGA |
| glucosyl transferase (*GT*) | ATGCAGTGTGCATTCCAT | CGAATCGGAACGAAGGTAG |
