# Supplementary material for: Methyl Salicylate Level Increase in Flax after Fusarium oxysporum Infection Is Associated with Phenylpropanoid Pathway Activation
Source: Front Plant Sci. 2017 Jan 20;7:1951. doi: 10.3389/fpls.2016.01951 (PMC5247452; doi:10.3389/fpls.2016.01951)
Supplement: Supplementary file 2 [file Table2.docx]

Supplementary Table S2. Phylogenetic trees of the studied genes. Organism names, sequence accession numbers and distances are included.

| shikimate dehydrogenase (*SD*) |
| --- |
| 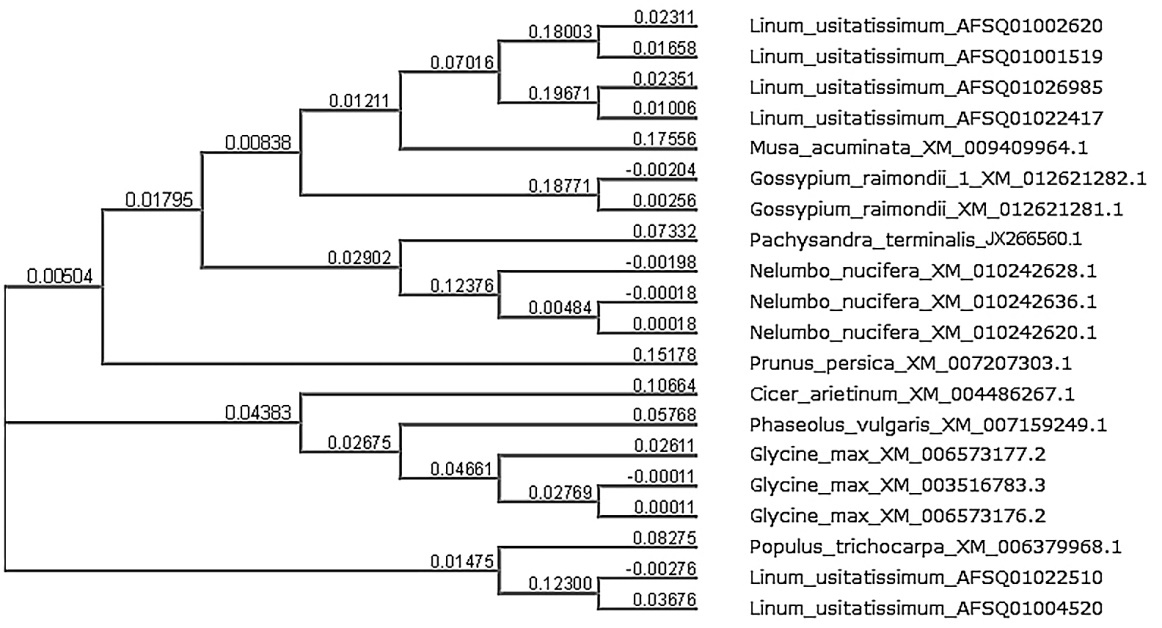 |

| chorismate synthase (*CS*) |
| --- |
| 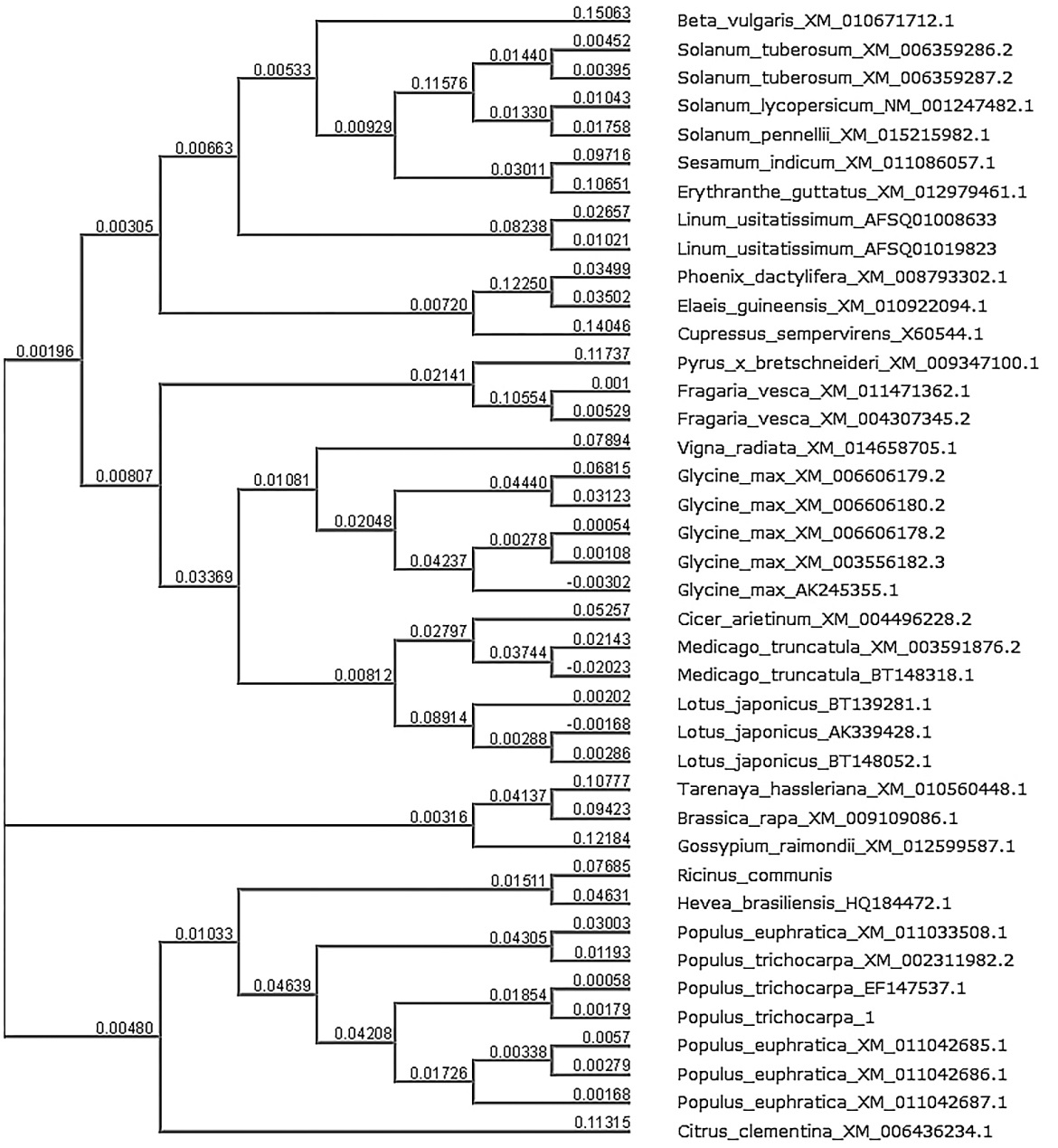 |

| chorismate mutase (*CM*) |
| --- |
| 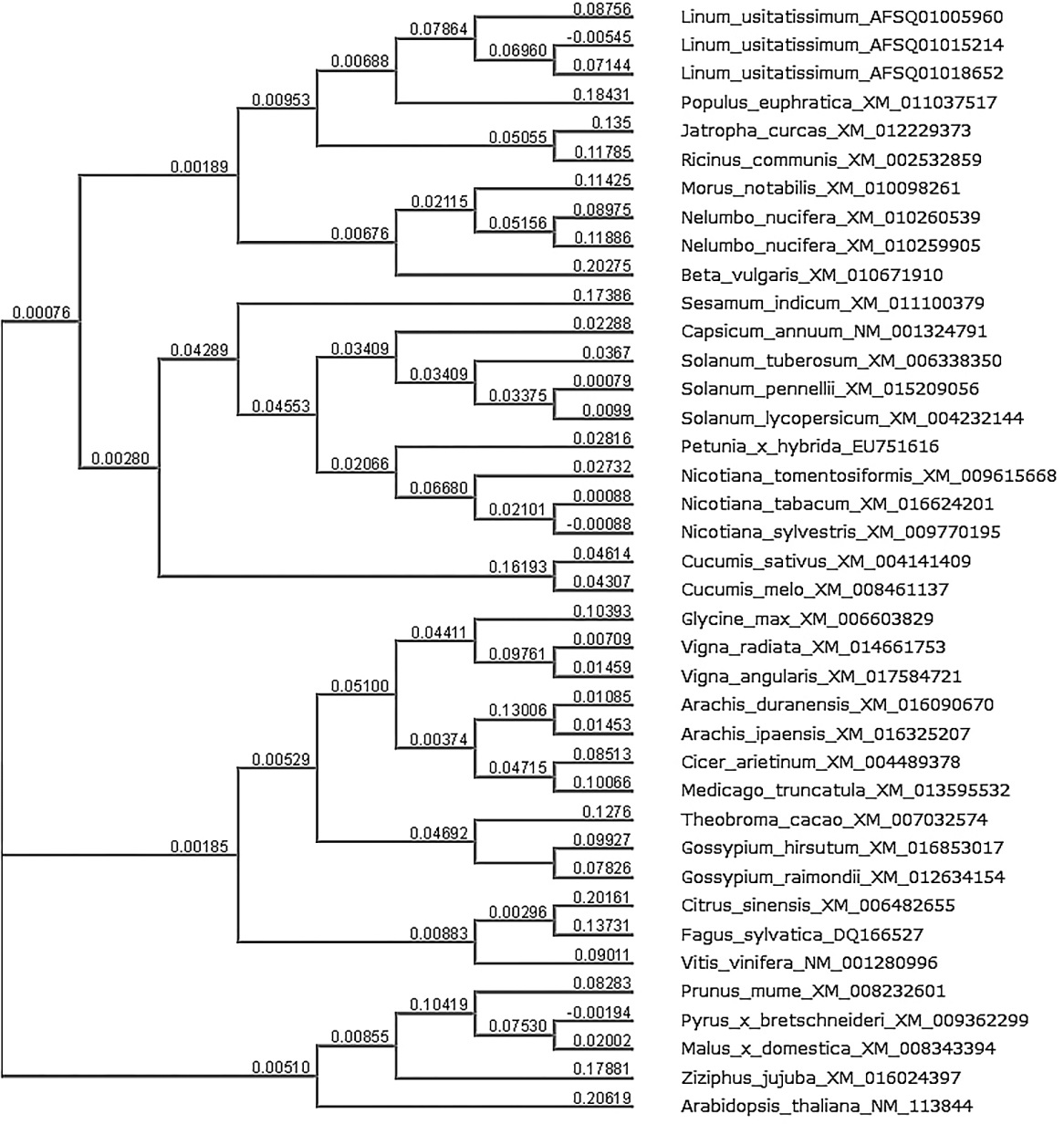 |

| phenylalanine ammonia lyase (*PAL*) |
| --- |
| 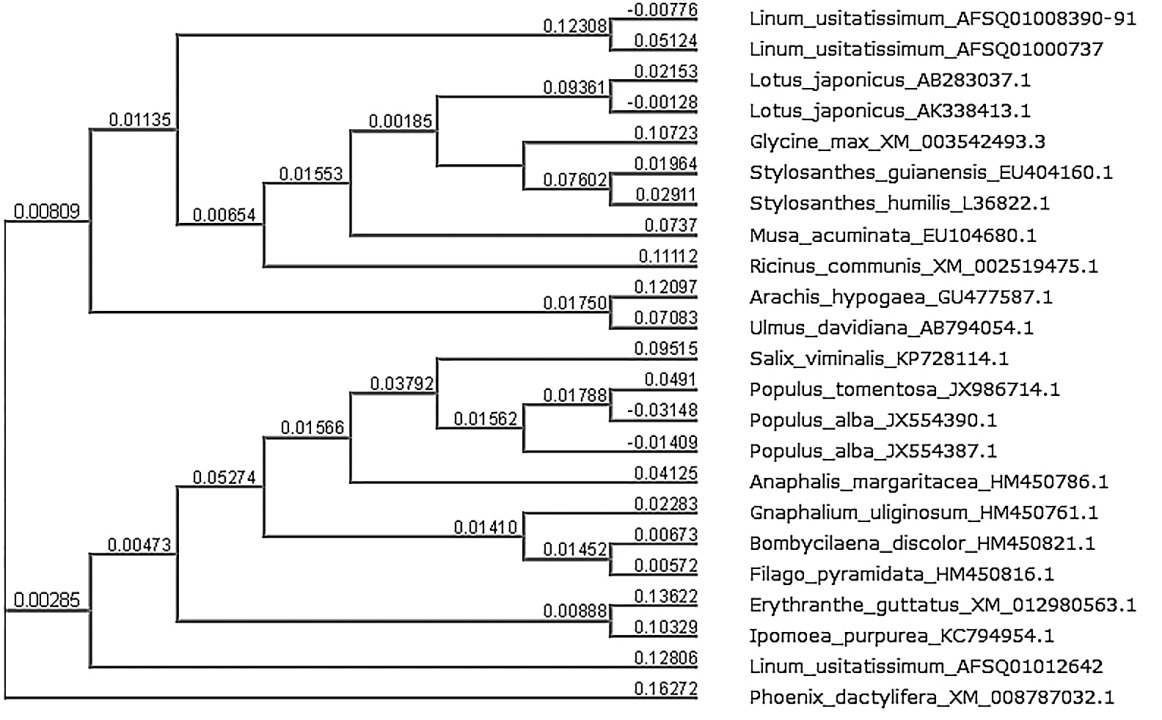 |

| trans-cinnamate 4-monooxygenase (*C4H*) |
| --- |
| 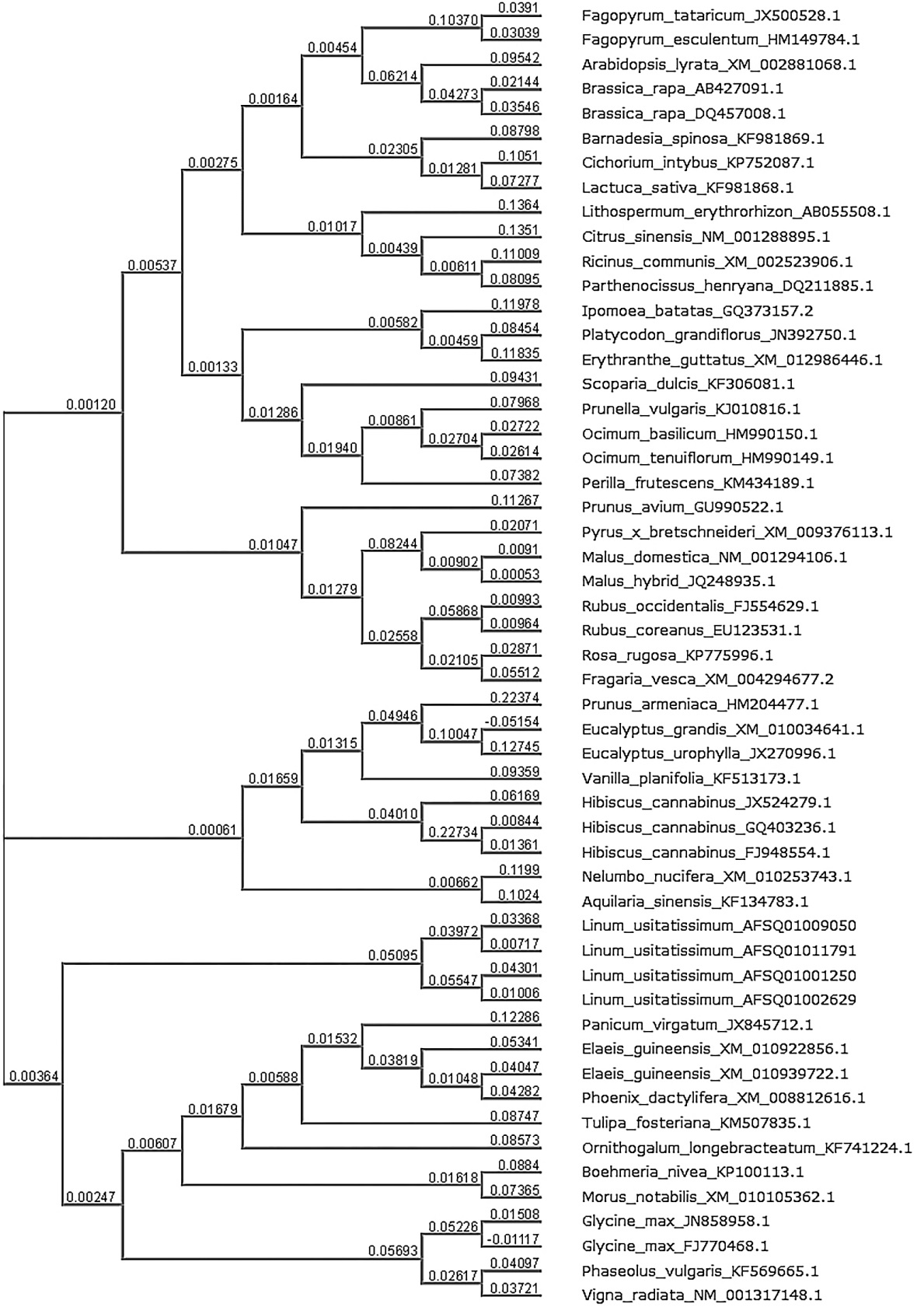 |

| 3-ketoacyl-CoA thiolase 2 (β-ketothiolase) |
| --- |
| 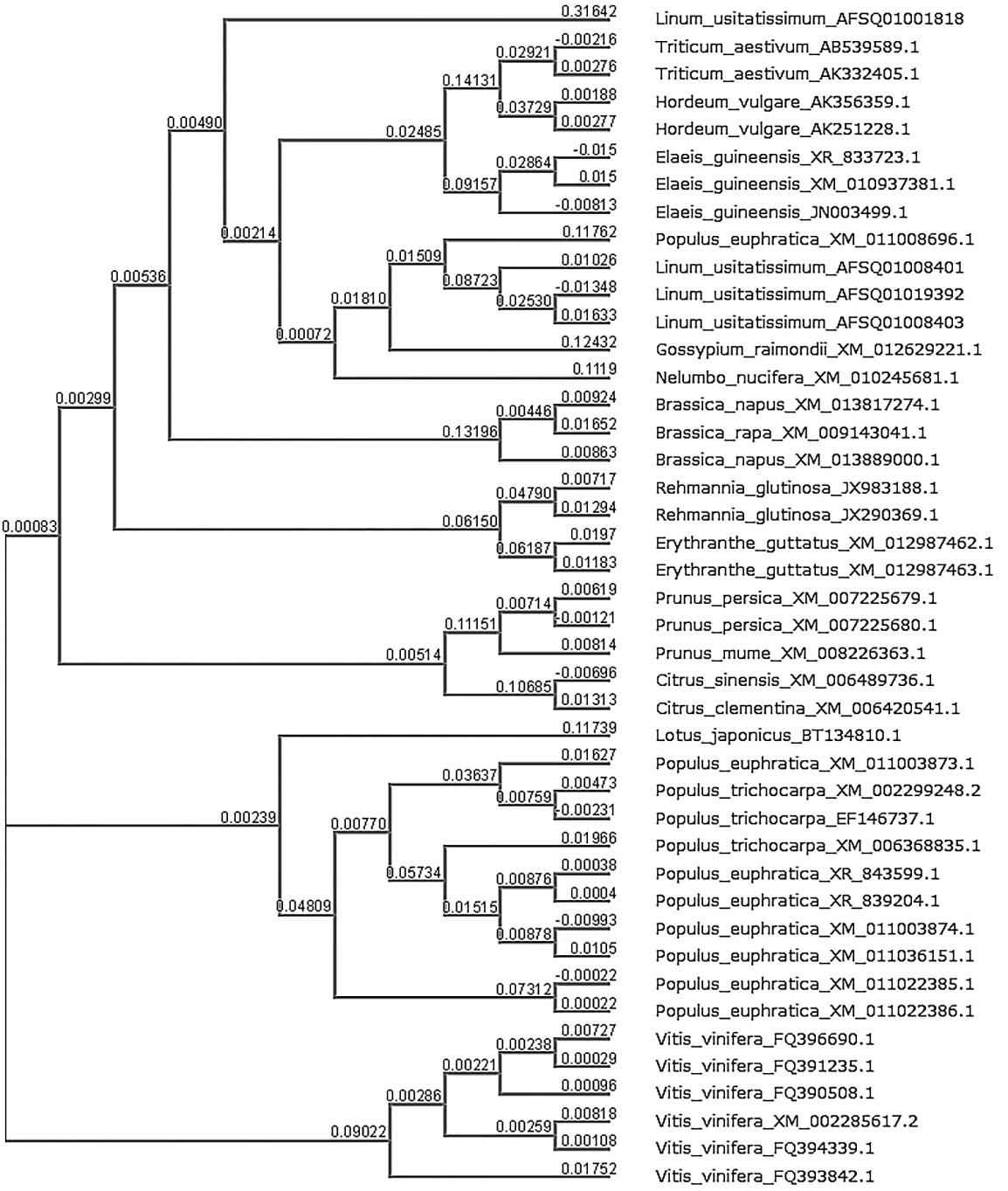 |

| benzoate/salicylate carboxymethyltransferase (*BSMT*) |
| --- |
| 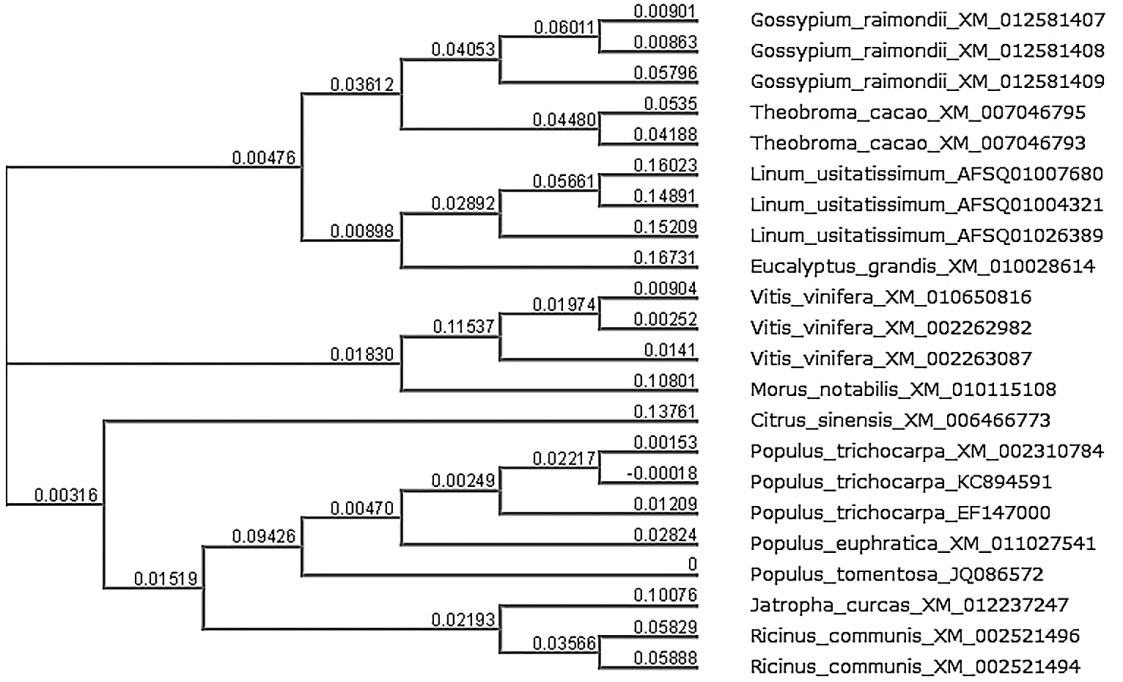 |

| benzyl alcohol O-benzoyltransferase (*BBT*) |
| --- |
| 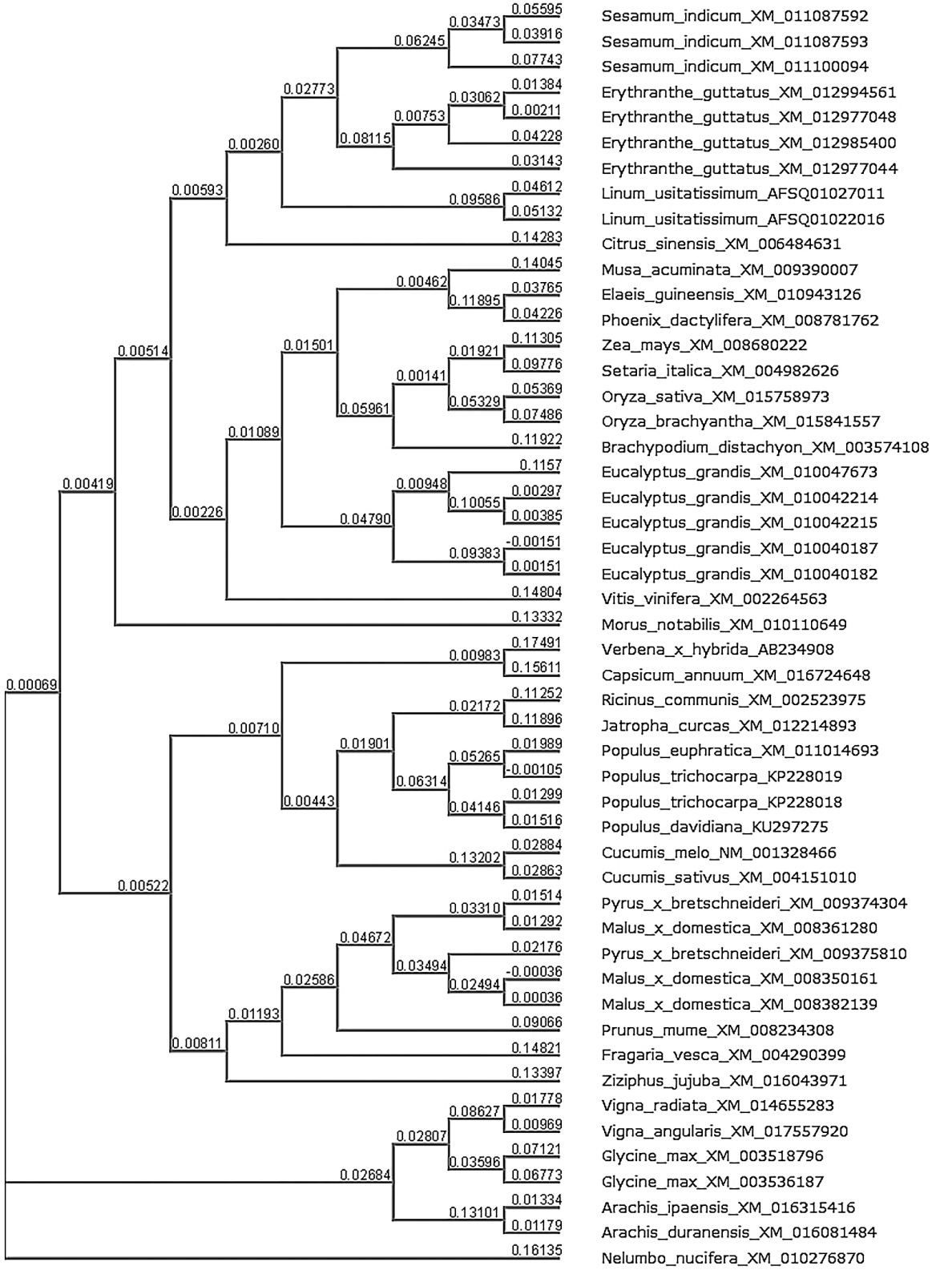 |

| 4-coumarate-CoA ligase (*4CL*) |
| --- |
| 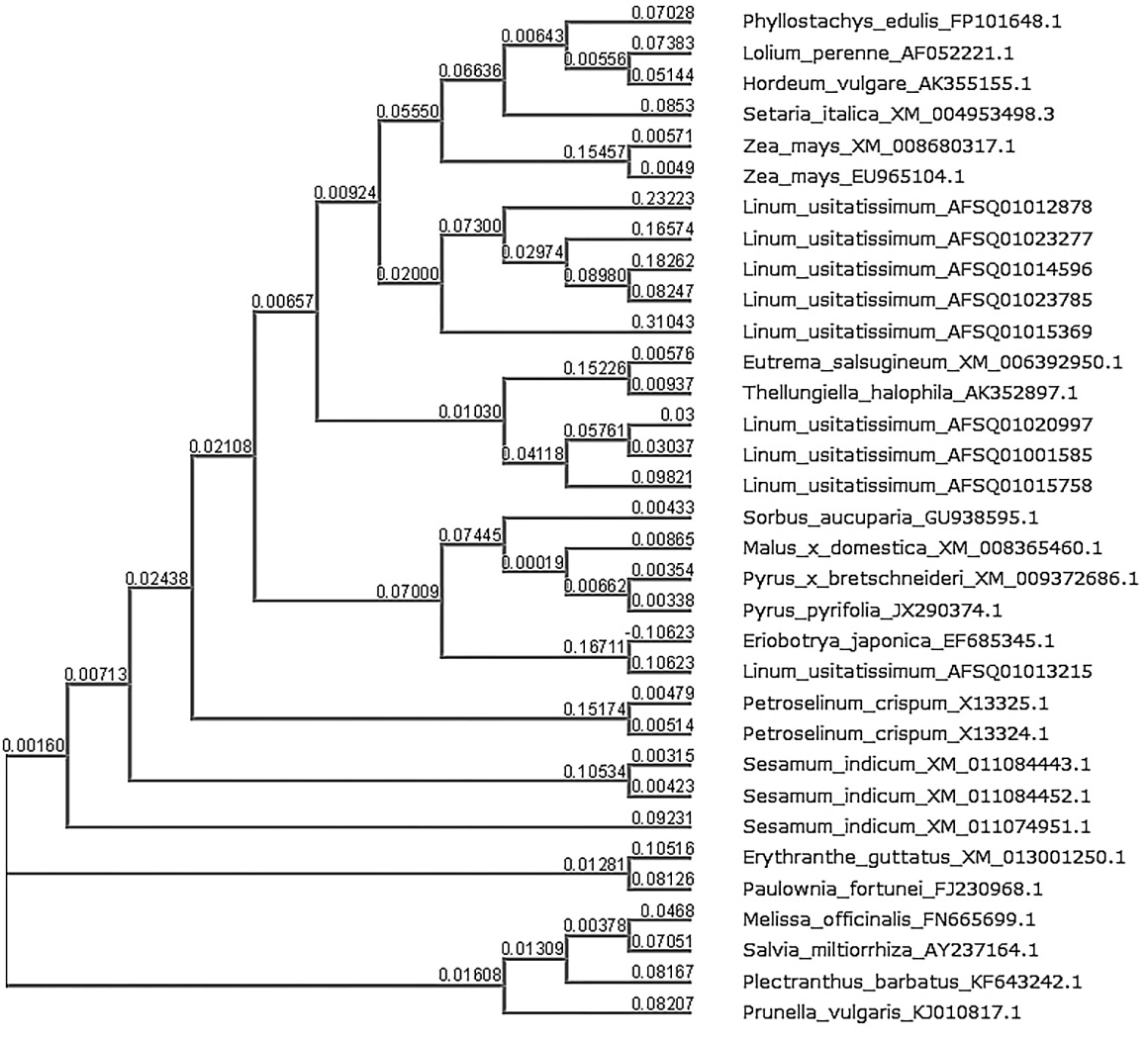 |

| hydroxycinnamoyl-CoA:quinate/shikimate hydroxycinnamoyl transferase (*HCT*) |
| --- |
| 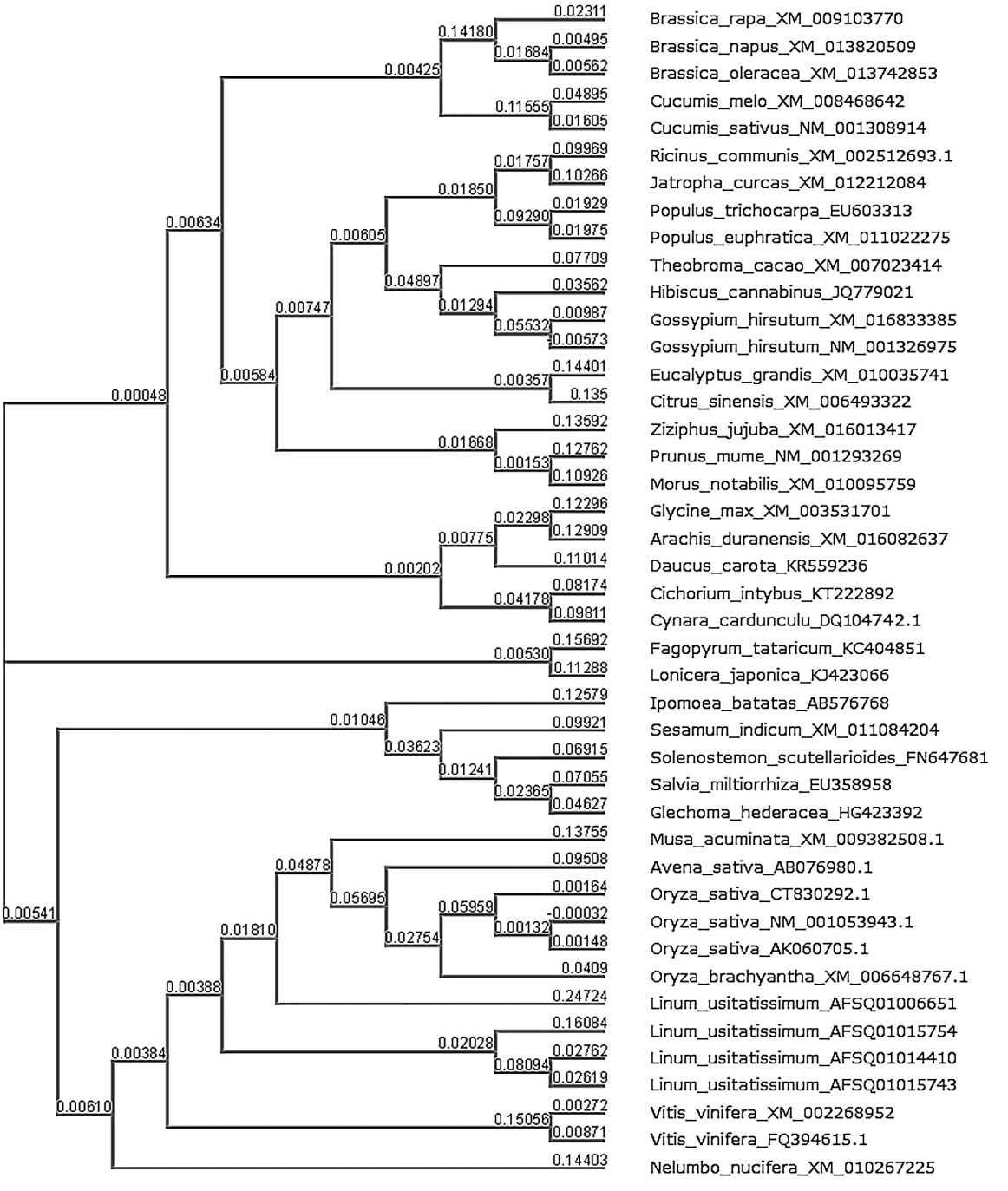 |

| caffeoyl shikimate esterase (*CSE*) |
| --- |
| 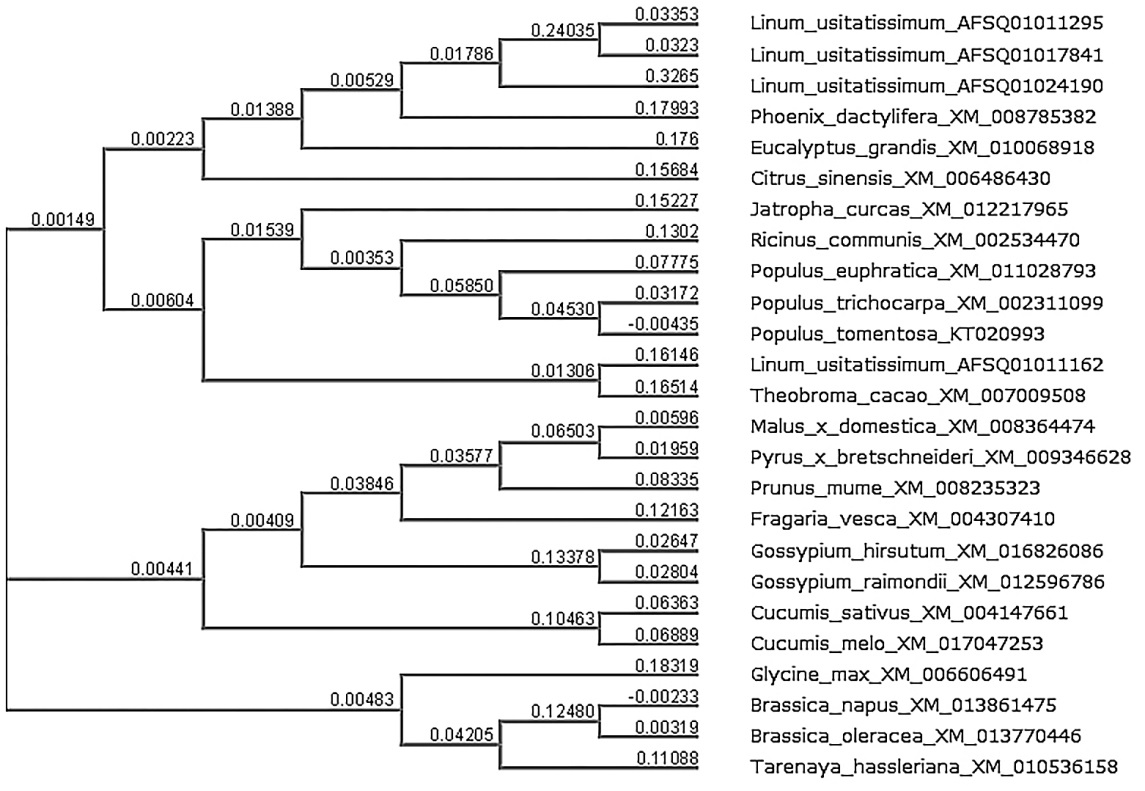 |

| caffeic acid 3-O-methyltransferase (*COMT*) |
| --- |
| 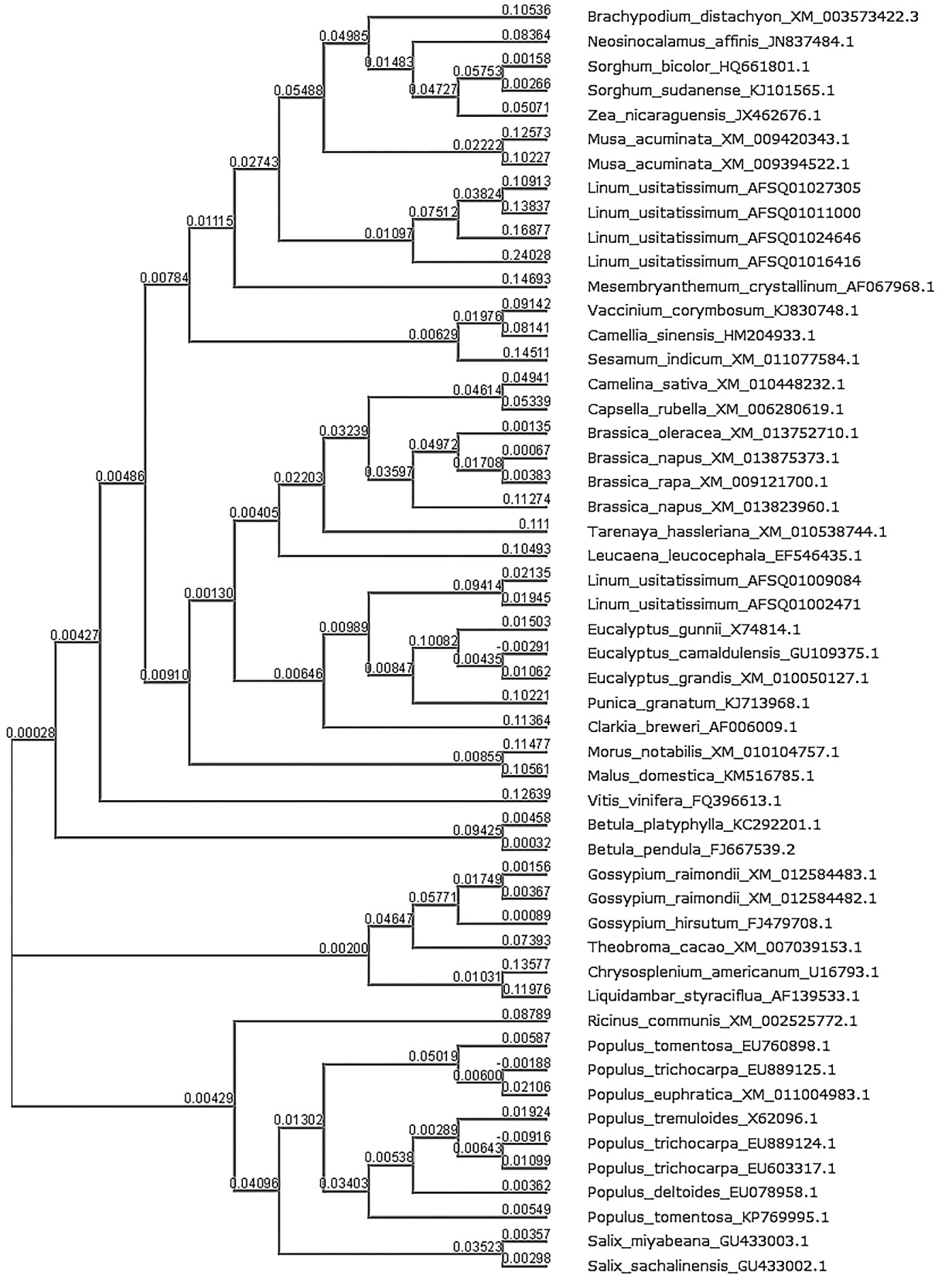 |

| caffeoyl-CoA O-methyltransferase (*CCoAOMT*) |
| --- |
| 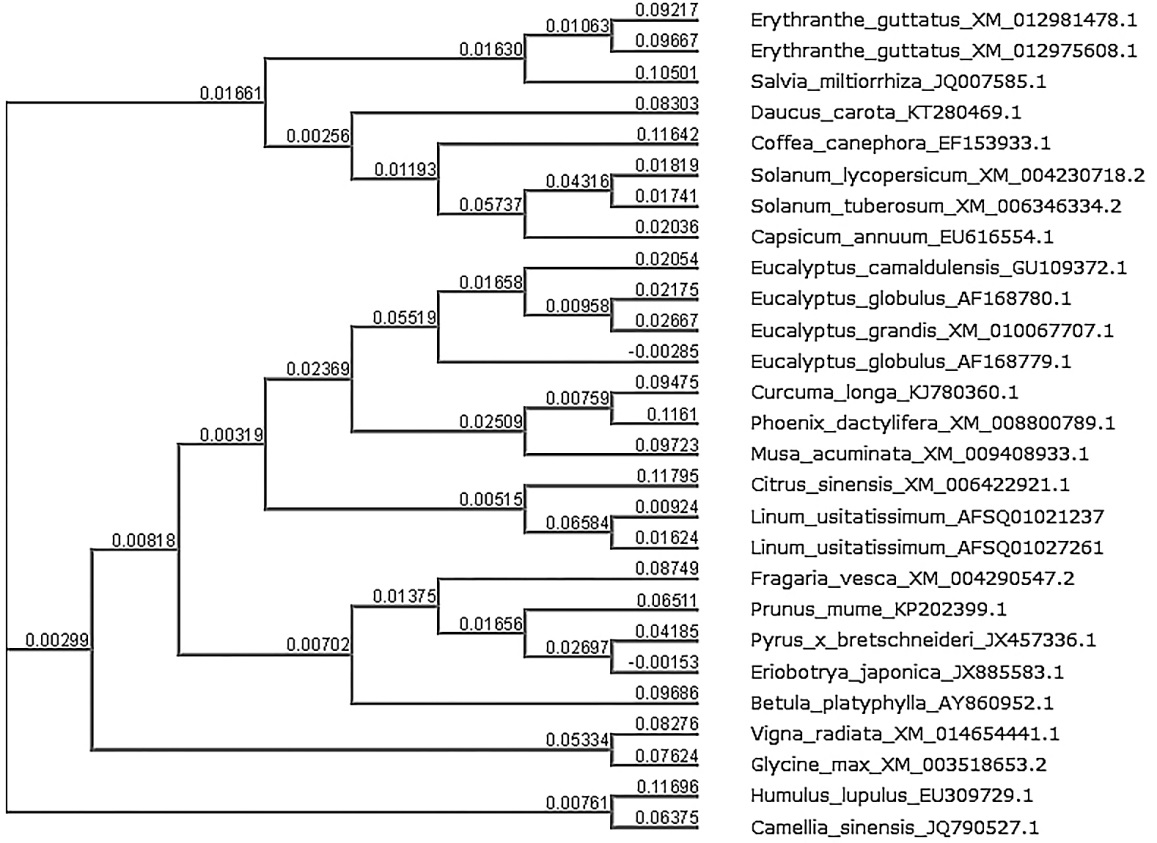 |

| cinnamoyl-CoA reductase (*CCR*) |
| --- |
| 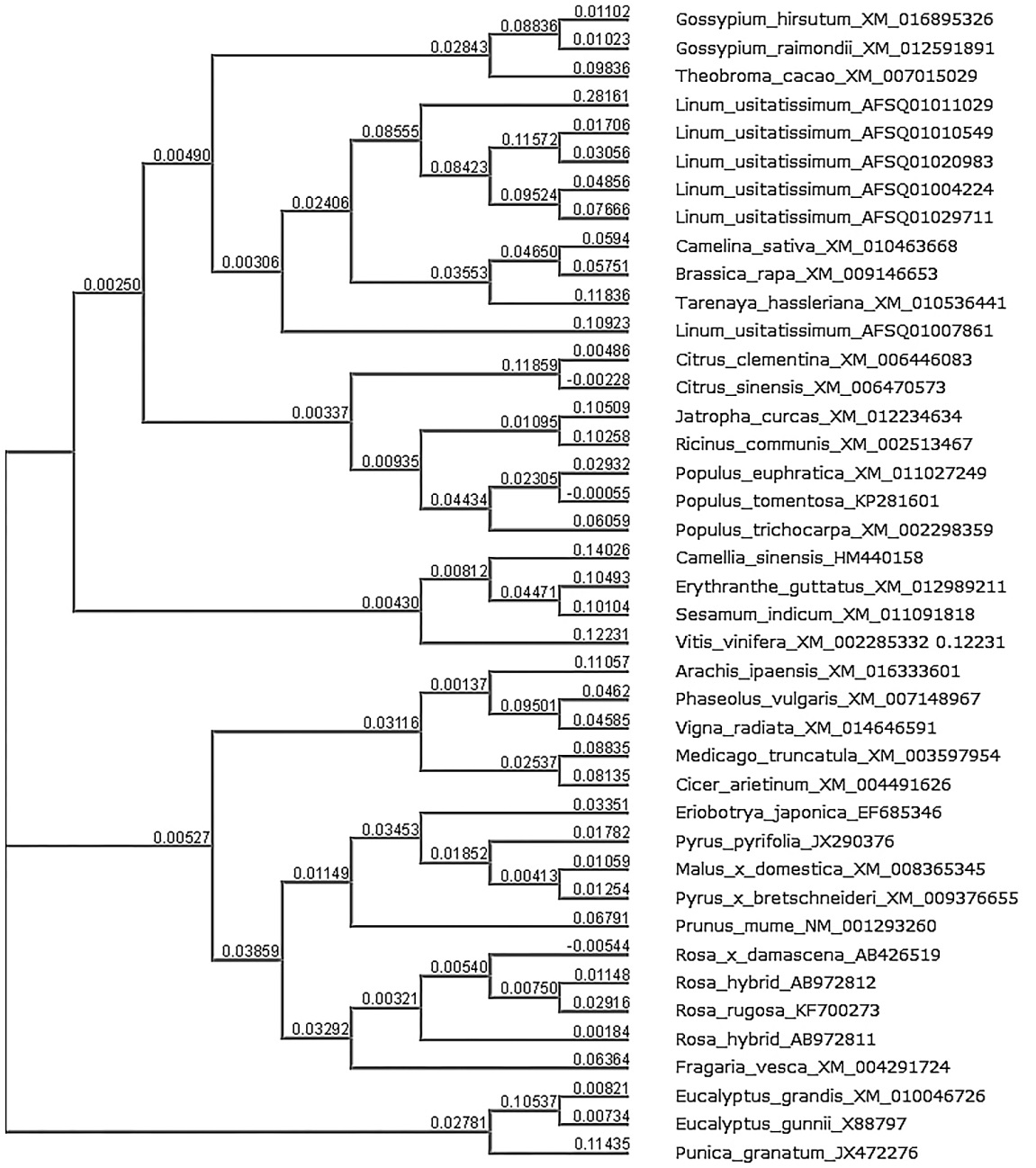 |

| cinnamyl alcohol dehydrogenase (*CAD*) |
| --- |
| 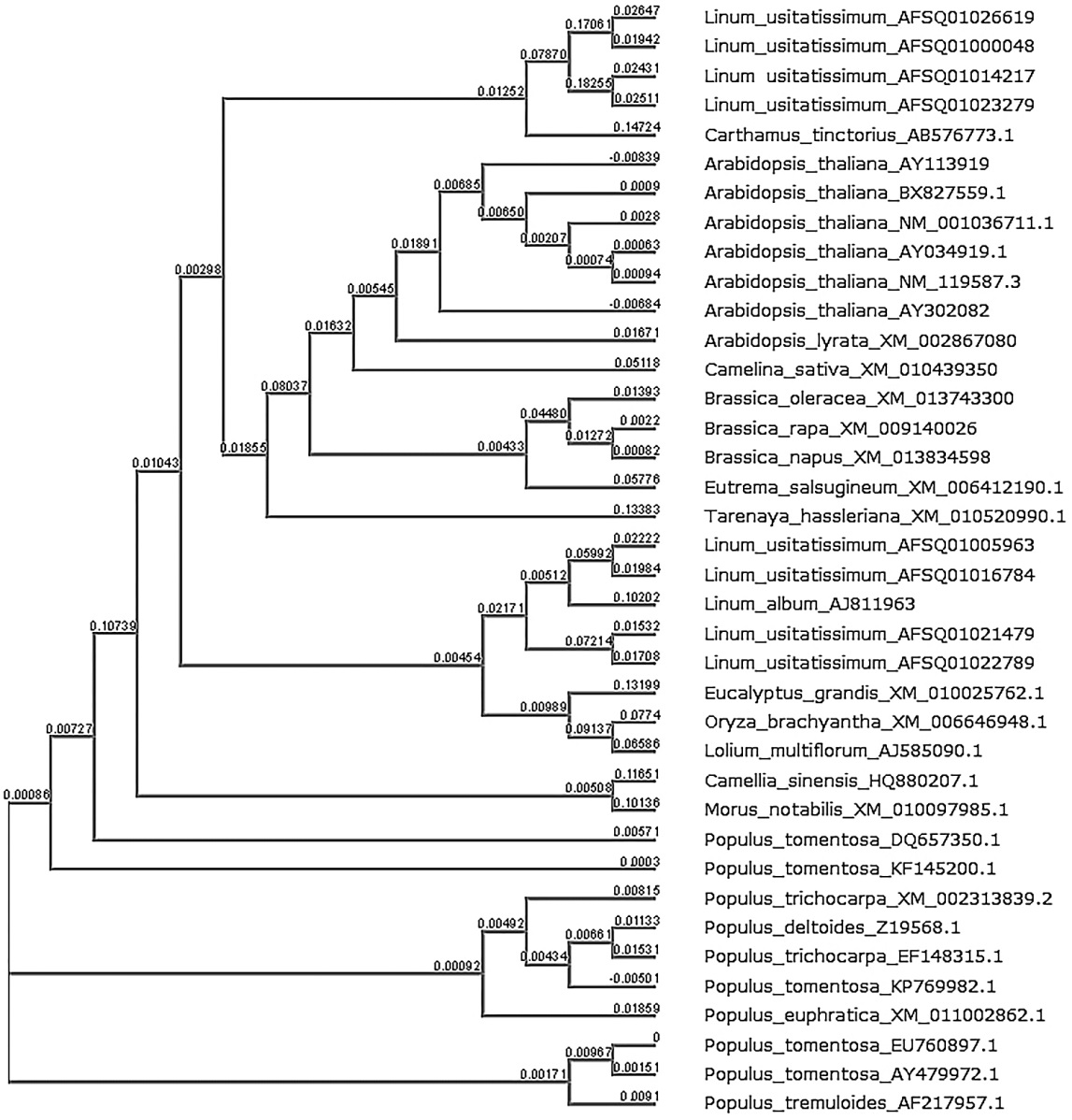 |

| chalcone synthase (*CHS*) |
| --- |
| 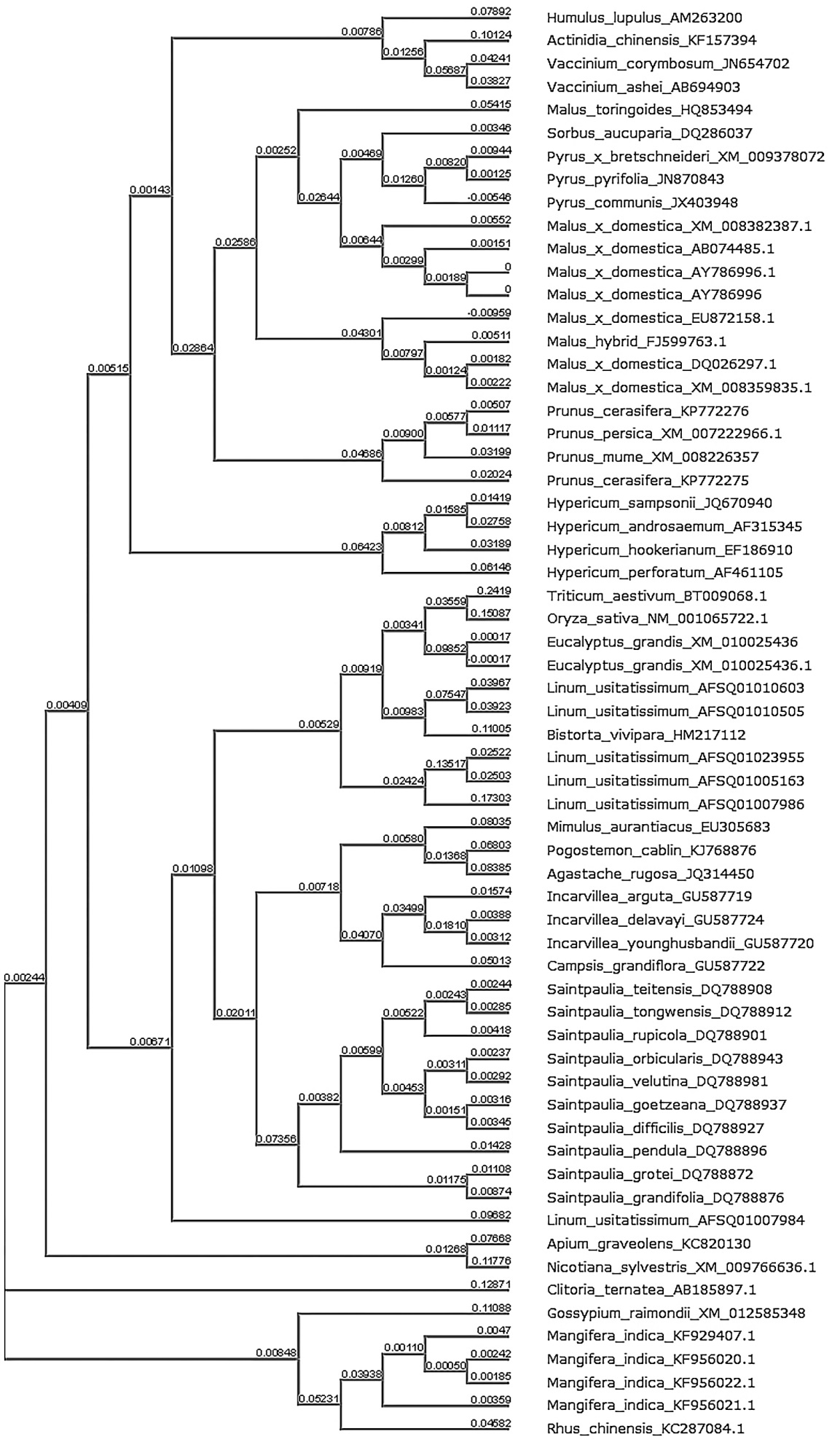 |

| flavanone 3-hydroxylase (*F3H*) |
| --- |
| 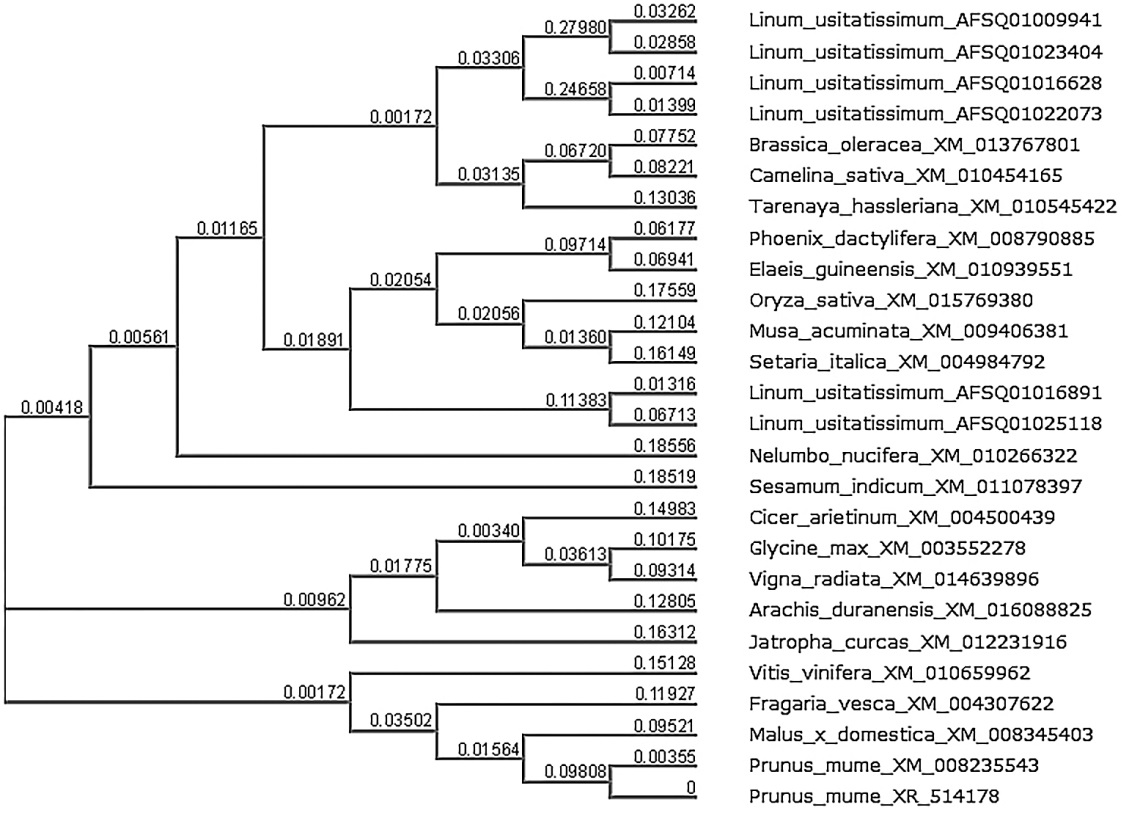 |

| isoflavone 2'-hydroxylase (*F2’H*) |
| --- |
| 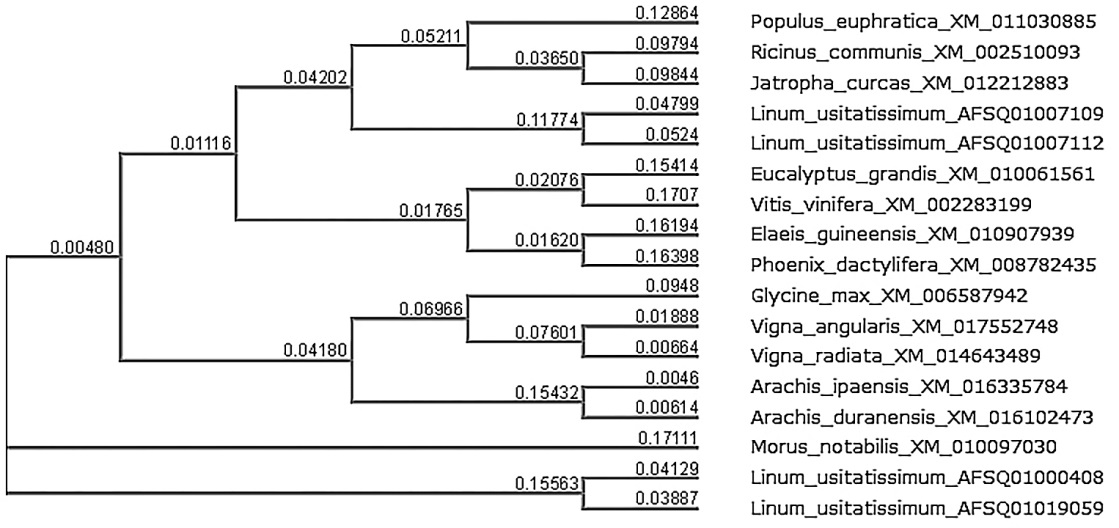 |

| flavonoid 3'-hydroxylase/monooxygenase (*F3'H*) |
| --- |
| 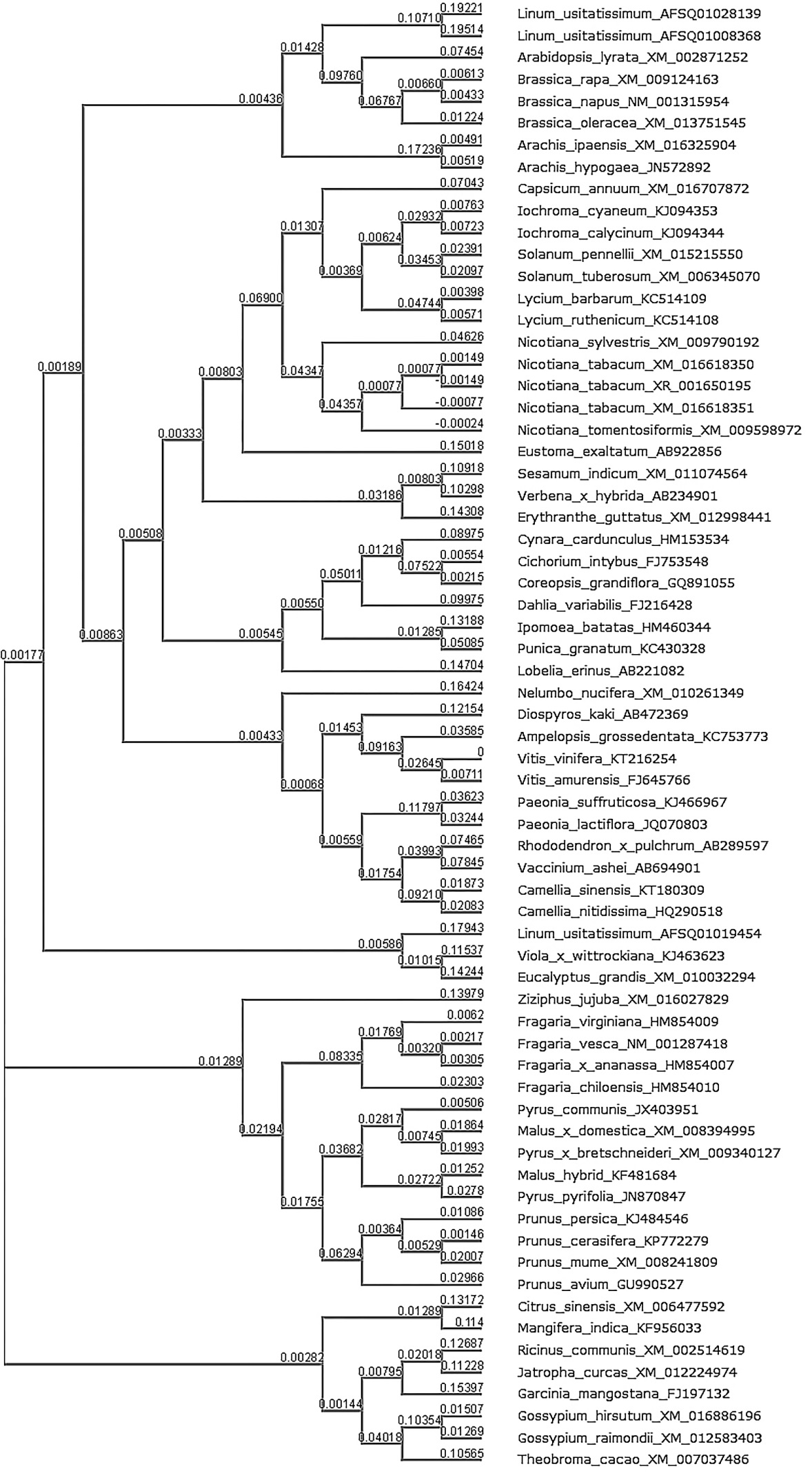 |

| flavonoid 3',5'-hydroxylase (*F3',5'H*) |
| --- |
| 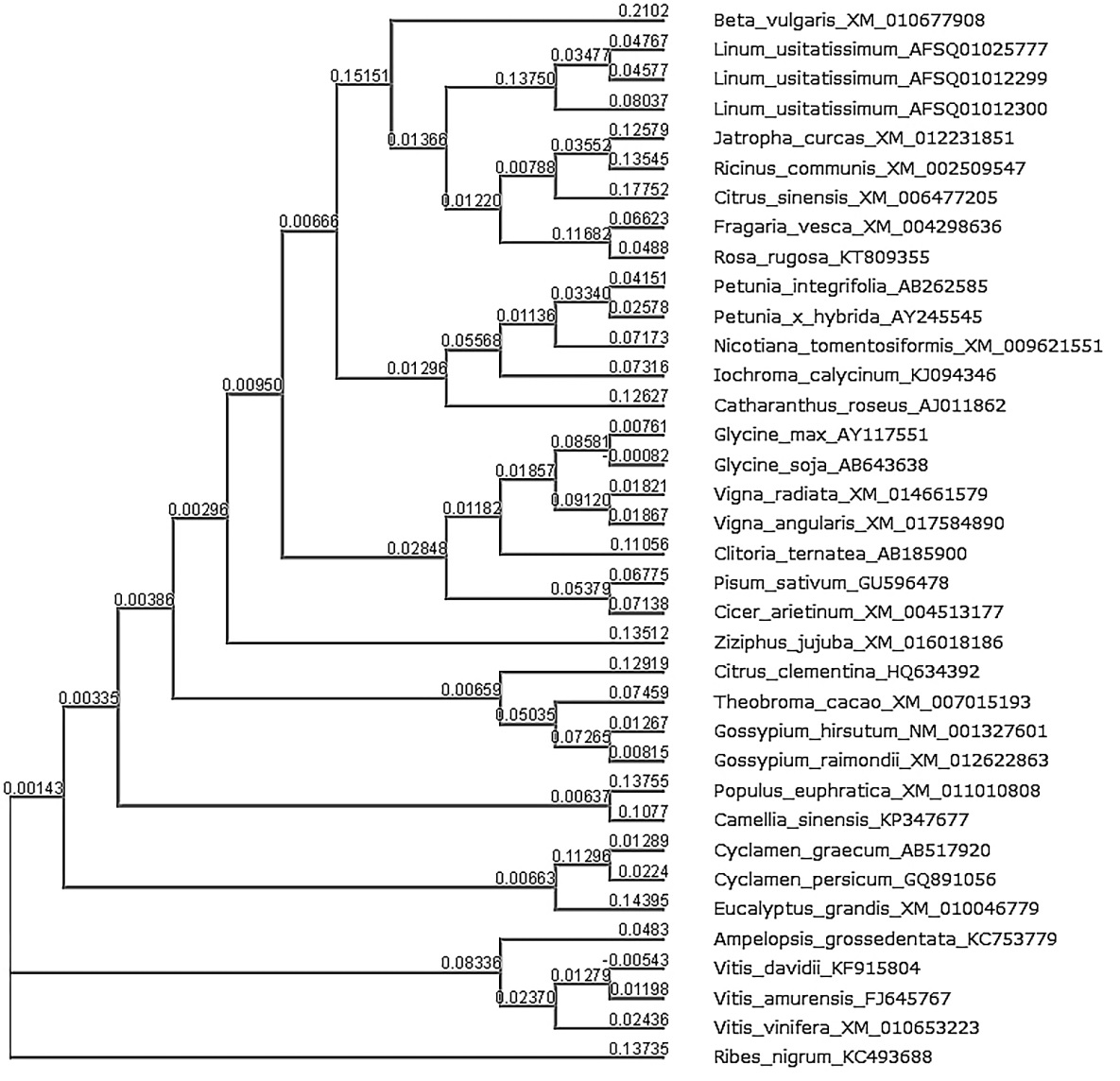 |

| leucoanthocyanidin dioxygenase (*LDOX*) |
| --- |
| 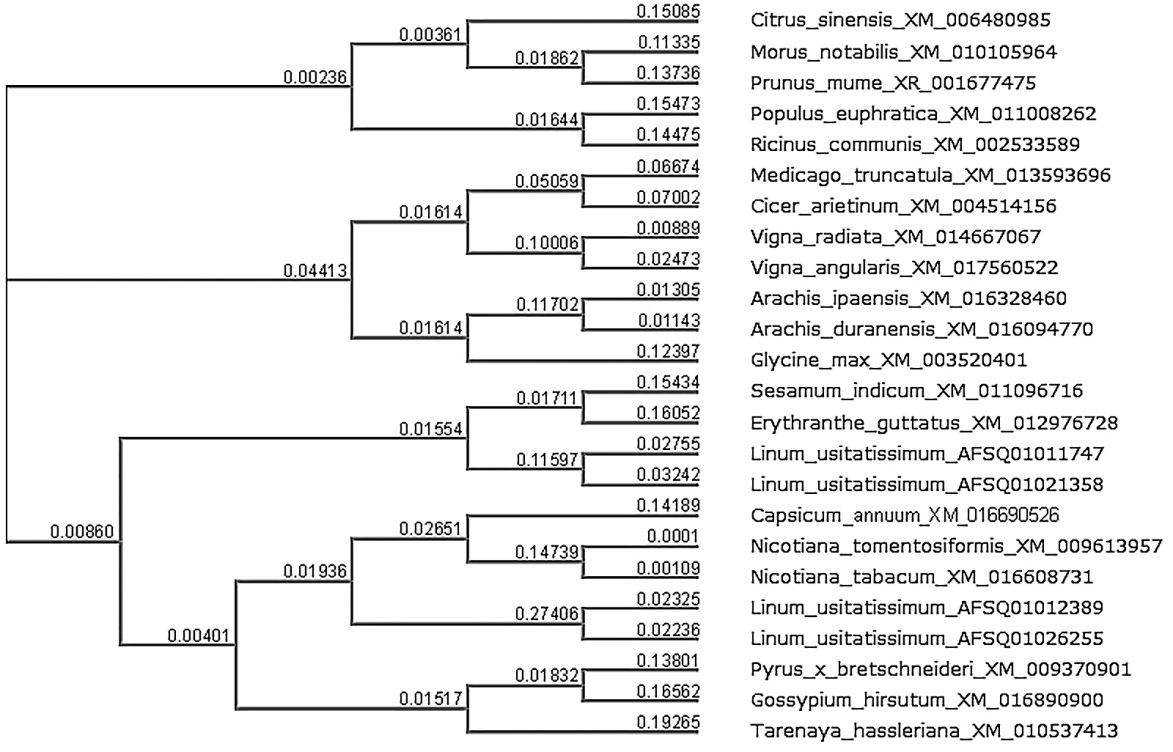 |

| anthocyanidin reductase (*ANR*) |
| --- |
| 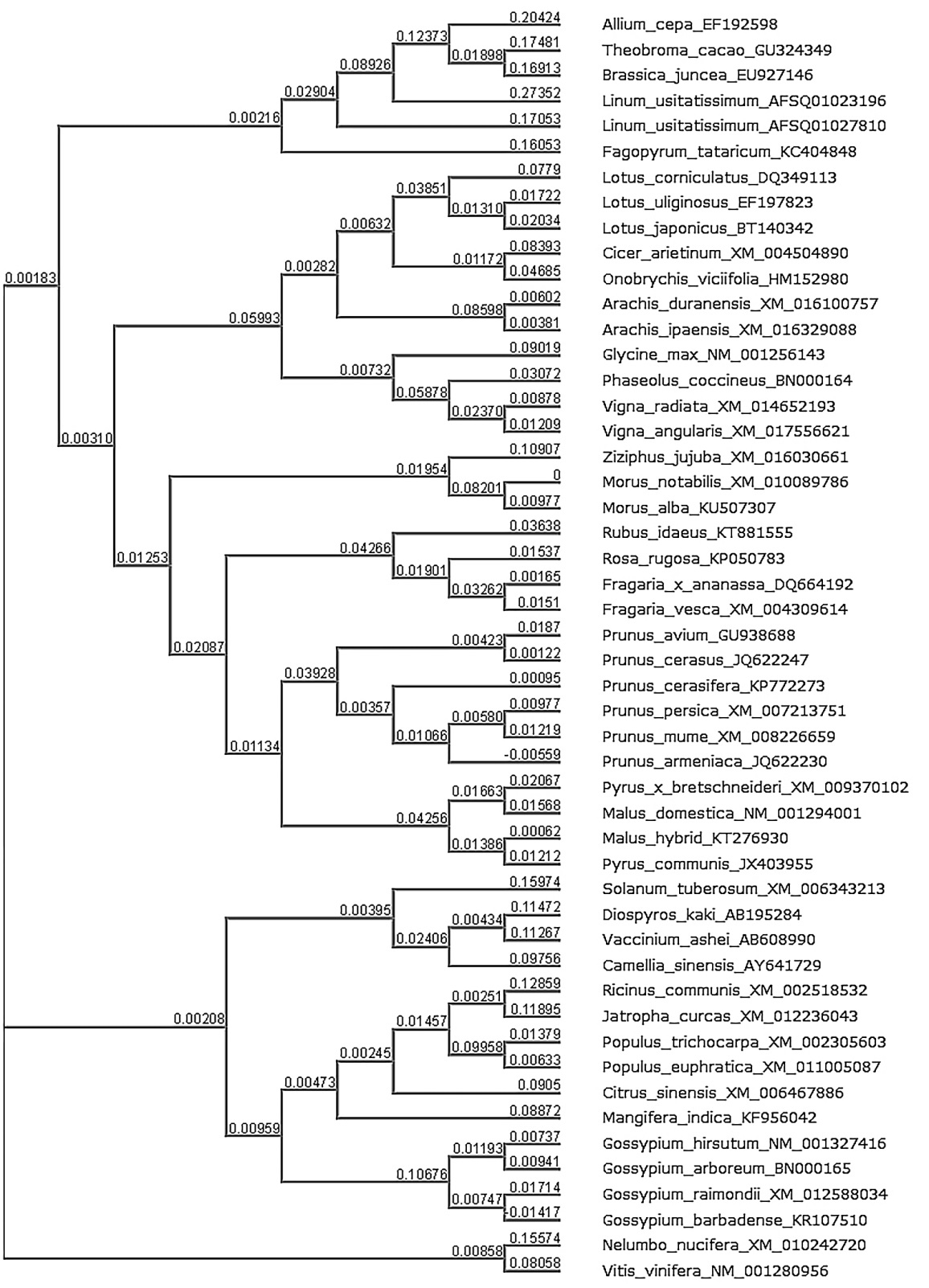 |

| UDP-glucosyltransferase (*UGT*) |
| --- |
| 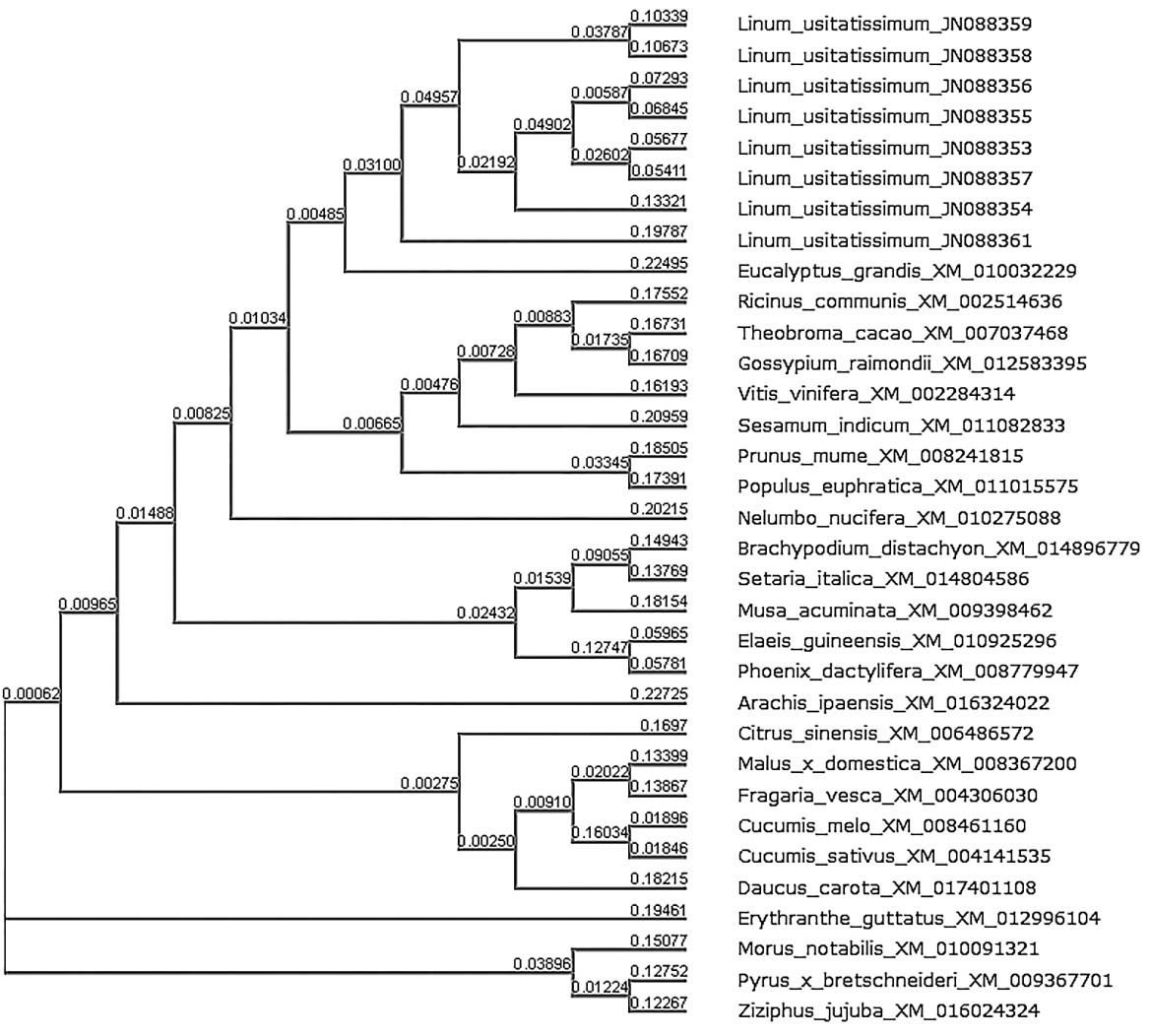 |
