# Supplementary material for: Methyl Salicylate Level Increase in Flax after Fusarium oxysporum Infection Is Associated with Phenylpropanoid Pathway Activation
Source: Front Plant Sci. 2017 Jan 20;7:1951. doi: 10.3389/fpls.2016.01951 (PMC5247452; doi:10.3389/fpls.2016.01951)
Supplement: Supplementary file 3 [file Table3.docx]

Supplementary Table S3. All recognized gene fragment sequences and accession numbers of genes analyzed in this study.

**SHIKIMATE DEHYDROGENASE**

**AFSQ01002620**

ATGGCATTCTGGACTAATCTTCTGGTGTGCACGCCTCTGGAACGTGAAACCACAGGAGGA

ATGTTGTGCGCCATGGAGAAAGCCAAGGCAGAAGGCGCAGACGTTGTGGAGCTTTCCATG

GGTTCCTTGTCATTCTCCCACATTTCTCAAGTTGAGACGCTCCTTGCACAAAGAACCCTG

CCTACTATCGTCTCTTGCAGCAGGCTGAAGAAATCATCAAGCGCCGAGGAAAGGGGGCAG

AGTCTCGGCACTTGTTTGGAAGTGTTGAGACTGGCTCTAGAATTAGATGTGGAGTTTCTA

GAAATTGATTACGAGATGGCTTCTGATGCCAGGATGGTAGAGGAGGTATTAGAGAGCCGC

AGGTCCAGCATCAAGCTTATTGTGTCAAGCCATGTAAATGGCCTGAAACCTTCAGCAGAG

GAACTTGGCAATCTAATTGCATCCATGCAGTCCACTGGAGCAGATGTTATTAAGCTTGTG

ATCGACGTGGAGTCCATTACTGATCTGGCTCCTGTTTTTAGAATGCTTACACATTCCCAG

GTACCATTGATTGCTCTAGCAGTGGGTAGTAGAGGCCTTATAAGCCAGCTTTTGGGGCCG

AAATTTGGCGGGTGTTTCGTGTATGGTTCATTGGATGAGGACAAAGCAGTAGCTGGTGGC

ATCCCAACTCTGTCCAGTCTCAAAAAAGTTTACAAGCTTGAACATATAAACGTAGACACA

AAAGTCTTTGGCCTAATTTCAAACCCCGTTGGGCATAGCAAGGGTCCTATTCTCCACAAC

CCTGCTTTTCGCCACACGGGATATAACGGGATTTACGTTCCTATGCAAGTGGATGATATC

AAAGAGTTCTTCACAATTTACACCAGCACTGATTTTGCAGGTTTCAGTGTTGGAATCCCA

CACAAGGAAGCTGCAGTAGGATGCTGTGACGAGGTCGATCCACTGGCTAAGTTAATAGGA

GCTGTAAATACAATAGTAAGAAGGGCCAAAGACGGGAAGCTGGTTGGCTACAACACAGAC

TGCGAGGCGTCAATCTCAGCGATAGAGGATGCACTCAGAGTCAGAGGTGAAAAGCCACCC

TTCCCTAAAACACATCTCGTTCCATCGTCAACTATGAATATTGATATACATGGTGCAAAA

AACGCAGAAAAAGGAAGGGTTTTTCGAAACAGAGGTTACTTGGAGGATAATAGCAGTAGC

AGTTTTGGTGGTCCTCTAGCTGGGAAGACTTTTGTGGTAGTGGGTGCAGGGGGAGCTGGG

AGAGCACTCGCATTCGGTGCCAAAAGCAGAGGTTGTAAAAGGGTTGTCATCTTCAACCGC

AATTTTGAGAGAGCCAAGGCTCTGGCTGCTGCAGTTTCAGGAGAAGCACTCCCTTACGAG

ACCCTGGACACATTCCAGCCAGAGAAAGGCATGATTCTTGCAAATGCTTCTGCCGTTGGA

ATGCAGCCAAACACAGACACCACACCTGTTTCCAAGGAGGCATTAAAGGTGTACGATCTG

GTTTTCGATGCGGTTTATACCCCGAGAAACACGAGACTGTTGCAGGAGGCTGGAGAAGCA

GGGGCCACCGTGGTAAGTGGAGTTGAGATGTTCATCAGACAGGCTCTTGGCCAGTTCACC

CTCTTCACTGGCGGCTTAGCTCCGGAGGCATTCATTCGTAAGCTTGTTCTGGAGCAATTC

TGA

**AFSQ01001519**

ATGGCATTCTGGACTAATCTTCTGGTGTGCACGCCTCTGGAACGTGAAACCACAGGAGGA

ATGTTGTGCGCCATGGAGAAAGCCAAGGCAGAAGGCGCAGACGTTGTGGAGCTTCACATG

AGTTCCTTGTCATTCTCCCACATTTCTCAAGTTGAGACGCTCCTTGCACAAAGAACCCTG

CCTACTATCGTCTCTTACAGCAGGCTGAAAAAATCGTCAAGCGCCGAGGAAAGGAGGCAA

AGTCTCAGCACTTGTTTGGAAGTGTTGAGACTGGCTCTAGAGTTAGATGTGGAGTTTGTG

GAAATTGATTACGAGATGGCTTCTGATGCCAGCATGGTAGAGGAGGTACTAGAGAGCCGC

AGGTCCAACATCAAGCTTATTGTGTCAAGCCATGTAAATGGCCTGAAACCTTCAGCAGAG

GAACTTGGCAATCTAATTGCATCCATGCAGTCCACTGGAGCAGATGTTATTAAGCTTGTG

ATCGACGTGGAGTCCATTACTGATCTGGCTCCTGTTTTCAGAATGCTTACGCACTCCCAG

GTGCCATTGATTGCTCTAGCAGTGGGTAGTAGAGGCCTTATATGCCAGCTTTTGGGGCCG

AAATTTGGCGGGTGTTTTGTGTATGACACCAAAATCTTTGGCCTAATTTCGAACCCCGTT

GGGCATAGCAAGGGTCCTATTCTCCACAACCCTGCTTTTCGCCACACAGGTTACAACGGG

ATTTACGTTCCTATGCAAGTGGATGATATCAAAGAATTCTTCACAATTTACAACAACACT

GATTTTGCAGGTTTCAGTGTTGGAATCCCACACAAGGAAGCTGCAGTAGGATGCTGTGAC

GAGGTCGATCCACTGGCTAAGTTAATAGGAGCTGTAAATACAATAGTAAGAAGAGCCAAA

GACGGGAAGCTGGTTGGTTACAACACAGACTGCGAGGCGTCAATCTCAGCGATAGAAGAT

GCACTCAGAATCAGAGAAAAAGGAAGGGTATTCCGAAACAGAGGTTACTTGGAGAATAAT

AGCAGTAGTAGTTGTGGTGGTCCTCTAGCTGGGAAGACTTTTGTGGTAGTGGGTGCAGGG

GGAGCTGGGATAGCACTCGCATTCGGTGCCAAAAGTAGAGGTTGTAAAAGGGTTGTCATC

TTCAACCGCAATTTTGAGAGAGCCAAGGCTCTGGCTGCTGCGCTTTTAGGAGAAGCACTC

CCTTTCGAAAAACTCGACACGTTCCAGCCGGAGAAAGGCATGATTCTTGCAAATGCTTCT

GCCATCGGAATGCAGCCAAACACAGACACCACACCTGTTTCCAAGGTGGCATTAAAGGTG

TACGATCTGGTTTTCGACGCTGTTTACACCCCGAGAAACACGAGACTGTTGCAGGAGGCT

GGAGAAGCAGGGGCCACCGTGGTAAGTGGAGTTGAGATGTTCATCAGACAGGCTCTTGGC

CAGTTCACCCTCTTCACTGGCGGCTTAGCCCCGGAGGCTTTCATGCGTAAGCTTGTTCTG

GAGCAATTCTGA

**AFSQ01022510**

ATGGACTCCGGCAATGTCATGCTGACCTCAAGCTCTTCTGCAATGGGCGGCGGAACTGGA

CTTCCGCTCAACAATCCGACCTTAATCTGTGTTCCAATCATGGCGGATTCCGTAGATGAC

ATGCTCGTTAAAGCATCCCAGGCCAAGACCGCCGGTGCTGATCTCGTAGAAATTCGATTG

GATAGCTTGACTAACTTCCTCCACCCTCGCCAGGATCTCACCACGCTCATTAATGGCTCT

CCCTTGCCCACTCTCTGTACCTACAGGCCGAGGTGGGAAGGAGGCCAGTACGGGGGTGAT

GAAAGCGAGCGTTTGGATGTTCTTAAGTTAGCCATGGAATTGGGAGCTGATTACATTGAC

GTTGAGCTTCAGGTTGCCGGTGATTTCATTGAATCCTTACATGGAAACAGACCTGCAAAG

TGCAAAGTCATTGTTTCTTCTCATAACTATGAAAATACGCCATCCGTTGAGGACCTGGGT

AACTTGGTGGCAAGAATACAATCAACGGGAGCTGACATAGTGAAGATTGCTACAACAGCC

TTGGATATTACTGACAACGCGAGAATGTTTCGAATAACAGTTCATTCTCAAGTTCCAATA

ATAGGACTTGTTATGGGAGAGAGGGGTTTGATGTCAAGGGTGCTTTGTGGCAAATTTGGG

GGATATCTCACCTTTGGAACTTTGGAGTCTGGAGTGGTCTCGGCTCCTGGGCAGCCGTTG

ATCAAAGACCTGCTGGATCTATACAATTTCAGACAAATCCAAGCTGATACCAAGGTTTTC

GGCATAATAGGGAAGCCAGTTGGGCACAGCAAATCACCTGTTCTGTTCAATGAAGCATAC

AGATCACTTGGTTTTAATGGGGTATATGTGCACTTATTAGTTGATGACCTGGCAAGTTTT

CTCAAGACTTACTCATCTCCGGATTTTGCAGGATTCAGTTGTACGATTCCTCACAAAGAG

GTTGCTGTGAAGTGTTGCGATGAAGTTGATCCAGTTGCAAAGTCGATAGGAGCTGTTAAT

TGCATTGTAAGGAGAGAAAGTGATGGGAAGTTATTTGGTTTCAATACAGACTATGTTGGT

GCAATTTCTGCAATTGACGATGGACTTCAAGCTTCACGAAATGGTAGCAGTCAATCTGGT

TCACCCTTAGCTGGTAAGGTGTTTGTGGTCATTGGCGCTGGAGGTGCTGGTAAAGCACTA

GCTTATGGTGCAAAAGAAAAGGGAGCAAAGGTCATCATTGCCAATCGTACCTACGGTCGA

GCGAAAGAGCTTGCTGACATAATTGGAGGAGAAGCCATCTCTCTAGCTGATCTAGAGAAT

TTCCATCCAGAAAGTGACATGATTCTTGCAAACACAACATCAATTGGAATGGAGCCAAAA

ATCGACGAAACACCCATCCCCAAGCATGCATTGAGACATTACGCCCTGGTATTCGATGCC

GTTTACACCCCGAAAATTACGAGGATGCTGAGAGAAGCTGAGGAGTGTGGAGCCACGATC

GTGTCGGGGCTGGAGATGTTCATTGGGCAAGCCTACCAGCAGTTTGAGAGGTTCACTGGA

TTACCAGCTCCCAAGGAACTTTTCCGCAAGACCATGTCCAAGTAA

**AFSQ01004520**

ATGGACTCCGGCAATGTCATGCTGACCTCAAGCTCTTCTGCAATGGGCGGCGGAACTGGA

CTTCCCCTCAACAATCCGACCCTAATCTGTGTTCCAATCATGGCGGATTCCATAGATGAC

ATGCTCGTTAAAGCTTCCCAAGCCAAGACCGCCGGTGCTGATCTCGTGGAAATTCGATTG

GATAGCTTGACTAACTTCGTTCACCCTCGCCAGGATCTCACCACGCTCATTAATGGCTCT

CCCTTGCCCACTCTCTGTACCTACAGGCCGAGGTGGGAAGGAGGCCAGTACGACGGTGAT

GAAAGCGAGCGTTTGGATGTTCTTAAGTTAGCTATGGAATTGGGAGCTGATTACATTGAC

GTTGAGCTTCAGGTTGCTGGTGATTTCATTGAATCCTTACATGGAAACAGACCTCCAAAG

TGCAAAGTCATTGTTTCTTCTCATAACTACGAAAATACGCCATCCGTTGAGGACCTTGGT

AACTTGGTGGCAAGAATACAATCGGCGGGAGCTGACATTGTGAAGATTGCTACAACAGCC

TTGGATATTACTGACAACGCAAGGATGTTTCGAATAACAGTTCATTCTCAAGTTCCAATA

ATAGGACTTGTTATGGGAGAGAGGGGTTTGATGTCAAGGGTGCTTTGTGGCAAATTTGGG

GGATATCTCACCTTTGGAACTTTGGAGTCTGGAGTGGTCTCGGCTCCTGGGCAGCCGTTG

ATCAAAGACCTGCTGGATCTATACAATTTCAGACAAATCCAAGCTGATACCAAGGTTTTC

GGCATAATAGGGAAGCCAGTTGGGCACAGCAAATCACCTGTTCTGTTCAATGAAGCATAC

AGATCACTTGGTTTTAATGGGGTATATGTGCACTTATTAGTTGATGACCTGGCAAGTTTT

CTCAAGACTTACTCATCTCCGGATTTTGCAGGATTCAGTTGTACGATTCCTCACAAAGAG

GCTGCTGTGAAGTGTTGCGATGAAGTTGATCCAGTTGCAAAGTCGATAGGAGCTGTTAAT

TGCATTGTAAGGAGAGAAAGTGATGGGAAGTTATTTGGTTTCAATACAGACTATGTTGGT

GCAATTTCTGCAATTGACGATGGACTTCAAGCTTCACGAAATGGTAGCAGTCAATCTGGT

TCACCCTTAGCTGGTAAGGTGTTTGTGGTCATTGGCGCTGGAGGTGCTGGTAAAGCACTA

GCTTATGGTGCAAAAGAAAAGGGAGCAAAGGTCATCATTGCCAATCGTACCTACGGTAGA

GCAAAGGAGCTTGCTGACATGATTGGAGGAGAAGCCATCTCTCTAGCTGATCTAGAGAAT

TTCCATCCAGAAAGTGGCATGATTCTTGCGAACACAACATCAATTGGAATGGAGCCGAAA

GTTGATGAAACACCCGTCCCCAAGCGTGCATTGAGACATTATGCCCTGGTATTCGATGCC

GTTTACACCCCGAAAATTACCAGGATGCTGAGAGAAGCTGAGGAGTGGGGAGCCACTGTA

GTGTCGGGACTGGAGATGTTCATTGGGCAAGCGTACCAGCAGTTTGAGAGCTCCCAAGGA

ACTTTTCCGCAAGACCATGTCCAATTACTCGACTTCCTCAAGAACGACGAACTTGAAGAT

ATCAAACCGATCCGAATCGATTGGCTAAAGGACCTAGCGAGGACGGCGGAGGGGATGGAA

AGTCGTCAAACACAGCGGGAAATTGGGGGAAGACGACTGGTGAATACTCATCTTGGTGTC

GACCGGTCATCGGGGGCTTGCAGGAAAAGTCATCGACTGATCACAGAGTATCGAAGGAAA

AGATGTCGGCCGGTCACCGGAGCATCATCGGAAAAGATGTTGGCAGTCACCGATGACGTG

GCAGTGGCTTCAAATTAA

**AFSQ01026985**

ATGGGTCTGATGGGTCGGGTCGGGAGTCGGGTTGCTGCTCCGTTGGTTTGTGCGACGGTG

ATGAAGAAATCCGTTGAGCAAGTGGTGGATGAAATGCAGGCTGCCAAGGCTCAAGGTGCT

GACGTTGTGGAGGTCAGGCTTGACGTCATCAATGGCGGTTCTTCTTCTGGTGAGCTGGAA

ATCTTGTTGCGGGATAAGGCATTGCCTGTTATCATTGTTTGCAGGCCTAAAGGGGAAGGT

GGTGAGTATGAAGGAGAAGAGAATTCAAGGTTGGAAACTCTTTACTTGGCTTCTCAATTG

GGTGCTGTAGGATCTTTGTTCGGTATAGCTGCTTCTTCTGGTGTGATGGAAGAACTGAGG

AAGAGAAGGCATTTCAACAGCAAGATCATTGTGTCCGGCAATTTGAATGGTCCAACTTCT

TCAAAGGAAAATCTGACCAAGTTGGTTAGATCCATGGCGGCTCTGGAACCAGACATCATC

AAGATTGTATCGAATGTGGATAGCATCACGGAGATTGAGAGGATCTTTCATCTGACTTCC

AATTCTCAGGCATGGACATTATTCCACTGTTTATGTTTTTGTGACATCGTTCTACCGTTA

GACTCACTGGTGCCAATAGTTGCATACTCGGCCGGGGAAAGAGGTCTGATAAGTCAAATA

CTGACCCCCAAATACGGTGGAGCTTTAGTCTATGGATCCATGGAAGGAAAACCGGTTCCC

GGGTTGCCTACTTTAACCAGCTTGAGAGAAGAAGCCTATGAAGTTGAGTGCATCAACTCA

GAGACACAAGTTTTCGGGTTGATTTCGAAACCGGTTGGCCACAGTAAAGGCCCCTTGCTG

CATAATCCTGCTTTCAGACATGTGGAGTACAATGGAGTCTATGTCCCTATGTTCGTTGAT

GATCTCAGAGAGTTCTTTGAAGCTTATTCTCATCCTGACTTCCATGGATTCAGTGTTGGG

TTTCCATACAAAGAAGCTGTTGTGGAGTTCTGTGATGAAGTCCATCCACTTGCCAAGACT

ATAGGTGCTGTTAACACCATAGTGAGAAGAACAAGTGATGGGAAGTTGATAGGTTACAAT

ACAGACTGTGAAGCTTCAATAACAGCCATTGAAGATGCTCTCAAAGAACAGGGAGACTCC

AATGGAAGTGTGGTGGCTTCTTCCAAGTCTCCACTCGCAGGGAAGCAGTTTGTCCTTGTT

GGAGCTGGAGGAGCTGGAAGGGCGCTTGGCTTTGGTGCCAAGACAAGAGGAGCCCGTGTC

TTAATTTTCGACATTGATTTCTTGAGGGCCAAGTCACTGGCTAAGGATGTCTATGGCGAA

GCACGCCCTTTTGAGACCCTGCCAAGTTTCGAGCCTGAGGATGGTGCAATTCTTGCTAAT

GCCACACCGGTAGGGATGCATCCGAGCACGGACAGGATCCCTGTTGCTCAGGCGAGCTTG

GCGAGATACAGAGTGGTGTTCGATGCAGTGTACACTCCGAGGAAGACGAGACTGTTGAAA

GATGCTGATGCTGCTGGTGCAATCATTGTGAGTGGAGTTGAGATGTTCCTTAGACAAGCC

ATTGGACAGTTCAAACTCTTCACTGGCAAACAAGCACCTGAAGATTTCATGAGGGAGATT

GTTATGGCCAAGTTCCGAGTTCATCACAAAAAAACTATACAGTAA

**AFSQ01022417**

ATGGGTCTGATGGGTCGGGTCGGGAGTCGGGTTGCTCTGCCTCCGTTGGTTTGTGCGACG

GTGATGGGGGATTCGGTTGAGCAAGTGGTGGATGAAATGCAGGCTGCCAAGGCTCAAGGT

GCTGACGTTGTGGAGGTCAGGCTTGACGTCATCAATGGCGGTTCTTCTTCTGGTGAGCTG

GAAATCTTGTTGCGGGATAAGGCATTGCCTGTTATCATTGTTTGCAGGCCTAAATGGGAA

GGTGGTCATTATGAAGGAGAAGAGAACTCAAGGTTGGAAACTCTTTACTTGGCTTCTCAA

ATGGGAGCTGATTACATTGACATTGAGCACAAGGCTTCTTCTTCTGGTGTGATGGAAGAA

CTGAGGAAGAGGAGCCATTTCAACAGCAAGATCATTGTGTCCTGCAATTTGAATGGTCCA

ACTCCTTCAAAGGAAAATCTGACCAAGTTGGTTAGATCCATGGCGGCTCTGGAACCGGAC

ATCATCAGGATTGTATCGAATGTGGATAGCATCACGGAGATTGAGAGGATCTTCCATCTG

ACTTCCAATTCTCAGGCATGGACACTATTCCACTGTTTATTTTTTTGTGACATCGTTCTA

CCGTTAGACTCACTAGTTCCAATAGTTGCATACTCGGCCGGGGAAAGAGGTCTGATAAGT

CAAATTCTGACCCCAAAATACGGTGGAGCTTTAGTCTATGGATCCATGGAAGGAAAACCG

GTTCCCGGGTTGCCTACTTTAACCAGCTTGAGAGAAGAAGCCTATGAAGTTGAGTGCATC

AACTCAGAGACACAAGTTTTCGGGTTGATTTCGAAACCGGTTGGCCACAGTAAAGGCCCC

TTGCTGCATAATCCTGCTTTCAGACATGTGGAGTACAATGGAGTCTATGTCCCTATGTTC

GTCGATGATCTCAGAGAGTTCTTTGAGGCTTATTCTCATCCTGACTTCCATGGATTCAGT

GTTGGGTTTCCATACAAAGAAGCTGTTGTGGAGTTCTGTGATGAAGTCCATCCACTTGCC

AAGACTATAGGTGCTGTTAATACCATAGTGAGAAGAACAAGTGATGGGAAGTTGATAGGT

TACAATACAGACTGTGAAGCTTCAATAACAGCCATTGAAGATGCTCTCAAAGAACAGGGA

GACTCCAATGGAAGTGTGGTGGCTTCTTCCAAGTCTCCACTCGCAGGGAAGCAGTTTGTC

CTTGTTGGAGCTGGAGGAGCTGGAAGGGCGCTTGGCTTTGGTGCCAAGACAAGAGGAGCC

CGTGTCTTAATTTTCGACATTGATTTCTTGAGGGCCAAGTCACTAGCTAAGGATGTCTGT

GGTGAAGCATGCCCTTTTGAGACCCTGCCAAGTTTCGAGCCTGAGGATGGTGCAATTCTT

GCTAATGCCACACCGGTAGGGATGCATCCGAGCACGGACAGGATCCCTGTTGCTCAGGCA

AGCTTGGCGAGATACAGAGTGGTGTTCGATGCAGTGTACACTCCGAGGAAGACGAGACTG

TTGAAAGATGCTGATGCTGCTGGTGCAATCATTGTGAGTGGAGTTGAGATGTTCCTTAGA

CAAGCCATTGGACAGTTCAAACTCTTCACTGGCAAACAAGCACCTGAAGATTTCATGAGG

GAGATTGTTATGGCCAAGTTCTGA

**CHORISMATE SYNTHASE**

**AFSQ01008633**

ATGAATCAGGCTGACTGGGGGTTAGGTAAGGGTGGAAGAGTAAAGAAAGATAAATTCCAA

AACAGATGCGCGTTGCGACGGTTGCTGCCCCATTTTCAAAGTGGCTGTGGCTCGTTTGAG

AGACGTGAGCAGGCGGAGCTCATAGATGATTCCGACGCGGGATCATCTTCTTCTTCCCCG

ACTACGACGACCTCAAACTCTAAGAGGCCAGAGTTAGGTTCTTCGGCTACCAACCCCATT

TCTTTCTCCCCTAAATCTCCCCTCCATTTTCCTTATTGGATCTCCGCTTTTTACCGCCAT

CTCCTTCCCCAGCTTCCTGCATTCTCCTTTCCATGGCTTCTTCTTCTCTCGCTTCTAAAC

AGTTCCTCGCCGCTTCCAAATCCAATGGATTCGCTTACTCTGATCTCCGCAAGCTCTCCC

TCCCCACCGTTCAAATCTCCGTCCGCCCTCGCACTCGGAGGAACCTCCAACACGACGAGA

CTAGGAGAAATAAATGCTGCTGGGAGTTCATATGGCAATCACTTCCGCGTTACCACATTT

GGTGAATCTCATGGTGGTGGTGTTGGATGTATTATTGATGGATGTCCTCCAAGGCTACCC

CTTGCTGAAGCTGATATGCAAGTAGATCTTGATAGAAGGAGGCCTGGTCAGAGCCGAATT

ACCACGCCTAGGAAGGAGACTGATACCTGCAAAATATCTTCTGGTGTTGCAGAAGGTTTT

ACTACTGGAACGCCAATCCATGTGTTCGTACCAAATACTGACCAAAGAGGTCATGATTAC

AAGGAAATGTCTCAAGCTTATAGGCCTTCTCATGCTGATGCAACTTATGACATAAAATAT

GGTGTCAGATCAGTTCAGGGTGGTGGCCGATCTTCTGCAAGAGAAACCATTGGAAGAGTT

GCAGCTGGAGCTGTTGCGAAAAAGATTCTAAAGCAATTTGCTGGGACGGAGGTTCTTGCA

TACGTTTCTCAAGTGCACAAAGTTGTTCTTCCCGAGGATGTGGTTGATCATGAATTGTTG

ACACTTGATCAGATAGAGAGCAATATTGTGAGGTGTCCAGATCCTGAGTATGCAGAGAAA

ATGATTAATGCTATTGACACTGTCCGTGTGAAAGGAGATTCAGTTGGTGGAGTTGTAACT

TGTATTGTGAGGAATGCGCCACGTGGACTTGGTTCGCCGGTGTTTGATAAACTTGAAGCT

GAGCTGGCTAAAGCTGCTATGTCGCTACCTGCAACGAAAGGTTTTGAATTTGGAAGCGGG

TTTGCAGGTACATATCTAACCGGTAGTGAACATAATGATGAGTTCTACATGGCTGAAGAC

GGTAAAATAAGGACGCGAACAAACCGTTCTGGTGGAATACAGGGAGGAATATCAAATGGT

GAAATCATACACATGAGAATAGGTTTCAAGCCAACATCAACCATTGCCAAGAAGCAAAAC

ACAGTGACAAGAGATAAACATGAGACGGAGCTAATAGCTCGCGGCCGTCATGATCCATGT

GTAGTCCCACGAGCGGTTCCGATGGTGGAAGCAATGGTGGCACTGGTGCTAGTGGATCAG

CTAATGGCACAATTTGCCCAAAACTACATATTCCCAATCAATCAAGAACTACAAGAACCC

TTTGGAACAAAGCTGGAGCCTGCCAATGTCTGA

**AFSQ01019823**

ATGGCTTCTGCTTCTTCTTCTCTCGCTTCTAAGCAGTTCCTCGCCTCTTCCAAATTCGAT

GGCCTCTCTTCCTCTGATCTCCGCAAGCTCTCCCTCCCAGCAGTTCAAATCTCCATCCGC

CCTCGCACACGGAGGACCTTCCAAATAAATGCTGCCGGGAGTTCATATGGCAATCACTTC

CGCGTTACCACATTTGGGGAATCTCATGGTGGTGGTGTTGGATGTATTATTGATGGATGC

CCTCCGAGACTACCCCTCGCGGAAGCTGATATGCAAGTAGATCTTGATAGAAGGAGGCCT

GGTCAGAGCCGAATTACCACGCCTAGGAAGGAGACTGATACCTGCAAAATATCTTCTGGT

GTTGCAGAAGGTTTTACTACCGGAACGCCAATCCATGTGTTCGTACCAAATACTGACCAA

AGAGGTCATGATTACAAGGAAATGTCTCAAGCTTATAGGCCTTCTCATGCTGATGCAACT

TATGACATGAAATATGGTGTCAGATCAGTTCAGGGTGGTGGCCGATCTTCTGCAAGAGAA

ACCATTGGAAGAGTTGCAGCTGGAGCTGTTGCGAAAAAGATTCTAAAGCAGTTTGCTGGC

ACGGAGGTTCTTGCATACGTTTCTCAAGTGCACAAAGTTGTTCTTCCCGAGGATGTGGTT

GATCATGAATTGTTGACACTTGACCAGATAGAGAGCAATATTGTGAGGTGTCCAGATCCT

GAGTATGCAGAGAAGATGATTAATGCTATTGACACCGTCCGTGTGAAAGGAGATTCAGTT

GGTGGAGTTGTAACTTGTATTGTGAGGAATGCGCCACGTGGGCTTGGTTCGCCGGTGTTT

GATAAACTTGAAGCTGAGCTGGCTAAAGCTGCCATGTCGCTACCTGCAACGAAAGGTTTT

GAATTTGGAAGCGGGTTTGCAGGTACATATCTAACCGGTAGTGAACATAATGATGAGTTC

TACATGGCTGAAGACGGTCAAATAAGGACGCGAACAAACCGTTCTGGTGGGATACAGGGA

GGAATATCAAATGGTGAAATCATACACATGAGAATAGGTTTCAAGCCAACATCAACCATT

ACCAAGAAGCAAAACACAGTGACAAGAGATAAACATGAGACGGAGCTAATAGCTCGCGGT

CGTCATGATCCATGTGTAGTCCCACGAGCGGTTCCAATGGTGGAAGCAATGGTGGCACTG

GTGCTAGTGGATCAGCTAATGGCACAATTTGCCCAAAACTACATATTCCCAATCAATCAG

GAACTACAAGAACCCTTTGGAACAAAGCTAGAGCCTGCCAATGTCTGA

**CHORISMATE MUTASE**

**AFSQ01015214**

ATGGAGTCCCAACTTCTGATGAGAGATTCCCCTTCTCCCGCAATTCATTCTCCTACTACC

AAGTTGTTTGTTCCTTTCTGCCGTCTTTCTCCGCTCGGAACAACCAGGACTTGGTCTAGG

CTCCCGCTTCCTTCTTCTTCAAATCTTGCTAAGCGTGTCATCATCCTCTGCGCAAACTCT

TCCCCGCCTTCTCCTGCGCTGGAGAAGAAGAAAAGGGTTGACGAAAGTGAAACTTTGACA

CTAGACAGCGTAAGGCGTTCCTTGATACGTCAAGAAGACAGTATCATATACAGCCTTTTG

GAGAGGTCTCAATACTGTTACAATGCAGACACCTATAATCCTGATGCTTTCTCCATGGAT

AATTTTCACGGTTCTCTTGTCGAGTACATGGTTAAAGAAACTGAAAAGCTGCACGCCAAG

GTGGGTAGGTACAGAAGTCCTGATGAGCATCCTTTCTTCCCAGATGATTTACCTGAACCA

TTGTTGCCACCAATGCAGTATCCCAAGGTGCTACATGATATTGCAGATTCAGTGAATATA

AATAGTACCATCTGGAGCATGTACTTTAACGATCTTCTTCCAAGGCTGGTTAAGGCAGGA

GATGATGGAAACTGTGGGTCCACAGCAGTATGCGACACGATCTGCGTCCAGGCTCTGTCC

AAGCGAATCCATTATGGAAAATATGTAGCCGAGTCTAAATTTCGTGAGAACCCAAATGAA

TACAGAGCTGCTATAGAAGCACAAGACAAGGAGGGATTGATGGCCTTGCTGACGTACCCA

GACGTTGAGAAAAAGGAAGAAGAGCGAGAAGAGGTGAAAGCCAGGACATTTGCGCAGGAA

GTAACAATGAAAGGAGGAAATTGTGAATGCGAACCAGTATACAAGATAGACACAAGCTTG

GTTGCTGATCTGTATGGGAAATGGATCATGCCTCTGACCAAGGAAGTTCAAGTTATGTAC

CTGTTGAGAAGGCTGGATTGA

**AFSQ01018652**

ATGGAGTCCCAACTTCTAATGAGAGCTTCAGCACCTCACACCTCCAAGTTGTCTGTTCCT

TTCTGCCGTCTTTCTCCGCTCGGAACAACCAGGACTTGGTCTAGGCTCCCGCTTCCTTCT

TCATCAAATCTTGCTAAGCGTGTCATCATCCTCTGCGCAAACTCTTCTCAGCCTTCTCCT

GCCTTGGCGAAGAAGAAAAGGGTTGATGAAAGTGAAACTTTGACACTAGACAGCGTAAGG

CGTTCCTTGATACGTCAAGAAGACAGTATCATATACAGCCTTTTGGAGAGGTCTCAATAC

TGTTACAATGCAGACACCTATAATCCTGATGCTTTCTCCATGGATAATTTTCACGGTTCT

CTTGTTGAGTACATGGTTAAAGAAACTGAAAAGCTGCATGCCAAGGTGCTACATGATATT

GCTGATTCAGTGAATATAAATAGTACCATCTGGAGCATGTACTTTAACGATCTTCTTCCA

AGGCTGGTCAAGGCAGGAGATGATGGAAACTGTGGGTCCACAGCAGTATGCGACACGATC

TGCGTCCAGGCTCTGTCCAAGCGAATCCATTATGGAAAATATGTAGCCGAGTCTAAATTT

CGTGAGAACCCAAATGAATACAGAGCTGCTATAGAAGCACAAGACAAGGAGGGATTGATG

GCCTTGCTGACGTACCCAGAAGTTGAGAAAAAGGTAGAAGAGCGAGTAGAGGTGAAAGCC

AGGACATTTGCGCAGGAAGTGACAATGAAAGAAGGAAATGCTGAATGCGAACCAGTATAC

AAGATAGACCCAAGCTTGGTTGCTGATCTGTATGGGAAATGGATCATGCCTCTGACCAAG

GAAGTTCAAGAAAGAAGGGGAACAGTTCCAAATGTCTCAGTCGCTCTATATAGTGAAGTC

AAGAGAGTTATAGTTTGTTATAAACCCATGTTGGTAGAACCCATTATTTGTTGCAGAGAG

GTGGAAGTTAGCATCAATGGAGGAAAGGCTAATACCGTTGAAGTCACTTGTGCTAACGGT

GGTGTTTGTTGCTGCTATGGTGGGGGAAGTTTCAGGTACAAGATGGATTGTAGGATCCAA

CATGGATGGGTTGGACTACTAATGTCAATTACACTATCTGGGCACAGGATAAACACTTCT

AGGATGGGGACTGGCTCTGTGAACAAGAGTGATGACGAAGAGTTGCAACTCAGAGAATCC

AAATAA

**AFSQ01005960**

ATGGAGGCCAAACTTCTGATGAGAGCTTCCCCTTCTACTGCAATTCATTCTCCTACTACT

CATCCTGCCTCCAAGTTGCCTGTTCGATTGATCTGCCGTCTTTCTCCGCTTCCTTCTTCA

TCAAATCTTGCCAAGTGCGGCATCATCCTCTGCGCAAAGTCTTCTCCTGCGTATGCTCTA

ACCCCCTTTCCCTCAATTTTGCTCTTCTTTTTCTTTGTGATTAACACCTATAATCCTTAT

GCTTTCCCCATGGATGACTTCCACGGTTCTCTTGTTGAGTACATGGTTAAAGAAACTGAA

AAGCGACATGCCAAGGTGGGTAGGTACAGAAGTCCTGATGAGCATCCTTTCTTCCCAGAT

GATTTACCTGAACCATTGTTGCCACCAATGCACTATCCTAAGGTGTCCGATTCAGTGAAT

ATAAATAGTACCATCTGGGGCATGTACTTCAATGATCTTCTTCCAGGACTGGTCAAGGCA

GGAGATGATGGAAACTGTGGATCATCTGCAGTATGCGACACCATCTGCCTCCAGGCTCTG

TCAAAGCGAATCCATTATGGAAAATATGTGGCCGAGTCTAAATTCCATGCGAACCCAAAT

GAATACCAAGCTGCTATAGAAGCACAAGACAAAGAGGGATTGATGGCCTTGCTGACGTAC

CCAGAAGTTGAGAAAAAGGTAGTGGACCGAGTAGAGCTGAAAGCCAAGATATTTGCGCAG

GAAGTGACAATGAAAGAAGGGAAATTAGCTGAATGCGAGCCAGTGTACAAGATAGACCCA

TGCTTGGTTGCTCATCTTTATGGGAAATGGATCATGCCTCTGACCAAGGAAGTTCAAGTT

AAGTACTTGCTGAGAAGGCTGGATTGA

**PHENYLALANINE AMMONIA LYASE**

**AFSQ01008390/91**

ATGTACGAAAGAGAAAGTAAAGGGAGTGATAGAGCAGTTATCCATAAAACAAAAAGAGTA

GATTCCCTCCCTCCGATCAGACATGGCACCAACACCAACCGCAGCAACCATTGCCCAGAA

CGGCAACCACCACCACACCAACGGCTCCCCCCGCCCGGCGCGTTGGGCCATTTGTGCCCC

ACCGCCCCTTCCGCCACCGGCAACCACTACCAAACCGACCCACTCAACTGGGGCGCCGCC

GCTGATGCCATGAAAGGGAGCCACCTTGACGAGGTGAAACGGATGGTGGCAGAGTACCGG

AAGCCTTCGGTCAAACTGGTCGGGGAGTCGCTCACCATCGCTCAGGTCGCCGCGATCGCC

GCCGGAAATGCCCCCCATGTCACGGTGGAGCTCTCCGAATCCGCCAGGGACCGCGTCAAA

GCCAGCAGCGACTGGGTCATGGAGAGCATGAACAAAGGTACTGACAGCTACGGCGTCACC

ACCGGATTCGGCGCCACTTCTCATCGGAGGACCAAACAAGGCGGCGCCCTTCAGAAGGAG

CTCATCAGGTTCTTGAATGCTGGAATCTTCGGCAACGGTACTGAATCAAGCCATACTCTG

CCTCACTCGGCCACAAGAGCAGCTATGCTTGTCAGGATCAACACTCTCCTCCAGGGATAC

TCCGGGATTCGATTCGAGATCCTGGAAGCCATCACCAAGCTTCTCAACAGCAACATTACT

CCATGTTTGCCACTCCGCGGAACAATCACAGCTTCCGGTGACTTGGTACCACTCTCTTAT

ATTGCTGGTTTGCTAACCGGAAGGCCTAACTCGAAAGCCACCGGTCCGAACGGTGAGATC

ATGGATGCCACCGAAGCATTCCGTTTGGCTGGGATCGATTCCGGTTTCTTCGAGTTGCAG

CCGAAAGAAGGGCTTGCTCTTGTCAATGGTACAGCTGTTGGATCTGGTTTGGCATCCATG

GTTCTGTTCGAAGCCAATGTTCTTTCTGTACTGTCTGAGATATTATCAGCCATTTTCGCG

GAGGTTATGAACGGGAAGCCAGAGTTCACTGACCATTTGACTCACAAGTTGAAGCATCAT

CCGGGGCAGATCGAAGCTGCAGCAATAATGGAGCATATTCTCGACGGGAGTGCTTACATG

AAGGCAGCCAAGAAGTTGCACGAGATCGATCCTCTCCAGAAGCCGAAGCAGGATCGTTAC

GCTCTCAGGACTTCGCCTCAATGGCTAGGTCCTCAGATCGAAGTCATCAGATTCTCGACC

AAGTCGATTGAGAGAGAGATCAACTCGGTGAACGACAACCCTCTGATCGATGTATCCAGG

AGCAAGGCCATTCATGGTGGCAACTTCCAGGGAACTCCCATCGGAGTGTCAATGGACAAT

GTCCGGTTGGCGATTGCTTCTATCGGGAAGTTAATGTTCGCTCAGTTCAGTGAGCTAGTC

AATGATTTCTACAACAATGGACTGCCATCGAATCTCACGGCCAGCAGGAACCCGAGCTTG

GACTATGGTTTCAAAGGAGCTGAAATCGCCATGGCCTCCTACTGCTCCGAGCTCCAATAC

CTTGCAAATCCTGTCACGACACATGTTCAGAGCGCGGAGCAGCACAACCAGGACGTGAAC

TCGTTGGGATTGATCTCTTCGAGGAAGACCGCCGAAGCTGTGGATATCCTAAAGTTGATG

TCTTCCACTTACTTGGTGGGACTTTGTCAAGCTGTTGACTTGAGGCACTTGGAAGAGAAT

CTCAGGAGTGCAGTGAAGAACACTGTGAGCCAAGTGGCTAAGAAGGTTTTAACAATGGGG

TCGAACGGAGAGCTCCACCCATCGAGGTTCTGCGAGAAGGACTTGCTTCAGGTGGTCGAT

CACGAATACGTGTTTGCATATGCTGACGATGCTTGCAGCGCAAGCTACCCGCTGATGCAG

AAGCTGAGACAGGTTTTGGTAGACCATGCATTGGCCAATGGAGAGAGTGAGACGAATGCT

AGCACTTCAGTGTTCCAAAAGATTGTAGCTTTCGAGGAAGAGCTGAAAACCCTTCTTCCT

AAAGAAGTGGAGAGTGTTAGGGCAGCTTATGAAAGTGGTAAAGCTCCTATCCCTAACAAG

ATATTGGAGTGCAGATCTTACCCGTTGTATAAATTTGTGAGGGAGGAGCTCGGGACGGCT

TTGCTGACCGGAGAGAAAGTTATGTCGCCCGGGGAGGAATTCGACAAAGTGTTCACTGCA

TTGTGCGAGGGGAAGATCATCGATCCGATGATGGATTGCCTCAACGAATGGAATGGTGCC

CCTATCCCAATCTGCTAA

**AFSQ01000737**

ATGTACGAAAGGGAAAGTAAAGGGAGTGATAAAGCAGTTATCCATAAAACGAAAAGAGAT

TCCCTCCCTCCGATCAGACATGGCACCAACACCAACCGCAGCAACCATTCCCCAGAACGG

CAACCACCACCACACCAACGGCCAACCCCCCACCACACCAACGGCTCGTCGGTGTCGTCG

TTGGGCCATTTGTGCCCCACCGCCCCTTCCGCCACCGGCAACCACTACCAAACCGACCCT

CTTAACTGGGGCGCCGCTGCCGATTCCATGAAAGGAAGCCACCTCGACGAGGTGAAGCGG

ATGGTGGCAGAGTACCGGAAGCCTTCCGTCAAACTGGTCGGAGAGTCGCTCACCATCGCT

CAAGTCGCCGCGATCGCCGCCGGAAATGCCCCCCATGTCACGGTGGAGCTCTCCGAATCC

GCCAGGGACCGCGTCAAAGCCAGCAGCGACTGGGTCATGGAGAGCATGAACAAAGGTACT

GATAGCTACGGCGTCACCACCGGATTCGGCGCCACTTCTCATCGGAGGACCAAACAAGGA

GGCGCCCTTCAGAAGGAGCTCATCAGGTTCTTGAATGCTGGAATCTTCGGCAACGGTACT

GAATCAAGCCATACTCTGCCTCACTCGGCCACAAGAGCAGCTATGCTTGTCAGGATCAAC

ACTCTCCTCCAGGGATACTCCGGGATTCGATTCGAGATCCTGGAAGCCATCACCAAGCTT

CTCAACAGCAACATTACTCCATGTTTGCCACTCCGCGGAACAATCACAGCTTCCGGTGAC

TTGGTACCACTCTCTTACATTGCTGGTTTGCTAACCGGAAGGCCTAACTCGAAAGCCACC

GGTCCGGACGGTGAGATCATGGATGCTACCGAGGCATTCCGTTTGGCTGGGATCGAGTCC

GGTTTCTTCGAGTTGCAGCCGAAAGAAGGGCTTGCTCTTGTCAATGGTACAGCTGTTGGA

TCTGGTTTGGCATCCATGGTTCTGTTCGAAGCCAATGTTCTTTCTGTACTGTCTGAGATT

TTATCAGCCATTTTCGCTGAGGTTATGAACGGGAAGCCAGAGTTCACTGACCATTTGACG

CCAGCAGGAACCCGAGCTTGGACTATGAGCGCGGAGCAGCACAACCAGGACGTGAACTCG

TTGGGATTGATCTCTTCGAGGAAGACCGCCGAAGCTGTGGATATCCTAAAGTTGATGTCT

TCCACTTACTTGGTGGGACTTTGTCAAGCTGTTGACTTGAGGCACTTGGAAGAGAATCTC

AGGAGTGCAGTGAAGAACACTGTGAGCCAAGTGGCTAAGAAGGTTTTAACAATGGGGTCG

AACGGAGAGCTCCACCCATCGAGGTTCTGCGAGAAAGACTTGCTCCAAGTTGTCGATCAC

GAATACGTGTTTGCATATGCTGACGATGCTTGCAGCGCAAGCTACCCACTGATGCAGAAG

CTGAGACAGGTTTTGGTGGAGCACGCATTGGCTAATGGCGAGAGTGAGACGAATGCTAGC

ACTTCAGTGTTCCAAAAGATTGTAGCTTTCGAGGAAGAGCTGAAAACCCTTCTTCCTAAA

GAAGTGGAGAGTGTTAGGGCAGCTTACGAAAGTGGTAAAGCTCCCATCCCTAACAAGATC

TTGGAGTGCAGATCTTACCCGTTGTACAAATTCGTGAGGGAGGAGCTCGGAACGGCGTTG

CTCACCGGAGAGAAAGTTATGTCGCCTGGGGAGGAGTTCGACAAAGTGTTCACCGCATTG

TGCGAGGGGAAGATCATCGATCCGATGATGGACTGCCTCAACGAATGGAATGGTGCCCCT

ATCCCAATTTGCTAA

**AFSQ01012642**

ATGGAGCTCTGCAGCAACAACGAGAACAACAACAATGGCGGTGGGTTTTCCCTCGCTGAT

CCGTTGAACTGGGGAATGGCCGCAGAGTCAATGAAAGGGAGCCACTTGGAGGAGGTGAAG

CGTATGGTTGCAGAGTCGAGATCCCCTGTCGTGAAGCTTGCCGGGCAGACTCTGTCCATT

GCCCAAGTCGTCGCAATTTCCCGCTCTGACGCGGCTGCCGAGCTGGACGAGGAGGCCCGG

CCGAGGGTGAAGGCCAGCAGCGACTGGGTCATGGAGAGCATGAACAAAGGCACGGACAGT

TACGGTGTCACGACTGGGTTCGGAGCCACGTCTCATCGTAGGACTAAGCAGGGTGGAGCC

CTTCAGAGGGAGCTGATCAGGTTCTTGAACGCTGGCATATTCGGCAACGGTACGGAGTCA

ACCCACACGCTGCCATACTCCGCAACCAGGGCAGCCATGCTGGTCAGGATCAACACCTTG

CTCCAGGGCTATTCAGGAATCCGGTTCGAAATTCTCGAAGCCATCACCAAGCTCCTCAAT

AACAATGTCACCCCATGTCTACCTCTCAGGGGCACCATCACCGCCTCTGGTGACTTGGTC

CCTCTATCCTACATTGCAGGCCTCTTAACCGGCCGCCCCAACTCCAAAGCAGTCGGCCCC

AAAGGCGAGTCCCTGAACGCGACACAAGCCTTTGAAGCTGCTGGGATCACCGGTGGATTC

TTCGAGTTGCAGCCAAAGGAAGGTCTGGCAATGGTGAACGGTACTGCAGTTGGTTCGGGC

ATGGCCTCGATGGTCCTTTTCGACGCCAACATTTTGGCGGTGATGTCCGAGGTTTTGTCA

GCGATTTTCGCTGAAGTCATGCAAGGGAAACCCGAGTTCACAGACCACTTGACGCATAAG

CTGAAGCATCACCCGGGCCAGATTGAAGCTGCAGCAATCATGGAACATATCCTGAAGGGC

AGCCCTTACGTCAAGGAAGCTGAGAAATTGCACGAAATTGATCCGTTACAGAAGCCGAAA

CAGGACAGGTACGCTCTCAGGACATCTCCTCAATGGCTCGGCCCCCTGATCGAAGTGATC

AGGTCATCGACCAAAATGATTGAAAGGGAGATCAACTCGGTCAACGACAACCCGTTGATC

GATGTCTCGAGAGGGAAGGCAATCCACGGAGGGAACTTCCAGGGAACACCGATCGGTGTC

TCGATGGACAACACTCGTTTGGCAGTTGCTTCAATTGGGAAGCTCATGTTTGCGCAGTTC

TCCGAACTTGTCAACGACTTTTACAACAACGGTCTGCCATCGAACTTAACAGGTGGACGA

AACCCCAGCTTGGACTACGGGTTCAAGGGGGCTGAAATCGCAATGGCATCCTACTGCTCC

GAGCTTCAGTTCCTGGCAAATCCAGTTACAAACCACGTACAGAGCGCTGAACAACACAAC

CAAGATGTCAACTCATTGGGACTGATTTCGGCTAGGAAGACTGCCGAAGCTGTGGACATA

TTAAAGCTGATGTCGACAACTTACTTGGTCGCTCTATGCCAAGCGGTCGACTTAAGGTAT

ATCGAGGAGAATCTGAAGCAGACAGTTAAGAACACGATCAGCCAAGTAGCGAAGAAAGTA

CTCCTAACCAACGGGGAGGTGCTCCACCCATCGAGATTCTGCGAGAAGGAGCTGTTGAAT

GTCGCGGAGAGGGAGTACTTGTTTGCATATGCTGACGATCCTTGCAGCGCAACCTACCCT

CTGATGCAGAAGCTGAGAGGGGTGCTGGTGGAGCACGCGTTGCTGAACGGTGAGAGCGAG

AAGGACTCGAGCACTTCCATCTTCCACAAGATTGCAGCTTTTGAGGCGGAGCTGAAGGCT

ATCCTGCCGAAAGAAGTCGAGAATGCGAGGAGCGAGCTAGAGAATGGGAACCCGGCTATT

CCGAACAGGATTAAAGAGTGCAGGTCGTATCCGTTGTACAAGTTTGTGAGGGAGGAAGTT

GGTACCGGTTTGCTGACGGGTGAGAAGGTTAAGTCACCTGGTGAGGAATTTGACAAGGTG

TTCACTGCTATGTGTGAAGGGAAGTTGATTGATCCTTTGTTGGAGTGCCTCAAAGAATGG

AATGGTGCTCCTCTTCCTATCTGCTAG

**TRANS-CINNAMATE 4-MONOOXYGENASE**

**AFSQ01009050**

ATGGATCTCCGCCTCCTGGAGAAGACACTCTTAGCTCTCTTCTCCGCCGTCGTCGTCGCC

ATTGTAATCTCCAAGCTCCGGGGCAAGCGATTCAAGCTCCCTCCCGGTCCCATACCCGTC

CCTCTCTTCGGCAACTGGCTCCAGGTCGGCGATGACCTCAACCACCGCAACCTTTCCGAC

CTCTCCAAGAGGTTCGGCGAGATCCTCCTCCTCCGCATGGGCCAGCTCAACCTCGTCGTC

GTCTCCTCCCCTGACCTCCCCAAGGAGGTCCTCCACACTCATGGAGTCGAGTTCGTCTCC

AGTACTCGCACCGTCGTCTTCGACATCTTCACCGGCAAGGGACAGGACATGGTGTCCACC

GTCTACGGCGAGCACAGGCGCAAGATGCGCCGGATGATGACCGTGCCGTTCTTCACCAAC

AAGGTCGTCCAGCAGAACCGCCAGGGGTGGGAGGACGAGGCCGCCGCAGTAGTCGAGGAC

GTGAAGAAGAATCCCGACGCCGCCACCAACGGAATCGTGCTCCGCCGCCGACTCCAGCTG

ATGATGTACAACAACATATTCCGGATCATGTTTGACCGGAGGTTCGAATCGGAGCAGGAT

CCGTTGTTTAATAAGCTCAAGGCATTGAACGGAGAGCGGAGTCGGTTGGCTCAGAGCTTC

GAGTACAACTATGGAGATTTCATTCCGATCTTGAGGCCTTTCTTGAGGGGCTATTTGAAG

GTCTGTGAGGAGGTTAAGAACAGGAGGCTTCAGCTATTCAAGGATTACTTCGTCGAGGAG

AGGAAGAGATTGATGAGCACAGCTGCGATGACGAACGAAGGGCTGAAGTGCGCCATGGAT

CACATTCTGGAAGCACAGCAGAAGGGTGAGATCAACGAAGACAACGCACTCTACATCGTC

GAGAACATCAACGTCGCCGATCTCCCACCTACCCCCTGCACATTTGTTTCAATGAAGAAA

ATGTCGCTTTTTCTTTCTGATGCTGCTGATGCTATCGAGACTACACTGTGGTCAATCGAG

TGGGGAATCGCCGAGCTCGTAAACCACCAACAAATCCAGAACAAGCTAAGGCAAGAACTC

GACACCGTTCTCGGACCAGACCACCAGATCACCGAGCCCGACATCCAGAAGCTGCCATAC

CTCCAGGCAGTGGTGAAGGAGACTCTCCGTCTCAGAATGGCCATCCCTCTGCTGGTCCCG

CACATGAACCTCCACGATGCCAAGCTCCGAGGATTCGACATCCCGGCAGAGAGCAAGATC

CTGGTGAACGCGTGGTGGCTGGCCAACAACCCCGAACAGTGGAAGAGGCCCGAGGAGTTC

AGACCGGAGAGGTTCCTGGAAGAGGAGGCTAAAGTCGAGGCCAACGGGAACGACTTCAGG

TACCTTCCGTTCGGAGTTGGGAGGAGGAGCTGCCCTGGAATCATTCTTGCACTCCCCATT

CTGGGGATTACGATCGGGCGTCTGGTGCAGAACTTCGAGCTGTTGCCTCCTCCCGGGCAG

TCCAAGCTTGATACGACTGAGAAAGGTGGTCAGTTTAGTTTGCATATCTTGAAGCACTCG

ACCATCGTGGCTAAGCCAAGATCGGTTTGA

**AFSQ01011791**

ATGGATCTCCGCCTCCTGGAGAAGACACTCTTAGCTCTCTTCTCCGCCGTCATCGTCGCC

ATTGTAATCTCTAAGCTCCGCGGCAAGCGATTCAAGCTCCCACCTGGTCCCATACCCGTC

CCTCTCTTCGGCAATTGGCTCCAGGTCGGCGATGACCTCAACCACCGCAACCTCACCGAC

CTCGCCAAGAGGTTCGGCGAGATCTTCCTCCTCCGTATGGGCCAGCGCAACCTCGTCGTC

GTCTCCTCCCCTGACCTCGCCAAGGAGGTCCTCCACACTCAGGGAGTCGAGTTCGGCTCC

AGGACTCGCAACGTCGTCTTCGACATCTTCACCGGCAAGGGACAGGACATGGTGTTCACC

GTCTACGGCGAGCACTGGCGCAAGATGCGCCGGATCATGACTGTTCCGTTCTTCACCAAC

AAGGTCGTCCAGCAGAACCGCCAGGGTTGGGAGGACGAGGCCGCCGCCGTCGTTGAGGAC

GTGAAGAAGAATCCCGAGGCCCCCACCAATGGAATCGTGCTCCGCCGCCGACTCCAGCTG

ATGATGTACAACAACATATTCCGGATCATGTTTGACCGGAGGTTCGAATCGGAGCAGGAT

CCGTTGTTTAATAAGCTCAAGGCATTGAACGGAGAGCGGAGTCGGTTGGCTCAGAGCTTC

GAGTATAACTATGGAGATTTTATCCCGATTTTGAGGCCTTTCTTGAGGGGCTATTTGAAG

GTCTGTGAGGAGGTCAAGAACAGGAGGCTTCAGCTATTCAAGGATTACTTCGTTGAGGAG

AGGAAGAGACTGATGAGCTCAAGTGCGATGACGAACGAAGGGCTGAAGTGCGCGATGGAT

CACATTCTGGAAGCACAGCAGAAGGGTGAGATCAACGAAGACAATGCACTCTACATCGTC

GAGAACATCAACGTCGCTGCTATCGAGACTACACTGTGGTCAATCGAGTGGGGGATCGCC

GAGCTCGTAAACCACCAACAAATCCAGAACAAGCTGAGGCAAGAACTCGACACCGTTCTG

GGACCAGACCACCAGATCACCGAGCCCGACATCCAGAAGCTGCCATACCTTCAGGCAGTG

GTCAAGGAGACTCTCCGTCTCAGGATGGCCATCCCTCTGCTGGTCCCACACATGAACCTC

CACGATGCCAAGCTCCGAGGATTCGACATCCCAGCAGAGAGCAAAATCCTGGTGAACGCG

TGGTGGCTGGCCAACAACCCCGAACAGTGGAAGAGGCCCGAGGAGTTCAGACCGGAGAGG

TTCTTGGAAGAGGAGGCTAAAGTCGAGGCCAACGGAAATGACTTCAGGTACCTCCCGTTC

GGAGTCGGGAGGAGGAGCTGCCCTGGAATCATTCTTGCACTCCCCATTTTGGGGATTACG

ATAGGGCGTCTGGTGCAGAACTTCGAGCTGTTGCCTCCTCCAGGGCAGTCAAAGCTGGAT

ACGACTGAGAAAGGTGGTCAGTTTAGTTTGCACATCTTGAAGCACTCGACCATCGTGGCT

AAGCCAAGATCGGTTTGA

**AFSQ01001250**

ATGGATCTCCTCCTCCTGGAGAAGACCTTGCTGGCCTTATTCTCCGCCGTTATCGTCGCC

ATTGTAATATCTAAGCTCCGCGGCAAGCGCTTCAAGCTCCCTCCCGGCCCATTCCCTGTC

CCAATCTTTGGCAATTGGCTCCAAGTCGGCGATGACCTCAACCACCGCAACCTAACCGAC

CTCGCCAAGAAATTCGGTGAGATCTTCCTCCTCCGCATGGGCCAGCGCAACCTCGTCGTC

GTGTCCTCCCCTGACCTCGCCAAGGAGGTCCTCCACACTCAGGGCGTCGAGTTCGGCTCC

CGTACTCGCAACGTCGTCTTCGACATCTTCACCGGCAAGGGACAGGATATGGTGTTCACC

GTATACGGCGAGCACTGGAGGAAGATGCGCCGGATCATGACCGTCCCGTTCTTCACCAAC

AAGGTCGTCCAGCAGAATCGCCAGGGGTGGGAGGCAGAGGCCGCCGCAGTGGTGGAGGAC

GTCAAGAAGGATCCGGAAGCCGCAGCTGGCGGAATCGTGCTCCGCCGCAGGCTCCAGCTG

ATGATGTACAACAACATTTTCCGCATCATGTTCGACCGACGATTCGATTCGATGGAGGAT

CCGTTGTTTAATAAGCTCAAGGCATTGAACGGGGAGCGGAGTCGGTTGGCTCAGAGCTTC

GAATACAACTATGGAGATTTCATCCCGATTTTGAGGCCGTTCTTGAGAGGCTATTTGAAG

GTGTGCAAGGATGTCAAGGATAGGAGGCTGCAGCTATTCAAGGACTACTTCATTGCAGAT

AGGAAGAAAGCGGTGAGCACGAAGGGGGAGATGAGTTGTGCGATGGATCATATCATGGAT

GCGCAGCAGAAGGGAGAAATCAACGAAGAAAACGTCCTCTACATCGTCGAGAACATCAAC

GTCGCCGTTGCTGGTGGAGCCATACTCGAGTTGAAAAATAATGAGGGGTTAGCCATTGAG

ACCACGTTGTGGTCAATCGAATGGGGGATTGCAGAGCTAGTCAACCATCCAGAGATCCAG

CAAAAGCTAAGGGAAGAACTGGACACGGTTTTGGGACCGGGTAACCAGGTGACCGAGCCC

GACGTCCAGAAGCTACCGTACCTTCAGGCAGTGGTCAAGGAAACCCTCCGTCTAAGAATG

GCAATCCCTCTGCTCGTCCCGCACATGAACCTCCACGACGCTAAGCTCCGTGGATTCGAC

ATCCCCGCCGAGAGCAAGATATTGGTGAACGCGTGGTGGCTGGCGAACAACCCTGAGCAA

TGGAAGAAACCTGAGGAGTTCAGGCCGGAAAGGTTCCTCGAGGAGGAGGCGAAGGTGGAG

GCGAATGGAAACGACTTCAGGTACCTTCCGTTCGGAGTTGGGAGGAGGAGTTGCCCTGGA

ATCATCCTTGCCCTGCCCATTTTGGGGATTACCATCGGACGTTTGGTGCAGAACTTCGAG

CTGTTGCCTCCTCCTGGACAGTCCAAGCTTGACACTTCTGAGAAAGGTGGACAGTTCAGC

TTGCATATATTGAAGCATTCGACCATTGTTGCTAAGCCAAGGATGGGAGGTGTTGTTTTG

ATCAATGCTTTAAAGTCGTTGTCGTCGTCGTCGTTTACCTTCTCTAGTCGATTTCACCTG

TTCGCCACACTAGATGACGAGGGGGTAAGGAGAATCGATTTTAACGGCGTCTCTGAACTA

AATCCGGTCATTGGGATGGTGACGAAGCCGACTTCCTTCAGTGACGACTACGACGGCTGT

AGATTTGAGCTATATGGCGACGGCGGCGGAGGTGGATCGTCCAACACGCCTATTTGA

**AFSQ01002629**

ATGGATCTCCTCCTCCTGGAGAAGACCTTGCTGGCCTTATTCTCCGCCGTTATCGTCGCC

ATTGTAATATCTAAGCTCCGCGGCAAGCGATTCAAGCTCCCTCCCGGCCCATTCCCTGTC

CCAATCTTTGGCAATTGGCTCCAAGTCGGCGATGACCTCAACCACCGCAACCTAACCGAC

CTCGCCAAGAAATTCGGTGAGATCTTCCTCCTCCGCATGGGCCAGCGCAACCTCGTCGTC

GTGTCCTCCCCTGACCTCGCCAAGGAGGTCCTCCACACACAGGGCGTCGAGTTCGGCTCC

CGGACTCGCAACGTAGTCTTCGACATCTTCACCGGGAAGGGACAGGATATGGTGTTCACC

GTCTACGGCGAGCACTGGAGGAAGATGCGCCGGATCATGACCGTCCCGTTCTTCACCAAC

AAGGTCGTCCAGCAGAACCGCCAGGGGTGGGAGGCAGAGGCCGCCGCCGTGGTGGAGGAC

GTCAAGAAGGATCCGCAAGCCGCCGCTGGCGGAATCGTGCTCCGACGCAGGCTCCAGCTG

ATGATGTACAACAACATTTTCCGCATCATGTTCGACCGACGATTCGATTCGATGGAGGAT

CCGTTGTTTAATAAGCTCAAGGCGTTGAACGGAGAGCGGAGTCGGTTGGCTCAGAGCTTC

GAATACAACTATGGAGATTTCATCCCGATTTTGAGGCCGTTTTTGAGAGGCTATTTGAAG

GTGTGCAAGGATGTCAAGGATAGGAGACTGCAGCTCTTCAAGGATTACTTCATTGCAGAT

AGGAAGAAAGCGGTGAGCACGAAGGGAGAGATGAGTTGTGCGATGGATCATATCATGGAT

GCGCAGCAGAAGGGAGAAATCAACGAAGACAACGTCCTCTACATCGTCGAGAACATCAAC

GTCGCCGGTGGAGCCGAAGTTGAAATATATTTAGTGGTTAGGTATGCCATTGAGACCACG

TTGTGGTCAATCGAGTGGGGGATCGCAGAGCTAGTCAACCATCCAGAGATTCAGCAAAAG

CTGAGGGAAGAACTGGACACAGTGTTGGGACCGGGTAACCAGGTGACCGAGCCCGACATA

CAGAAGCTACCATACCTCCAGGCGGTGGTCAAGGAAACCCTCCGTCTAAGAATGGCAATC

CCTCTGCTGGTCCCGCACATGAACCTCCACGATGCTAAGCTCCGTGGATTCGACATCCCT

GCAGAGAGCAAGATACTGGTGAACGCGTGGTGGCTGGCGAACAACCCTGAGCAATGGAAG

AAACCCGAGGAGTTCAGGCCGGAGAGGTTCCTCGAGGAGGAGGCCAAGGTGGAGGCCAAT

GGAAACGACTTTAGGTACCTTCCGTTCGGAGTTGGGAGGAGGAGTTGCCCTGGGATCATC

CTTGCCCTGCCCATTTTGGGGATTACCATCGGGCGTTTGGTGCAGAACTTCGAGCTGTTG

CCTCCTCCTGGACAGTCAAAGCTTGATACTAGTGAGAAAGGTGGACAGTTCAGCTTGCAT

ATCTTGAAGCATTCCACTATTGTTGCTAAGCCAAGGTCGGTTTAA

**3-KETOACYL-COA THIOLASE 2 (Β-KETOTHIOLASE)**

**AFSQ01008401**

ATGGACAAGGCGATCAATCGGCAGAAAGTTCTGCTAGCTCACCTGAAACCCAGCACCCGG

ACTCAATCCCATCATGAATCGCCCTTCCTCTCCGCTTCAGCTTGCGCTGGTGGAGCTGCA

TCGTTTGGGGATGACATCGTGATTGTGGCTGCGTATCGAACTGCCTTGTGCAAGTCCAGG

CGTGGTGGGTTCAAGGATACGCTTCCTGATGATTTACTCGCCCCAGTTCTGAAGGCAGTG

GTGGAGAAAACGAATGTGAATCCAAGTGAAGTAGGAGATATAGTGGTGGGTACGGTTCTG

GCTCCTGGCTCTCAGAGAGCAACTGAGTGCAGAATGGCAGCTTTCTATGCTGGTTTCCCT

GAAACTGTGCCTGTTAGAACAGTCAACAGGCAGTGGTCTTCTGGGCTGCAGGCGGTTGCT

GATGTTGCTGCTTCAATCAAAGCAGGATTTTATGACATTGGAATTGGAGCTGGTCTAGAG

TGCATGACAATTGACAATATCAGAGGAGTTCAGAAAGCTAATCCTAGGGTTGAGAGCTTT

GTTCAAGCCCGAGATTGCCTTCTTCCTATGGGCATTACTTCCGAAAACGTTGCCCAGCGG

TATGGGGTAACAAGACAAGAGCAGGATCAAGCTGCTGTCGACTCTCATAGGAAGGCTGCT

GCCGCAACAGCCGCTGGTAAATTTAAAGATGAGATTATCCCAGTTTCTACCAAGGTCATT

GACCCAAAAACTGGGAACGGAACTCCCGTGACTATCTCTGTTGATGACGGGATCCGACCG

AACACAAACCTTGAAGGCCTTGCCAAGTTGAAACCTGCATTCAAAAAGGATGGCTCTACA

ACTGCAGGTACTGCTAGCCAAGTGAGTGATGGTGCTGGAGCAGTCCTCCTAATGAAGAGA

AGCTTGGCCATCCAGAAGGGCCTTCCCATTCTTGGCGTGTTCAGGAGTTTTGCTGCTGTT

GGAGTGGATCCTGCTGTGATGGGGATAGGTCCGGCTGTTGCGATTCCAGCAGCAGTGAAA

GCTGCTGGATTGGAGCTTCAAGATATTGACCTCTTCGAAATTAACGAGGCATTTGCTTCC

CAATTTGTCTACTGTCGTAAGAAGCTAGAGCTTGACCCAGCGAAGGTTAACGTTAATGGC

GGAGCAATGGCTCTTGGACACCCTTTGGGCGCTACAGGTGCTCGCTGCGTCGCCACTCTT

TTGCACGAGATGAAGCGTCGTGGAAGGGATGCTCGTTTCGGAGTCATCTCAATGTGCATT

GGTACTGGAATGGGGGCGGCAGCTGTTTTCGAACGAGGAGACTGTGTCGACGAGCTCTAT

AATGCTCGATCTGTCGGAACCAGTGGCAGCCTCCTGTCGAACGGTGCTCGCTAG

**AFSQ01001818**

ATGGCGATTCTGTCGTCAGTAATAAGAAAATTGTTACCTTCAGTCTTCACAGCTTATTTC

CTTCTAACATTGGTTAGTACCGTAGCCTCCTTGCAGACAATGTGCCACCAAGACTCTGAG

TCATACCAAGCTGGTGATTTCCGCATAGAGTGCATGCAAGATTTCCTGGGCCGGCTAGCT

GTGGATTACAATCTCCCATCTTCATCCTGCGACTACAACACAACCTATGACTGCAAGGGC

AGCACGACTCCGCTGTACGGCTACATATGGGCGGCTGGTTGTGGCGATAGATTATCGGAT

TCGGTTTCGTGCCTGCGTACAGCTGCAGCAATGGTATACTATGGTTGCCCCAACAGCGTT

GGCGGCAGGGCGTGGATCACCAATGGTGACCACTGTCTCGTTCGGGGGAAGTCCGATCAA

AGTCGTAGATGGTTCCAGATTTGGTTGGCATTTATGATTGACGATTGCTTCTGCCTGATG

CACACCTATCTGAAATCAGCCTATAATTTCAGTCGACGTGCTCATCCTCTGTTGAAGAGT

TCAGGGGAAGGAAGCATAGTAGTCTTCATGTCATCTGCTGCCGTTGTGAGTTGTGACTGC

TTTGAACATTGGAGCCCTGCAAGTTATTTGCATTTCGTATTGCTGCATATAACTTTCTTT

TGGGTTCAAAAGCAACAACATCGTAACTGTCGATTTGAGAAAATTATGGGTTATAATGGT

TATATGTGTATGTATCTCTGTGCCATATCTGAATGGGAAGGAGAGTTGATGATGTGGCGT

GCATGCGTGGCCTGTTCGCGTTGGACTATTAAAGTTGATAATCCGATTTTTAGGAAACCG

CAAGGTCTTCACCGTTGTAGGGTGATAGACTTTTGCTCCTGTGCCCGTTGGTGGGTCCGG

CTGTTGCGATTCCAGCAGGCAGCAGCAGTGAAATCTGTTGGATTGGAGCTTCAAGATATT

GACCTCTTCGAAATTAATGAGCCATTTGCATCCCAGTATGTCTACTGCCGTAAGAAGCTA

GATCTCGACCCAGCAAAGGTTAATGTTAACGGTGGAGCAATGGCTCTCGACCACCCGTTG

GGCGCTACAGGTGCTCGCTGCGTCGCCACCCTTTTGCATGAGATGAAGTGTCGTGGAAAG

GGATGCTCGTTTCAGAGTCATCTCAATGTGCATAGAAAGAAAAAGATATTACAACGAATC

CCCAAGAAACCCCCATGTTCCTATGAATCAGGTACCGCCTCAACGTTTTCCAAACCACTC

TCTTCAACATGTTCCATCTCTAAGCTCCTGAATCATTCATTCTCTCGGTCTGGCTCTGAA

GAATAG

**AFSQ01019392**

ATGGACAAGGCGATCAATCGGCAGAAAGTTCTGCTAGCTCACCTTCAACCCAGCTCCCGG

ACTCAATCCCATCATGAATCACCCTTCCTCTCCGTGAGAAATGAGAATTCTTTCCTCCTA

AATCCTTGGATCCCGCTAAATTTTACACTGTCTAATTTATCTTCTGTTTCGCAACTGGGT

CGCTTAATTGCTTACCGAAGGCTTCAGCTTGCGCCGGTGGAGCTGCATCATTTGGCGACG

ACATCTGCGTATCGAACTGCCTTGTGCAAATCCAGGCGCGGTGGGTTCAAGGATACGCTT

CCTGATGATTTACTCGCCCCTGTTCTTAAGGCAGTGGTAGAGAAAACGAATGTGAATCCA

AGTGAAGTGGGAGATATAGTGGTGGGTACAGTTCTAGCTCCTGGCTCTCAGAGAGCAACT

GAGTGCAGAATGGCAGCTTTCTATGCTGGTTTCCCTGAAACTGTGCCTGTTAGAACAGTC

AACAGGCAGTGTTCTTCTGGGCTGCAGGCCGTTGCTGATGTTGCTGCTTCAATCAAAGCA

GGATTTTATGACATTGGAATTGGAGCTGGTCTAGAGTGCATGACAATTGACAATATCAAA

GGAGTTCAGAAAGCTAATCCCAGGGTTGAGAGCTTTGTTCAAGCCCGAGATTGCCTTCTT

CCTATGGGCATTACTTCCGAAAACATTGCCCAGCGGTATGGGGTAACAAGACAAGAGCAA

GATCAAGCTGCTGTCGACTCTCATAGGAAGGCTGCTGCCGCAACAGCCGCTGGTAAATTT

AAAGATGAGATTATCCCAGTTTCTACCAAGGTCATTGACCCAAAAACTGGGAATGAAACT

CCCGTGACTATCTCTGTTGATGACGGGATCCGACCAAACACAAACCTTGAAGGCCTTGCC

AAGCTGAAACCTGCATTCAAAAAGGATGGCTCTACAACTGCAGGTACTGCTAGCCAAGTG

AGTGATGGTGCTGGAGCAGTCCTCCTAATGAAGAGAAGCTTGGCCATCCAGAAGGGCCTT

CCCATTCTTGGTGTGCTCAGGAGTTTTGCTGCTGTTGGAGTGGATCCTGCTGTGATGGGG

ATAGGTCCGGCTGTTGCGATTCCAGCAGCAGTGAAAGCTGCTGGATTGGAACTTCAAGAT

ATTGACCTCTTCGAAATTAACGAGGCATTTGCATCCCAGTTCGTCTACTGCCGTAAGAAG

CTAGAGCTTGACCCAGCCAAGGTCAATGTTAACGGTGGAGCAATGGCTCTCGGCCACCCT

TTGGGCGCTACAGGTGCTCGCTGCGTCGCCACTCTTTTGCACGAGATGAAGCGCCGTGGA

AGAGACTCTCGTTTCGGAGTCATCTCAATGTGCATCGGCACAGGAATGGGGGCGGCAGCT

GTTTTCGAACGAGGAGACTGTGTCGACGAGCTCTGTAATGCCCGATCTGTCGGAACCAGT

GGCACCCTCCTATCAAAGGATGCTCGCTAG

**AFSQ01008403**

ATGGACAAGGCGATCAATCGGCAGAAAGTTCTGCTAGCTCACCTTCAACCCAGCTCCCGG

ACTCAATCCCATCATGAATCACCCTTCCTCTCCGTGAGAAATGAGAATTCTTTCCTCCTA

AATCCTTGGATCCCGCTAAATTTTACACTGTCTAATTTATCTTCTGTTTCGCAACTGGGT

CGCTTAATTGCTTACCGAAGGCTTCAGCTTGCGCCGGTGGAGCTGCATCATTTGGCGACG

ACATCTGCGTATCGAACTGCCTTGTGCAAATCCAGGCGCGGTGGGTTCAAGGATACGCTT

CCTGATGATTTACTCGCCCCTGTTCTTAAGGCAGTGGTAGAGAAAACGAATGTGAATCCA

AGTGAAGTGGGAGATATAGTGGTGGGTACAGTTCTAGCTCCTGGCTCTCAGAGAGCAACT

GAGTGCAGAATGGCAGCTTTCTATGCTGGTTTCCCTGAAACTGTGCCTGTTAGAACAGTC

AACAGGCAGTGTTCTTCTGGGCTGCAGGCCGTTGCTGATGTTGCTGCTTCAATCAAAGCA

GGATTTTATGACATTGGAATTGGAGCTGGTCTAGAGTGCATGACAATTGACAATATCAGA

GGAGTTCAGAAAGCTAATCCCAGGGTTGAGAGCTTTGTTCAAGCCCGAGATTGCCTTCTT

CCTATGGGCATTACTTCCGAAAACGTTGCCCAGCGGTATGGG

**BENZOATE/SALICYLATE CARBOXYMETHYLTRANSFERASE**

**AFSQ01004321**

ATGAAGGTAGATGAGATGTTACGTATGAATAGCGGAACTGGCGAGACAAGCTACGCTACC

AACTCCTTCCTTCAGAAAAAGGCCATATCAATGACTCGGCAAATAACAGAGGAGGCGATT

ACGGGACTAGTACTCGGCACCTCAAGCTTCCCGAAAGGAAGTTTGTCCATTGCGGATTTG

GGATGTGCTTCAGGACCCAACACTTTCTTCACTGTGTCGGAGCTTGTTAAGTCTGTCGTC

AGGATTTCGAGAAACCTAGGCCGAGAACCTCCTAAAGAGTTTCAGGTTTTCCTGAACGAC

CTCCCCGGAAACGACTTTAATAGCATCTTCACCTCTGTGCCAAAATTCAAGAAACAAATG

AAGGAAGAACTAACGGTGGAGGCTGGTGAATTTTCTATTTTCGTCAACGGAGTCCCTGGT

TCTTTCTACGGGAGATTGTTCCCGACGAAAAGTCTTCACTTTGTCTTCTCTTCCTACAGT

CTGCATTGGCTTTCTCAGGTTCCTGATGGACTTGTTGAGCTGAACAAAGGGAATGTTTCA

TATGGCATCAGCAGCTCTCGTGGCGTGTTTGATTTGTACTCGGGGCAATTTAGGAAGGAT

TTCGCCGAGTTCTTGAGGTGTCGAGCTTTGGAGTTGGTCTCTGGAGGGAAAACGGTGTTG

ACATTTCCTGGCAGGAGGAATGACGACTGTTCGAGGTGTTTGTACATCTTGGATCCCTTG

TGCATTGCTCTTAACGAAATGGCATCTGAGGGTTTGATAGACACAGAAAAGTTGGCTTCG

TTCAATATACCAGCTTATTTGGCATCTCCGATGGAAGTTGAAGCCGAAGTTCAGAAACAG

AGCTCCTTCACTATAGATTGCTTGGAGGACAAGGAAATGAGCTGGGATCCTTACGAAGGT

GAAGCAAACCTTCCAGAATCCTTAAAAGACTCTGGATCTGACATTGCAAAATGTATGAGG

GCAGTGGTAGAGCCATTGTTGGCCTGCCATTTGGATCTCCGGATGGAAATCATCGACGAA

GCATTCCAACGGTATGGCATAATAGTCTCGGAGCGGATCACCTCCGGCTATAAATTTACT

TACGCCAATCTAACCATCTCACTCACTAAGCTCTAA

**AFSQ01007680**

ATGAAGATCGATCAGTTGCTACACATGAATGGCGGAAAGGGAGAGACGAGCTACGCTGCA

AACTCCATGCTTCAGCAAAAAGCCATAACAATGGCTAGTCAAATTACAAAGGAAGCAATC

ACAAGACTTATAAAGTTAACCAATCCGACAAACCGGGTAACAATCGCTGATTTGGGATGT

GCTTCAGGGCCCAACACTTTCTTAGCCGTGTCAGAAATTGTTGAATCCTTTCTCAGGATT

TCCGGAGAGCAAGGGCGAGAATCTCCTGAAGAGTTTCAGGTTTTTCTGAACGATCTCCCT

GGAAACGATTTCAACAGCATCTTCACCTCCCTGCCAAAATTCAAACAAAAATTCAAGAAA

GATTTGATTTTTTTGAATGGAGTCCCCGGTTCTTTTCACGCAAGGTTGTTCCCGGCAAAG

ACTCTGCACTTTGTTCACTCTTCCTATAGCCTGCAATGGCTTTCTCAGGTTCCTGAAGGA

CTTGAGGAAAACAAAGGGAATGTTTGCATAGGTAGTAGTAGCCCGCCGAGTGTGTTCAAA

GCATACTATGATCAGTTTCGGAACGACTTTTCGAGTTTCCTAAAGTGTCGAGCTCTGGAA

ATTGTGGATGGAGGTCAAATGGTTTTGACGTTACTGGGTAGGAAGAGTGAAGACAGTTGC

ACAAGAAACACTTACTTCATCTGGGAGCTCTTGTCCATTGTCCTCAACCAAATGGCCGTT

GAGGGATTAATAGACCTGGAGAGACTGAACTCGTTCAATATCCCAAATTACTTCCCATCA

ATAACAGAAATCGAAGGTGAAACCCATAAACAAGGCTGCTTCACCATAGACCGCACAATG

GTTACGGAAGCGAGCTGGAATCCCTACGAAGGGGAGGAGGAATCGGACCTCTCAGAAGCC

ATGAAAGACGGCGGCTACAACGTGGCCAAGTACATCAGGGCCGTGGTCGAACCACTCTTG

GTTCGGCAGTTTGGTTCCACGAGGGAAGTCATCGACGAGGTTTTCGACAGATATTGCGTC

ATTGTGTCCAAATGGATGGTTATGATAAAGAAGAGAATGGTGTCCCTCCGCCACTCGGAG

GCTATGGCTCTTTTCAAGGCTATAGAATTTGCGAAAGATATAGGATGTAGAATGCTTATC

AAGTCAGATTGTTTCACTTTTATCAGAGTTTTCATGGGGCGGTTCATAAGCAAAGAGAGA

CTGGACTCGTTCAATGTTCCACTCTACATGCCATCTCCGGTGGAGATCGATGTCGAAGTC

AACAAGCAAGGGGAAGCGAATGTTGCCGAAGAATCCCATAAGGATGGTGGCTACAATGTG

GCCAAGTTGATGAGGGCTGTGGCAGAGCCATTGTTGGTCAGCCATTTCGGTTCCGACGAG

CAACTCATCGATGAGGTCTTCCGGAGGTATAGAGTGATTGTATCAGAGTGGATGCGGACT

GAGGAGTCTAATTTTGTGAATGTGACTGTCTCACCCACTAAGCAGTACAACTGA

**AFSQ01026389**

ATGACAATAATGGAGGTGGTCGATGTGCTTCACATGAATGGCGGTATGGGGCAAAACAGCTATGCCATGAACTCTTTGCTTCAGCAAAAGGTTATATCGATGACTAAGCCGATAGCAGAGGAGGCAATCACGCAACTCTACGTCTCCACCTTCCCGACGGCGAGGATAGCAATCGCGGATTTGGGTTGCTCGTCAGGGCCCAACGCTCTTTTCACCGTCACACTGCTGATCAAAACAGTGGAGGCTCTTTCCAAGAAGTTGTCTCGTAAGTCACCTGAGTTCCAGGTGTTCCTCAACGACCTCCCTGGAAATGACTTCAACAACATCTTCAGGTCAATCAACAGGAAATCAAACTGCGGCTCGCCGTTCATCAACGGGGTTCCGGGGACATTCTACGGCAGGCTTTTCCCACTCAACAGCATTCACTTTGTTCATTCTTCCTACAGCCTCCAGTGGTTGTCTCAGGTTCCGAAAGGGGTCCAGGAGGAGAACATCGGCAACATTTATATGGCTAGTAGCAGCCCTCCGAGCGTGCTGGATGCTTATTACGGCCAGTTCCGGAGCGATTTCTCGCTATTTCTCCGATGCCGAGCTCAGGAGGTGGTGACCGGTGGAAGGATGGTTTTGACGTTCCTCGGGAGAAGAAGCGATGACCCTTCCAGTAAGGAGTGTTGCTTTATTTGGGAGCTCATGTCCATCGCCCTCAACCAAATGGCCTCCGAGCTCGAGAGTTATGGATCCCATAATGATGAGAAGTCTGACATTTTTAAGGGCTTGATCGACCACGACAAGCTGGACAAGTTCAACATCCCGCAGTACACGCCGTCGCCGGCGGAAGTGGAGGCTGAAGTGAAGAAGGAGGGATCCTTCGTCATCGACCGCCTTGCCGTTTCGAAAGTGAGGTGGGATGCTTACGACGACGGCAACGAAGATGGTGCCCGGAAGGACGGTGGGTACACGGTGGCAGCTTGCATGAGGGCGGTGGCGGAGCCGCTGCTCGTCAGCCAGTTTGGTGGGGGGGAAATCATCGACGAAGTTTTCAGGAGGTACAGAGTCATTGTCTCTGAGCGGATGGCCAATGAGAAGACTGAATTTGTTAACGTCACTGTCTCTCTTATCAAGGATGCTGAATTTCATTGCGAGTGA

**BENZYL ALCOHOL O-BENZOYLTRANSFERASE**

**AFSQ01027011**

ATGGCCACATCACTAGTCTTCAACGTCGAGAGAAGCGAACCGGAGCTGGTCCGACCAGCG

AAACCGACCCCCTACGAGTTCAAACCACTATCCGACATCGACGACCAGGAGGGCCTCCGG

TTCCACATTCCGGTGATCCAGTTCTACAAGCACCAACCTACCATGGCCGGAAAGGACCCC

GTAGCGGTCATTCGCAACGCCCTGGCCGAGACACTAGTGCCTTACTACCCGTTTGCGGGA

CGACTCAGGGAATCGGGACAGAACAAGAAGCTTGTGGTTGAGTGTACCGGGGAAGGTGTT

CTGTTTATTGAGGCTGACGCCGACGTACGGATGGAGGACTTCGGCGAGTTTCTTCAGCCG

CCGTTCCCTGGGTTTGAAGAGTTGCTTTTTGATGTCCCTGGGTCTAGCGCCGTGCTCCAC

TCGCCGTTGTTGCTTATTCAGGTCACGCGTCTCCGATGCGGCGGATTTATCTTCGCTCTC

CGTCTCCACCACTGCATGAGCGACGCTCCCGGTCTGGTCCAGTTCATTTCCGCCATTGGC

GAGATAGCCCGTGGCGCGAAGTCTCCCTCCGTCCCGCCGGTTTGGGAGCGCCATGTGCTC

AATGCGCGGTCACCTCCGCGGGTGACTTGCACGCACCGCGAGTATGAAGCGGTACCAGAC

ACAAAGGGAACACTCATCCCGCTAGATGACATGGAGCACCGTTCCTTCTTCTTCGGCCGG

AAGGAGATGACGGCGCTCCGAGCCCACGCGCCGCCTCACCTCCGCGAGAGATGCTCCAAA

TTCGAGATCCTGACCGCATGCCTCTGGAAGTGCCGCACCGCCGCGCTGCGACCCGACCCG

GAGGAGGAGATGCGGATCATCTGCATCGTCAACGCGCGCGCCAAATTCGACCCGCCGCTC

CCCAAGGGATACTACGGCAACGCGTTCGCCTTCCCCGCGGCAGTGGCACGTGCGGACGAT

TTGTGCAGGAACCCGCTCGGGTACGCGCTGGATCTGGTGAGGAAGGCCAAATCGGACGTG

AACGAGGAGTACATGAGGTCCGTGGCGGATATGATGGCGACGAACGGGAGGAGGCACTTC

ACCGTGGTGAGATCTTACCTGGTGTCCGACGTCACCCGGGCCGGATTTGATGTGGTGGAC

TTCGGGTGGGGGAGGGCGGCTTACGCCGGACCGGCGAAGGGTGGCGTCGGCGCAATTCCC

GGAGTGGCGAGCTTTTACATTCCCTTTACCAACAAGGAAGGGGAGAAGGGAATTGTGTTG

CCGATCTGTCTGCCGGCGGAGGCAATGAAGATTTTTGTAAAGGAGCTAGAGAGGATGTTG

AAGGAGGCGCCGATCGCCGGTACCGGAGCGAAGGTTAACAGATCCACGTTAATCTTATCG

GCCTTGTGA

**AFSQ01022016**

ATGAATTGTACTCATCAAGATAGTTTTATTTATTCCCGGCAGGTCACACGCCTCCGATGC

GGCGGCTTCATCTTCGCCCTCCGCCTCCACCATTGTATGAGCGACGCTCCGGGTCTGGTC

CAGTTCATTTCCGCCATCGGTGAGATAGCCCGTGGCGCGACCTCCCCTTCGATCCCGCCA

GTGTGGGAGCGCCACGTTCTCAACGCGCGGTCTCCCCCGCTGGTGACATGCACGCACCGC

GAGTACGAAGAAGTGCCCGACACGAAGGGAACCCTCATCCCGCTAGATGACATGGAGCAC

CGTTCCTTCTTCTTCGGCCGGAAGGAGATGACGGCGCTCCGAGCCCACGCACCGCCGCAC

CTCCGCGGGAAATGCTCCCAGTTCGAGATCCTAACCGCCTGCCTCTGGAAGTGCCGCACC

GCGGCGCTGCGACCCGACCCGGAGGAGGAGATGCGGATCATCTGCATCGTCAACGCTCGC

GCCAAATTCGACCCGCCGCTTCCCAGGGGGTACTATGGCAACGCGTTCGCCTTCCCCGCC

GCGGTGGCACGTGCGGACGATCTGTGCAGGAACCCGCTCGGGTACGCGCTGGATCTAGTG

AGGAAGGCCAAATCGGACGTGAACGAGGAGTACATGAGGTCGGTGGCGGATATGATGGCG

ACAAACGGGAGGAGGCACTTCACGGTGGTGAGATCTTACCTGGTCTCCGACGTCACCCGC

GCCGGGTTTGACGTGGTAGACTTCGGGTGGGGGAAGGCGGCGTACGCCGGACCGGCGAAG

GGTGGCGTCGGCGCAATTCCCGGAGTGGCGAGCTTTTACATTCCCTTTACCAACAAGGAA

GGGGAGAAGGGAATTGTGTTGCCGATCTGTCTGCCAGCGGAGGCGATGGAGATTTTTGTG

AAGGAGCTGGAGAGGATGTTGAAGGAGGCTCCGGCGGCCGGTACCGGAGATAAGGTTAAC

AGATCGACGTTTATCTTATCGGCGTTGTGA

**4-COUMARATE-CoA LIGASE**

**AFSQ01015758**

ATGGAGTCCAACCAGCAGCAAACTCCGGCGACGTCGGCCGGTGAGGAATTCATTTTCCGG

TCGAAATTGCCGGACATCGACATCCCCAACCACCTCCCCCTCCACTCCTACATCTTCCAG

AACATCTCCAACCACGCCACCCGCCCTTGCCTCATCAATTCCGTCACGGGAGAAGTCTAC

ACCTACGCCGACGTCCACCTCACCGCCAGACGAGTCGCTTACGGGCTGGACAAGCTGGGG

ATCCGTCAGGGAGATGTGATTATGCTCTTGCTCCCGAACTGCCCCCAATTCGTCCTCGCC

TTCCTCGGGGCTTCCTTCCGCGGAGCAATCGCCACCGCCGCCAACCCGTTCTTCACCCCC

GCCGAGGTCGCCAAGCAGGCCAAGGCCTCCGGCGCGAAATTGGTCATCACTCAGGCCGCC

TTCGCTGATAAACTCAAGGATCTGGCTGCTGACGACGCGATTAAGGTGATGTGCGTCGAT

TCACCTCCCGACTGGTGCTTGCATTTCTCCCAATTGAGTGAATCCGATGACTCAGCGGTG

GAGGTAGAGATCAGCTCCGATGACGTGGTGGCGCTGCCGTACTCATCGGGAACCACCGGG

TTGCCAAAGGGAGTGATGCTGACTCACAAGGGGCTGGTGACGAGCGTGGCGCAGCAGGTG

GACGGCGAGAATCCGAATCTGTACTTCAACTCGGAGGATGTGATACTGTGCGTTCTGCCG

ATGTTCCACATCTACGCGCTGAACTCGATCATGCTGTGCGGGTTGCGGGTCGGGGCGGCG

ATCCTGATCATGCCGAAGTTCGAGATCGGACCTCTTCTGGAGCTGATTCAGAGGTACCGT

GTCACCATAGCGCCGATGGTTCCTCCGGTGGTGCTGGCGATTGCGAAGTCGCCGGACACG

GAGAAGTACGACCTTTCGTCGATTCGGATGTTGAAATCTGGTGCGGCGCCGTTGGGGAAG

GAACTTGAAGACGCCGTGGGAAACAAGTTCCCGAATGCGCGACTCGGCCAGATTATTGCT

AGGGTGGTGGCGCGGCGGGCCCTCCGTCCATCCACCATCGGCGACTTCTAA

**AFSQ01015369**

ATGACGATCGATCTTGTTCTGAAGCCGTCTTGTAGGTGCGGATCCACCTCGAATTTGTAT

GGAAGCAATTGCAAGCACATGACTTTGTGCTTGGACTGCGGCAAAAAGATGGCGGAAACA

GGCGCCAAATGCTACGACTGTGGCGCCCCTGTCACTCGATTGATTCGAGAATATAACGTT

CGGGCTACTCCTAGCAGTGACAAGAACTATTTCATTGGTAGGTTCATGGGTGGTCTGCCA

AGTTTTTCGAAGAAGAAAAGTGCTGAGAATAAATGGTCTATTCATAAAGAAGGGCTGCAA

GGGCGCATGCTGTCTGAAAATTTGAGGGAAAGGTACAAGAATAAACCCTGGCTGTTGGAG

GATGAAACTGGCCAGCATCGGTTTCATGGCCAAATGGAAGGCTCACAGTCTGTAACCTAT

TATCTATTAATGGTACAAGGAAAAGAGTTTGCTGCTATTCCTGCTGGTTCCTGGTACAAC

TTCAACAAGGTTGCACAATATAAGCAACTGACGCTGGAGGAAGCGGAAGAGAAGATGAAG

AACAGGAGAAAGACGGCAGAGGGATATGAAAGATGGATGATGAAAGCAGCAGCTGATGGA

CCTGCTGCATTTGGTGTTAAGGATAGTCTTGATGACGCAGATGCTGGTACAGGTGGTACC

AGGGGACGCAAAAAAGCATCGGGTGACAATGACGATGGCAATGTATCTGACAAGGGAGAG

GAAGATGAGGATGATGAAGCTTCTCGGAAGAGCAGACTTGGACTAGCCAAAAGAGGTGGA

GATGATGATGAAGAAGGAAAGAGAGGAGGTGACCATGATGATGATGATGATATTGAGAAG

GGTGATGATTGGGAGCATGAAGAAATATTTACTGATGATGACGAAGCAGTTGGAAGTGAT

CATGAGTTACCGGAAGAGTTGGCTCCTGCTCCTCCAGAAATAAAGCAGGATGATGAAGAC

GAGGATGAAGAGAATGAGGAAGGAAAGTTGAGTTCGTCTGGGAAAGAGTTGAAGAAGTTA

CTCGGGAAAGCTGCTGGGCTTAGTGATTCTGATGGTGAAGATGACGACGATGATGATGCG

GAGGATGAGATTTCTCCTGTACTGGCTCCCAAGAAAAAAGAGGCTGTTAAGGAGGAGCCA

AGTGACAATAATACTCCATCAAAAACAACAGCTGCTGGTTCGACAAAGGGAAATGCAACA

ACTTCAAAGTCAGGAAAGGGAAAGAGAAAAGCCGATGATTTCAAAGCCTCTAATTCTGCA

ACTCCCAAGAAGGCTAAGGCAGAAGAATCAAATTTAAAGAAGGTGAAGACGGAAGTTGAA

TCGAAACCTAGTGTAAAAGAAGAAGTCACACCTAATAGTAAGGGTAATTCGTCCAGCAAA

GTAGCACCTCCACCAGCAAAAGCTAGTTCAGCGCCAGCTTCTGGCCCTGTCACTGAAGAG

GAGATAAAGGCTGTTCTATTGGAGGGTGGGCCTATCACTACACAGGAACTTGTTGGCAGG

TTTAAGTCACGTCTAAAATGCTCTGAGGATAAGAGAGCATTTGCAGATGTATTGAAGAAA

ATATCAAAGATTCAGAAGAATGCTGGCGGCGGAGCCAGCTATGTTGTGTTGAGAGGCAAA

TAA

**AFSQ01001585**

ATGGAATCCAACCAGATTCAGCTGAACCCTGCGGCGCCGGAAAAGCAGGAGGAATTCATT

TTCCGTTCGAAATTGCCGGACATCGACATTCCCAACCACCTATCCCTCCATTCCTACATC

TTCCAGAACATCTCCAACCATGCATCCCGCCCCTGCCTGATCAATTCCATCACCGGAGAC

GTCTACACCTACGCCGATGTCCACCTGACCTCCCGCCGTGTCGCCGCCGGACTCGACAAG

CTCAGCGTCCGTCAGGGTGATGTGATTATGCTCCTCCTTCCTAACTGCCCCCAATTCGTG

CTCGCCTTCCTCGGCGCTTCTTTCAGGGGAGCCATTGCCACCGCCGCCAACCCTTTCTTC

ACCCCCGCCGAGATCTCCAAGCAGGCCAAGGCCTCCGGGGCTAAATTGGTCATTACTCAG

TCCGCCTTCGCCGAAAAAGTCAAGGATCTCGCTGATTCGATCAAGGTCGTGTGTGTGGAT

TCAACTCCGTTTGATTGGTGCTTGCATTTCTCTGAATTGGCTGATTCCGACGAGGCGGCG

GCTCCGGAGGTAGAGATCAACCCCGACGACGTTGTAGCTCTGCCTTACTCGTCCGGGACC

ACCGGATTACCGAAAGGAGTGATGCTGACGCACAAGGGGCTGGTGACCAGCGTGGCGCAG

CAGGTTGATGGAGAGAATCCGAATCTGTATTTCCACTCGGAGGACGTGATCCTATGCGTG

CTGCCAATGTTCCACATATACGCACTGAACTCGATAATGCTCTGCGGTCTTCGGGTCGGG

GCGGCGATCCTAATCATGCCCAAGTTCGACATCGGATCCCTTCTAGAGCTGATCCAAAGG

TACAGGATCACGATAGGGCCGATGGTGCCGCCGGTGGTGCTGTCGATCGCGAAGTCAACG

GAGACGGAGAAGTACGACTTGTCGTCGATTAGGATGTTGAAGTCCGGTGCGGCGCCGTTG

GGGAAGGAGCTGGAGGATGCTGTCAGAAACAAATTCCCAAATGCCAGACTTGGCCAGAAA

CGTAGAGCCGACGACGGTGATCATGTGGGTGCGAAAAATTTCGTCAACGGAGGTTGTAGT

CACCGGGAAATTGGTCGGGAAATTTTTCAGTCACTAGAGACGGGATACGGAATGACAGAG

GCCGGACCAGTGCTGTCAATGTGCTTGGCATTCGCCAAGGAACCATTCGAGATCAAAGCA

GGTTCGTGTGGCACCGTTGTCAGGAATGCCGAGATGAAGATCGTCGACCCTGACACTGGA

TCCTCCCTTCCCAGAAACCAACCTGGAGAGATTTGTATCCGAGGAGACCAAATCATGAAG

GATGATCAATACTTTGTTCTCCGAAAACTTTCAGGGTACCTGAATGACCCTGAATCCACA

TCAGCAACCATTGACAAACAAGGGTGGCTACACACAGGGGACATTGGCTACATTGATGAC

GACGATGAGCTCTTCATTGTCGATAGGTTGAAGGAGATCATTAAGTACAAAGGGTTCCAG

GTTGCTCCTGCTGAACTTGAAGCCTTACTTGTTGCACACCCTCAGATCTCTGATGCAGCT

GTTGTCGGGATGAAAGATGAGTCAGCAGGGGAAATCCCAGTTGCATTTGTGGTAAAAGAA

GACAACTCCGAGCTCACAGAGGATGCAATCAAGCAGTACATCTCGAAACAGGTTGTGTTC

TACAAGAGAATAGGAAGAGTGTTCTTCAGGGATTCTATCCCAAAGGCACCCTCCGGCAAA

ATATTGAGGAAGAATTTGAGAGCAGAGTTTGCCAATGGTTTAAGAAACTGA

**AFSQ01020997**

ATGGAATCCAACCAGATTCAGCTGAACCATGCGACGCCGGAAAAGCAGGAGAAATTCATT

TTCCGATCAAAATTGCCAGACATCGACATTCCCAACCACCTACCCCTCCATTCTTACATC

TTCCAGAATATCTCCAACCACGCCTCCCGCCCCTGCCTTATCAATTCCCTCAACGGAGAC

ATCTACACCTACGCCGATGTCCACTTGACCTCCCGCCGTGTCGCCGCCGGACTCAATAAA

CTAGGCGTCCGTCAGGGTGATGTGATTATGCTCCTCCTTCCTAACTGCCCCCAGTTCGTG

CTCGCCTTCCTCGGCGCTTCTTTCAGGGGAGCCATTGCCACCGCCGCCAACCCTTTCTTC

ACCCCCGCCGAGATCTCCAAGCAGGCCAAGGCCTCCGGGGCTAAATTGGTCATTACTCAG

TCCGCCTTCGCCGAAAAAGTCAAGGATCTCGCTGATTCGATCAAGGTCGTGTGTGTGGAT

TCAACTCCGTTCGATTGGTGCTTGCATTTCTCCGAATTGGCCGAGGCAGACGAAGCGGAG

GCTCCGGAGGTGGAGATCAACCCGGACGACGTCGTAGCTCTGCCTTACTCGTCCGGGACC

ACCGGATTGCCGAAAGGAGTGATGCTGACGCACAAGGGGCTGGTGACGAGCGTGGCGCAG

CAGGTGGACGGAGAGAATCCGAATCTGTATTTCCACTCGGAGGATGTGATCCTCTGCGTG

CTGCCGATGTTCCACATATACGCGCTGAACTCGATAATGCTTTGCGGTCTCCGGGTCGGG

GCGGCGATCCTAATCATGCCCAAGTTCGACATCGGGTCCCTTCTAGAGCTGATCCAGAGG

TACAGGATCACGATAGGGCCGATGGTGCCGCCGGTGGTGCTGTCGATCGCGAAGTCAACG

GAGACGGAGAAGTACGACTTGTCGTCGATTAGGATGTTGAAGTCCGGCGCGGCGCCGTTG

GGGAAGGAGCTGGAGGATGCCGTCAGAAACAAATTCCCAAATGCCAGACTTGGCCAGATG

ACGAAGGGGCATCCCCGTAATTATGGTCAGTTCAAAGCCCAACAAAATCTTGTAGGTATC

TACCAGGCAACGCCGTTGAATATTGATGCTGAGGATGTCAATTACAAACCCAGAACGGAT

TGCCGCCAAATTTATCTGCATAGCGACAAAGACATTATTGGGGGTAGAACAGTAATTTTA

TCGTTTATGAGCTGGACTGAACTTGGTTGTAACATGACCGTATTGCCTGTAGTTAACTTG

GTCCTTATATCATGGGGAGATCTAAGGATTCAGATTGCTCGTGGGGGATACGGAATGACA

GAGGCAGGACCAGTGCTGTCAATGTGCTTGGCATTCGCCAAGGAACCATTCGAGATCAAA

GCAGGTTCGTGTGGCACTGTTGTCAGGAATGCTGAGATGAAGATCGTCGACCCTGATACT

GGATCCTCCCTTCCAAGAAACCAACCTGGAGAGATTTGCATCCGAGGCCACCAAATCATG

AAGGGGTACCTGAATGACCCTGAATCCACATCAGCAACCATTGACAAACAAGGGTGGCTA

CACACAGGGGACATTGGCTACATTGATGACGACGATGAGCTCTTCATTGTCGATAGGTTG

AAGGAGATCATTAAGTACAAAGGGTTCCAGGTTGCTCCTGCTGAACTTGAAGCCTTACTT

GTTGCACACCCTCAGATCTCTGATGCAGCTGTTGTCGGGATGAAAGATGAGTCAGCAGGG

GAAATCCCAGTTGCATTCGTGGTAAAAGCAGACAACTCTGAGCTCACAGAGGATGCAATC

AAGCAGTACATCTCGAAACAGGTTGTGTTCTACAAGAGAATAGGAAGAGTGTTCTTCAGG

GATTCTATCCCAAAGGCACCCTCCGGCAAAATATTGAGGAAGAATTTGAGAGCAGAGTTT

GCCAATGGTAATTCTACAGCAATCCAAAGTCGATTGCACCTCCGACGATCAACGATCATT

CCTCGCAAACTCTAA

**AFSQ01014596**

ATGGAGCCGCCGATCAACTCAGTGATTGACCCGAGATCGGGATTCTCCCAATCCAACTTG

GTTTTGTACAGCAAACGTAACCCAATCCCTCTCCCACCAAACCCTTCCCTCGACGTCACA

ACCTTCATCTCTTCTCAAGCTCACCACGGCAAGACCGCCTTCATCGACGCCGCCACCGGC

CGCCACCTCACTTTCCGAGACCTATGTCCGCCGCCAATCCCTCGCCACGCACCTCTCGGG

AACCATGGGAATCCGAAAGGGACACTAATCACCACCACCAATCCTCTCAACATACCTCGC

GAAATTGCCAAACAGATCTCCGATTCCAAGGCTTCGTTGGCCTTCACCGTCCCCGAATTA

GTCCCCAAACTCGCCGGCAAAAGGTCTGCACTACCTTCATACAAGTCAGACGGAGGGATC

ATCCACATGGATGTGAAGACCACAAACATATTGCAGGACGATAATTTGGTGGCGAATGTG

ACAAATTTCGGGCTATCACAGTCGGGTCCGCCTGATCCGGATCACCATACCGTGGCGTTA

AAAGGATGCTTCGGTTATCTCGATCCAAAATTCTTCAGGATGCTTCAGTTGACTGATAAA

TCCGACGTGTACACGTTGAGGAAATATGTTGAGATTGCTGAGAAACGCTTGAGGGTGAAT

GGAGTGGAGAGGCCTGCGATGTAG

**AFSQ01023277**

ATGGCCCACCAGATACACTCCAAGCACCCCGCCGCCGTCGACCCCAACAACGGCTTATGC

AAATCCACCTCCGTCTTCTACAGCAAGCGAGACCCCACCCCACTCCCGCCGGCCGACTCC

CTCGACGTCGCCACCTTCATCGCCGCCCATGCCCACAGCGGCAAAACCGCCTTCATCGAC

GCCGCCACGGGGACCCACCTCACCTATCTCCAGTTCTGGCGGGCCGTTGACTCGGTCTCC

GCCCGTCTCTCCGAATTGGGCCTCCGTAAGGGCCACGTGGTCCTCCTCCTTTCCCCGAAC

TCCATCCTCTTCCCTATAGTCACACTCTCCGTTATCTCCTTAGGCGCCGTCATCACGACC

GCTAACCCGCTCAACACTCCTAACGAGATCGCCAAACAGATTGTCGATTCCTGTCCTTTC

CTCGCCTTCACCACACATGAACTCGTCAATAAGCTCGACGCCGTTAACTTCCCCGTCATC

CTAATGGACGACGTCAATAGTGGTGGGTTTCACCACACGCCAAAAGTGGAAATAGTGACG

ACGTTAACGCAGATGATCAAGAAGCCGTTGGATCGTAATTATCGGACGGCGAGGGAGCGA

GTTAGTCAAAACGACGTCGCGACTCTGCTTTACTCGTCCGGGACGACGGGAGCGAGCAAG

GGTGTTTTGTCGTCACATGGGAGTCTAATTGCCGTGGTACAGCATGTAGTGAGACTACAC

CGTTTAGCTACTTTGGAACGCAAGAGACAAAATCCGTTAAAGTTCCTTTGTACCATACCG

ATGTTTCATATCTATGGTTTGACCATGTTCGCGATGGGGCTCCTGGCGGCAGGAACCACC

ATCGTGATATTGTCAAAGTACGATATTGAAGATATGTTATCGGCAATCGAAAAATTTGGT

GTGACGAATTTGCCTTTGGTGCCTCCTATTTTGGTCACGATGACCAAGGACGCTGACCGG

ATAAAGTCAAAGTTTGACCTGGAAGTGTTAGAGTGTGTGTTGTGCGGTGGGTCGCCGTTG

AGCAAGGAAGTGGCGGAGGGTTTCGCAGCTAAGTACCCGACGGTGAGGGTGAGTCAGGCT

TACGGGATGACGGAGTCGCCGTGGATCGCATCGATGGACACGCGGGAAGAGTGCCAACAA

CAGCCGGGAGCGGTGGGGAAACTACTACCGAACATGGAGGCGATGATCGTGGCTCCGGAG

AGTGGGGATGCTTTGGGAGTGAACAAGACGGGTGAGCTTTGGCTTAGAGGTCCTTACATA

ATGAAAGGCTACCTTAACAACCTGGAGGCAACATCGTCAACTATTGACCCAAAGGGATGG

TTAAAAACAGGGGACATCTGCTACATAGACGAACATGGCTTCCTCTTCGTGGTTGATCGG

CTCAAGGAGCTCATCAAATACAAAGGATATCAAGTTGCGCCTGCGGAGCTGGAGGCATTA

CTGCTGACGCATCCCCAAGTGGATGATGCGGCTGTGATTCCAATGCCACACAAGGAAGTT

GGGCAGTACCCGATGGCTTATGTGGTGAGGAAGCCTGGCACCAACTTATCCTCCGCCGAC

ATCATTGAATTCGTTGCCTCGGAGGTTGCTCCATACAAGAAAGTTCGGGAAGTGAAATTT

ATCGCAGCAATACCGAAAAATCCGTCAGGAAAAATCCTGAGGAAGGATTTGATCAAGCTC

GCACCCGCCGCGGCCGGCTCCTCCAGGCTCTGA

**AFSQ01012878**

ATGGCCGCCCCCGCTACACCTCACCGGAGCGACAATTCCGTCGATCCCTACACCGGATTC

TGCAGCACCACCAAAATCTTCCACAGCCTGAGGCCTCCTGCGCCCCTCCCTCCTATTGAC

CTGCCTCTCTCCATCGTACACTACGCCTCCGACCTCCTTTGCTCATCCCCCACCGTCTCC

GACCTATCCTCCGCAATTTTCGTCATCGATGCAGCTACCGGCGACGTCCTCACCTACGCT

TCCTTCCTCCGTCAAACCCTCTCCCTCTCCCTCGCCTTGAAAAGGCTCTACCCTTCTCTG

TTACCAAACGACGTCGCATTCATCGTCTGCCCATCTTCTGTCCACATACCCATCCTCTAC

TTCGCCCTCATGTCAATTGGCGTCACAATCTCGCCTTCCAACCCGCTGAGTTCCCACTCC

GAGCTGGCTCACCAAATCCAACTCACTCAACCTAAAATCGCCTTTGCTACTTCCCAAACA

GCCTCTAAGCTCCCTTCTCATCTTCTCCCTCTAGGCACCTTTCTAATTGACTCTCCTCAA

TTTCAATCTCTATTAACCGAGCTGGATAGTCCTGTTCCGGTTGCCCAATCCCAATCCGCC

GTTGTAAGGCAATCCGACACGGCTGCCATTTTGTACTCCTCGGGAACCACGGGGAAGGTG

AAGGGAGTGAGATTGAGCCATCGGAACGTAATCGCCCTAACTTCCGGGTTTTACCACAAC

AGGCGGGAGTACGAGCCGGGGCAACCCGAACCGCATCCGGTTGCGCTCATCACGGTGCCA

CTGTTCCATGTATTTGGGTTCTTTATGTTGGTGAAAGGGATTGCAATAGGCGAGACGCTG

GTTCTGATGCAGAGGTTCGATTTCGAAGGGATGCTTCGGGCTGTGGAGAAGTACAAGGTG

ACTTACATGCCGGTATCTCCGCCGCTCATCGTGGGGATGCTCAAGTCGGAGTTGACCAAT

AAGTATGATTTGAGCTCTCTCTTAGTTTTGGGCTGCGGCGGGGCTCCACTCGGCAAGGAT

GTTGCCGAAAAGTTCAAGGACAAATTCCCAAATGTGGATATCATCCAGGGATATGGATTG

ACGGAAACTGGGGGAGGAGGGACACGAATGATTGATGCTGAGGAGAGTAAAAATTATGGG

TCAGCTGGTAAGTTATCGGAGAATCTGGAAGCTAAAGTTGTGGATACTGAAACAGGGGAG

GCTCTGGGACCTGAGCAGAGAGGGGAGATATGGTTGAGAGGCCCAGTCATCATGCAAGGG

TATGTTGGGAATGACAAGGCAACTGCTGAAACTTTGGATCGGGAGGGATGGTTGAAGACC

GGGGATCTTGGTTACTTCAACTCTCAAGGCCACCTCTACATCGTTGACAGGCTCAAGGAG

TTGATCAAATACAAGGCTTATCAGGTTCCACCTGTAGAGTTGGAGCAGTTGCTTCAGTCA

AACCCTAAAATAGCTGATGCTGCTGTAATCCCGTATCCTGATGAAGATGCAGGGGAAATC

CCCATGGCATTTGTGGTGAGGAAACCAGGGTTCAATCTTACAGAATCTCAAGTCATGGAA

TTTGTCGCTAAGCAGGTAGCACCATACAAGAAAGTAAGGAAAGTGGCTTTCGTGAATTCC

ATTCCGAAATCACCTGCGGGGAAGATTTTGCGGCGGGAGCTTGTGAAGATTGCTGTCTCT

GCTCCTGCATCTAAGTTGTGA

**AFSQ01023785**

ATGGCGCCGCCGATCAACTCAGTGATTGACCCGAAATCCGGGTTCTCCAAATCCAACTCG

GTCTTCTACAGCAAACGTAACCCAATCCCTCTCCCACCAAACCCTTCCCTCGATGTCACA

ACCTTCATCTCTTCTCAAGCTCACCACGGCAAGACCGCCTTCATCGACGCCGCCACCGGC

CGCCGCCTCACTTTCGGGGACCTCTGGTCCGCCGTCGACTCCCTCGCCACGCATCTCTCG

GAAACCATGGGAATCCGAAAGGGACACGTGGTCCTCCTCCTATCCCCTAATTCCATCTCC

TTCCCAATCGTCTGTCTCGCCGTCATGTCCCTCGGCGCAGTAATCACCACCACCAATCCT

CTCAACACTCCTCGCGAAATCGCCAAACAGATCTCAGATTCCAAGGCTTCGTTGGCTTTC

ACCGTCCCCGAATTAGTTCCCAAACTCTCCGGTTCGAACCCTAAACTCCCGATCGTCCTA

ATCGATGGAGAGAATCACGACTACGGCGTTCAATCTAGAGCCAAACAGCCCCTGATCGTT

GCGAGATTGAGCGAGTTGTTGATTAAGAAGGCGGTTAATCGGAGTGGAGGAGGACGTGTC

AGGGAGCAAGTTCACCAGGAAGACACGGCGACTCTGCTCTACTCCTCGGGGACTACAGGG

GAAAGCAAAGGAGTCGTTTCCTCTCATCGGAATCTAATCGCGATGGTCCAGACGGTCGTC

GGACGGTTCGATCCCGCCGACGGAGATCAGATCTTCGTCTGCACGGTCCCGATGTTTCAC

ATCTACGGCCTCGCTGCGTTCGCCACTGGACTGTTGGCGTCCGGATCGACTATCGTCATC

CTGCCTAAGTTCGAGATGCACGAGATGCTGTCGGCGATCGAGCGCTTCGGAGCGACTTAC

CTGCCGTTGGTGCCGCCGATCCTGGTGGCGATGATCCACGGCAGCGATCAGATGCGGTCG

AGATACAATTTGGGATCGCTGAGGTCTGTTTTGTCAGGCGGAGCGCCGCTCAGTAAAGAA

GTGATCGAAGGGTTTTTGGAGAAGTATCCGACGGTTAGCATTCTTCAAGGGTACGGATTG

ACGGAATCGACGGGTATCGGTGCGTCCACGGATAGCGTGGAGGAGAGCCGGCGGTATGGG

ACGGCCGGATTGCTGTCGCCGAGCATGGAGGCGAAGATTGTCGAGCCGGAAAGCGGGAAA

GCATTGCCGGTGAATATGACCGGTGAGCTTTGGCTCCGTGGTCCGTCCATTATGAAAGGT

TACTTTAGCAATGCTGAGGCAACGTCATCGACTTTGGACTCGGAAGGGTGGTTAAGAACA

GGGGACCTTTGCTACATAGACGAGGACGGCTTTATTTTCGTGGTAGATAGGTTGAAGGAG

CTGATCAAGTACAAAGGATATCAGGTTCCTCCTGCGGAGCTGGAAGCTTTGTTGCTTACT

CATCCCGAAATCACAGATGCTGCCGTGATTCCGTTTCCTGACAAAGAGGTGGGACAATAC

CCAATGGCGTATATTGTGAGGAAATCACCAGGAAGTAGTCGATTATCAGAAAGAGATGTG

ATGGGGTTTATTGCAGAGCAGGTTGCACCGTACAAACGTGTAAGGAGAGTTTCGTTCATT

GATGCGGTGCCGAAGAACCCTTCTGGTAAAATTCTGAGGAAGGATCTTATCAAGTTTGCT

ACCTCAAAACTGTAA

**AFSQ01013215**

ATGGCCACGTGGACTAGCCACCAAATTCCGCCAGTCACCAATTTGCCAATTCACCACCAA

ACACAACTAAATTTTATAAGCTTTGAAAAAAAAAAGCCATTCTCTCTCCTCCTCAATCCC

CATTCTCTATCCATTCCCCTCTCATTACCGATGCTCCAATCAGAAACTCGTCATCAAGGA

CTCAAGTCCGTAACTAGTCATCACCGGCTCGGAGCTCCGACGATTTTCCTGTCCAAATTC

AGTGGGAATCTTGAGTTTTCATCAAGTTGTGGAGATGGCCGATGGGTTCAGCGGAAACAC

CGAAACAGAGTTTCCGCATTGGGTGTGAAGAAGATCGGCACGGGACGATTCTGTACTCGT

CGAGGACGACTAGGTTGA

**HYDROXYCINNAMOYL-COA:QUINATE/SHIKIMATE HYDROXYCINNAMOYL TRANSFERASE**

**AFSQ01015754**

ATGGAAATTAAGGTGAAAGAATCGACGCTGGTCCAACCTGCCGCTCCGACGCCGACGAGG

AGACTCTGGCTCTCCGACGCCGACCTCGTCGTCCTCAGGACTCACCTCCTGACCGTCTAC

TTCTATCGGCAGCCTATCACTAGGACCGCAGATTTCTTTGACCCTGCAAAGCTGAAGCAG

GGTTTGAACAAGGCCCTAGTGCCTTTTTACCCGATGGCCGGCCGTCTTGGGCTGGCCGAA

AATGATGGTCGGGTTGAGATCGACTGTAACTCTCAAGGAGTTCTGTTCGTGGTTGCGGAG

TCGACTTCGGTCGTTGACGATTTCGGGGACTTTGCTCCGTCGTTGGAGCTCCGGCAGCTT

ATCCCGGCTGTTGACTACTCTGCTGGGATCTCATCCTACCCTTTGTTGGTTGTTCAGGTA

CGTAGATCATTTACCGACCGTGTTTCTAGATACGCTGGAGACAGGCGGGAGTTTGGTGCT

GGTGTGGTTCGGTGGGGAGCCATGGCTGACGATCGGCCAGCGGTCTTGGCTGGGTTCGGG

TTGCAGACTTGCAACGTAACATTCTTTAACTGCGGCGGAGTATCACTCGGGGTTGGAATA

CAACACCAAGTCGCCGACGGCTTTTCAAGTCTCCACTTCATCAACACATGGTCCGATATG

TGTCGAGGCCTTCCCCTAAATCTCCCTCCGTCGATTGACCGGACCCACCTTCGTCCTCGA

GACCCACCAACGCCCACCTACCATCACATCGAATACCAGCCTTCACCAGCCATGAAAACT

GCTAACCTCACCAGTGGAGCCATTGTCACATGCCGAATCTTCAAGCTCACAAAGGACAAG

ATCAACACACTTAAAGAAGCACGAAACCCAAACGACGCCGTTAGGCACTACAGCTCCTAC

GAGGTCCTAGCCGACCACATCTGGCGGTGTGTCTCCAAAGCCAGGGGCCTCTCCGGAGAC

CAGGAAACGAAACTATACATCGCTACCGATGGACGTTCCCGTCTCCGGCCGCCCCTCCCA

CCTGGATACTTCGGCAACGTGATTTTCACGACTACCCCGGTGGTCACGGTGGGCGAGCTG

TTAAACCGGCCGAGTTGGTCAGTCGCGGGTCGGATCCGTGACGCTTTGGCCCGGATGGAT

GATGAGTATCTGAGATCGGCGTTGGATTACCTGCACGTGGAACCGGACCTGTCGTCGTTG

GAGAAAGGAGCCCATACTTTTAGGTGTCCGAACTTGGGGATAACTAGTTGGGCCCGACTT

CCGGTCCATGATGCGGATTTCGGATGGGGGAGGCCCGTTTTTATGGGCCCTGGGGCAGTT

CCGTTTGAGGGGCTATCGTTCTTGCTGCCGAGCCCAACTAAGGATGGGAGTATGTCGGTG

GTGATCTCGTTGCTCGCCGAGCACATGAAGGAATTCGAGAAGCTGATCAATGAAGTATGA

**AFSQ01014410**

ATGAAAATCTCGGTGAAAGAATCGACGATGGTCCAGCCGGCGGAGGAGACCCCCAACGTG

GGACTCTGGAACGCCAACGTTGACCTTGTGGTCCCCAGGTTTCACACCCCTAGCGTTTAC

TTCTACCGGCCGACTGGAGCGGCCGATTTCTTTGATCCGGCCGCTTTGAAGCTGGGCCTT

GCTAAGGCCCTGGTTCCATTTTATCCGATGGCCGGCCGGTTGAGGCGTGACGAAGACGGT

CGGATTGAGATAAATTGTAATGCGGAGGGTGTTCTGTTTGTGGTTGCTGAGACCACCTCT

GTGATCGATGATTTTGGGGACTTTGCTCCCACTTTGGAACTCCGGCAGCTTATTCCGGCT

GTGGATTACTCCGGCGGCATCTCTACCTATCCTCTCTTGGTTCTCCAGGTGACATACTTC

AAGTGCGGTGGAGTCTCACTGGGAGTCGGGATGCAACACCACGCCGCCGACGGCTTCTCC

GGCCTCCATTTCGTCAACACATGGTCCGACATGTCCAGAGGACTCGACCTCACCCTCCCT

CCATTCATCGACCGGACCCTCCTCCGTGCTCGGGACCCGCCCCGACCCGCTTTCCAACAC

ATAGAATACCAGCCCCCTCCCTCCTTGAAAAGCACCTCCTCCGCCACCGCTGCCTCCGAT

TCCGCTGCAGTGAAAGCCTCCGACGCCGGCGTCACGCCGCCAGCAGCTGTGTCGATATTC

AAGCTGACCCGGGACGAGCTCAACGCCCTGAAAGCCAAATCGAAAGAGGCAGGTAATACA

ATTAGCTACAGCTCCTATGAAATGCTCGCTGGCCACGTATGGCGGTGCGCCTGCAAAGCC

AGGGGACTCGCCGACGACCAGGACACTAAACTGTACATCGCCACCGACGGAAGGTCCCGC

CTCCGCCCTCCGCTCCCGGAGGGGTACTTCGGGAACGTAATATTCACGACTACCCCGGTC

GCTTCCGCTGGGGATCTGGCGACAAACCCGACGTGGTTCGCAGCGGGTCGGATCCACGAC

GCTTTGGGGCGGATGGACGATGAGTACTTGAGATCGGCGCTGGATTACCTGGAAGTGCAG

CCGGATCTTTCGGCGCTGGTGAGAGGGGCGCACACTTTCAAGTGTCCGAACTTGGGGATA

ACGAGTTGGGCTAGGCTTCCGATCCATGAAGCGGATTTCGGGTGGGGTAGGCCCATATTC

ATGGGCCCCGGTGGGATTCCGTTCGAAGGGTTGTCGTTTATACTGCCGAGCTCTAACAAG

GACGGGAGTATGTCTGTGGCGATATCGTTGCAGGTGGAGCATATGAAATTGTTTGAAAAG

TTCATTTATGAGATCTAA

**AFSQ01015743**

ATGAAAATCTCCGTCAAAGAATCGACGATGGTCCAGCCGGCGGAGGAGACCCCCAACGTG

GGACTCTGGAACGCCAACGTTGACCTTGTGGTCCCCAGGTTTCACACCCCAAGCGTTTAC

TTCTACCGGCCGACTGGAGCGGCCGATTTCTTCGATCCCGCCGCCATGAAGCGGGGCCTT

GCTAAGGCCCTGGTCCCTTTCTATCCGATGGCCGGCCGGTTGAGGCGCGACGAAGACGGT

CGGATTGAGATCAACTGTAATGCGGAAGGTGTTCTGTTTGTGGTCGCGGAGACTACCTCT

GTGATCGATGATTTCGGGGACTTTGCTCCCACTTTGGAGCTCCGGCAGCTTATTCCGGCT

GTGGATTACTCCGGCGGGATCTCTACTTATCCTCTCTTGGTTCTCCAGGTGACATACTTC

AAGTGCGGCGGAGTCTCACTGGGAGTCGGGATGCAACACCATGCCGCCGACGGCTTCTCC

GGCCTGCACTTCGTCAACACATGGTCCGACATGTCCAGAGGACTCGACCTCACCCTCCCA

CCATTCATCGACAGGACACTCCTCCGTGCTCGGGACCCGCCCCGACCCGCTTTCCAGCAC

ATCGAGTACCAGCCTCCTCCCTCCTTGAAAACCATCTCCTCCTCCGCCACCGCTGCATCC

GATTCCGCCGCAGTGAAAGCCTCCGACGCCGGCGTCACGCCGCCAGCAGCCGTGTCGATA

TTCAAGCTGACCCGGGACGAGCTCAACGCCCTGAAAGCTAAATCGAAAGAAGCAGGAAAC

ACCATCAGCTACAGCTCCTATGAAATGCTCGCCGGCCACGTCTGGCGGTGCGCCTGCAAA

GCAAGGGGACTCGCCGACGACCAAGACACAAAATTGTACATCGCCACCGACGGCAGGTCC

CGCCTCCGCCCTCCACTCCCCGAGGGATACTTCGGGAACGTGATATTCACGACGACCCCG

GTAGCTTCCGCCGGGGACCTGGCGAGCAACCCGACGTGGTTCGCGGCGGGTCGGATCCAC

GACGCTTTGGGGCGGATGGACGATGAGTACTTGAGATCGGCGCTGGATTACCTGGAAGTG

CAGCCGGATCTTTCGGCGCTGGTGAGAGGGGCCCATACTTTTAAGTGTCCGAACTTGGGG

ATAACGAGTTGGGCCCGGCTGCCGATCCATGAGGCGGATTTCGGATGGGGTAGGCCCATT

TTCATGGGCCCCGGTGGGATTCCGTTCGAAGGGTTGTCGTTTATACTTCCGAGCTCTAAC

AAGGACGGGAGTATGTCTGTGGCGATATCGTTGCAGGTGGAGCATATGAAATTGTTTGAA

AAGTTCATTTATGAGATCTAA

**AFSQ01006651**

ATGAAGGTAACGGTGAAGGAGACGACCATCGTCCGGCCGCAGTCGGAAACCCCACGGCGC

AGTATATGGCTGTCCAACATGGATCTCCTACACGGCCGGATCCACCTCCCTACCATCTAC

CTCTACAAGCCCGACGGCTCCGCTGACTTCTTCGACTCCAAATCTCTCAAGGATGCTCTC

TCTAAGCTCCTAGTCACCTTTTACCCTGCCGCCGGGAGGCTCTCCAAGGACGAGAAAGGC

AGGATCGAGATCAACTGCAATGGCGAAGGCGTCCTGTTCCGTGAAGCTGAGACGGATCTC

AAAATGTCTGACCTTGGAGAGTTCATGCCTGGGACTGACCTTCTTCAGTTCGTGCCTAGA

GTTGACTACTCTGCTGGCATTTCTTCTTACCCTCTTTTCATCAGTCAGGTAACGAGATTC

TCATGCGGAGGAGTATGCGTGGGAACCGGCTGGCACCACACTGTGGCTGATGGGGAAGGC

GCGTTGAGCTTCATCAACGCCTGGTCCGACGTGGCACGTGGGAAACCGGTGGCGGTCCAA

CCGTTCCTTGACCGAACCCTTCTCAAAGCCCGATCCCCACCCAGCCCAAAGTTTGACCAC

CTGGAGTACGCCCCACCGCCGTCAATGATAACCGGGGAAGAGAAAAAGGAAGATGAATCG

ACATCCATGGCCATCTTTCAGATCACACTCGACCAAATCAACAGCCTGAAAGAGCGAGTC

AACAGAGACAACGATTCCAAGACCAAGTACAGCACCTACCAGATCTTGACCTCCCACATT

TGGCGCGCCGTCACCAAGGCCCGCGACCTCCCCGCCGAACAGCCGACTAAGCTCCACATC

TCCACCGACGGAAGGACTCGCCTCAATCCTCCGCTTGCCAAAGGATACCTCGGCAATTGC

GTCTTCCACGCCACGCCCATCGCCACATCGGGAGATCTGTTGTCTGAGCCGCTGATCCGG

ACCGTGGACCGGATCCACGACGCGATCAAGAGGATGGACGACGAGTACCTGAGATCCGCC

ATCGATTATTTGGAAGATCCGAACGACTCGTCTGCGATAATGCAGGTTCCGGGGACTTGC

AGGACGCCGAATCTGAAGATTATTAGCTGGATGCGGCTGCCATTTGAGTGCGCTGATTTC

GGATGGGGGAAGCCGATTTTTAACAGGCCGGCGAATCTTTTCGAAGGGAAAGGGAATATC

TTGCCCAAGTACAGCGTCGACGGGAATCTGTCGCTGGCGCTCTGCTTGGAGACTTACGCC

ATGGAGAATTTCAGGAAGACCTTTTACGATGTCACTGTGTGA

**CAFFEOYL SHIKIMATE ESTERASE**

**AFSQ01024190**

ATGACGAACGGGCTGCGTGCGGAATGGACTTGTTTCTTGTTCGTCCTAGCTAGGACGGGA

GACCTGGAGAGTCAAGAGTGTGTGGAATGGAATGGAAGCGTGGATATTGGGATAAGGACG

ACGACCAGAATCAAGGTCCACGAGCTGAGGCAGAAGAACAAGTCGGACCTTTTGAACCAG

TTGAAGGATCTCAAGGCTGAGCTCGCCCTCCTCCGCGTCGCTAAGGTCACTGGCGGCGCT

CCTAACAAGCTCTCCAAGATTAAGGTGGTGCGCTTGTCCATTGCCCAAGTCTTGACTGTG

ATTTCACAGAAGCAGAAAGCTGCTTTACGAGAAGTCTACAAGCACAAGAAGCTCTTGCCT

CTTGATCTTCGTCCTAAGAAGACTAGAGCCATTAGAAGAAGGCTTACAAAGCACCAGATT

TCGCCGCCTCGGCGTCGCGGAAATCGATTCCTCATCGATTTCGCGCGATTCCTTAGTCAT

TCCTCGGTCGGCTGCCTTATTTCATCACCTTGTCCGAGGCGAGGCGTTTTGCCGCAATCA

TTGAAGACGGAGAGGGAGCAGAAGAGAGAAATGTACTTCCCAATGAGAAAGTATGCAATC

AAGGTTTAA

**AFSQ01011162**

ATGAGTCTCTGTTTTTTTTTTGGGTGTGTCTCTGCACTGCCTTTCCAGGCCGATTTTCCC

TTACTGGGTTTTCATTCTTTCCAGGAGAGAGAAGAAGATGGTGTCAAATACAGTGAGGAA

AAGAATGGAACTAATGAACCGCAGGAGTTTACAGTGAATTCGAGAGGGATAAAGTTGTTT

ACGTGCAATTGGGTTCCGTTGAAGCAAGATGCAAGAGCTTTGATCTTTCTTTGCCATGGA

TATGGCATGGAGTGCAGCGTCACCATGAACACAGGGACGGCGATGAGGCTGGCGAAAGAA

GGATACGGAGTACATGGGATGGATTACGAAGGACATGGGAAATCAGGTGGATTACAAGGA

TACATAGAAAACATTGATTCTGTGGTTGAGGATTGCTCCAAACATTTCACCAACATATCC

GAGGAGGAAGAGAAGAAGAGATCAGGGAAGAAGATAGAGAGGTATATGATGGGAGAGTCG

TTGGGGGGAGCAGTGACGTTGATGCTCCACCGATCAATGCCACTTTTCTGGGACGGTGCC

ATTTTGCTGGCTCCTATGTGTAAGATAGCCGACGATGTGAAGCCTTCCCCGTTGATCACT

AACGTTCTCACAAAGCAACAAGTGAGAGAGAATCTGCTTTGCTACAAGGGCCGACCTCGT

TTGAAGACAGCATTTGAACTTCTTAGAGTTAGCATTGATATTGAGCAACGACTTCACGAG

GTGACAATGCCGTTTCTGCTTCTCCACGGGGAAGAAGATAGAGTGACAGATAAGTCGGTT

AGCAAACAACTCTTCGATGTAGCGTCGAGCAAAGACAAGACAATCAAGTTGTACGACGGA

ATGTGGCATGGCCTGCTGTACGGGGAGACACCGGAGAATGTAGAGCTTGTGTTTAAGGAC

ATTACATCATGGCTTGAGCAGAGAACCAGCAGCAGTACTAGTACCAAGGTTTTGGATTCA

ACAATGTTGGAAATGGAGCAAAAGGTTGGGAATGACAAGAAGAATTGTCCCACCACAACT

CCTGCTAGCTGA

**AFSQ01011295**

ATGCAGGAGCAACAAGAAGAAGAAGGAGAAGAGCTCCATTACTGGGGGGATCTGCCAGAG

GTAGAATACTACAAGCAGCAGAAGATCAGAGCTGGACATTCTTACTACACATCGCCCAGA

GGTCTGAAGCTTTTCACCAGATCATGGCTGCCCAACTCGTCGAAGCCGCCCCGAGCTTCG

ATCTGTATGGTCCATGGATACGGCAACGACTGCAGCTGGACGTTCCAATCCACTGCCATC

TTCCTAGCGAAGAATGGCTTCGCCTGCTTCAGCCTCGACATCGAAGGCCACGGCAGATCC

CAAGGTTTGAAAGCCTATGTTCCTAATATTGACCTCGTCGTTGATGACTGCTCGTCGTAT

TTCGCAACCGTGAAACGAGATCCCTTGTTCGCCGGCGTACGCTCCTTCCTCTACGGCGAA

TCGATGGGCGGCGCCATGTGCCTCCTCCTCCACTTCGCTAACCCTAGCTCGTTCGACGGC

GCCGTCTCGATCGTTGACTGTTATGGGAGGCGAAACAGTGTTGGTATCTCCGATGTGCAG

AATCGCGGACGAGATCAAACCAAAGTGGCCGATCCCGGAGATATTGACCTTCGTGGCGAG

ATTTCTCCCAACGCTGGCGATCGTGCCGTCGGGAGACGTGCTGAGGAGCTCGGTGAAGGT

GAAGGACAAGCTGGAAGTGGCGGAGATGAATCCGACGAGGTACAGAGGGAAGCCGAGGCT

GGGGACTGTCCTGGAGCTGATTCGAGTGACGGATTGTCTAGCGAAGAGGCTAACCGATGT

GAGCTTGCCGTTCATGGTCTGCCACGGGAGCGCGGACGTGGTGACGGATCCGAACGTGAG

CAGGTCGCTGTACGAAGAAGCCAAGAGCGAGGACAAGACGGTGAAGATCTACGACGGGAT

GATGCATTCGTTGCTGTTCGGGGAAACGGACGAGAACATCGAGGTTGTTCGTCGGGATAT

TCTGGATTGGATCAGCGATAG

**AFSQ01017841**

ATGCAGGAGCCACAGGATGAAGGAGAAGAACAAGAGCTCCATTACTGGGGGGATCTACCA

GAGGCAGAGTACTACAAGCAGCAGAAGATCAGAGCTGGACATTCTTACTACACATCGCCC

AGAGGGTTGAAGCTTTTCACCAGAACATGGCTGCCCAACTCGTCGAAGCCGCCACGAGCC

TTGATCTGTATGGTCCATGGATATGGCAACGACTGCAGCTGGACATTCCAATCCACTGCC

ATCTACCTAGCGAAGAATGGGTTCGCCTGCTTCAGCCTTGACATCGAAGGCCACGGAAGA

TCGCAAGGTTTGAAGGCTTACGTTTCTAACATTGACCTCGTCGTTGATGACTGCTCGTCG

TATTTCGGTGCCGTGAAACGAGATCCTTTGTTCGCCGGCGTAAGGTCATTCCTTTATGGA

GAATCGATGGGCGGCGCTATGTGCCTCCTCATCCACTTTGCTAACCCTAGCTCGTTTGAC

GGCGCCGTTGGGGAAAACAGTATTGGTGTCTCCGATGTGCAGGATCGCGGACGAGATAAA

GCCGAAGTGGCCTATCCCGGAGATATTGACGTTCGTGGCGAGATTCCTGCCGACACTGGC

GATTGTGCCGTCGGGAGACGTGCTGAGGAGCTCGGTGAAGGTGAAGGACAAGCTGGAAGT

GGCGGAGATGAATCCGACGAGGTACAGAGGGAAGCCGAGGCTGGGGACTGTCCTGGAGCT

GATTCGAGTGACGGATTGTCTAGCGAAGAGGCTAACCGATGTGAGCTTGCCGTTCATGGT

CTGCCACGGGAGCGCGGACGTGGTGACGGATCCGAACGTGAGCAGGTCGCTGTACGAAGA

AGCCAAGAGCGAGGACAAGACGGTGAAGATCTACGATGGGATGATGCATTCGTTGCTGTT

CGGGGAGACGGACGAGAACATCGAACTTGTTCGTCGGGATATTCTGGATTGGATCAGTGA

**CAFFEIC ACID 3-O-METHYLTRANSFERASE**

**AFSQ01002471**

ATGGGCTCAACACCAGAAACCCAGATGACCCCAACCCAGATCTCCGACGAGGAAGCGCAT

CTCTTCGCTATGCAGCTAGCCAGCGCATCGGTCCTACCAATGGTCCTCAAATCAGCACTC

GAGCTCGACCTTCTCGAGATCATTTCCAAATCTGCCGGGTCTGGAACTTACCTTTCCCCT

TCCGAAATCGCCTCTCACCTACCCACTAAAAACCCAGAAGCTCCCGTCGTTCTTGACCGC

ATCTGCCGCCTCTTGGCTTCTTACTCTGTTCTCACTTGCTCCCTCCGTCCTTTATCGGAC

GGCACCGTCGAGCGCCTCTATGGGCCCGCCCCTGTCTGCAAGTTCCTCACCAAGAACGAG

GACGGCGTCTCCATCGCCGCCCTTGCTCTCATGAACCAGGACAAGGTCCTCATGGAGAGC

TGGTATCACTTGAAAGATGCGGTGTTGGACGGAGGGATCCCGTTCAACAAGGCGTACGGG

ATGACAGCGTTCGAGTACCACGGTACCGACCCACGTTTCAACAAGGTGTTCAACAAAGGG

ATGTCTGATCATTCCACCATCACAATGAAGAAGATCTTGGAGACTTACACAGGATTCGAC

GTCCTGAAGTCGCTGGTGGATGTTGGTGGTGGCACTGGAGCTGTTCTGAGCATGATCCTA

TCCAAGCACCCTTCCATTAAAGGGATTAACTTCGATTTGCCTCATGTCATTGAAGACGCC

CCTGCTCTTCCTGGCGTCCAGCACGTTGGTGGTGACATGTTTGCCAGCGTCCCCACTGGA

GATGCCATTTTCATGAAGTGGATATGCCATGACTGGAGTGACCAGCACTGCCTGAAGTTC

CTGAAGAACTGCTTCGACGCACTGCCAGCAAACGGGAAGGTGATAGTGTGCGAGTGCATA

ATGCCAGTGGCTCCGGACACGGGCCTGGCGACGAGGAACGTGGTGCACATAGACTGCATC

ATGTTGGCTCACAACCCAGGTGGCAAAGAGAGGACCCAGGCGGAGTTTGAGTCCTTGGCT

AAGGGCGCTGGGTTCCAGGGCTTCCGAGTCGTGTGCTCTGCTTTCAACACCTATGTCATG

GAGTTCCTCAAGACCGCCGCCTAA

**AFSQ01009084**

ATGGGCTCAACACCAGAAACCCAGATGACCCCAACCCAGGTCTCCGACGACGAAGCGAAT

CTCTTCGCTATGCAGTTAGCCAGCGCCTCCGTTCTACCAATGGTCCTCAAATCAGCACTC

GAGCTCGACCTTCTCGAAATCATTTCCAAATCTGCCGGCTCTGGATCTTACCTTTCCCCT

ACCGAAATCGCCTCTCACCTACCCACTAAGAACCCAGAAGCTCCCGTCGTTCTAGACCGC

ATCTGCCGCCTCTTGGCTTCTTACTCTGTTCTCACTTGCTCCCTCCGCACTTTACCGGAC

GGCACCGTCGAGCGCCTCTACGGCCCCGCCCCTGTCTGCAAGTTCCTCACCAAGAACGAG

GATGGCGTCTCCATCGCCGCCCTCGCTCTCATGAACCAGGACAAAGTCCTCATGGAGAGC

TGGTATCACTTGAAAGACGCGGTGTTAGACGGAGGGATCCCGTTCAACAAGGCGTACGGG

ATGACGGCGTTCGAGTACCACGGTACCGACCCGCGTTTCAACAAGGTGTTCAACAAAGGG

ATGTCTGATCATTCCACCATCACAATGAAGAAGATCTTGGAGACTTACACCGGGTTCAAC

GGCCTGAAATCGCTGGTAGATGTTGGTGGTGGTACCGGAGCTGTTCTGAGCATGATCCTA

TCCAAGCACCCTTCCATTAAAGGGATTAACTTCGATTTGCCTCATGTCATTGAAGACGCC

CCTGCTCTTCCTGGCGTCCAGCACGTTGGTGGTGACATGTTCGCCAGCGTCCCCACTGGA

GATGCCATTTTCATGAAGGTGATAAGTTTGACTCTTATGATATGCTTTTACTGGATTGAA

CTAATGTGGATATGCCATGATTGGAGTGACCAGCACTGCCTGAAGTTCCTGAAGAACTGC

TTCGACGCACTTCCAGCAAACGGGAAGCTGATAGTGTGCGAGTGCATAATGCCAGTGGCT

CCGGACACGAGCCTGGCGACGAGGAACGTGGTGCACATAGACTGCATCATGTTGGCTCAC

AACCCAGGAGGCAAAGAGAGGACCCAGGAGGAGTTCGAGTCCTTGGCTAAGGGCGCTGGG

TTCCAGGGCTTCCGAGTTATGTGCTCTGCTTTCAACACCTATGTCATGGAGTTCCTCAAGACCGCTGCCTAA

**AFSQ01016416**

ATGGCAGCAGACCACGATGACGATGAGCTGTACGCGATACAGCTCGCGACAGCTTCCGTC

GTGCCCATGGCTCTAAAGGGCGCGATCGAGCTAGGCGTGTTGGACATCATAAACTCGGCT

GGACCGGGAGCTTCCCTCACCCCTTCTCAGATCGCTTCCCACCTTGCTAATTATGGTGGT

GGTGATCGTGATCGGGTTGAGTCGATTGCTGACCGGCTCGATCCGATCCTCCGACTCCTT

TCTTCCCATTCAATCATGACTCGTGATGATGAGTCGATCCGCGGGTCGTACGGATTGGCG

CCCGTGGCTAGGTTCTTTCTGAAGGGTGGTGATGGGTCTTTGGGTCCTTTGGTGTGTATG

ATTCAGGACAAAGTTATTCTTGATGCTTGGATGCATTTGAAGGATTCAGTGCTTGAGGGT

GGAGGCAACGCATTCTACAAGGCTTACGGAATGAACGGTGGAGATTACTTGGGGAAAGAT

CCACGGTACAGGGAAGCTTTCTTCGGTTCCATCTCAGAGCTCAACCCTCTAATTATGGAG

ACATTCCTAAAAAGGTACTCTGGTTTTGAAGGCGTGGAGACACTTGTGGATGTTGGTGGT

GGCAATGGCTATAGCGTTCATCTAATATCATCTAGGGTTCCGACTATTCGCAAGGTTATC

AACTTCGATCTGCTTTCCGTCGTCGAAAAATCTCCACCATATCCAGGTGTGGAGCATATA

GCAGGAGACATGTTTGAGAGTGTCCCAAAAGGAGATGCCATCTTCATGAAGTGGATTCTG

CACCTATGGGACGATGAACAATGCCTGAAACTCCTCACCAACTGCCACAAATCACTACCA

GAGAATGGCAAAGTTGTAGCACTTGATGCTCTTGTCCCAGAGATCTCTAATGAAGCCATT

ACTGTTGCTGCTAAGGCCATGCTCCAGTTCAGTCTTTACAGAAACAACACCAAACCTCAG

GGCAAAGAGAGGACTCTCATCGAGTTCGATCGCTTGGCTAAACGAGCTGGATTCTCTCAG

GCTCGTGTCGTTTGCTACGCCTACAACTTTGCCGTCTTCGAGTTCCACAAACTTGAATCA

TCATCCATCTAG

**AFSQ01027305**

ATGGCAGCGACCCAAAACGTCACCGTACCAAAAGAAGATCAAAATGAGAGCTATCTATAC

GCAATGCAACTGGTAATGGGGTCAGTGGTTCCTATGACACTGCAGGCTGCCAACGAGCTC

GGAATCTTTAAAGTCATGGCCGCTGGTGCGAAACTGAGCGCCTCTGAAATCGTGTCCAAG

CTGGATTCGATGACGAATCCAGATGCACCGGTCATGGTTGACCGGATTCTTAGGCTAATG

GGTAGTCATTCGATTGTTGATTGCACTTTGGAGGACGATGGGTCCGGCCAGAAACAGAGG

AAGTATGAGTTGAACTCTGTTTCTAAGTACTTTGTGAAGAATGAGGATGGTCTCTCTTTG

GTTCCCTTGATGTCCTTGCTTCAAGATAAGGTCTTCTTGGACAGCTGGCCCGAGCTGAAG

AATGCGGTGTTGGAAGGAGGAGTTCCATTTGACAGAGTCCATGGTGCTAACACTTTCGAG

TATCCGAGACTTAATGAGAGGTTCAATCAAGTTTTTAACGCAGCAATGTACGACCATTCG

ACCATGGTAATTAAACAGGTTCTCAATTCTTACAAAGGATTCGAACCATTGAACCTTGTC

GTGGATGTCGGAGGTGGATTAGGACACACCATAAAGGCCATTACTTCCAAGTACCCACAT

ATCAAAGGCATCAACTTTGACTTGGCCCATGTTATAAACCAGGCTCCTAATATTCCTGGT

GTGGAGAATGTAGCTGGGGATATGTTTAAATGTGTTCCTCAAGGAGATGCCATTTTTATG

AAGTGGATACTCCATGATTGGAGCGACGTCCATTGTTTGACACTATTGAAGAACTGTTAT

AAGGCAACTCCGACACAGGGGAAGGTAATAGTTATGGATGCGGTGGCTCCGATAATGGCG

GAGACGAATGCGAGCTCAAAATTAACCGCTCAAATTGATATGGTAATGCTGACACAAGAT

CCTGGAGGGAAAGAAAGAACAAAAGACGAGTTCATGGCTCTAGCAATCGGAGCTGGTTTC

AAAGGGATCCGATTTGAGTTACATGTTTGTAACTATTGGGTGATGGAGTTCTACAAATGA

**AFSQ01011000**

ATGACATTGCAGGCCGCCAACAAACTCGGAATCTTCGACATCATGGCTGCAGATGCCTCT

GGTGCAAAACTAAGCGCCTCTGAAATCGTGTCCAAGTTGGATTCGGTGACGAATCCAGTT

GCTCCGGTCATGGTTGACCGGATTCATAGGCTATTATTGGGGAGTCAATCCATTGTGGAT

TGCACTTTGGAGGAGAATGGGAAGGAGCTGAAGAATGTGGTGCTGGAAGGAGAAGAATAC

AATAATGTAACTCGTACCAACCACCTCTCTTCAATACAAGTAATCTCTCCATCTCCTCTT

CACAATCTATCACAGCCCATTTCTTTCATCCATGGCCGCCGTAACCAGTCCGCCGGACGA

CCAAGAGAGCTTTTTACATACGCTATGCAGCTGGTGATGGGCTCAGCGGTTCCCATGACA

TTGCAGGCTGCTAACGAACTCGGGATCTTCGAGGCGATGGCTGCAGAGGGTGATGGTGCA

GAACTCAGCGCTTCTGAGATCGTGTCCAAGTTGGATTCGGTGATGAATCCAGATGCTCCG

GTCATGGTGGACCGGATTCTTAGACTATTGGGTAGCCATTCGATCGTCGATTGTACGTTG

GATGTGGACGATGGGTCTGGCCAGAAGCAGAGGAAGTACGGGCTGAACTCTGTTTCAAAG

TACTTTGTAAAGGATGAAGATGGTCTCTCTTTGGTTCCTATGATGTCCTTGATTCAAGAC

AAACTAGTCATGGCATCCTGGTCTGAGCTGAAGAACGCAGTGCTAGAAGGAGGAATCCCA

TTCAATAGAGTCCATAACATCGGAAATTTCGAATGTACAAGACTAGATCCAAAGTACAAT

CGAGTTTTCAGCGCAGCAATGTTCGATCATACAACCATGGTTATGAAACAAGTCCTTCAT

TCCTACAAAGGGTTTGAAAGGTTGAAGATTCTCGTCGACGTGGGAGGTGGATTAGGACAC

ACCATTAAGACAATAACTTCAAAGTACCCTCAAATCAAGGGAATCAATTTCGACTTGCCT

CATGTCTTGATACTCCATAACTGGAGTGATGCACATTGTATGGTGCTATTGAAGAACTGT

TACAAGGCGACTCCGGAAGATGGAAAGGTCATAGTTATGGATACTGTGGCACCAAACACG

CCCGAGGCGACGACAAGCTCGAAGTATACCGCTCAACTCAATGTGTTGATGATGGCGGTA

AATACAGGAGGGAAGGAGCGGACGAGAGATGAGTTGATGGCGTTAGCGATCAGAGCTGGG

TTTAAAGGGATTCGATTCGGGGGGGGGTAG

**AFSQ01024646**

ATGGATAGTACTAGAGAAGCCAACTTTCAGTTTGCCATGTGGCTTGCAGTCGGCCAAGTG

CTGCACAATACCATGCATGCAGTGAACGAGCTCGGAATTTTTGACATCATGTCGGAATCG

GGAACTGATGCTAAACTCAGCGCTCCCGAGATCGTGTCAAAATTGGGATCAAAGAACCCT

GATGCTCCGGCCATGGTAGACCGCTTTCTTAGGTTGTTGGCTACTCACTCAATCGTCGAC

TGCACGGTGGACGGTGCCAGCCCGGGTAGGATATACTATTACACCCTGAACTCTGTTTCC

AAATACTTTGTGAGGAATGAGAACAATGGTGTCTCGTTGGCTCCACTTCTGGCCTTGACT

CAGGACCATATCCTCGAGAAGAGCTGGTCTCAAGTGAAAGATGCGGTACTAGAAGGTGGG

ATACCGTTTGATAGGGCACACGGGTCCCATATTTTTGAGTATCCTAGTCAAGATCTAAGG

TTTAATCACACCTTTAACGATGGGATGCTTAACCAAAGCACTCTCGTCATTAAGGAGGTC

CTCGATTCCTACGAAGGATTCGAGAATCTGAAAACGATGGTTGATGTCGGTGGTGGATTA

GGACATACTATAAGTGCCATCACTTCCCGGTACCCACATATCCAAGGAATCAATTTTGAC

TTGCCACACGTCATCAAACAAGCTCCGCCTATCCCTGGGGTGGAACATGTGGCAGGGGAT

ATGTTTGAAACAGTTCCTCAAGCAGAAACAATTTTCCTCAAGTGGGTACTACATGATTGG

AGCGACGAAGGTTGTCTGACGTTGTTGAAGAACTGTTACAAGGCAACTCCAGAAGATGGG

AAAGTGATAGTTATGGATGCTGTGGCCCCGGTGATGGCTGAGACAACCATGAGTTCGAAG

CTAACCGCTGCATTTAATGTCTTAATGACGATATTATGCCAGGGAGGGAAAGAACGAAGC

AAAGATGAATTCATGTCGCTAGCAATTGGGGCAGGATTTAGGACCATCAAGTTTGAATGC

TTTGTTTGCAATTTTTGGATCATGGAGTTTCTCAAGTAG

**CAFFEOYL-COA O-METHYLTRANSFERASE**

**AFSQ01021237**

AACTCCCTTCTCGCCACCGCCCTCGCTCTCCCCGACGACGGAAAGATCTTGGCAATGGAT

ATCAACAGAGAGAACTACGAAATCGGCCTGCCAATCATCGAGAAAGCCGGACTTGCTCAC

AAGATCGAGTTCAAAGAAGGCCCTGCACTGCCCGCTCTTGACAAGATGGTCGAAGACAAA

GCGAACCACGGAGCGTACGACTTCATATTTGTGGACGCGGACAAGGACAACTACATCAAC

TATCACAAGAGGCTGATCGACTTGGTGAAGATCGGTGGAGTCATCGGGTACGACAACACC

CTATGGAACGGGTCCGTGGTTGCGCCTCCTGATGCGCCGTTGAGGAAGTACGTCAGGTAT

TACAGGGACTTCGTTTTGGAGCTGAACAAGGCGCTCGCTGCTGACCCGAGAATCGAGATC

TGCATGCTCCCCGTTGGAGACGGAATCACTCTCTGCCGCCGGATCAGCTAA

**AFSQ01027261**

ATGGCTGAGCAACAGCAATCGGGGGAGAATGTCAGCAGGCATCAGGAAGTCGGCCACAAG

AGTCTCCTCCAGAGCGATGATCTTTACCAGTATATTCTCGAGACGAGCGTTTACCCAAGG

GAGCCGGAGTCGATGAAGGAACTCCGAGAGGTCACCGCCAAACACCCCTGGAATATCATG

ACCACGTCTGCCGACGAAGGACAGTTCCTCAACATGCTACTGAAGCTCATCAATGCTAAG

AACACAATGGAGATCGGCGTCTACACTGGCTACTCCCTTCTCGCCACCGCCCTCGCTCTC

CCCGACGACGGCAAGATCTTGGCAATGGATATCAACAGAGAGAACTACGAAATCGGCCTG

CCAATCATCGAGAAAGCCGGACTTGCTCACAAGATCGAGTTCAAAGAAGGCCCAGCACTC

CCCGCTCTTGACAAGATGGTCGAAGATAAAGCCAACCACGGAGCGTACGACTTCATATTT

GTGGACGCGGACAAGGACAACTACATCAACTATCACAAGAGGCTGATCGACTTGGTGAAG

ATCGGTGGAGTCATCGGGTACGACAACACCCTATGGAACGGGTCCGTAGTCGCGCCTCCT

GATGCGCCGTTGAGGAAGTACGTTAGGTACTACAGGGACTTCGTTTTGGAGCTGAACAAG

GCGCTCGCTGCTGACCCGAGGATCGAGATCTGCATGCTCCCCGTTGGAGATGGAATCACT

CTCTGCCGCCGGATCAGCTAA

**CINNAMOYL-COA REDUCTASE**

**AFSQ01011029**

ATGGGGGAAATAGAAGTGAAGGCAGCAGAGAACGTGGTGGAGGCATGCTCAGCAACTCCA

TCAGTGAGGAGCTGTGTGGTGACTTCATCGCTTCTGGCCTGCATTTGGCGAGACAGCACC

TTGCAGGATTACCCTTGTGTCATCAACCACAACTCCTGGAGCGACGAATCACTTTGCAGA

GATAAAAGGCTGTGGTATGCATTGGGGAAGCTGAGAGCAGAGAAAGCAGCTTGGAGTAAA

GCAAAAGAGAAAGGGGTAAAACTGGCGACTATCTGCCCGGGACTCGTCACTGGTCCTGAA

TTCTTCGCCAGAAATCCAACATCAACAGTCGCCTACTTAAAAGGAGCACAAGAAATGTTT

GCAGATGGGCTGCTAGCAACAGTGGATATAATGAAGGTAGCAGAAGCACATGTGTGTGTG

TACGAGGGGATGAGGAAGACGGCAGGTGGGAGGTACGTATGCTACGATAAGGTGATAGAG

AGGGAGGAGGAGGCGGAGAAGCTGGCGGAAGAAGTTGGGATGCCGGCGGAGAGGATATGT

GGGGAAAGAGGGGAATATATTCCAGCCCCATTCCAGTTATCAAACAGGAAGCTTTCTAAC

CTCATGTCCACAACTCTTCGTACTTGTTACAATCACAGCTG

**AFSQ01010549**

ATGAGTCATCGAGTGGGTCTTGAATTTATTGAATCAAGTCCTAAAGCTTTGCTTGAATTC

ATAAATCGCCTTGCGCAAACGGCATACAATGGCTTAGGAAGTCCGAATGTTCAAATTTAT

GAGGATGAGCTAGAAGATACACTTTCTCGCCAACCCACTACACCTCCTCCCATTCCAACA

ACCAATTCACCCTCCTCTTCATCCTTTTATCCCAAAAAAGCATACACTCACAAACAACGA

TGCCGGCTGACAGCTCATCCTTACCTGGCCACGGCCAGACCATCTGCGTCACCGGCGCCG

GCGGCTTCATTGCCTCCTGGATGGTTAAGCTTCTTCTCGAAAGAGGCTACTCTGTCAAAG

GAACTGTCAGGAATCCAGGTTTCCTTTTCCTTCCTCTTTCAATTCATCCATTTCGATCCC

GGCGATGCGATTCCCAATCGGATCTTCATATTGCTTTTTCTTTTCCCGGCTATCGCAGAT

GATCCGAAGAACAGTCATTTGAGAGAACTGGAAGGAGCTAGCGAGCGGCTGACTCTGTGC

AAAGCCGATCTTCTCGACTATGAGAGCCTCCGAGAAGCCATTACCGGCTGCCAGGGCGTT

TTCCACACCGCTTCCCCTGTCACCGACGATCCAGAACAAATGGTGGAGCCGGCTGTGGTT

GGTACGAAGAATGTAATTAATGCTGCAGCTGAAGCCCAAGTCCGGCGGGTTGTGTTCACG

TCATCAATTGGCGCTGTCTACATGGACCCCAACCGGAGCCCGGACGTCGTCGTGGATGAA

TCTTGTTGGAGCGATCTGGAGTTTTGCAAAAACACCAAGAATTGGTACTGTTATGGGAAG

ATGGTGGCGGAGCAGGCGGCATGGGAGACAGCGAAGGAGAAGGGAGTGGATGTAGTGGCG

GTGAACCCGGTTCTGGTAATGGGACCATTGTTGCAATCAACCATCAACGCCAGCACAATT

CACATCCTCAAGTACCTAACTGGTTCTGCCAAGACTTACGCCAATTCGGTTCAAGCCTAT

GTTGATGTCAGGGACGTGGCACTAGCTCACATTATTGTCTTCGAGAACCCAGCGGCTTCC

GGCCGGTACCTTTGCGCCGAGAGTGTGCTCCACCGTGGTGAGGTTGTCGAAATCCTGGCC

AAGTTATTCCCCGAGTATCCTGTCCCCACCAAGTGTTCAGATGAGAAGAACCCAAGAGCA

AAGGCATACAAGTTCTCATGCCAAAAACTCAAGGACTTGGGACTGGAATTCACACCAACG

AAGCAGAGCCTGTATGAAGCAGTCAAGTCCCTTGAGGAAAAGGGTCATCTTTCTCCCCCT

AAACAACAGCAGCACAAGGAGGAGGAACCCATCAAAATTCAGTCCTCTTCTTAA

**AFSQ01020983**

ATGGTGGAGCCGGCTGTGGTTGGTACGAACAATGTAATAAATGCTGCATCTGAAGCCAAA

GTCCGGCGGGTTGTCTTCACGTCATCAATTGGCGCTGTCTACATGGACCCCAACCGGAGC

CCGGACGTTGTCGTCGATGAATCGTGTTGGAGCGATCTGGAATTTTGCAAAAACACCAAG

AATTGGTACTGTTATGGGAAGATGGTGGCGGAGCAGGCGGCGTGGGAGACTGCTAAAGAG

AAGGGAGTGGATGTAGTGGCGGTGAACCCTGTTCTGGTAATGGGACCATTGTTGCAATCA

ACCATCAACGCCAGCACAATTCACATCCTCAAGTACCTAACTGGTTCGGCAAAGACTTAC

GCCAATTCGGTGCAAGCCTATGTTGATGTCAGGGACGTGGCACTAGCTCACATCATCGTC

TTCGAGAACCCGGCGGCCTCCGGCCGGTACCTCTGCGCCGAGAGTGTTCTCCACCGTGGC

GAGGTTGTCGATATCCTTGCCAAGTTATTCCCCGAGTATCCCGTCCCCACCAAGTGTTCA

GATGAGAAGAACCCAAGAGCAAAGCCCTACAAGTTCTCGTGCCAAAAACTGAAGGACCTG

GGACTGGAATTCACACCAACAAAGCAGAGTCTGTATGAAGCAGTCAAGTCCCTTGAGGAA

AAGGGTCATCTTTCTCCCCCTAAACAGCAGCAGCAGCAGCACAAGGAGGAGGAACCCATC

AAAATTCAGTCCTCTTCTTAA

**AFSQ01004224**

ATGCCAGCAGTAGCTGACACCTCATCCATCATCATCACCCCCCAGACAGTCTGCGTCACC

GGCGCCGGCGGCTTCATTGGCTCATGGATCGTTAAACTTCTCCTCCACAGAGGCTACTCC

GTCAGAGGAACCGTCAGGAATCCAGAGGATCCGAAGAATAGACACTTGAAAGAGCTAGAA

GGATCCAATGAGCGGCTGACCCTGTGGGAAGCTGACCTACTGGATTACGATGGTCTCCGA

CGAGCCATTGATGGATGTCACGGCGTTTTCCACACCGCTTCCCCCATCTCCGATGATCCT

GACGAAATGCTGGAGCCGGCGGTGGACGGGACTAAAAATGTAATAATAGCAGCATCGGAA

GCAAAAGTCCGGCGAGTTGTGTTCACGTCGTCCATCGGCGCCGTCTACATGGACCCCAAC

CGAAACCATGACGTGGAGATGGATGAGTCCTTTTGGAGCGATCTCGACTATTGCAAGAAC

ACCAAGAACTGGTACTGCTACGGCAAAACGGCAGCGGAGCTGAGAGCATGGAAGGTGGCG

AAAGAGAAGGGTGTGGATTTGGTTGTTGTCAACCCTGTGGTTGTGGTCGGACCGTTGCTC

CAACCAACTGTCAACGCTAGCAGCGTTCACATACTCAAGTACCTAGCCGGTTGGGTAAAA

ACCTATGCCAATGCAGTTCAAGGGTATGTTCACGTTAGAGACGTGGCACTAGCCCACATC

CTCGTGTTTGAGAACATGTATGCATCTGGTCGCTACCTTTGCGTCGAAAGTGTCCTCCAC

CGAGGCGAGGTGGTTGAGATTCTCGACGAGTTGTTCCCCGGTTATCCCCTCCCAACCAAA

TGCAAGGACGAGAAGAGCCCCAGAGCCAAACCGCACAAGTTTTCGTGCCAAAAGTTGAAG

GACATTGGACTGGAGTTCACACCGGGTCAGCTTGATATGATTTTCCAACATTATGGTGTT

ACCCATAGATTGACGAATACTTACCACAACATGCAAGTGGATGTGATGAATCGCGAGCTT

AAGAGAACTCGTAAAAAGACGGTCCACCAATCTCACAGTGATTGCTCGATTAAGCTCGAT

GAGGCCTCGTAA

**AFSQ01029711**

ATGTCGGGGACTATGAAGGTAGGGAAACAGATAAGTAGTAACCTAAAAATAGGAAAGAGG

TCCGACCGGAGAGGGAACTGGTACTGCTACGGCAAAACGGCAGCGGAGCTGAGAGCATGG

AAATTGGCGAAAGAGAAGGGTGTGGATTTGGTTGTTGTCAACCCTGTGGTTGTGGTCGGA

CCGTTGCTCCAACCAACTGTCAACGCTAGCAGCGTGCACATACTCAAGTACCTAGCCGGT

TGGGTAAAAACCTACGCCAATGCAGTTCAAGGGTACGTTCACGTTAGAGACGTGGCACTA

GCCCACATCCTCGTATTCGAGAACATGTATGCATCTGGTCGCTACCTTTGCGTCGAAAGT

GTCCTCCACCGCGGTGAGGTGGTTGAGATTCTCGACGAGTTGTTCCCCGGTTATCCCCTC

CCAACCAAGTGCAAGGACGAGAAGAGCCCTAGAGCCAAACCTCACAAGTTTTCGTGCCAA

AAGTTGAAGGACATTGGTTTGGAGTTCACTCCGATTGACGATCGTGCGCTTATCACCCCA

GACAAGTGGTCAAGTGGACGTGACGAATCACAAGCTCAAAAGAATTCATAA

**AFSQ01007861**

ATGAGCTCCGGCGAAGGCCAAACCGTTTGCGTCACCGGCGCCTCCGGCTACATCGCCTCT

TGGATCGTCAAGCTCCTCCTCTCTCGCGGCTACACTGTCAAGGCCTCCGTCCGCGATCCA

AACGATCCGAAGAAGACCGAACACTTGCGCGCTCTCGACGGAGCTTCCGACCGACTGTTG

CTGTTCAAGGCAAATCTGCTGGAGGAAGGCTCGTTTGATTCCGGCGTCGAAGGCTGCGTC

GCCGTTTTCCACACCGCTTCCCCTTTCTATCACGACGTCAAGGATCCTCAGCTAGAATTG

ATTGATCCGGCGTTGAAAGGCACGCTGAATGTGCTGAATTCATGTGCGAAAACTCCGTCC

GTCAAGCGAGTTGTCTTGACGTCTTCGATTGCTGCAGTGGCTTACAATGGCAAACCACGT

ACTCCAGAAGTTGTAGTTGATGAGACTTGGTTTTCGAACCCTGACTTTTGCAGAGAGTCT

AAGCTTTGGTACGTGGTCTCCAAAACACTGGCAGAGGATGCTGCATGGAAATTTGCAAAA

GAGAAAGGCATGGACTTGGTTGCCATAAACCCTGCTATGGTTATTGGTCCTCTGTTGCAG

CCAACACTTAACACTAGTGCCGCAGCTGTTCTAAGCATCATGAAAGGAGCTCAGACATTC

CCGAACGCTACATTTGGATGGGTACACGTGAAAGATGTGGCAGAGGCACACATCAAAGCT

TTTGAGATCCCATCAGCTAATGGAAGATATTGTTTGGTTGAGACAGTTGCACACTACTCT

GAAGTTGTGAAAACCCTAAAGAAACTTTACCCTAGTGTCCAACTCCCGGAAAAGTGTGCG

GATGACAAGCCTTCCGTTCCAACATACCAGGTTTCCAAGGAGAAGGCAAAAACCTTGGGG

ATTGAGTTCATCCCCTTGGAAGTAAGTCTGAAGGAGACTGTGGATAGCTTGAAGGAGAAA

GGATTCGTCGACTTCTGA

**CINNAMYL ALCOHOL DEHYDROGENASE**

**AFSQ01005963**

ATGGGTAGCATGGAAAAGGGAAGAACCATCGTTGGTTGGGCAGCTACAGACCCTTCCGGC

GTCCTCGCTCCTCAAACTTTCACTCTCAGGAACACGGGCCCAGAAGATGTGTTCATCAAA

GTTATGTGCTGCGGAATCTGCCACACTGATATCCACCAAATCAAGAACCACCTCGGCATG

TCTCGTTACCCTATGGTTCCTGGACACGAAATGGTTGGCGAGGTATTGGAAGTGGGATCG

GAGGTGACCAAGTTCAAAACCGGAGACATAGTCGGCGTCGGACTCCTCGTCGGATGCTGC

CGGAGCTGCCGCGCATGTGATGCTGACATCGAGCAGTACTGCAACAAGAAAATCTGGTCA

TACAACGACGTCTATACCGACGGGAACCCAACTCAAGGCGGATTCGCCGAGTCCATGATT

GTCGACCAGAAGTTTGTAGTGAAGATCCCAGAAGGGATGTCGCCCGAGCAAGCAGCTCCT

TTGCTATGCGCAGGGGTGACGGTTTACAGTCCGCTGAATCACTTCGGGCTGAAGAAGAGT

GGGTTGAAAGGAGCGATCTTGGGGCTAGGAGGCGTTGGGCACATGGGAGTTAAGATTTCC

AAAGCAATGGGACACCATGTGACTGTAATTAGCTCCTCTGATAAGAAAAAGGTTGAAGCA

CTGGATCATCTCGGAGCTGATGAGTATTTGGTCAGTTCGGATTCGTCAACAATGGAACAA

GCTGCTGATTCGTTCGATTACATAATCGACACTGTCCCTGTGAACCACCCGCTCGAGCCT

TACCTATCGCTGTTGAAGGTCGATGGGAAGTTGATCTTGATGGGTGTGATCAACACCCCT

CTTCAGTTCATAAGTCCTATGGTGATGCTAGGGAGGAAGGTGATAACAGGGAGCTTCATA

GGGAGCATGAAGGAAACAGAGGAGATGCTGGAGTTCTGCAAGGATGAAGGGTTGAGCTCC

ATGATTGAGGTGGTGAAGATGGACTATGTGAATACGGCGTTCGAGCGGCTAGAGAAGAAT

GATGTGCGGTATAGGTTCGTGGTTGATGTTGCAGGAAGTGATCTTCAGCAGCAGTGA

**AFSQ01016784**

ATGGGTAGCATGGAAAAGGGAAGAACCATCGTTGGCTGGGCAGCTACTGACCCTTCCGGC

GTCCTCGCTCCTCAAACTTTCACTCTCAGGAACACGGGCCCAGAAGATGTGTTCATCAAA

GTTATGTGCTGCGGAATCTGCCACACTGATATCCACCAAGTCAAGAACCACCTCGGCATG

TCTCGTTACCCCATGGTTCCTGGACATGAAATGGTTGGCGAGGTATTGGAAGTGGGATCG

GAGGTGAACAAGTTCAAAACCGGAGACATAGTCGGAGCCGGACTCCTTGTCGGATGCTTC

CGGAGCTGCCGCGCATGTGATGCTGACATCGAGCAGTACTGCAACAAGAAAATCTGGTCC

TACAACGACGTCTACACCGACGGGAACCCAACTCAAGGAGGCTTCGCCGAGTCGATGGTT

GTCGACCAGAAGTTTGTAGTGAAGATCCCAGAAGGGATGTCGCCGGAGCAAGCAGCGCCA

TTGCTATGCGCAGGGGTGACGGTTTACAGTCCTCTGAACCACTTCGGGTTAAAGAAGAGT

GGGATGAAAGGAGCAATATTGGGGCTAGGAGGAGTTGGCCACATGGGAGTCAAGATCGCC

AAAGCAATGGGACACCATGTGACTGTAATTAGCTCCTCGGATAAGAAAAAGATCGAAGCA

GTGGATCACCTCGGAGCTGATGAGTATTTGGTCAGCTCGGATTCGGCAACAATGGAACAA

GCTGCTGATTCGTTCGATTACATAATCGACACTGTCCCTGTGAACCACCCGCTCGAGCCT

TACCTCTCGCTGTTGAAGGTTGATGGGAAGTTGATCTTGATGGGTGTGATCAACACCCCT

CTTCAGTTCATAAGTCCCATGGTGATGCTAGGGAGGAAGGTGATAACAGGGAGCTTCATA

GGGAGCATGAAGGAAACAGAGGAGATGCTTGAGTTCTGCAAGGATGAAGGGTTGAGCTCC

ATGATTGAGGTGGTGAAGATGGACTATGTGAATACTGCGTTCGAGCGGCTGGAGAAGAAT

GATGTACGGTATAGGTTCGTGGTTGATGTTGCAGGAAGTGATCTTCAGCAGCCGTAA

**AFSQ01021479**

ATGGGTAGCATCGGAAAGGAAAGAACCATCGTCGGCTGGGCCGCCACCGATCCTTCAGGC

GTCCTCACTCCTCACACCTTCACTCTCAGGAACACCGGCCCTGAAGATGTGTTCATCAGA

GTGAAGTGCTGCGGAATCTGCCATAGCGACATCCACCAGGTGAAGAACGACCTTGGAATG

TCTCGTTACCCCATGGTCCCTGGACATGAAGTGGTGGGGGAGGTAATGGAGGTGGGATCG

GAGGTGACCAAGTATAAAGCAGGGGACATCGTCGGAGTCGGACTCCTCGTCGGATGCTGC

CGGAGCTGCCGCGCTTGCGAATCCGACATCGAGCAGTACTGCAACAAGAAGATCTGGTCG

TACAACGACGTGTACACCGATGGGAAACCCACGCAAGGCGGGTTTGCAGAGTCCATGGTC

GTTGATCAGAAATTTGTGGTGAAGATCCCGGAAGGGATGTCACCTGAGCAGGCTGCACCC

CTGCTATGCGCAGGGGTGACGGTTTACAGCCCGCTGATCCATTTCGGGCTGAACCAAACT

GGATTGAGGGGCGGCATCTTGGGGCTTGGTGGAGTTGGCCACATGGGAGTCAAGATTGCT

AAAGCAATGGGTCACCATATAACTGTGATAAGCTCTTCTGATAAGAAGAGAGTTGAGGCA

TTGGAGCATTTGGGAGCTGATGACTATCTGGTTAGCTCTGATAAGGAAAAGATAGAAGCG

GCTGCCGATTCGTTCGATTACATCATTGATACCGTACCGGTTAACCACCCGCTCGAGCCA

TACCTTTCTCTGTTGAAGGTTGATGGAAAGTTGATCTTGATGGGTGTTATCAACACACCT

TTGCAGTTTATCAGCCCCATGGTCATGCTTGGGAGGAAGGCCATAACGGGGAGTTTCGTG

GGGAGCATGAAGGAAATGGAGGAGATGCTTGAGTTCTGCAGGGAGAAAGGGTTGAGCTCC

ATGATAGAGGTGATTAAGATGGACTACATCAACACGGCATTCGAGAGGCTGGAGAAGAAT

GATGTTCGCTACAGGTTCGTGGTCGATGTCGAGGGGAGCGACAAACTTGTTCAGCAATAA

**AFSQ01014217**

ATGGCTCAGACGACACCGAATCACACTCAGACAGTTAGCGGGTGGGCAGCTCAGGACACC

TCCGGCCACATCTCTCCTTACACTTTCAAACGCCGTGAGAACGGAGATACGGATGTAACC

ATAGACATACTCTACTGTGGAGTCTGCCACACGGATCTCCACCAGGCCAAGAACGATTGG

GGCATCACCATGTACCCTATTGTTCCTGGGCATGAGATAACTGGGATAATAAGGAAAGTG

GGGAAGAAGGTGGAGGGTTTTAAGGTTGGAGACAGAGCTGGAATTGGGTGCCTAGCTGCT

TCCTGTTTGGACTGTGAGTTTTGCCGGAGCTCCCAGGAGAATTACTGCGACAAAATGCAG

TTCACTTACAATGGTGTTTTCTGGGACGGCAGCATTACTTACGGCGGCTACTCCAATGTG

ATTGTTTCTGATTACAGGTATGTGGTGCACGTGCCAGAGAACCTCCCGATGGACGCAGCG

GCGCCGCTGCTCTGTGCCGGGATCACCGTGTTCACGCCGTTGAAAGACCACAACTTGATT

GAATCTCCGAGGAAGAAAATCGGGGTAGTTGGGCTGGGCGGTTTGGGGCATGTCGCCGTC

AAATTCGGGAAGGCATTCGGTCACCACGTCACCGTCATCAGCACATCTCCTTCCAAAGAG

AAGGAGGCCAGGGAACGCTTGGGTGCCGACGACTTCATCGTCAGCACCAACCCCACTCAC

ATGCAGGCCGGAAGGAGGACTCTGGACTTTATACTGAATACGGTGTCGGCTGGGCACTCA

TTGGGACCCATTTTGGGGTTGCTAAAAGTCAACGGAACGATGGTCATAGTTGGGGCTCCC

GGAAGTTTGCTTGAACTCCCGGCGTTCTCTCTCATATTCGGGAAAAGTACAGTGAAAGGT

GGGATAATAGGAGGGATGAAGGAGACGCAAGAGATGATGGACTTGTGCGGGAAACATAAC

ATAACCTGTGATATCGAGGTTGTGAAACCGGACAACATTAACAAAGCATTCCAACGCCTA

GCAGCAAATGATGTCAAGTATCGTTTCGTGATTGATATTGCTGGATCACGCTCCTCTCTC

TAA

**AFSQ01023279**

ATGGCTCAAACGACACCAAATCACACGCAGACAGTTAGCGGGTGGGCAGCTCAGGACACC

TCCGGCAACATCTCTCCTTACACTTTCAAACGCCGTGAGAACGGAGATACGGATGTAACG

ATAGACATACTCTACTGTGGAGTCTGCCACACTGATCTCCACCAGGCCAAGAACGATTGG

GGCATCACCATGTACCCTATTGTTCCTGGGCATGAGATAACGGGCATTATAAGGAAGGTA

GGGAAGAAGGTGGAGGGTTTTAAGGTTGGAGACAGAGCTGGAATCGGGTGCCTAGCTGCT

TCTTGTTTGGACTGTGAGTTTTGCCAGACCTCTCAGGAGAATTACTGCGACAAGCTTCAG

TTCACTTACAATGGTGTTTTCTGGGACGGTAGCATTACTTACGGTGGCTACTCCGATATG

ATCGTCTCCGATTACAGGTACGTGGTGCACGTGCCAGAGAGCCTCCCGATGGACGCAGCG

GCGCCGCTGCTCTGTGCCGGGATCACCGTGTTCACGCCGTTGAAAGACCACAACTTGATT

GAATCTCCGAGGAAGAAAATCGGGGTAGTTGGGCTGGGCGGTTTGGGGCATGTCGCCGTT

AAATTCGGCAAGGCGTTCGGTCACCACGTCACCGTCATCAGCACATCTCCTTCCAAGGAG

AAGGAGGCCAGGGAACGCTTGGGTGCGGACGACTTCATAGTCAGCACCAATCCCACTCAC

ATGCAGGCCGGAAGGAGGACCCTGGACTTTATACTGAATACGGTGTCGGCTGAGCACTCA

TTGGGACCCATTTTGGAGTTGCTAAAAGTCAACGGGACGATGGTCATAGTTGGCGCTCCC

GGAAATTCGCTTGAACTCCCGGCGTTTCCTCTTATATTCGGGAAAAGGACAGTGAAAGGT

GGGATAATAGGAGGAATGAAGGAGACGCAAGAGATGATGGACTTGTGCGGGAAACACAAC

ATAACCTGCGACATCGAGGTTGTGAAACCGGTCAACATTAACAAAGCATTCCAACGCCTA

GCAGCAAATGATGTCAAGTACCGCTTCGTCATTGATATCGCTGGATCAGGCTCCTCTCTC

TAG

**AFSQ01022789**

ATGGGTAGCATCGGAAAGGAGAGAACCATCGTCGGCTGGGCCGCCACCGATCCTTCCGGC

GTCCTCACTCCTCACACCTTCACTCTCAGGAACACCGGCCCTGAAGATGTGTTCATCAGA

GTGAAGTGCTGCGGAATCTGCCATAGCGACATCCACCAGGTGAAGAACGACCTTGGGATG

TCTCGTTACCCCATGGTTCCTGGACATGAAGTGGTGGGGGAGGTGATGGAGGTGGGATCG

GAGGTGACGAAGTATAAAGCAGGGGACATCGTCGGAGTCGGGCTCCTCGTCGGATGCTGC

CGGAGCTGCCGGGCTTGCGAATCCGACATCGGGCAGTACTGCAACCAGAAGATCTGGTCG

TACAACGACGTGTACACCGATGGGAAGCCCACGCAAGGCGGGTTTGCAGAGTCCATGGTC

GTTGATCAGAAATTTGTGGTGAAGATCCCGGAAGGGATGTCCCCGGAGCAGGCTGCACCC

CTGTTATGCGCAGGGGTGACGGTTTACAGCCCGCTGATCCATTTCGGGCTGAACCAAACC

GGATTGAGAGGCGGCATCTTGGGGCTTGGTGGAGTTGGCCACATGGGTGTTAAGATTGCT

AAGGCAATGGGTCACCACATAACTGTGATAAGCTCTTCTGATAAGAAGAGAGTTGAGGCA

TTGGAGCATTTGGGAGCTGATGACTATCTGGTTAGCTCTGATAAGGAAAAGATGGAAGAG

GCTGCCGATTCGCTCGATTACATCATTGATACCGTACCGGTTAACCACCCGCTCGAGCCG

TACCTTTCGCTGTTGAAGGTTGATGGGAAGTTGATCTTGATGGGTGTTATCAACACACCT

TTGCAGTTTATCAGCCCCATGGTCATGCTTGGGAGGAAGGCGATAACAGGGAGTTTCGTG

GGGAGCATGAAGGAAATGGAGGAGATGCTGGAGTTCTGCAGGGAGAAAGGGTTGAGCTCC

ATGATAGAGGTGATCAAGATGGACTACATCAACACGGCATTCGAGAGGCTGGAGAAGAAC

GATGTTCGTTACAGGTTCGTGGTCGATATCGAGGGGAGCGACAAGCTTGTTCAGCAACAA

TAA

**AFSQ01026619**

ATGGCGAATAAATCCCCGGAGACAGAGCACCCTCAGAAGGCCTTCGGATGGGCTGCCAGA

GATAACTCCGGCGTCCTTTCTCCCTTCAATTTCTCCCGCAGGTCGTTTCTCTACTCTACT

GCTCATATATATGTGCCAGCTGTAGCTGCTGGAAACAAAAAGGGGAAGTTGGTGGGTCAT

TACGATATGATTAAATTGGGTCATCTTAAATCATTTTTCAATGGATTATTCTTGATTCTG

GATTTTGGCGGGGAGAATGGAGATGAAGACGTGACCATAAAGATCCATTTCTGTGGGATT

TGCCACTCAGACTTGCACTCCCTCAAGAACGAGTGGGGCTTTTCTCGTTACCCTATGGTT

CCAGGGCACGAGATCGTTGGCATTGTGACGAAATTGGGCTCGAACGTGTCGAAGTTCAAG

GAAGGGGACCGAGTTGGGGTGGGAGTGATGGTTGGTTCATGCAAGTCGTGTGACTACTGT

AACCAGGACTTAGAGAACTACTGCCCGCAGATGGTGTTCACTTACAATGCAACCTACATT

GACGGGACCAGAACCTACGGTGGCTACTCGGACATGATTGTTGTGGACCAGCGGTTCGTG

GTTAGATTCCCCGAAAGCATGCCGTTCGATGCTGGTGCGCCTTTGCTTTGTGCTGGTGTG

ACGGTGTATAGCCCCATGAAGTACTATGGAATGACCGAGGCTGGGAAGCACTTAGGGATT

GTTGGGCTTGGTGGACTCGGACATGTTGCTCTTAAGATCGGTAAGGCGTTTGGTTTGAAG

GTTACTGTCATTAGTAGGTCAGCTGATAAGGAGATTGAAGCTGTTGAGAGATTGGGTGCT

GATGCTTTCCTTGTCAGCAGTGATCCTCTCAAGATCAAGGCTGGGTTTGGTACAATGGAT

TATATAATAGATACAGTTTCAGCAGTTCACTCATTGGCACCATTGTTGGCACTTCTGAAG

CCAAATGGAAAGCTGATTACTTTAGGCTTACCTGATAAGCCGCTTGAGCTTCCAATCTTC

CCTTTAGTTTTGGGTCGAAAGCTGGTGGGAGGAAGTGACATAGGAGGGATGAAGGAGACG

CAGGAGATGTTGGACTTCTGTGCAAAGCATGGAATCACGGCTGATGTTGAGGTGATCCCG

ATCGATCAAGTCAATGTCGCACTGGACAGGCTGGCGAAATCGGATGTTCGGTACCGGTTT

GTCATTGATGTCGCTAACTCCATGTCCAAATAG

**AFSQ01000048**

ATGGCGAATAAATCCCCGGAGACAGAGCACCCACACAAGGCCTTCGGATGGGCTGCCAGA

GACAACTCCGGCGTCCTTTCTCCCTTCAATTTCTCCCGCAGGGAGAACGGACACGAAGAT

GTGACCATAAAGATCCATTTCTGTGGGATCTGCCACTCAGACTTGCACTCCCTCAAGAAC

GAGTGGGGCTTTTCTCGTTACCCTATGGTTCCAGGGCACGAGATCGTTGGCATCGTGACG

AAATCGGGCTCGAACGTGTCCAAGTTCAAGGAAGGGGACCGAGTCGGGGTGGGAGTAATG

GTTGGTTCATGCAAGTCGTGTGACTACTGTAACCAGGACTTGGAGAACTACTGCCCGCAG

ATGGTGTTCACTTACAATGCAACCTACATCGACGGGACCAGAACGTACGGTGGCTACTCG

GACATGATTGTTGTGGACCAGCGGTTCGTGGTTAGGTTCCCTGACAGCATGCCGTTCGAT

GCTGGTGCGCCTTTGCTTTGTGCTGGTGTGACAGTGTATAGCCCCATGAAGTACTATGGG

ATGACTGAGGCTGGGAAGCACTTGGGGATTGTGGGGCTTGGTGGGCTTGGACATGTTGCT

GTTAAGATCGGTAAGGCGTTTGGGTTGAAGGTTACTGTGATTAGTAGGTCATCTGATAAG

GAGATTGAAGCTGTTGAGAGATTGGGTGCTGATGCTTTCCTTCTCAGCAGTGATCCTCTT

AAGATCAAGGCTGGGTTTGGTACAATGGACTACATTATAGATACGGTTTCAGCAGTTCAC

TCATTGGCACCATTGTTGGCACTTCTGAAGCCAAATGGGAAGCTGATCACTTTAGGCTTG

CCTGATAAGCCACTTGAGCTTCCAATCTTCCCTTTGGTTCTGGGTCGAAAGCTGGTGGGA

GGGAGCGACATAGGAGGGATGAAGGAGACGCAGGAGATGCTGGACTTCTGTGCAAAGCAT

GGAATCACAGCTGATGTTGAGGTGATACCGATCGATAAAGTCAATGTCGCAATGGACAGG

CTGGCAAAATCGGATGTTCGGTACCGGTTTGTCATTGATGTCGCTAACTCGATGTCCAAA

TAG

**CHALCONE SYNTHASE**

**AFSQ01023955**

ATGGGCACGATGATCGTCGATCAATTCCGGAAAGAACAGAGGTCGGACGGAGTTGCGACG

ATATTGGCCATCGGTACCGGAACTCCACCGTTCTGCATTGAACAGAGCAGTTTTCCTGAC

TACTACTTCCGCATTACCAACATGGAACACAACACTCAGCTTAAAGACAAGTTCAAACGC

CTCTGTGAAAGGTCCAACATTGAGAAGCGTTACTTTCACCTGACTGAACAATTATTGAAA

GAAAATCCAGATTTTTCATCATACACAGAACCAACATTCAATGCTCGTCAAGATATGGTC

CTGGAGGAAGTCCCAACGCTAGCCCACGAAGTGGCCACAAAGGCCATAAAAGAATGGGCC

CAGCCCAAATCCAAAATCACCCACGTAATATTCTGCACAAGTACCGGAGTGGACATGCCC

GGAGCCGACTACCAACTCACCAAGCTCTTGGGCCTCGAATCGTCCGTAAACCGGACCATG

ATGTACCACCAGGGCTGCTATGCCGGTGGCGCGGCCCTCCGCCTGGCCAAGGACCTGGCC

GAGAACAATCGAGGCGCTCGAGTTCTCGTAGTCTGCTGCGAGATCACCACAATCGCATTT

CGCGGGCCCAGCGACGACGACTTCGCGACCCTCGTGTGTCACGCGTTGTTCGGGGACGGT

GCGGGTGCGGTGATTGTGGGGGCGGACCCGGTACCGGATGCAGAGAGGCCGTTGTTCGAG

CTGATCCGCGGGGCCCAGACTATCGTACCGGATTCGGAAGGGGCGATCGTGGGGAACAGC

CGCGACGTCGGGATGACTTATAAACTAAGAAGGGATGTGCCTAGCCTTGTTGGTAACAAC

ATTGAGAAGTGTCTTGTTGATGCATTTGAGCCATTGGGGGATATTGTTGGTGATCGTGAT

TGGAACTCTTTGTTTTGGGTGGCCCACCCTGGTGGGCCTGCCATACTGGCTAAAGTGGAA

GAGAAGTTAAGGCTCCAGCCCGGGAAGCTACGGGCTTCGAGGCGGGTTATGGCCCAGTAT

GGGAACATGTGGAGTGGGACTGTGTTGTTTGTGTTGGATGAGATGAGGAACAAGTCTGCT

GGAGATGGGCTGAGTACTACTGGAGAAGGGCTTGAATGGGGCGTGCTTCTGGCTTTTGGG

CCTGGTTTGACCGTCGAGACTTTGATTCTTCGTAGTTGTGTAGCTGGAATCTAA

**AFSQ01005163**

ATGGGCACGATGATCGTGGATCGATTCCGGAAAGAACAGAGGTCCGATGGAGTTGCGACG

ATATTAGCCATCGGTACCGGAACTCCACCGTTCTGCATTGAACAGAGCAGTTTCCCTGAT

TACTACTTCCGCATTACCAACATGGAACACAACACTCAGCTTAAAGACAAGTTCAAACGC

CTATGTGAAAGGTCCAACATTGAGAAGCGTTACTTTCACCTGACTGAACAATTATTGAAA

GAAAATCCAGATTTTTCATCATACACCGAGCCCACATTCAATGCTCGTCAAGATATGGTC

TTAGAGGAAGTCCCAAAGCTAGCCCAAGAAGTTGCCACAAAGGCCATAACAGAATGGGCC

CAGCCCAAATCCAAAATCACCCACTTAATATTCTGCACAAGTACGGGAGTCGACATGCCC

GGCGCTGACTACCAACTCACCATGCTCTTGGGCCTCGAGTCGTCCGTAAACCGGACCATG

ATGTACCACCAGGGCTGCTACGCGGGGGGCGCGGCCCTCCGCCTGGCCAAGGATCTGGCC

GAGAACAATAGTGGCGCTCGGGTTCTCGTAGTTTGCTGCGAGATTACCACCATCGCATTT

CGCGGGCCTAGTGACGACGACTTCGCGACCCTCGTGTGTCACGCGTTGTTTGGGGACGGT

GCGGGCGCGGTGATCGTGGGCGCGGACCCAGTACCGGACGTGGAGAGGCCGTTGTTCGAG

CTGGTCCGCGGGGCCCAGACTATTGTACCGGATTCCGAAGGGGCGATCGTGGGGAACAGC

CGCGACGTCGGGATGACTTATAAACTAAGAAGGGATGTGCCTAGCCTTGTTGGTAACAAC

ATTGAGAAGTGTCTTGTTGATGCATTTGAACCATTGGGGGATAATATTGTTGGCGATTGT

GATTGGAACTCTTTGTTTTGGGTGGCCCACCCTGGTGGGCCTGCTATACTGGCTAAAGTG

GAAGAGAAGTTGAGGCTTGAGCCCGGGAAGCTACGGGCTTCGAGGCGGGTTATGGCCCAG

TATGGGAACATGTGGAGTGGGACTGTGTTGTTTGTATTGGATGAGATGAGGAACAAGTCT

GCTGGAGATGGGCTGAGTACTACTGGAGAAGGGCTTGAATGGGGTGTGCTTCTGGCTTTT

GGGCCTGGTTTGACCATCGAGACTCTGATTCTTCGTAGTTGTGTAGTTGGAATTTAA

**AFSQ01007984**

ATGTCGACTATCACCGTGGATGAAGTACGCAAGGCCCAACGTGCACAAGGTCCCGCCACC

GTCCTCGCCATCGGAACCGCCACTCCTGCCAACTGCTTCGATCAAAGCACCTACCCTGAT

TACTACTTCCGTATTACCAACAGCGACCACAAGACTGAACTCAAGGACAAGTTCAAGCGC

ATGTGCGAGAAGTCGATGATCAAGAAACGTTACATGCACTTGACGGAAGAGTTCCTCAAG

GAGAATCCGACCATGTGTGCCTACATGGCAAATTCCCTCGACGCTCGCCAAGACGTCGTC

GTCCCGGAAGTTCCGAAGCTAGGAAAAGAAGCTGCTACAAAAGCCATCAAGGAATGGGGA

CAGCCCAAATCCAAAATCACCCACCTCATCTTCTGCACCACCAGTGGGGTCGACATGCCA

GGCGCCGACTACCAGCTCACCAAGCTGTTGGCCCTCCGCCCGTCGGTGAAGCGCTTCATG

ATGTACCAACAAGGCTGCTTCGCCGGTGGCACGGTACTCCGCCTCGCCAAGGACCTCGCC

GAGAACAACAAGGGTGCACGCGTCCTCGTCGTGTGTTCCGAAATAACCGCCGTCACCTTC

CCCCAGACCATCCTGCCCGATAGTGAAGGAGCTATCGACGGACACCTTCGTGAAGTGGGG

TTGACTTTCCACCTTCTGAAAGATGTCCCCGGGCTGATTTCGAAGAACATTGAGAAGAGC

TTGGTGGAGGCGTTTAAGCCTTTGGGGATATCGGATTGGAACTCGCTTTTCTGGATAGCT

CATCCGGGTGGCCCGGCGATTCTGGACCAAGTGGAGGCTAAATTGAACCTCAAGGAGGAG

AAACTGCGAGCCACGAGGCAGGTTCTGGCTGATTATGGTAACATGTCGAGTGCTTGTGTG

TTGTTCATATTGGATGAGATGAGGAAGAAATCTGCTGCGGATGGGTTGCAGACCACTGGT

GAAGGGCTTGATTGGGGGGTTCTCTTTGGATTCGGGCCTGGCCTTACTGTGGAGACTGTA

GTCCTTCACAGTGTGGCTGTTTGA

**AFSQ01010505**

ATGGCGGGTGCAGGAGGAGCCAAGGTAGTGGAGATCCGAGCGGCTCAGCGAGCGAGTGGA

CCGGCCACGGTTCTGGCCATAGGAACTGCAACCCCGCAAAACTGCGTCATCCAGGCCGAC

TACCCTGACTACTACTTCCGCATCACCAACAGCGAGCATATGTCCGACCTCAAGCTCAAA

TTCAAACGCATGTGTGAAAAGTCGATGATAAGGAAGCGATACATGCACATAACGGAAGAC

TACCTAAAGGAAAACCCAAACATGTGTGCTTACATGGCGCCGTCCCTGGATGCCCGCCAG

GACTTGGTGGTCGTGGAAGTCCCAAAACTCGGGAAAGAAGCCGCCACCAAGGCAATCAAA

GAGTGGGGCCAGCCCAAATCCAAAATCACCCACCTCGTATTCTGCACCACTTCCGGAGTC

GACATGCCAGGCGCCGACTACCAGCTCACCAAGCTCCTCGGCCTCCGCCCTTCCGTCAAG

CGCTTCATGATGTACCAGCAAGGCTGCTTTGCCGGCGGCACCGTCCTCCGCATGGCCAAG

GACCTCGCCGAGAACAACAAGAACGCCCGCGTCCTCGTCGTCTGCTCCGAGATCACCGCC

GTCACCTTCCGGGGACCTTCCGACACCCACCTCGACTCCCTTGTCGGACAGGCTCTGTTC

GGAGACGGAGCCGCCGCTGTCATCGTCGGCGCCGACCCTGACCTGACCGTCGAGAATCCG

ATGTTCGAGCTGGTGTCGGCGGGGCAGACGATACTCCCGGACTCGGGGGGGGCCATCGAC

GGGCACCCGAGGGGAGTGGGGTTGACTTTCCACTTGTTGAAAGACGTGCCCGGGTTGATT

TCCAAGAACATCGAGAAAAGTCTGGTGGAGGCGTTTAAGCCGTTGGGAATCAGTGACTGG

AACTCGCTGTTCTGGATAGCTCACCCCGGTGGGCCTGCTATTCTGGACCAGGTTGAGGAG

AAGCTTGGGCTTAAGCCCGAGAAGATGCGGGCCACGAGGGAGGTGTTGAAAGAATACGGC

AACATGTCGAGCGCGTGCGTTCTGTTTATAATGGATGAGATGAGGAAGAAGTCGGCGGAG

GAAGGGAAGTTGACCAACGGGGAAGGGCTGGAGTGGGGTGTGCTGTTCGGGTTCGGACCT

GGACTCACCGTCGAGACTGTCGTGCTTCACAGCCTTCCCACCGCCAAACAACCGGCGGTG

GTTGGTGGTGCTAATAATGGGAATGGCGTCGTTACTACCGCTGTCGCTGTTAGTAATTAA

**AFSQ01010603**

ATGGCGGCCAGTGAAGGAGCAGCCAAGGTAGCCGAGATCCGAGCGGCTCAGCGAGCTAGT

GGTCCGGCGACGGTTCTGGCCATTGGAACTGCTACCCCCCAAAACTGCGTGATCCAGGCC

GACTACCCCGACTACTACTTCCGCATCACCAACAGCGAACATATGACTGACCTCAAGCTC

AAATTCAAACGCATGTGTGAAAAGTCGATGATAAGGAAGCGATACATGCACATAACGGAG

GACTACCTAAAGGAAAACCCAAGCATGTGCGCTTACATGGCGCCGTCGCTGGACGCCCGC

CAGGACTTGGTGGTCGTCGAAGTCCCAAAGCTCGGGAAAGAAGCAGCCACCAAGGCAATC

AAAGAGTGGGGCCAGCCCAAATCCAAAATCACCCACCTCGTATTCTGTACCACTTCCGGC

GTCGACATGCCAGGCGCCGACTACCAGCTCACCAAGCTCCTCGGCCTCCGCCCTTCCGTC

AAGCGCTTCATGATGTACCAGCAGGGCTGCTTCGCCGGCGGCACGGTCCTCCGCATGGCC

AAGGACCTCGCGGAGAACAACAAGAACGCACGAGTCCTCGTCGTCTGCTCCGAGATCACC

GCCGTCACCTTCCGCGGACCCTCCGACACCCACCTTGACTCCCTTGTCGGACAGGCTCTG

TTCGGTGACGGAGCCGCCGCCGTCATCGTCGGCGCCGACCCTGACCTGGCAGTCGAGAAT

CCGATGTTCGAGCTGGTGTCGGCGGGGCAGACGATACTCCCGGACTCCGAGGGGGCCATC

GACGGTCATCTCCGGGAAGTGGGGTTGACTTTCCACTTGTTGAAAGACGTGCCCGGGTTG

ATTTCCAAGAACATTGAGAAGAGTCTTGTGGAGGCGTTTAAGCCGTTGGGAATCAGTGAC

TGGAACTCGCTGTTCTGGATAGCGCATCCTGGTGGGCCTGCTATTCTGGACCAGGTGGAG

GAGAAGCTTGGGCTTAAGCCGGAGAAGATGCGGGCCACGAGGGAGGTGTTGAAGGAGTAC

GGGAACATGTCTAGCGCGTGTGTTCTGTTCATACGGGGGGGGTGGTGTCATCTCATGTCT

GGGATGCTTTCTTAA

**AFSQ01007986**

ATGGTGAAGGAAAATGCAGGAAGTGCCAGCATCCTTGCCATTGGAACATCAAATCCTTCC

AACTGTGTTCTTCAGAGCAGTTACCCAGATTACTATTTCCGGGTCACCAACGCACAACAC

CGCACCGACCTCATCCCCAACTTCACCCGCATCTGTGAAAACTCGAAAATAAAGAAGCGT

TATCTACATTTAACTGAGCATCTCATTCAAAAGCACCCAAATCTGGCTTCCTACATGGCA

CCTTCTTTGGACATTCGACAAGACATGGTGGTAGGTGAGGTCCCAAAACTAGGAAAACAA

GCGGCCACAAAAGCCATCGAAGAATGGGCCCAACCCAAGTCCAAAATCACTCACTTGATC

TTCAGCACCAGCACTGGCGCCCACATGCCAGGAGCAGATTATCAACTCACCAAACTCATG

GCCCTCGACCCATCCGTCAATCGCTTCATGATCTACCAACAGGGATGCTTCGGTGGCGGC

ACTGGCCTCCGCCTCGCCAAGGACATTGCCGAGAGCAACAGAGGTGCTCGAATCCTCGTG

GTTTGGTCCGAGATAACCACCATCGGGTTTCGTGGACCACCGCCCGATTTCGAATCGGAC

GTCTCCACTCTTGTGGGCCAGGCCATATTCGGGGATGGTGCGGCTGCCGTCATAGTTGGG

TCGGATCCAGATCTCGAGATAGAAAAGCCCTTATTTGAAATTGTGTCAACGACCCAGACT

ATTGTGCCGGATTCGGTTGGAGCGATAGAAGGGTACACTAGAGAAGCGGGGCTGGTGTAT

TTCTTGTCCAAGAGGTTACCGGACCTTGTGTCCAAGAACGTTGAGAAATGCGTGATGGAG

GCGTTCGGGGCTTTGGGGTTGAGTTTAGACTGGAACTCGATATTCTGGGCAGTGCATCCA

GGTGGGCCTAAGATTCTGGACAGGATGGAGATGGAGCTGAATCTCGAAGCTGAGAAGCTA

AGGGCTACAAGGCATGTGTTGGCGGAGTACGGGAACATGTGGAGTGGAAGTGTGATTTTT

GTGCTGGATGAAATGAGAAACAAGTCTATGGAGAACGAGATGGGTACAACTGGAGAAGGG

TTGGAATGGGGAGTGCTGCTTGGATTTGGGCCTGGGCTTACTGTCGAGACTGTGGTTCTA

CGAAGTGTCCCCACCGATTAG

**FLAVANONE 3-HYDROXYLASE**

**AFSQ01016891**

ATGAGCAACACTACTACCACTACTACCCTCCTCCATGACTGGCCGGAACCAATCGTCCGG

GTCCAGTCCTTATCCGACAGCGGCCTCCACGTCATCCCACCTCGTTACATCAAGCCGGCC

CATCAGCGGCCCAATAACCCTCCTCCTCCTCCTCCTCCGTTGACTCCGATCCTAAACAAC

AACAACAATATTAACATCCCGATCGTTGACCTGAGCCACGGCGTGGCGGCGGCGGAGGAG

GAGATATCGGCGGCGTGCAGGGATTGGGGGTTCTTCCAGGTGGTGAACCACGGTGTTAAC

CCGGCGTTGATGGACCGGGTGAGAGAAGCGTGGCGGGAATTCTTCCACCTCCCCATGGAT

GTCAAACAAAGCTACGCTAACTCTCCGAAGACTTATGAAGGGTACGGCAGCCGCCTTGGC

GTCGAGAAAGGCGCCATTCTCGATTGGAGCGATTACTACTTCCTCCACCTCTTGCCGTTG

GCGTTGAAGGACCATCGCAAATGGCCCGCCCGCCCTTCCGACATCAGGGAAGCGATCGAC

GAGTACGGGAAGCAAGTGGTGGAGCTATGCGGGAGGCTGATGAGGATTCTGTCGGCAAAC

CTGGGATTGGAACAGGAACGCCTCCAGAATGCGTTCGGCGGGACCGACATTGGGGCGTGC

TTGAGAGTGAATTTCTACCCGAGGTGTCCGCAGCCGGACTTGACGCTCGGCTTGTCGTCG

CACTCCGACCCTGGAGGGTTGACCGTCTTGTTGCCGGACGACCAGGTTCCGGGGCTGCAG

GTTCGGAGAGGGGAGGATTGGATTACCGTTAAGCCGGCTAAGCATGCGTTTATCGTCAAC

ATTGGCGATCAAGTTGAGGTATTGAGCAATGCTATTTACAAAAGTGTGGAGCATCGGGTG

ATAGTGAACTCGGCGATGGAAAGAGTGTCGTTGGCGTTTTTCTACAATCCGAAGAGCGAC

ATCCCAATCGAACCGGTGAAAGAGCTTGTGACCGAAGAGAGACCCTCGTTGTACCCACCC

ATGACATTTAACGAGTACAGGCTTTACATAAGAACCAAGGGTCCACGTGGCAAGTCCCAA

GTGGAGGCTTTGAGATCTCCAAGGTGA

**AFSQ01025118**

CACGACGATGAGCAACACTACTACCACTACTACCCTCCTCCATCACTGGCCGGAACCAAT

CGTCCGGGTCCAGTCCTTATCCGACAGCGGCCTCCACGTCATCCCCCTCGCTACATCAAG

CCGCCCCATCAGCGGCCCAATAACCCTCCTCCTCCTCCTCCTCCGTTGACTCCCATCCTA

AACAACAACAATATTAACATCCCGATCGTTGACCTGAGCCGCGGCGTGGCAGCGGCGGCG

GAGGAGATATCGGCGGCGTGCAGAGATTGGGGTTTCTTCCAGGTGGTGAACCACGGGGTT

AGTCCGGCGTTGATGGACCGGGTTCGTGAAGCGTGGCGGGAATTCTTCCACCTCCCCATG

GTTGTCAAACAAAGCTACGCCAACTCTCCCAAGATATATGAAGGATACGGCAGCCGCCTC

GGCGTCGAGAAAGGCGCCATTCTCGATTGGAGCGATTACTACTTCCTCCACCTCCTCCCT

TTGGCGTCGAAGGACCACAGCAAATGGCCCGCCCGCCCTTCCGACATCAGTTTAATCGAT

GCCGTACTTGAATGA

**AFSQ01016628**

ATGGTGGAAATCGATCCATCTTTCATCCAAGAAGCTGATCACAGACCACACCCAAACCAC

ACAGAAGCGAAATCGACTGCAAATGAAATCCCAGTAGTCGACCTCTCTTCTCCAGCCGAA

TCCCTAGTTTCTCAGATTGGGGACGCCTGTGAGAAATGGGGATTCTTTCAGGTGATCAAC

CATGGAGTCCCGCTGGAGCTGGTGCGCAGCATGAAGAAAGTTGGGAAAGAGTTCTTCGAC

TTGGCCATGGAAGAGAAGAAGAAAGTGAAGAGGGATGAAGTTCATCCCATGGGGTATCAC

GACAGCGAGCACACCAAGAACGTTCGGGACTGGAAGGAAGTCTTCGATTTCTTGGTGGTT

GATCCGACTTTCGTTCCAGCTACTGAAGTTGCTGAGGATTTGGAGCTCAGAGAATTGACT

AATCAGTGGCCTCAGATGCCTGCTGATTTCAGGGAGGTGTGCGAGGAATACAACAGAGAA

GTTGAAAAGCTAGCATTCAAGCTTCTTGAACTGATTTCCCTGAGCTTGGGCGTGCCTGCT

GATAAACTGAGCAGCTACTTCAAGGACCAAATCAGCTTTTCAAGGTTCAACCACTACCCG

CCATGTCCAGCTCCGGAGCTAGCTCTCGGAGTTGGAAGGCACAAGGACGGCGGTGCCTTA

ACCGTGCTAGCTCAAGACGACGTAGGAGGGCTGGAAATCCGCGAGAGAAGATCCGGGGAA

TGGATCCCTGTCAAGCCTGTTGCTGATGCATTCATCATCAACATTGGCAACTGTATGCAG

GTGTGGAGCAATGACAAGTACTGGAGTGCAGAGCATAGAGTTGTGGTGAACTCCAAAAGG

GAAAGGTTTTCAATACCATTCTTCTTCTTCCCTTCACACTATATCCAGATCAAGCCTATG

GATGAGCTAGTGAATGATGAAAACCCTCCCAAGTACAAGGAGTTCAACTGGGGGAAGTTC

TTCACTTCCCGAAACCGCAGCGACTTCGCGAAGCGCGAGGTCGAGAACATCCAAATCGAC

CATTTCAAGGTGTCAGACTGA

**AFSQ01022073**

ATGGTGGAAATCGATCCATCTTTCATCCAAGAAGCTGACCACAGACCAAACCCAAACCAC

ACAGAAGCGAAACCGACTGCAAATGAAATCCCAGTAGTGGACCTCTCTTCCCCAGCCGAA

TCCCTAGTTTCTCAGATTGGGGACGCCTGTGAGAAATGGGGATTCTTTCAGGTGGTCAAC

CATGGAGTCCCGCTGGAGCTTGTACGCAGCATGAAGAGAGTTGGGAAAGAGTTCTTCGAC

TTGGCCATGGAAGAGAAGAAGAAAGTGAAGAGGGATGAAGTTCATCCCATGGGGTATCAC

GACAGCGAGCACACCAAGAACGTTCGGGACTGGAAGGAAGTCTTCGATTTCTTGGTGGTT

GACCCGACTTTGGTTCCGGCTACTGAAGTTGCTGAGGATGTGGAGCTCAGAGAATTGACT

AATCAGTGGCCTCAGATACCTGCTGATTTCAGTCGAGATCCTGAGCCAAAATTAGGATTT

CCTCGTTCCAGAACCTGGCCTGGATCCGCCATTGTTAGTTGGCATTTTGATCTAATGATG

TTAGAATTTGACTTGGTTCGTTTTGTTAGGGAGGTGTGCGAGGAATACAACAGAGAAGTT

GAGAAGCTAGCATTCAAGCTTCTTGAACTGATTTCCCTGAGCTTGGGCGTGCCTGCTGAT

AAACTGAGCAGCTACTTCAAGGACCAAATCAGCTTTTCAAGGTTCAACCACTACCCGCCA

TGTCCAGCTCCGGAGCTAGCTCTCGGAGTTGGAAGGCACAAGGACGGCGGTGCCTTAACC

GTGCTAGCTCAAGACGACGTTGGAGGGCTGGAAATCGGAGAGAAAAGTTCCGGGGAATGG

ATCCCTGTCAAGCCTGTTGCTGATGCATTCATCATCAACATTGGCAACTGTATGCAGGTG

TGGAGCAATGACAAGTACTGGAGTGCAGAGCATAGAGTTGTGGTGAACTCCAAAAGGGAA

AGGTTTTCAATACCATTCTTCTTCTTCCCTTCACACTATATCCAGATCAAGCCTATGGAT

GAGCTAGTGAATGATGAAAACCCTCCCAAGTACAAGGAGTTCAACTGGGGGAAGTTCTTC

ACTTCCCGAAACCGCAGTGACTTCGCGAAGCGCGAGGTCGAGAACATCCAAATCGACCAT

TTCAAGGTGTCGGACTGA

**AFSQ01009941**

ATGGAAGCTTCACCACATTCCATCCCAGAAACTCACCACGTTGATTTCAGAGCTCCACCC

CCTTCTCCGATCGCGTCAGGGAGGCGATCGTCCTTCACCAACGATGCTGTCCTCACCGAA

TATCTGGAGAACTCATTGCGAGTCCCGGCATTGATACTTCCTGACAGAATATTCCCCAGC

CATAAGATCCTTGAAACACCTCAAGTGGTTGATTTCCGGTCACTGCAAAATGGCGATTTC

ACTCGTCAGTTGGGATGTGATATGGATTCCCTGGCAAGATTGGGGTGCTTCCAACTTGTT

AACCATGGGATTTCTAGTGAATCTGTGAGGTCAGCCATGGATTTGGCTCAGGAAATCTTC

AATGTTCCCGTGGACAAAAGGGCAATGGTGATGGTGTCCACTGAGAAGCCATATGGGTTT

GAGGAGGAGATCATCCAGCAGGATGGTGATGATGATAAGGAAGAGATTATTAGCAATTTG

AGTGAAGAATTTGTTTGGTGTAGAGATGGAAGTTTGAAGTTAGAGATGGAGGCACTTTTG

GGTTATAGCTACTCCAATTTCAGTGTGAAAATGGAAGCTCTGATGTCCAACATAGAGGCA

GTGGGAGAGAGGTTGCTAGGAGTTGTGAGGCAAAAGATGGAGTTGGAAAGTTTGGCAAAT

GATCATCAAAATGAAATGGTGATGCAGCTGGATGAGGAGGAGGAGGATAAGGATCATCAT

GATGATATGGGTCGAGTTGCGGAACAATTGACTTGCTGCTACCTTCGAAAGCACCGGGGA

AATGTTCCGGCAGAGAGGTGGGAGAGATCACTTAGGTATGATGTGATAAAGATGCTGATA

AGAGGCACTGATCACTCTCATGCCTTGTGCTTTCACATTTGTAATGCCTCCTCAGAGTTT

CATGTTTACTCCAAGAAAGGTTGGATTTCTTTCTCCCCTGAGAAAGATGCTCTAGTTGTC

ACAATTGGAGATCAAATTCAGGCTATGAGCAATGGGGAGTTCAGACATGTGGTAGGGAGG

CCAATGGTTAAAAGTGGAGAAGAAGATGACAGTCCTCCTGATAGTAACTCGATTTCAATG

GCGTTTATGTATTCACCTCCACCACCCACCATCAACAGTAGTAGTAACAATCAAATGAAG

GAGGAAAATACTATTTCAATCAAACAACAAGCAATAGTAGCCCTTGTTTTCACTCTTATT

TGCCAAATTTTGGCTTTTCTTTATAAGAATTTTTAA

**AFSQ01023404**

ATGGAAGCTTCACCACATTCCATCCCAGAAACTCACCACGTTGATTTCAGAGCTCCACCC

CCTTCTCCGATCGCGTCAGGGAGGCGATCGTCCTTCGCCAACGATACTGTCCTCACCGAA

TATCTAGAGAACTCGTTGCGAGTCCCGGACTTGATCCTTCCTGACAGAATATTCCCCAGC

CAGAAGATCCTTGAAACACCTCAAGTGGTTGATTTCCAGTCACTGCAAAATGGCGATTTC

ACTCGTCGGTTGGGATGTGATATGGATCGCCTGGCCAGATTGGGGTGCTTCCAACTTGTC

AACCATGGGATTTCTAGTGAATCCGTGATGTCAGCCATGGATTTAGCTCAGTACATCTTC

AATGTTCCCGTGGAGAAAAGGGCAATGGTGATGGTGTCTACAGAAAAACCCTATGGGTTT

GAGGAGGAGATCATCCAGCACGGTGATGATGATGATAAGGAAGAGATTATTAGCAACAGT

TGGAGTGAAGAATTCGTTTGGTGTAGAGATGAAAGCTTGAGGTTGGAGATGGAGGCAATT

TTGGGTGATAGCTACTCCAAATTCAGCACGACCCTTCAAGCGAAAGTCCTCATAGGAGCT

AATGTTTGCACACTTTCGGTAAATTACATACAAGAATTGAGGCTATCGAAATTCGAACAG

AGACTCTGTGTCAAAATGGAAGCTCTGATGTCCAACATAGAGGCAGTGGGTGAAAGGCTG

CTAGGAGTTGTGAGGCAAGAGATGATGAAGTTGGAAAGTCCGGCAAATGATCATCAAAAT

GAAATGGTGATGCAGCTGGATGAGGAGGAGGAGGATAAGGATGACCTGGGTCGAGTTGCG

GAACTATCGAGTTGCTGCTACCTTCGAAAGCACCGGAGAAATGTTCCGGCGGAGAGGTGG

GAGAGATCACTTAGGTATGATGTGATAAAGATGCTGATAAGAGGCAGTGATCACTCTCAT

GCCTTGTGCTTTCACATTTGCAATGCCTCATCCTCAGAGTTTCATGTTTACTCCAAGAAA

GGTTGGATTTCTTTCTCCCCTGAGAAAGATGCTCTGGTTGTCACAATTGGAGATCAAATT

CAGGCTATGAGCAACAGACATGTGCTAGGGAGGCCAATGGTTAAAAGTGGAGAAGAAGAA

GAAGAAGAAGAAGAAGAAGAAGACAGTCCTAACTCGACTTCAATGGCGTTTATGTATTCT

CCTCCACCACCCACCATCAACAGTAGTGGTAATAATCAAATGAAAGAGGAAATTACTATT

TCAATCAAACAACAAGCGATGGTAGCCCTTGTCTTCTCTCTTGTTTGCCAATTTTTGGCT

TTTCTTTATAAATTCTTTTAA

**ISOFLAVONE 2'-HYDROXYLASE**

**AFSQ01007109**

ATGGCGTTCACTCTCTTCTTTGGTTTTCTTGCCTTCATCTCCTTCCTCATCTTCCTTGCT

CGTAATTCAAAACTAATCAACCCCGCCATAAATTCGCGGCCGCCAAGCCCATCTCCTCGT

CCCATCACAGGCCACCTCCACTTGCTAAAGACATCGCCGCTACATCGCGCCCTACACAAC

ATTTCGCAAACATACGGAGACATCTTCTCCCTCCGGTTTGGCACCCGAAACGTCCTTGTA

ATATCCTCGCCATCCCTCGTGGAGGAGTGTCTCACCAAAAATGACGTCACATTCGCCAAT

CGCCCACGCCTAATCACCGGAAAGCACCTCAACTACAACAACACCACCATGGGGTTTTCT

TCCTACGGCGACCACTGGAGAAAGCTTAGGCGTCTGACCACGATCGAGCTTTTTTCCACC

AGCCGGATCAATTCCTTCTCCGGGATTCGAACCGACGAGGTCCAACTGCTGATCAAGAAG

ATTTTCCGAGGCGGGAAGGCGAACAATAAGGTTAATCTGACGGAGAAACTCCTGGAGCTA

ACTTTCAACATCATGATGAGGATGATTGCCGGGAAGAGGTACTACGGCGACGGTGAGGAG

GTGGTGGATGAAGGAGCTAAGGAATTTCAGGACATAATTAAAGAGACGGATGCCCTCCGT

GGAAGCTCCAATATCAACGATTATATACCCATTTTGAAGTGGGTGGATTACGGAGGCGTG

GAGAAGAAGATGATGAAAGTGGGGCAAAAGATGGATAAATTCTTCCAAACCCTCGTCGAC

GAGCAACAACGGCTAACAAGTAATGTCGGAATCGATGAATCGAATCAGCGACGAAAGACC

AACTTGATCGATGTCCTGCTATCGCTTAGAGAAACTGATCTCGAGTTTTACACTGATCAG

ACCATCAAAGGCATAATATTGACGACGGTAACAGCAGGGACTCAAACATCAGCAGCAACA

TTAGGGTGGGCAATGTCGCTTCTGCTCAACAATCCAGAAAAGATGGACAAGGCTTTTTCA

GAGATCGAATCCATCGTCGGATTAGACCATTTGTTCGAAGAATCAGACATTCCAAAGCTG

AAATACTTACACAACATAATCAACGAATCCCTTCGACTGTTTCCCCCAAATCCACTTCTA

CTGCCACACGAATCGTCAACAAAGTGCAAGGTGGGTGGGTTCGATGTGGCAAGTGGCACA

ATGTTGCTGGTGAACACATGGAGCATGAACAGAGACCCTAAGTTCTGGGACGAGCCGGAA

AAATTTGTGCCGGAGAGATTTGAAGGTGGCGGAGGTGGAGGAGAAGGGAATAGCGGTGGT

TTCAAATTGTTGCCGTTTGGGGCCGGAAGGCGTGCTTGTCCCGGAGCAGGCCTGGCCAAA

AGAATAGTGGCGTTGACGTTGGGTGCTCTGATTCAGTCATTTCGATGGGAAAGGGTTGGG

GAGGAGGAGATTGATATGAAAGAAGGAAATGGCCTCACGATGTGTAAAGCTTTGCCGTTG

GAAGCGATTTGTAGGCCACGGGAAGAAATGGTCGATCTTCTCAACTTGCTCTAA

**AFSQ01007112**

ATGGAGTTCACTCTCTTCCTCGGTTTCTTTGTCTTCATCTCCTTCCTCATCGTTCTTGCT

CGTAATTCAAAACTAATCAATCCCGTCAAAAATTCGCGGCCGCCAAGCCCACCTCCTCGT

CCAATCACAGGCCATCTCCACTTGTTAAAGAAATCGCCGCTCCATCGCGCCCTACACGAC

ATTTCGCAGAAATACGGAGACATCTTCTTCCTCCGGTTCGGAACCCGAAACGTCCTCGTA

ATTTCCTCGCCATCCCTCGTAGAGGAATGCTTCACCAAAAACGACGTCACATTCGCCAAT

CGCCCACGCCTAATGACAGGAAAGCACCTCAACTACAACAACACCACCATGGGGTTTTCT

TCCTACGGCGACCACTGGAGAAGCCTCAGGCGTCTAACCACCATCGAGCTCTTCTCCACC

AACCGGATCAATTCCTTCTCCGGGATTCGAACCGATGAGGTCAAACTGCTGATCAAAAAG

ATTTTCCGTGGCGGGAAGGCGAACAGTAAGGTTAATCTGACGGAGAAACTACTGGATCTT

ACTTTCAACGTCGTGATGAGGATGATTGCAGGGAAGAGGTACTATGGCGACGGTAAGGAG

GAGGTGGTGGATGAAGGAGCTAAGGAGTTCCAGGACATAATTAAAGAGATGGATGCTCTC

CGTGGAAGCTCCAATATCAACGATTATATACCCATTTTGAAGTGGGTGGATTACGGAGGC

GTGGAGAAGAAGATGATGAAAGTGGGTCGGAAGATGGATGGATTCTTGCAAAGCCTCGTC

GACGAGCAACTAAGGGTAACAAGTAATGTCGGAATTGATGAACCGAATCAGCAACGAAAG

TTGAACTACTTACACAACATAATCAACGAATCCCATCGACTGTTTCCACCAACGCCACTT

CTTTTGCCACACGAATCTTCAGCAGAGTGCAAGGTGGGTGGTTTTGATGTGCCAAGTGGG

ACAATGTTGCTGGTGAACACATGGAGCATGAACAGAGACCCTAAGTTCTGGGACGAGCCG

GAAAAATTTCTGCCGGAGAGATTTGCAGGCGGAGGTGGAGGAGAAGGGAATAGCGGTGGT

TTCAAATTGTTGTCGTTTGGAGCTGGAAGGCGTGCTTGTCCAGGAGCAGGTCTGGCCAAG

AGAATAGTGACGTTGACGTTGGGTGCTCTGATTCAGTCGTTTCGATGGGAGAGGGTCGGG

GAGGAGGAGATTGATATGAAAGAAGGATCTGGCCTCAGTATGCCTAAAGCTTTGCCGTTA

GAAGCGATTTGTAAGCCACGGGAAGAAATGGTCGACCTTCTCAACTCGCTCTAAACCAAT

TTGATCGATGTCCTGTTATCGCTTAGAGAAACAGACCCCGAGTTTTACACTGATCAGACC

ATCAAAGGCATAATATTGACGACGGTGACAGCAGGGACTCAAACATCAGCAGCAACATTA

GAATGGGCAATGTCGCTTCTGCTCAACAATCCAGACAAAATGAACAAGGCTTTTTCGGAG

ATTGAATCTATCGTCGGATTAGACCATTTGCTCGAAGAATCAGACATTCCAAAG

**AFSQ01000408**

ATGGAGATCAACTACTATCTCTCTATTTCCATTCTCATACTCATCGTAGCTTTCTTATTC

CACCGCTCCAAAAGTATTATTACCCACCGTCGTCGGCCACCATCCCCACCGGGAAGCCTT

CCAATCATCGGCCACCTCCATCTTCTCAAACAACCCCTCCACCGCTCTCTTCACTTCCTC

TCTCACAAACACGGCCCAATCATGTGGCTCAACTTCGGTTCACACCCAGTTGTAGTGGTG

TCATCTTCCGAGGCAGCTGAAGAATGTTTTACCAAAAACGACGTCACTCTGGCCAACCGT

CCCAATCACATGGTGGGGAAGCACATTGGGTACAACCACACTACAATGGTCCAGGCACCC

TACGGGGACCATTGGCGCAACCTCCGCCGCATTGGATCCCTTGAGATCTTCTCCTCAGCC

CGTCTCAACAACTTCTTAGCCATCCGAGCTGACGAGGTCAAGCGCCTCACGCTGCGCCTC

GTATCCTCATCGTCATCCAATAATCATAATAAGGTGGAGCTGAGGGGGGTGCTTCAGGAT

TTAACACTCAACAACATGATGAGGATGATTGCCGGGAAGAGGTACTATGGGGAATCCGAT

GGAGACATGGAAGAGGCGGATCAGTTCAAGGAGATGATGGCTGAGGTTATGGGGATTGGA

TCAGGCAACCCTGCCAATTTCATACCACTCTTGAAATGGGTTGGCTTTGGCGGCTTTGAG

AGGAAGCTGATCCGCCTTTCTAAGAAAATGGACGCTTTCATGCAGAAGTTGATTGATGAT

CGAAGATGCATATTGGAAAAAGACGACCACTCCAAAAACACAATGATCGATCACCTACTG

TCCTTGCAGCAGTCACAGCCAGAGTACTACTCTGACCAAATTAAAGGGATTATACAGATA

ATGCTACTAGCCGGAACGGACACATCGGCGGTGACAATAGAATGGGCGATGTCAAACTTA

CTCAACCATCCAGAAAAGCTAAAGAAAGCAAGAGATGAGATCGACAGCCAAGTCGGGGAA

GAGAGAGTGGTGGAGGAATCAGACCTCTCCAAGTTGAAGTACATTCACAACATAATCTCC

GAAACTCTGCGATTAAACCCGTCAGCTCCGCTACTAGTCCCTCACCGCTCTTCCAAGGAC

TTCACCCTCAGCGGCTACCACATCCCGCAAAACACAATGCTGCTGGTTAACGCATGGGCT

ATCCACAGGGATCCTCGTGTCTGGGAAGATGCTACCACTTTCAAACCTGAGAGGTTTGAA

AATGATGGTGATGATGGGGATAATAAGTATAAGCTGATTCCTTTTGGAGTTGGTCGAAGG

GCTTGTCCAGGGACTGGGTTGGCTCACAGAATGGTTGGATTGACTCTAGCAACTTTGATA

CAGTGCTTTGACTGGGACAAACTTGAAGAGATTGATATGGATGAAGGCAGTGGCCTTACG

ATGCCTAAACTCATTCCTCTTCAGCTCAATTGCAAGCCTCGTCATCCCATTTTCACCGCC

ATCTTTTCTTCCATCAACTCAATCTAA

**AFSQ01019059**

ATGGAGATCAATTACAGCAGCTACATGTCCATTTCCATTCTCCTGCTCATCGTGGCTTTC

ATCTTCCACCGCTCCAAAACTAACATTACCTACCGTCGTCCACCAAGCCCACCAGGCAGC

CTTCCAATCATCGGCCACCTCCATCTTCTCAAACAACCCCTCCATCGTTCTCTTCACTTT

CTCTCTCACAAACACGGCCCAATCATGTGGCTCAACTTCGGTTCACACCCTGTTGTAGTG

GTCTCATCCTCCGAGGCAGCTGAAGAATGCTTTACCAAAAACGACATCACTCTCGCCAAC

CGTCCCAATCACATGGTGGGAAAGCACATTGGGTACAACCACACCACAATGGTCCAGGCA

CCCTACGGGGACCATTGGCGCAACCTCCGCCGCATCGGATCCCTTGAGATCTTCTCCTCC

TCCCGCCTCAACAACTTCCTAGCCATCCGAGCTGACGAGGTCAAGCGCCTCATGTTGCGC

CTTGTATCGTCGTCGTCGTCGTCGAATAATCACAGTAAGGTGGAGTTGAGGGGGATGCTT

CAAGACTTAACGCTCAACAACATGTTGAGGATGATTGCCGGGAAGAGGTACTATGGGGAA

TCCGTTGGAGATAAGGACGAGGCGGATGAGTTTAAGGAGATGATGGTGGAGGTCATGGGG

ATTGGATCAGGCAACCCTGCCAATTTCATACCCCTTTTGAAATGGGTTGGCTTTGGCGGC

TTTGAGAGGAAGTTGATCCGCCTTTCGAAGAGAATGGATGCTTTCATGCAGAAGTTGATT

GATGACCGTAGATGCATATTGGAAGATAAAAACACAATGATTGATCACCTATTGTCCTTA

CAACAGTCACAGCCAGAGTATTACTCTGACCAAATTATTAAAGGGATTATTCAGATAATG

CTACTAGCTGGAACGGATACATCAGCGGTGACAATAGAATGGGCAATGTCAAACTTACTC

AACCATCCAGAAAAGCTCAAGAAAGCAAGAGATGAGATCGACAGTAAAGTCGGGGAAGAG

AGAGCGGTGGAGGAATCAGACCTCTCCAAGTTGAAATACATTCAGAACATCATCTCTGAA

ACTCTGCGATTAAACCCCTCAGCTCCGCTACTAGTCCCCCACCGCTCTTCCAAGGACTTC

ACCCTCAGCAGCTACCATATCCCCGAAAACACAATGTTGTTGGTCAACGCCTGGGCTATC

CATAGGGATCCTCGTGTCTGGGAAGATGCAACCACTTTCAAACCTGAGAGGTTTGAAAAT

GATGATGATGGTGGTGGTGATCATAATAAGTATAAGCTGCTTCCTTTTGGAGTTGGTAGA

AGGGCTTGTCCAGGGACTGGGTTGGCTCACAGAATGGTTGGATTGACTCTAGCAACTTTG

ATACACTGCTTTGACTGGGAGAAACTTGATGATCAAGAGATTGATATGGCTGAAGGCAGT

GGCCTTACGATGCCTAAACTCATTCCTCTTCAGCTCAATTGCAAGCCTCGTCGTCCCATT

TTCAACTCTAAATATATCTAA

**FLAVONOID 3'-HYDROXYLASE/MONOOXYGENASE**

**AFSQ01028139**

ATGGAGAAGATGTATCCGATCACTCTCTCAAGCACCACCACTCTAGCCACCGCATGGGCG

ATCACCGTCGCCGCAATCCTCCTCGCCGGTTGGCTCCGCCGCCGCAGCCGGCGTAATCCA

AACCCGCCACCGGGCCCAACACCCTGGCCCATTATCGGCAACCTCAACCTGATAGGCACA

CTACCCCACCGGTCCCTCCACTCCCTCTCCCAAAAATACGGGCCCATCATGCAGCTCCGA

TTCGGGTCCCACCCAGTCGTCGTCGGGTCCTCCGTCGACATGGCCAAATCCATCCTCAAG

ACCCACGACCTCGCCTTCGCCGGCCGCCCGGAGATTGCCGCCGGGAAGTACACCACCTAC

GAATACTCCGACATCACCTGGTCCCAGTACGGCCCTTACTGGCGGCAGGCGAGGAAGATG

TGTTTAACCGAGCTCTTCAGCGCCAAACGGGTCGGGTCGTACGAATACATCCGGCGGGAG

GAGATGAAATGGGTCGTCAACAACCTTTTCGGGTCGTGCGGAAACCCGATTAACTTGAAG

GACCATTTGTCCGATCTGAGCCTCAATGTGATCAGCCGGATGGTTCTGGGCAAGAAATAC

ACCAATTCCGGCGACGGAGAGAATGAAATTGTGACGCCGGAGGAGTTTAAGGAGATGCTG

GACGAGCTTTTTCTGCTGAACGGTGTGTTGGATATCGGAGATTCGATTCCGTGGCTGGCG

GGGCTGGATCTGCAAGGGTATATAAAAAGGATGAAGGCGGTGGCGAAGAAATTCGATGCC

TTTCTCGAGAGGGTTTTGGATGAGCATAATTCGAGGAGGCGGGTGAAGGATTTTGTGGCG

AAGGATATGGTGGATGTTTTGTTGGAGCTCGCCGCCGATCCTGATTTGGAGGTCAAGCTT

GATCGCCGGGGAGTTAAGGCGTTTACTCAGGCTTGGCAATTCGAACCTTTTAACCGTTTA

ACCGATTATAACCTCATCGAATTTTATGTAAAGGGGAGTGGTTATAACTATTGGAAGCTG

TTACAACCGTTAACCGGTGAATATGGTGATGGTTCTAATTGTATAATCGAAATAAAAATT

GGGATGGTTATGGTTAATAACCATCACGAAAGCGGATGGGATATGATTGCCGGCGGTACA

GAGAGTTCGGCGGTGACGGTAGAATGGGGGATATCGGAACTGTTAAAGAGGCCGGAGATA

TTCGAAAAAGCGACCGAGGAGCTAGACAGAGTAGTGGGGAGAGAGCGTTGGGTCGAAGAG

AAAGACGTGGCCAACCTGTCGTACATCAACGCGATCGTGAAAGAGACAATGAGGCTGCAC

CCCGTGGCGCCACTGTTGGTTCCCAGGATGGCCCGTCAGGATGTCGACATTGCCGGCTAC

CGGGTGGCTAAGGGGACGCGAATATTGGTCAACACTTGGACGATAGGGAGGGATCCGGCC

ATCTGGGACGATCCAGAGGAGTTCCAGCCCGAGAGGTTTATAGGAAAAGAGATTGATGTG

AAGGGTCACGATTTCGAGTTGTTGCCGTTCGGTGCTGGGAGGAGGATGTGTCCCGGGTAT

CCGTTGGGGATCAAAGTGATTCAAGCTAGCTTGGCTAACCTTGTGCATGGGTTCAAGTGG

GGATTGCCAAGTGGGATGAAGAAAGAGGATTTGAATATGGAGGAGATTTATGGACTCTCT

ACTCCTAAGAAGTTCCCACTTGTTGCCGTTGCTCAACCTAGGCTTCCACTTCATCTCTAT

GCCAACTGA

**AFSQ01019454**

ATGTCTACGTCGACGGCCATCATGTGCGACGGCATTATCATCGCCACCGCCATCTACCTC

CTTCTCAGCCTCATCCACCTCCTCCGCCAACGTGGCAGAAAGCCTCTCCTACCGGGCCCC

ACTCCGTGGCCGATTGTGGGGAACCTTCTCCACTTGGGTCCAATGCCCCACCACACTATA

GCCGCCCTGGCTAGGAGGTACGGACCCCTAATATACCTCAGGCTAGGGTACGTGGACGTG

GTGGTGGCCGCCTCGGCCTCGGTGGCTGCTCAGTTTTTGAAGCACAACGACTCCAACTTC

TCCAATCGGCCTCAAACTTCTGGTGGGAAGTACATGGCTTACGGCTTCCAGGACATGGTG

TTCGCCCCCTACGGCCCACGTTGGAAGCTTCTCCGGAAAGTCAGCGCCGTCCATCTCTTC

TCCGGCAAGGCCTTGGATAATTTTAGACACGTCCGACAGAGAGAAGTGACGAGTCTGACT

CGGGCGCTAGCGGGTTCTGGCGGCGCGACAGTGGACCTGGGCTTACTCATAACTTTCTGC

TCCACAAACGCGTTGGGGAAAGCGTTAGTTGGGAAACAAGTGTTCGGGGACATCACCGGC

GGGGGGGGGCCCCCGGCGGAAAAGGTGAAACAATGTGGGGGGGACACCCCCGGCGGTGTC

GACCCTAGGGCGGAAAAGTTCAAGACCATGGTGGTGGAGCTTATGACCTTGGCCGGAATT

TTCAACATCGGCGACTTCATCCCGGCCGTGGAATGGCTAGACTTACAGGGCATTGCTGCT

AGGATGAAGAGTGTCCACAACAGGTTCGACTCCTTCTTGAATCAAATACTTGTGGAATAC

AGGAAGACCGCCCGCGAGGGAGAAAACGTGGACTTTTTGAGTTCGTTGATGTCGTCAAGA

ATTGCGGACGGCCCCGATGGAGTGGAGCGTCAGATCACTGACACTGAAATCAAAGCTTTG

CTTCTCAATATGTTCAGTGCCGGAACCGACACGTCATCAAGTACGGTGGAATGGGCCGTG

GCGGAGCTAATCCGTCACCCGAAAGTCCTGACCCAACTCCAAACCGAGCTGGACACCGTA

GCGGGCCGAGACCGTCTGGTCAACGAACTTGACATCCCTAACCTCCCATACTTAAACGCC

GTCGTAAAGGAGATCTTCCGTTTACATCCACCAACTCCTCTTTCTCTCCCCCGGATGGCC

GCCGAAAGCTGCGAGATCAACGGCCTCCATATCCCGAAAGGTGCAACACTCTTGGTCAAC

ATATGGGCCATAGGCCGCGATCCGGATGTGTGGTCTGACCCGTTGAGGTTCGACCCGGGC

AGGTTCTTGCCCGGTGGGGAGAAGCCCGGAGTGGGTGTGAAGGGGAGCGACTTCGAGCTG

ATTCCATTCGGGGCGGGTAGGAGGATCTGCTCGGGTATGAGCTTGGGGCTCCGGACGGTT

CAGCTGATGACGGCTGTTCTTGCCCATGGGTTTGATTGGGAGCTTAAAGACGGAGTCTCG

GCGGAGGAGCTGAATATGGATGAGGTTTTCGGGATTTCGTTACAACGGGCTGTGCCGCTG

GTCGTGCGGCCAAAAGCACGATTGGCGGAGCATGTTTATCGAGGGTGA

**AFSQ01008368**

ATGGAATCAGCTGTGTCATTAGACAATATGATTGCCCTGGCCTTATTACTTTTCAGCATT

CTTCCCCTTCTAGTATTCATCCTAAAGAATCACTTAAACGTACGCCGCCGCCTGCTTCCT

CCAGGTCCAAGACCATGGCCGATCATCGGCAACCTCAACCTCATCGGACCTCTCCCTCAC

CAGTCCCTCCACAAACTATCCCAAAAATATGGCCCTGTTTTGCACCTCAAGTTCGGCTCC

ATCCCTGTGGTGGTGGTGTCCTCTCCGGACATGGCCAAACAAGTCCTCAAGACCAACGAC

CACCTCCTTGCAGGTCGTCCCGAAACGGCGGCCGGAAAACACACTACTTACAACTACCGC

AACATCACGTGGGCGCCTTACGGACCTTACTGGCGGCAGGGGCGCAAGATATACCTGACC

GAGCTCTTCAGCTCCAAGAGGCTGGCCTCCTACGAGTACATCCGTGTCGAGGAGAGACAC

GCATTTACATCGCGTCTTTTCTCGACAAAAGGACGCGAAGTGGTCCTCAAGGATCACCTT

TCGAGGCTGACGCTGAGTGTTATTAGTAGAATAGTGTTGGGAAAGAAGTACTTTCGTGAC

GGCGATAATGACGACGACAAGGAAGCCGCCATAGTGACCCTGGAGGAGTTTCAAGAGATA

CTGGATGAGTTGTTTTTGCTGAATGGTGTGCTGAACATTGGAGATTGGATACCGTGGCTT

GATTTTTTTGACTTGCAGGGTTACGTCAAGAGGATGAAGGGTTTGAAAGTCAAGCTTGAT

AGGTTTCACGACTTTGTCCTGGACGAGCATCGGAGTAAGAGACATGATCATGTTGGGTCG

TGGAATAAGGACGAGGAAGACATGGTGGATCTTCTCTTGCGACTTGCTGATGATCCTAAT

CTTGATGTCAAACTTGACGATGATAGTGTTAAAGGCTTCTTACAGGACCTCATAGCGGGA

GGAACGGACACGTCAGCGACGACAGTGGAGTGGGCTATGTCAGAGTTGATGAAGCAACCA

AAGCTGATCGAAAAGGCAACAGCTGAGCTGGACGCAGTAGTCGGACGAGAGAGATGGGTT

GAAGAAAGTGATATCCCCAATCTGCCCTTCATCGATGCCATAATGAAGGAGACAATGCGG

AAGCACCCAGTTGCTGTACTGCTAGCCCCTCATTTGGCACTCGACGACTGTACTCTAGGC

GGATACGACGTCGCCAAGGACACCCGAGTTTTCATTAACACGTGGAGCATGGGGAGGGAC

CCTTCCATTTGGGCCGAACCCGAACAGTTCAGCCCGGAAAGGTTTCTTAAGAGTAAGGAT

AGGATTGAGGTGAAAGGGCAGAGTTTCGAGTTGTTGCCGTTCGGATCGGGGAGGAGGATG

TGTCCAGGGTATAGCCTTGGACTCAAAATGATTGCATCAAGCTTAGCTAATTTGCTCCAT

GGGTTCAATTGGAAGTTGGGTGAAGGTGTTACTCCGGACAGTTTGAGCATGGACGAGGTT

TATGGGCTGGCTACTCCTCGAAAGTTCCCACTTGTTGTTATCCTTGGCCCTAGGCTTTCG

CATTCACTCTACCATCAGTGA

**FLAVONOID 3',5'-HYDROXYLASE**

**AFSQ01012300**

ATGGGGTCATGGTGGTGGGCAGTAAGCAGCGATGGATCGCTGCAAGTGGAAGTTGTTATG

GCAGCTCTCGTCATGGTATCGATCCCGGTGATATGGTCATTACTCGAGAGAGCAGCCGAC

GAGAAGTTGCCACCAGGCCCACGAGGATTCCCAATAGTTGGCTACCTGCCGTTTCTTAGC

ACCGAACTCCACAAGAGCTTCAAAGAGCTGGCAGAAGTCTACGGCCCTGTCTACAAGCTC

TGGCTCGGAAGCAAAATGTACGTGGTGATTGGCGCACCAACACTAGCTAAACAAGTTCGA

GAACAGGTCGTAACATTTTCAGATCGTGACCCGCTGATTGCTTCCAAAATCATCAGCTAT

GGTGGCAATGACATCGGTTTCAGCTCACACAGTCCTAACTGGAGAAAGCTGCGCAAGACA

TTCGTTCGCGAACTTTTGTGCAGTGCAAGACTTGAAAGTTCCTATGTGCTGAGAAAGAGG

GAAGTTCAGAAAACTGTTAAGGATGTTTACCAGAAAAAAGGTAAAGCTTTGGACTTTGGT

CAGTTGGTGTTTATGACAGTATCAAACACTGTCCTCAGCATGCTCTTGGGATGCACTGTC

CATGGAGACGAAGGGGTGATCTTCTTTTCGAAGATTCGGAAATTGGTGGATGAATTCAAT

GTGCTACAGTCAACACCAAACATCTCAGACCTAATTCCTGCACTTGCCAGATTTGACTTG

CAGGGGATAGAGAGGAAAACCACACAGGCACAGCAGAGTTTCGATCGAATTCTCGATTCG

ATAATCAATGAGAGGAGGAAGCTGAGAGGTGAAGTGATAGAAACAAAGGACTTCTTGCAG

ATTCTACTAGACCTAAACAGCAATGGAGATGCTGCTTCATCAATTACAGACGACCAACTC

AAAGGGATTCTGGTGGATACGATTATAGGTGGAACAGACACAACAGCAACGACGATAGAG

TGGACGATGGCAATGCTGATGCAACATCAAGACGCAATGCAAAAGATCTACAAAGAACTA

GATGAGGTAGTAGGGAGAAGCAATACTGTCGAAGAGTTGCATCTGCCGAAGCTACGCTAC

TTGGACGCAGTTGTAAAGGAAACATTACGCCTGCATCCAGCGGTGCCTATGCTCGTCATG

CGTTGCCCATCCCAGGACTGCAAACTAAGCGAATACACCATTCCAAAAGGCGTTACAGTC

ATCATTAACGCCTATGCAATCCACAGGGATCCACAGGTCTGGACCAGTCCTCTGGAATTC

CGACCTAACAGGTTCCTAGACAAGAATGCAACCAAGTTCGATTACTTGGGGAATAGTGAC

CGGTATTTCCCGTTTGGGACAGGAAGGAGAGTCTGTGCAGGGCTTCCGATGGCCGAAAAG

ATGCTGAAATACGTACTGGCATCGTTGCTCCACTCGTTTGAATGGAGACTAGCACACGGC

ACAGAGGTGGTGGATTCGACAGATAAGTTTGGAATTGTTTTGAAGTTAGAGAAACCATTG

ATTCTTGTTCCTAGGCCGAAGAAAGGAGCGAAACTAAATTTCACCCGCAATCAGATGAAG

GATCAGAATGCATTGAAAAGGTCCAATCCGACAACAGCACAAGCTCCTGCCAAACGGCCC

CAAATCCAGTTCATCCAAACGAACTCTAGCAATCGCGCTGAGCTCAAACTTGCCAGACGA

GGAAACAGCTCACTTCCGCGAACGGATCATCGTTTCCAATCTGCTCCCAGCTTCCTTTTG

CTCGTCTCTCCTCCTACACGCAGAATGAAATATCACCGCCGTTCCAGCTTGCGTTCCTCT

CTTTCTCTCTGA

**AFSQ01025777**

ATGGATTTGCTCGTCGTGGTGGTTTCTATCCTCATCGTGGTATCGATCCCGATGTTATGG

TCATTACTCAAGAGAGCAGCCGACGAAAAGTCGCCACCGGGTCCACGAGGGTTGCCTATA

GTTGGCTACTTACCATTTCTCAGCACCCAACTCCACAAGAGCTTCGCGGAGCTGGCTGAA

CTCTACGGCCCTATCTACAAACTATGGCTCGGAAGCAAAATGTACGTAGTGATCGCCTCG

CCAAAACTGGCTAAACAAATCCGAGACCAGGTCGTAACGTTTACAGATCGCGACCCGGTC

ATTGCTTCAAAGATCATCAGCTACGGTGGGAGTGATGTCGCTTTCTGCACAAACGGTCCT

GACTGGAGAAAGCTGCGCAAGGTTTTCGTGCACGAATTGTTGAGCAGTTCGAGGCTCGAA

AGCTCGTACGTGCTGAGAAAGAGGGAAATCGAAAAGGCTGTTAAGTATGTGTATGGAAAA

AGAGGTAAAGCTTTGGACTTTGGCCGGTTGGTGTTTATGACAGTATCAAACACTGTCCTG

AGCATGCTCTGGGGAAGCACTGTCTATGGAGAAGAGAGGAAGATGTTGTTTTCGGAGATA

CGGAAATTGGCAGATGAATACAATGTGCTAATGTCATCACCAAACATCTCGGACCTGATT

CCTGCACTAGCCAGATTTGACTTGCAGGGGATAGAGAGGAGAACCAGAGAGGCACAGCAG

AGTTTCGATCAGATTCTGAGTTTCATAATCAATGAGAGGAGGAAGCTGGGAAGTGAAGCG

AACGAGACAAAGGACTTCATGCAGATTCTACTAGACCTAAACAGCAATGGAGATGCTGCT

GCTTCAGTTACAGACGACCAACTCAAAGGGATTCTTGTGGACGTTATTATCGGAGGAACA

GACACAACATCAACGACAATAGAGTGGACAATGGCAATGCTGATGCAACATCCAGATGCA

ATGCAAAAGATCTACAAGGAACTAGATGAGGTAGTAGGAAGAAGCAACACTGTCGAGGAG

TTGCATCTGCCGAAGCTACACTACTTGGACGCAGCTGTTAAGGAATCGCTCCGCCTGCAT

CCGGCAGTGCCTATGCTGGCACTACGTTGCCAATCTCAGGACTACAAACTAGGGAAATAC

ATCGTTCCCAAAGGAGTCACAGTCATCGTCAACACCTACGCAATCCAAAGGGATCCACAG

CTCTGGACCAGTCCGCTAGAATTCAGACCCGAAAGGTTCCTAGACATGAATGTTACAGCC

AAGTTCGATTACTTGGGCAGCAGTTCGCAATATTTCCCGTTTGGGACAGGGAGAAGGGTC

TGCGCAGGGCTTCCAATGGCGGAGAAGATGCTGAAGAACGTTCTGGCATTGTTGCTCCAC

TCGTTCGAATGGAGACTGGCACAGGGGACAAAGGTGGATTCGACAGAAAAGTTTGGAATT

GTTTTCAAGTTAGCGAAACCATTAATTCTTGTCCCTATACCGAAGTTAGCCAATCCAGAT

CTGTACTAA

**AFSQ01012299**

ATGGATTCGCTCGTCGTGGTGCTTTCTATCCTCGTCGTGGTATCGATCCCGGTGTTATGG

TCATTACTCAAGAGAGCAGCCGACGAGAAGTCGCCTCCGGGTCCACTAGGGTTGCCTATA

GTTGGCTACTTACCATTTCTCAGCACCCAACTCCACAAGAGCTTCGGGGAGCTCGCTGAA

CTCTACGGCCCTATCTACAAGCTCTGGCTCGGAAGCAAAATGTACATAGTGATCGCCTCG

CCAAAACTGGCTAAACAAATCAGAGACCATGTCCTAACGTTTACAGATCGGGACCCGCAG

ATTGCTTCCAAAATCATCAGCTATGGTGGGAGTGACGTAGCCTTCTCCACGTACGGTCCT

GACTGGAGAAAGCTACGGAAGGTTTTCGTGCATGAACTGCTGAGCAGTTCGAGGCTCGAA

GGTTTGAACTTGCTGAGGAAGAAGGAAGTTGAGAGCGGTGTTAAGGTTGCGTATGAGAAA

CGAGGCAAAGTTTTGGACTTTGGGGAGTTGGTGTTTATGACAGTATCAAACACTGTCCTG

AGCATGCTCTGGGGAAGCACTGTGAATGGAGAAGAAGTGGAGATGTCGCTTTCGGAGATT

CGGAAACTGGCAGATGAATACAATGTGCTAATGTCATCACCAAACATCTCGGACGTGATT

CCGGCGCTAGCTAGATTTGATTTGCAGGGGATAGAGAGGAAAACCAGAAAGGCTCAGCAG

AGTTTCGATCGGATTTTGAGTTCCATAATCAATGAGAGGAGGAAGCTGGGAAGTGAAGTG

AAAGAGACCAAGGACTTCTTGCAGATTCTACTAGACCTAAACAGCAATGGAGATGCTGCT

GCATCAATTACAGACGACCAACTCAAAGGGATTCTTCTGGACGTTATTATCGGGGGAACA

GACACAACATCAACGACGATAGAGTGGACGATGGCAATGTTGATGCAACATCCCGATGCA

ATGCAAAACGTCTGCAAAGAACTAGATGAGGTAGTAGGAAGAAGCAACAATGTTGAGGAG

TTGCATCTGCCAAAGCTACGCTACTTGGACGCAGCTGTTAAGGAATCGCTCCGCCTGCAT

CCGGCAGTGCCTATGCTGGCATTACGTTGCCAGTCCCGGGACTACAGACTAAGCAAATAC

ATCGTTCCAAAAGGAGTTACAGTCATCGTCAACACCTTCGCAATCCAAAGGGATCCACAG

CTCTGGGCCAGTCCGCTAGAATTCAGACCCGAAAGGTTCCTAGACGAGAGTGCTACAGCC

AAGTTCAATTACTTGGGCAGCAGTTCGCAATATTTCCCATTTGGAATGGGAAGGAGGGTC

TGTGCAGGGCTTCCGATGGCAGAGAAGATGCTGAAGTACGTTCTGGCATCGTTGCTCCAC

TCGTTCGAATGGAGACTAGCGAAGGGGACGAAGGTGGATTTGACGGAAAAGTTTGGAATT

GTTTTCAAGTTAGAGAAACCGTTGATTCTTGTCCCTACGCCGAAGTTGGCCGACTCAGAT

**LEUCOANTHOCYANIDIN DIOXYGENASE**

**AFSQ01011747**

ATGAACTGCGTCCAGAAATGGCCCGAACCCGTAACCCGAGTCCAGTCCCTGTCCGAGAGC

GGCATCCGAAAGATCCCCGACCGTTACGTCAGCAGGCCCCACCACCTCCCTTCTCTCACT

CTCAACATCAACAAACCTCACGCCGCACCACCGCCGGCCACGTCCAACATCCCGGTGATC

GACCTGGCGGAGGCGTTCGGCGGCGACCCGGCGGCCAGGGCGGAGACCATGAGGCGGCTA

TCCGCGGCCTGCAAGGACTGGGGGTTTTTCCAGGTGGTCAACCACGGAGTTGACCTCGAG

CTGATGACGAAGATTAGGGAGATTTGGCGGGAGTTCTTTAACCTCCCGCTCGGGACGAAG

CAGATTTACTCAAACGACCCCGTTTCGTACGAGGGTTACGGCAGTCGGTTGGGGGTCGAG

AAGGGGGCCGTCTTGGACTGGAGTGATTACTTTTTCCTTCATTACCTACCGGCGAGGATC

CGGAACCCAACCAAGTGGCCCGCGTTCCCTGATTCTTGCAGGGAATTGGTGGGTGAATAT

GGAAAAGAGGTTGCAAAGCTAGGAGGAAAACTAATGAAGATATTCTCGAGCAATCTTGGC

CTTGAAGAGGACGGTCTCCAAGACGCCTTCGGCGGGGAAGACTTCGGTGGATGCTTAAGG

GTTAATTTCTACCCGAAATGCCCTCAGCCGGATCTCACCCTAGGTCTCTCCCCTCACTCC

GACCCCGGTGGCATGACCATCCTCTTGCCTGACGAAAATGTCTCCGGACTCCAGGTCCGT

AAATCCGGCAATTGGGTTACTGTTAAGCCCGTCGCCAATGCCTTCATCATCAACATCGGC

GACCAGATTCAGGTGTTGAGCAATGCGATCTACAAAAGCGTGGAGCACAGGGTGATAGTG

AACTCGGCCAAGGATCGAGTCTCCCTCGCGTTCTTCTACAATCCAAGGGGCGACCTTCAG

ATCGCACCGTCGAAGGAGCTAGTGAAGGCCGATCAACCAGCGCTTTACCCGTCAATGACG

TATAATGAGTACAGAATGTTCATCAGGACTAAGGGACCGTGTGGAAAGCAACAAGTTGAA

TCTTTCAAATCAACCTGTACTGGTAGTACTGGTACCAGTACTAGACGAAGTTAG

**AFSQ01021358**

ATGAACTGCGTCCAGAAATGGCCCGAACCCGTAACCCGAGTCCAGTCCCTGTCCGAGAGC

GGGATCCGAAAAATCCCCGACCGTTACGTCAGCAGGCCCCACCACCGCCCTTCTCTCACT

CTCAACATAAACAAACCTCAAGCCCCACCGCCGCCGCCGCCGGCCGCGTCCAACATCCCG

GTGATCGACCTTGCGGAGGCGTTCTGCAGCGACCCGGCGGCCAGGGCGGAGACCATGAGG

AAGCTTTCCGCGGCCTGCAAGGACTGGGGGTTCTTCCAGGTGGTCAACCACGGCGTTGAC

CTTGAGCTGATGAGGAAGATTAGGGAGATTTGGCGGGATTTCTTTAACCTCCCGCTCGGG

ACGAAGCAGATTTACTCAAACGACCCCGTTTCGTACGAGGGTTACGGCAGCCGGTTGGGT

GTCGAGAAGGGGGCCGTCTTGGACTGGAGTGATTATTTTTTTCTTCATTACCTCCCGGCG

AGGATCCGTAACCCGACCAAGTGGCCCGAGTTCCCCGATTCTTGCAGAGAATTGGTGGGT

GAATATGGAAAAGAGGTTGCAGGGCTAGGAGGAAAACTAATGAAGATATTCTCGAGCAAC

CTTGGCCTCGAAGAGGACGGTCTCCAAGACGCCTTCGGCGGGGAAGACTTCGGCGGATGC

TTAAGGATTAACTTCTACCCGAAATGCCCTCAGCCGGACCTAACCTTAGGCCTTTCACCT

CACTCCGACCCCGGTGGCATGACCATCCTCTTGCCCGACGAAAACGTCTCTGGTCTCCAG

GTCCGTAAATCCGGCAATTGGGTCACCGTTAATCCCGTCGCCAATGCCTTCATCATCAAC

ATCGGAGACCAGATTCAGGTGTTGAGCAATGCGATTTACAAAAGCGTGGAGCATAGGGTG

ATAGTGAACTCGGCCAAGGATCGAGTTTCCCTGGCGTTCTTCTACAACCCACGGGGCGAC

CTTCAGATCGCACCGTCGAAGGAGCTAGTGAAGGCAGATCAACCGGCGCTTTACCCGTCA

ATGACGTATAATGAGTACAGATTGTTCATCAGGACTAAAGGACCGTGTGGAAAGCAACAA

GTTGAATCTTTCAAATCGCCTTATACTAGTAGTAGTACTGATACTAGTACCAGACGAAGT

TAG

**AFSQ01012389**

ATGGCGGCTTCGAGAGCTTCAGAAGTCGAGAGCTTGGCAAGCAGTGGGCATCCCAAAGAG

CAGAATCAAATGGCTGCTTCGGTAACTTCAAGAGTCGAGAGCTTGGCAATCAGCGGGATA

GTATCAATCCCGAAAGAGTACATCAGGCCGGGAAACGAGCTAAAGAGCATCGGAGACGTG

TTCGAGGAAGAGAAGAATCAAGAAGGGCCACAAGTTCCCACCATTGATTTGAAAGGGATG

GACTCGGAAGACATTGTAGTTCGCGAAAAGTGCAGGGCGCAGCTTATCGATGCAGCAAAG

AAGTTAGGCGTTATGCACCTGATCAACCATGGGATTCCTGAAGATCTCATCGAATCTGTG

AAGAAATCAGGGGAGGAGTTCTTCAACCTTCCTGTGGAGGAAAAGGAGAAGTATGCGAAT

GAACAAGCTTCGGGGAAGATTCAAGGGTATGGAAGCAAGCTTGCTAACAATGCTAGTAGC

CAGCTTGAATGGGAAGACTATTTCTTCCACCTTGTCTTCCCTGAGGAACAAAGGGACCTC

TCCATCTGGCCGAAGAACCCCGCTGACTACACCAAGGTGACTAGTGAATATGCAAGGCAG

CTGAGAAAGCTAACGACCAAAATCCTGTCAGTTCTGTCTCTTGGCTTGGGACTAGAAGAA

GGAAGGCTAGAGAAAGAAGTCGGAGGGATCGAAGAGCTTCTGCTTCAAATGAAGATCAAC

TACTACCCGAAGTGTCCACAGCCTGAACTGGCCCTCGGTGTGGAAGCTCATACCGACATC

AGTGCCCTAACTTTCATCCTCCACAACATGGTCCCAGGTCTGCAACTCTTCTATGAGGAA

AAATGGGTGATTGCCGAATGTGTCCCGAACTCCATCATCATGCATATCGGGGACACTCTC

GAGATCCTGAGCAATGGAAAGTACAAGAGCATCCTCCACAGGGGACTTGTTAACAAGGAG

AAGGTCAGAATCTCATGGGCGGTCTTTGCCGAGCCGCATAAGGAGAAGATCATACTCCAG

CCGTTGCCAGAGGTAGTCACTGATGCAGAGCCTGCACTGTTCCCTCCTCGTACTTTTGCT

CAGCATATCGAGCACAAGGTGTTCAGGAAGAGCCAGGAAGATGCCCTTCTGGCCAAATGA

**AFSQ01026255**

ATGGCAGCTTCGGTAGCTTCGAGAGTCGAGGGCTTAGCAAGCAATGGGTCTCCGACACAA

CAGACTCGAATAGTGGCTTCGGTAGCTTCAGAAGTTGAGAGCTTAGCAAACAACGGTCCT

CCCACACGGAAGACTCAAATAGTGGCTTCAGTAGCTTCAGAAGTCGATAGCTTAGCAAGC

AATGGGCATCCCAAAGTGCAGAATCAAATGGCGGCTTCAATAGCTTCTGAAGTCGAGAGC

TTAGCGAGCAATGGGCATCCCAAAGAGCAGAATCAAATGGCGGCTTCGGTACCTTCACGA

GTCGAGAGCTTGGCGAGCAGCGGGATCGTATCGATCCCGAAAGAGTACATCAGGCCGGAA

AACGAGCTAAAGAGCATTGGAGACGTGTTCGAGGAAGAGAAGAATCAAGAAGGGCCCCAA

GTTCCCACCATTGATTTGAAAGGGATGGACTCGGAAGACATGGTAGTCCGCGAGAAGTGC

AGGGCGCAGCTTATTGATGCAGCAAAGGAGTTGGGCGTTATGCACCTGATCAACCATGGC

ATTCCTGACGATCTCATCGAATCTGTGAAGAAAGCAGGGGAGGAGTTCTTCAACCTTCCT

GTAGAGGAAAAGGAGAAGTATGCGAATGATCAAGCTTCGGGGAAGATTCAAGGGTATGGA

AGCAAGCTTGCTAACAATGCTAGTGGGCAGCTTGAATGGGAAGACTATTTCTTCCACCTT

GTCTTCCCTGAGGAACAAAGAGACCTCTCCATCTGGCCGAAGAACCCTGCTGACTACACC

AAGGTGACTAGTGAATATGCAAGGCAGCTGAGAAAGCTAACGACCAAAATCCTGTCAGTT

CTGTCTCTTGGCTTGGGACTAGAAGAAGGAAGGCTGGAGAAAGAAATCGGAGGGATTGAA

GAGCTGCTGCTTCAGATGAAGATCAACTACTACCCCAAGTGTCCACAGCCTGAACTAGCC

CTCGGTGTGGAAGCTCACACCGATATCAGTGCCCTAACTTTCATCCTCCACAACATGGTC

CCTGGTCTGCAACTCTTCTATGAGGACAAATGGGTGATTGCGAAATGTGTCCCGAACTCC

ATCATCATGCATATCGGGGACACTCTCGAGATCCTGAGCAATGGAAAGTACAAGAGCATC

CTCCACAGGGGACTTGTTAACAAGGAGAAGGTCAGAATCTCATGGGCGGTCTTTGCCGAG

CCGCATAAGGAGAAGATCATACTCCAGCCATTGCCAGAGGTGGTTACTGATGCAGAGCCA

GCACTGTTCCCTCCTCGCACTTTCGCTCAGCATATCGAGCATAAGGTGTTCAGGAAGAGC

CAGGAAGATGCCCTTCTGGACAAATGA

**ANTHOCYANIDIN REDUCTASE**

**AFSQ01027810**

ATGGCCACACCTACCACTGTCGTCGAGCCTCCCCCGGGAAAGAAAACTTCATGTGTGATA

GGAGGCACCGGCTTCGTGGCGTCTAGGCTGATCAAATTGTTCTTGGAAAACGGCTACTCA

GTTCGGACCACTGTCCGGAACCCTGACAACCAGAGGAGGATTTCACACCTGATACCGCTG

AAAGAATTGGGGGAGCTGGAGATTTTCGCAGCAGAGTTAACTGACGAGGAGAGTTTCATC

TCTCCGGTGGCAGGGTCCGACTTGCCAGCAATCCAAGGAGTGGAAAACGTGATGAAAGCT

TGCGCCAAGGCCAAAACGGTGAAAAGGGTGGTGCTTACTTCCTCTGCAGCAGCAGTTACT

ATTAATAATGTTGAGGGAACCGGTTTGGTCATGGACGAGAATCACTGGACCGACGTTCAC

TTTCTAACTTCAGACAAGCCTCCAACTTGGGTATACAGAGCACATTTGTTTGTTGCGGAG

AAGGAATCTGCTTCTGGCCGATTCATATGCTGTGCTGCCAATACTAGTGTCGCTGAGCTT

GCCAAATTCCTCAGCTTGAGATATCCACAGTACAAAGTCCAGCCCCAGATTGGAGATGTT

CCGGAACAGGCGAAGCTGACGATTTCTTCAGATAAGCTGATCGGAGAAGGATTCAAGGAC

TGGAGGATACAGCTTGTAAAGTGA

**AFSQ01023196**

ATGGCCCAGTCAATTGCTTCATCGATGTGTGGCTCCACCACCCGATTGAACATAGCTACT

ACCGCTGGCCGACTACCCGCCGTGGCCCGCCCTAGTCTCACGGTTCGAGCCCAACAGCAG

ATTGCAGTTGGTGAGACTAGCCGACGGTCCATGATAGGCCTCGTTGCTGTTGGGTTGGCT

TCTGGTTCTTTCGCTCAGGCTGTGCTCGCCGAGGCCAATGCAATCAGGGTTGGACCGCCT

CCGCCTCTCTCCGGCGGCCTACCGGGGACGGAGAACTCGGACCAGGCGAGGGACCTGGAT

TTGCCACTAAAGGAGAGGTTCTACCTACAGCCACGTACCCCAGAAGAAGCAGCAGCAAGG

GTGAAAGTGTCGGCTAACGAGATAGTGAACGTGAAGAGTTTCATCGACAAGAAGGCCTGG

CCATATGTGATGAACGACCTTCGTCTTAGGGCATCGTATCTTCGCTACGACCTCAAAACC

GTGATCTCAGCCAAGCCTAAGCCAGAGAAACAAGCCCTCAACGACCTCACTGGAAAACTT

TTCCAGAGCATCAACAACTTGGATTATGCGGCGAAGATCAAGAACAGTGCTCAGGCAGAG

AAGTATTATGCTGAGACTGTATCTAATTTGAATGATGTTCTGGCTAAGATTGGATAG

**UDP-GLUCOSYLTRANSFERASE**

**JN088353**

ATGGCGACCAAGAAGAAGAAACCACATGTTCTTCTGGTTCCACATCCAGCACAAGGTCAT

GTCTTCCCAATGCTGAAGCTGGCACATAAGCTTACCGACCATGGAATCAGCGTCACCGTT

GCTAATTTGGATTTCATCCACCGCAAGATTATACCTCAGCAACAACAGGGGAAGCAGAGC

CATGGTACTGATGGTGGTGGTATCAGAATGGTTTCGCTTCCTGACGGATTGGGTTCCCAT

TCTGACAGCATCGACGTAGTGTTGCGTACGGAGACCGTGCAGAAAGTACTTCCAGTTCGA

TTGCGGGAGCTGCTGATTCAGCAGCAGTCTCTAAGCAATGATGATGAAGAACAAAAGTTC

AGTTGGATCATTGCCGATGCATGCCATTTTGGAGTGTTTATTGTTGCCAGGGAAATGGGG

ATCAAAACGGTTGCGCTTTGGACCGCCTCGCAGGAGAATTTGGCTCTAGTGTTGCGTATT

CCTCAGCTGATCGAGACCGGCACTATCAATGAAAACGGTAATCAACTACACATTAATTTC

ATATTTTTTAAATGCTTGGAGTTTTTCAATTTTTCTTTGTTCTTGCAGGATTTTTAGTAG

ACAAAGAGTTGCCGATTTCGATATCTGAAGAGATGGTTGCTTGGAAAGCTAACGAGTTAC

CATGGAGTGCTCCATCTGAAGAACTCCAGTCCTTTTATTTCAAGAACTGTTATTCGAAGC

CATCGGAGCACTGTTCGCTTTACCACCACGTCATAGTCAACTCATTTCATGAACTGGAAC

CGTCAGCTTTTCAACTGTTTCCAAACTTCCTCCCAATAGGTCCTTTGGTTATAAATTCAG

CTAATTCAGGAGGAAGCTTTTGGCGTCAGGATGAAACTTGCCTGACCTGGCTCGACAACC

ATCCTTCGAAATCAGTCATATACGTCGCATTTGGAAGCATCACAATTCTGAGTCAAAAAC

AGTTTCAGGAACTTGCATTGGGACTTGAACTGGCAGGAAGACCATTCCTCTGGGTGATAA

GAACGAATTTCGTGCAAGGACCAGGAGGGTCAGGTCTGGAATTTCCAAATGGGTACCTCG

AAAGGGTTGCGAATATGGGGAAAATAGTGGAGTGGACGAATCAAGCACGAGTGCTTTCTC

ACCCTTCCGTTGGGTGTTTCGTGAGTCATTGCGGATGGAACTCCACACTGGAAGGGTTGT

GGTGTGGAGTTCCGTTTCTGTGCTGGCCTTACTTTTTGGATCAGTTCCATAACAAGGAGT

CTATATGTGAAGCTTGGAAGGTTGGCCTGAAACTGAAGGCTGAAGAAGATGGAAGTGGAC

TGATCACCATGTCTGAAATTGCTAGCAAGGTTGAACAACTTCTAAATGATGAAACCATAA

AAGGCAATGCAAATAGGTTGAGGGAAGTTGCTAGGGAAAGTGTGAATCAGGGTGGCTCTT

CTTTTCACAGTTTCTCGAGTTTCGTCAACCAATTATGTTCTAATGATGTGGCTTGTGAGT

AG

**JN088354**

ATGGCGGCTAAGAAGAAACAAGTAGATGTGCTTCTGGTTCCACATCCAGCACAGGGCCAT

GTCTTCCCAATGCTCAAGTTAGCACAAAAGCTAACCGACCATGGCATCAGTGTCACCGTC

GTTAATTTCGATTTCGTCCACCTAAAGATTGTGCCAGAGGAGCAGAGCAATGGTGGGAGT

GGGATCAAGCTGGTTTCAGTTCCTAACGGATTCGGATCCGATTTCAACGACTCCAATCCG

ACGATGATAACTGATTGCGTGGAGAAAGTACTTCCAGTTCACTTGCGGAAGTTGCTGATT

GATGAGCATCAGCAGGAGTTCAGTTGGGTCATTGCTGATGCATTCCTTTCTGCGGCTTTT

GTGGTTGCTAAAGAAAAAGGGATCAGAACTACGGCGTTTTGGACCGCATCAATGGAGAAC

TTGGCTTCGATTTTGCGTATTCCTCAGCTGATCCAGGATGGTACTATAGATGAAAACGGT

AATTAATCAATCAAAGTTTTTATTTTCAGAGCAGAGCAAGGTTCAAAAAATTAATGCTTG

TGAAGATTCGATTTCTTTGCAGGATCTTTAATAAACGAAGACCTGCCGATTTCCCTGTGT

CGGGAAATCCCATCCTGGAAAGCAAACGAATTGCCATGGAGCTGTCAACCTGACGAAATT

CAGTCGTTCATGTTCAGACGCTACTACGTGAACCCAGCAAAGTACTTCGCATTGTTCGAC

TGCTTCATCGTCAACTCGTTCCACGAACTCGAGCATTCGGCTTTCCAATTGTACCCCAAC

ATCCTCCCAATAGGTCCACTGGTCACAAATTCCACTTCAATAGGAAGCTTCTGGCGCCAG

GATCCAACTTGCTTGACCTGGCTTGACAAGCATCCACGGAGGTCGGTCATATACGTTGCA

TTCGGAAGCATCTCGGCCCTGAATCCGCGACAATTCCAGGAGCTAGCAATGGGTCTAGAA

ATGACAGGGAAACCTTTCCTTTGGGTGATCAGGGCAGGTTTCGTGAAGGGGGTGTTAGGG

TCGTCAGAATCTGATGTTGAATTTCCTGATGGGTTTCTGGAGAGGGTTGCGAACCGTGGG

AAGATTGTGAAGTGGTCGAATCAAGCTGAGGTGCTTTCTCATCCTTCTGTAGCGTGTTTC

GTTAGCCACTGCGGGTGGAACTCGACGCTGGATGGTTTGTGGTCTGGAGTTCCGTTCCTG

TGCTGGCCGAACTTTACGGATCAGTTTCATAACACGGAGTCCATATGTAAAACTTGGAAG

GTTGGGATGAAACTTAAGGTTGAAGGTGATACTGGATTGATCACGATGTTGGAAATTGCA

AGCAAAGTTGGAGAAATGTTTGACGATGAGTCCATCAGAGATAATGCAAATGGGTTGATG

GGAATGGCCACGGAGAGTGTGAATGAAGGTGGCTCTTCATTTTGTAATTTCCAGAAGTTT

ATTAACAAATTGTGCTCTTAA

**JN088355**

ATGCCGGCGACCAAGAAGAAACCACATGTGCTTCTGGTTCCACATCCAGCACAAGGCCAT

GTTTTCCCCATGCTCAAGTTGGCACATAAGTTGACCGACCATGGAATCAGTGTCACTGTC

ACTAATTTGGATTTCATTCATCGCATGATTATAGCAGAGGAGCAGCAGGTCCATGGTGGG

ATCAGGCTGGTTTCACTTCCTGATGGATTCCGTTCCAATTCCGACAGCAGTGATCATAGA

ATGTTCACAGAGGCTGTGAAGAAAGTACTGCCAATTCAAATACGGGAGCTGCTGATGAAT

CAGCAGCAGTCTCAAAGTAATGATGAAGAGCAAGAGAAGTTCAGTTGGGTGATTGCCGAT

GCTTTCCTTTCTGGAGTGTTTATTGTTGCCAAGGAAATGGGGATCAAAACGGCTGCGCTT

TGGACTGCCTCGCTGGAGAATTTCGCTCTGATGCTTCATATTCCTCAACTGATTGAGGCT

GGCACTATTGATGAAAACGGTAATCAAAATTTCGTACCAGCGTTTTCAATCAGCTAATCA

TATTTTTAGAGTTTTCAAAAAAGTCTTTTCTTTTCTTGCAGGATTCTTAATAGAGAAAGA

GTTGCCGGTTTCGATATATAACGAGATGCTTGCCTGGAAAGCTAACGAGTTGCCGTGGAG

TTATCAACCTGAAGAACTCCAGCCCTTTCTTTTCAAAAACTACTATGCCCAGCCATCCAA

GCACTGTTTGCTTTTCGACCACGTTATATTCAACTCGTTTCATGAGCTCGAACCATCAGT

TTTTCAATTGTTCCCACATTTCCTCCCAATAGGTCCATTGGTTACAAATTCAACTAATTC

AGGAGGAAGCTTTTGGCATCAGGATGAAACTTGCCTGGCCTGGCTCGACAAGCATCCTCC

AAAATCAGTAATATACATTGCATTTGGAAGCATCGCAGTTCTAAGTCAACAGCAGTTTCA

GGAACTTGCATTGGGTCTTGAACTGACAGGAAGACCATTCCTGTGGGTGATCAGAACAGA

TTTTGTGCAAGGGTCAGGTCTGGAGTTCCCATATGGATACCTGGAGAGGGTTTCGAATCG

AGGGAAGATAGTGGAGTGGACGAATCAAGAGCAAGTGCTTTCTCATCAATCCATAGCATG

TTTCCTAAGCCATTGCGGATGGAACTCGACGTTGGATGGGTTGTGGTCTGGAGTTCCATT

TTTGTGTTGGCCTTTTTGTTTTGATCAGTTCCGTAACAAGGAGTCTATATGTGAAGCTTG

GAAGGTTGGCCTGAAGTTGGAGGCTGAAGATGGAACTGGGTTGATCACCATGTCTGAAAT

TGCTAGCAAGGTTGCAGAACTTCTTATTGATGATACCATAAGAAATAATGCAAATAAGTT

GAGGGAAGTTGCTCAGTCGAGTGTGAACAAGGATGGCACTTCTTTCCACAATTTCTCGAG

TTTCATCGATAATTTGAGTTCTTAG

**JN088356**

ATGGCGACCAAGAAGAAACCACATGTGCTTCTGGTTCCACATGCGGCACAAGGCCATGTC

TTCCCAATGCTCAAGCTGGCACATAATCTGGCCGACCATGGTATCACCGTCACCGTCGCT

AATTTGGATTTCATTCACCAAAAGATTGCGCCACAGGAGCAGCAGCAGGGCCATGGTGGT

ATTGGTATCAAGCTGGTTTCACTTCCTGACGGATACAATTCTGATTTCGACATCAGTGAT

GTAGTGAGGTTTACAGATTCTGTGCATAAAGTACTGCCGGTTCAATTGCGGGACCTTCTG

ATTCAGCAGCAGTCTCTAAGTAATGATGATGGTGAAGAGCAAGAGAAATTCAGTTGGGTG

ATTGCTGATGCATTCCTTTCTGGAGTGTTTGTTGTCGCCAAGGAAATGGGAATCAAAACG

GTTGCGCTTTGGACTGCCTCGCTGGAGAATTTCGCTCTGATGCTGCGTATTCCTCAACTG

ATTGAGGCTGGCACTATTGATGAAAACGGTAATCAAATTTCAGACTAGCATTTTCAATCC

GTTATTCATTTCAATCCAGCTAATCATGCATACATATAAATGCTTGGAATATTCATTTTC

TTTTTCCTTGCAGGATTTTTAACAGACAAAGAGTTACCGATTTCGATCTCTGACGAGATA

CTTGCTTGGAAAGCTAACGAGTTACCATGGAGTTGTCAATCTGAAGAATTCCAGTCCTTT

CTTTTCAAGAACTTCTATACCCAACCATCCAAGCACTGTTTTCTTTGCGACCACGTCATA

ATCAACTCATTTCATGAACTGGAACCGTCAGCTTTCCAATTGTTCCCAAACTTCCTCCCA

ATAGCTCCATTAGTTACAAATTCAACTAATTCAAGAGGAAGCTTTTGGCGTCAGGATGAA

ACTTGCCTGACCTGGCTCGACAAGCACCCTCCGAAATCAGTAATATACGTTGCATTTGGA

AGCATTGCAGTTCTCAGTCAACAGCAGTTTCAGGAACTTGCATTAGGCCTTGAACTGGCA

GGACGACCATTCCTCTGGGTGGTCAGGACAGATTTTGTGCTAGGGTCGGGTCTGGAGTTC

CCAGATGGATACCTAGAGAGGGTTGCGAATCGTGGGAAAATGGTGGAGTGGACGAACCAA

GAGGAGGTCCTTTCTCACCCTTCCGTAGGGTGTTTCCTGAGCCATTGCGGATGGAACTCT

ACGCTTGATGGGTTGTGGTCTGGAGTTCCGTTTCTGTGTTGGCCTTACTTTGTGGGTCAG

TTTCATAACAAGGAGTCCATATGTGAAGCTTGGAAGGTTGGTCTGAAATTGGATGTTGAA

GAAGATGGAACTGCAGGATTGATCACCATGTCGGAAATTGCTAACAAGATTGAACAACTG

TTTAATGATGAAATCATAAAAAGTAACGCGATTATGTTGAGGGGACTTGCTCGGGCTACT

GTGAACAAGGATGGCACTTCTTTCCGCAGTTTCATGAGTTTTGTCGATAATTTGTGTTCT

TAG

**JN088357**

ATGGCGACCAAGAAGAAACCACATGTTCTTCTGGTTCCACATCCAGCACAAGGTCATGTC

TTCCCAATGCTCAAGTTGGCACATAAGCTGACCGACTACGGAATCAGCGTCACCGTCGCT

AATTTGGATTTCATTCACCGCAAGATTGCGCCAGAGGAGACGACGTCCAAGGAGCAGCAG

CAGGGCCATGGTACTGGTATCAGGCTGGTTTCACTTCCTGACGGTAACGGTTCTGATTTC

GACATCAACGATGTAGTGAAGTTTGTAGAGACAGTGCACAAAGTACTGCCGTTTCAATTA

CGGGAGCTGCTGATTCAGCAGCAGTCTCTAACTCTAAGTAATGATAAAGAGCAGGAGTTC

AGTTGGGTGATTGCCGATGCATTCCTTTCTGGAGCGTTTGTAGTAGCTAAGGAATTGGGG

ATCAAAACGGCCGCGCTTTGGACTGCCGCCATGGAGAATTTCGCTCTGATGCTACGTATT

CCTCAACTGATTGAGGCTGGCACTATTGATGAAAACGGTAATCAACTGGACATTAATTTC

ATATTTTTAAATGCTTGGAGTTTTCAATTTTTCTTTCTTCTTTCAGGATTCTCAACAGAC

AAAGAGTTGCCGATTTCGATCTCGGAAGAGATACTTGCTTGGAAAGCTAACGAGTTACCA

TGGAGCGTTCAACCTGAAGAGCGCCAAACCGTGTTTTTCAACACCTCCTATACACATCCA

TCCAAGCACATCTCGCTTTTCGACCACGTCATTGTCAACTCATTTCATGAACTTGAACCT

TCAGCTTTTCAACTGTTTCCAAACTTCCTCCCAATAGGTCCTTTGGTTACAAATTCAACT

AATTCGGGAGGAAGCTTTTGGCGTCAGGATGAAACTTGCCTGACCTGGCTCGACAACCAT

CCTTCGAAATCAGTCATATACGTCGCATTTGGAAGCATCACAATTCTGAGTCAAAAACAG

TTTCAGGAACTTGCATTGGGACTTGAACTGGCAGGAAGACCATTCCTCTGGGTGATCAGA

ACAAATTTTGTGCAAGGTCCACCAGGAGAGTCGGGTCTAGAGTTCCGGAGGAAGCTTTTG

GCGTCAGGATGAAACTTGTCTGCCCTGGCTCGACAACCATCCTCCGAAATCAGTCATATA

CGTCGCGTTTGGAAGCAAGGCAGTTCTGAATCAACAGCAGTTTCAGGAACTTGCATTGGG

CCTTGAACTGGCAGGAAGACCATTCCTCTGGGTGATCAGAACAAATTTTGTGCAAGGTCC

ACCAGGAGAGTCGGGTCTAGAGTTCCCAGACGGGTACCTCGAAAGGGTTGTGAATATTGG

GAAAATAGTGGAGTGGACGAATCAAGAGCGAGTGCTTTCTCACCCTTCCGTAGGATGTTT

CCTAAGTCATTGCGGATGGAACTCCACACTGGAAGGGTTGTGGTGTGGAGTTCCATTTCT

GTGCTGGCCTTACTTTTTGGATCAGTTCCATAACAAGGAGTCTATATGTGAAGCCTGGAA

GGTTGGTCTGAAACTGAAGGCCGAAGAAGATGGAACTGTTGGAGGATTGATCACCATGTC

TGAAATTGCTAGCAAGGTTGAACAACTTCTAAATGATGAAACCATAAAAGGCAATGCAAA

TAGGTTGAAGGAAGTTGCTAGGGGAACTGTCAATCAGGGTGGCTCCTCTTTTCACAATTT

CTTGAGTTTCGTCAACCAATTACGTTCTACTGATGTGGTTTGTGAGTAG

**JN088358**

ATGGCGGCGATGAAGAAGAAGCCTCATGTTCTTCTTGTCCCATACCCAGCACAAGGCCAC

GTCGTTCCCATGCTTAAGCTGGCACAGAAGCTAGCAGACGACCACGGCTTCACTGTCACG

GTCGTTAATTTGGAGTTCATACACCAAAAGCTCGTTTCGGATGCAACGATATCGGAGCAC

CAGAGCATCAGCCTGACTGCGATTCCCAACGGATTCGAACTCTCGTCCGTGTCCGGGCAG

GCTGAGAGTGTAACCAAGATAATGGAGAACGTAGAAAATGTTCTGCCAATTCACTTAAGA

ACCCTACTTGATGTGAAGAAGAATAAGAGGAACAAGTCTGCAGCTGGAGATATCACTTGG

CTCATCGGAGATGCATTTCTGTCGGCAGGGGCGTTTCAGGTGGCCAAGGAAATGGGGATC

AAAACGGCTGCGTTTTGGACCGGCTCCGCGGCTACCTTGGCCTTGCTACTGCGTATCCCA

CAGCTGATCCAGGATGGAATTCTCGATGAAAACGGTAAGAATGATTTAACTAAAAAGCAA

TCTGCAGGAGAGATAGAGTCGTTCAAGATTGTATATAAAACTTGGTTAATCGATTAACCG

AATTAACCGACCATGGTAGTAAGTTTCGGTAAAAATAGGTGTAAGTATGAAAAGAGTGGG

TTAAACATTAGTTATCAAATGATGAACCGAATAACGGGATCAATTATTCACATTAACTGA

TTAACTATCGAATTTGGCCTTTTAATATAGTCAATTCCGGTTAAAAAGGTACAAATATGA

AAAGAGTCGGCTAAACATTAGTTTCAGCTAATAAAAACTCAAATCATTAATCGAATAACC

AAACGAGTTATTCAAATTAACTGATCGACAACCAAAGTTGGTCGGGTAATCGATTTTAAC

CGTCGAAGGTTAATGTCCATCCTAATCTGCAGAGGGTTCAATTTGCTAGTCCTATATTAA

AACAATTCTCAGCTTTGTATCAGAAATCACAGCTTCAAAATTTTGACAGCAGGAACATTG

ATAAACCGCGGTATGCCAATTTGCCTTTCAAAGGACATCCCTGCATGGCAACCTGACGAA

TTCCCATGGAGCTGTCAACCTGAACAATTTCAGAGATTTGGTTTCAAGGCCTTCTCCAGC

AAGCCATCTGAGAATTCTACACTTTTCGACTGCTTCATCGTCAACTCACTCTACCAACTC

GAGCCTGCAGCTTTCCAATTGTTTCCTAAACTCCTCCCAATAGGTCCATTGGTTACAAAT

TCAACTTCCGGAGGAAATCAGCATAACCAGATTCCAGGAAGCTTTTGGCATCAGGATCAA

ACCTGCTCGACCTGGCTCGATAAGCATCCTCCGAAATCGGTCGTATACGTAGCGTTCGGA

AGCACCACTGCTCTAAACCAGAAGCAATTTCAGGAGCTAGCCACGGGTCTCGAAATGACG

AAAAGACCTTTCCTTTGGGTCATCAGGTCAGATTTTGTGAACGGGACAGGATCATCAGGA

CAGGAATTTGTAGACGGTTTCCTCGAGAGGGTTGCGAATCGGGGGAAGATTGTGGAGTGG

GCGAATCAAGAGGAGGTTCTTTCTCATCGTTCTACGGCATGTTTCGTGAGCCACTGCGGA

TGGAACTCCACGTCGGACGGACTGTGGAATGGAGTTCCGTTCCTGTGTTGGCCTTACTTC

AGTGATCAGTTTCATAATAGGGAGGCGATATGTGAAGCTTGGAAGGTTGGTTTGAAACTA

AAGGCTGAAGATGAAGATGGGTTGGTTACAAGGTTTGAGATTTGCAGCAGAGTTGAAGAA

TTGATTTGTGACGCCACCATAAGAGAAAATGCCAGCAAGCTTAGGGAGAATGCCAGAGAG

TGTGTGAGTGATGGTGGCACTTCTTTCAGGAATTTTCTCAGTTTTGTTGAAATTTTGTGC

TCCTAG

**JN088359**

ATGGCGGCAATGAAGAAGCCTCATGTTCTTCTGGTGCCATACCCAGCGCAAGGCCATGTT

ATTCCGATGCTGAAGCTGGCGCAGAAGCTAGCAGACCATGGCTTCAATATCACGGTCGTT

AATTTTGAGTTTGTCCACCAAAAGCTCGTTTCGTCGCCCGAGCATCAAAGCATCAGGCTC

ACTGCAATTCCCTTCGAGCTGGAACCCGGATTGGGGCAAGATGATGCAGTGACCAAGTTG

ACAGAGAGCATAACAAATGCACTGCCAATTCACTTGCGGAATCTAATTCATCAGATGGAG

CAAGAGATCACTTGGGTCATTGGTGATGCACTCCTGTCAGCTGGGGTGTTTCAGGTTGCT

AAGGAACTAGGGATCAAAACGGCAGCGTTTTGGACGGCCTCCATGGAGAACTTGGCATTC

TTACTGAGTATTCCGCAGCTGATCCAGGACCGAATCATAGATGAAAAAGGTAGTAATGAT

TTAAACATAAAGCTAGGTAAGGTATTTAGAAGGTACCGGTATCCTTTATATCATATTTTT

ATTATATTTTGTAGTTAAAATTAAGAGTTTTAAGGTTGTATGTTTTGTGTTCAGGATTTT

GGAATAATATTAGGGATTTCGAGTTTACTTTTTGAGGTTTAGGATTTATTAATTGTGATT

TATGGACAAAAGTTATAATATAGAGGTACCGGTACATCTTAATGTAAAGGTTTTATTTGT

GAACAGAAATCATTCATTGCCTTCAAAATATTGGTTGCAGGAACCTTAATAAACAGCAGT

TGGCCAGTTTGCCTGTCAAAAGACATCCCTTCCTGGCAACCTAATGAGCTCCCGTGGAGC

TGTCAACCTGAAGAATTTCAGAGATTTATTTTCAAGAACTACTCCCTAAAGCCGTCTCAA

AATTCTGCACTTTTCGACTGCTTCATCGTCAACTCCTTCCACCAACTCGAGCCGACAGCT

TTTCGAATGTTCCCCAAAATCCTGCCAGTAGGTCCACTTGTTATTACAAACTCCACTTCA

GGAGGACATCACCAATATAGCCAGGTTCCAGGAAGCTTTTGGCATCAGGATCAAACTTGC

GAGACCTGGCTCGACAATCAGCCTCCGAGATCAGTCATATACGTTGCATTCGGCAGCATC

GCTGTTCTAAACCAGAAGCAGTTTCAGGAACTGGCGTGGGGTCTTGAAATGACAAAAAGG

CCTTTCCTTTGGGTGATCAGAGCAGATTTTGTGAACAGAACAGGATCATCAGGTCTGGAA

TTCCCATATGGGTTCCTCGAGAGGGTTGCGAATCGGGGGAAGATTGTGGAGTGGGCGAAT

CAAGAGGAGGTGCTTTCTCATCGTTCTACGGCATGTTTCCTGAGCCATTGCGGATGGAAC

TCCACGTTGGATGGACTCTGGTGTGGAGTTCCATTCCTGTGCTGGCCTTATTTTACTGAT

CAGTTTCATAACAAGGAGTCGATATGTGAAGCTTGGAAGGTTGGTTTGAAACTAAAGGCG

GAAGATGGAAATGGGTTGGTTACGAGGTTTGAGATTTGCAGCAGAGTTGAAGAATTGATT

GGTGATGCCACCATGAGAGAAAATGCCAGCAAGTTTAGGGAACAGGCCAGAGAGTGTGTT

AGTGAAGGTGGCAATTCTTTCAGAGGTTTTCTTAGGTTTGTTGAAACTTTGTGCTCATAG

**JN088361**

ATGGCCAAGCAACCTCATGTCTTGGTTGTGCCACTCCCGGCACAAGGCCATCTACTCCCT

CTCATGAAGTTGGCGCGCAAATTAGCCAACCGCGGGATCAACGTCACAGTTATGAACTTG

GAGACCATACACCGCAAAATCATTCACGCAATGCCAACTCGAGTGAGGCTCGTTGGAGTT

CCTGATGGCCTGGAGCTCGATCACCGCCACGATCTGGTCAAGCAAATGGAGTGTTTGGAA

AGAGTGATGCCGGGTCAGTTGAGGAGCCAGCTGGTTGAGGGGGAGGTGGTTTGTGTGATT

GCGGATGTGTCCCTGGCATGGGCGTTCCATGAGGCTAAGGCGATGGGGACCAAAACGGCT

GCGTTTTATCCTGCTAGTGCTGCCACCTTGTCCTTGTTGCTGGATATTCCACGTCTTCTT

CAGCTTAGGATTTTAGATCATGATGGTGAGTGTGAATTCTGTGAAGATTGAGTGTGAATT

CTGTTCCTTCTGTTTTCTGACTCAAAACAATGTGGGTTGTTAATAGGAGTTGGATTAACA

GAATCATCCATTGGAATGGCGAAAGAAATCCCAAGCTGGGAGGCCAACGAGCTCCCCTGG

AGCCATCCTGCATATACCGACGAGCTGCGAAAACTGTCATTCCAATCTTGCTGCTTCAAC

GTCAGAGAATGTTCTCAAAATTCCGACTACATGTTGGTGAATTCATCCCAAGAACTCGAG

CCATCAGCTTTTCGATTGATTCCCAACGCCTTCCCTATAGGCCCGCTGCAAATCAGTACT

GACATAGACCCTGACGACGATACTGATAATTCAGTCCTTGTAGGAAGCCTGTGGCCTGAA

GACCAAACTTGCTTAACATGGCTCAACATGCAAGACCAGGGCACAGTCATTTACGTTGCA

TTTGGGAGCATCGCGACAATAGAAAACCAGCAACAATTCGCTGAATTGGCAATAGCCCTG

GAATTCACTGGCAATCCCTTTCTATGGGTGGTCAGGCCTGGCGGATCAGGTACATTTATC

AATAGCCAGCTATTCGAAAGTTACTAGTAAGGCAGTAATTAAATTTATTTCAAGGTTACT

TTTCTTAATGCAGAGTTCCCAGACGGGTTCCTGAAAAGGGTCGGGGATCGAGGCAAAATA

GTGGAATGGGCAAATCAAGAGGAGGTGCTTTCGCACCCTTCAATTGCATGTTTTGTGAGC

CATTGCGGATGGAACTCTACATTGGACGGCTTGGTAGCCGGAGTTCCGTTCCTGTGCTGG

CCTTTCTGTTTTGATCAGTTCCACAACAAGAAGTACATTTGTGAAACTTGGAAGATTGGT

TTGGAATTGAAGGCTGAAAATGGGACGGATGTAGGCATCATTACGAATGCAGAAATTGTG

AGGAAGCTCGATGAGTTGCTTTATGATGACACCATAAAATCCAATTCAATGAAGCTTCGG

GAAATAGCTAGAGATGCTACTTGTGGTAGTACTACTGATACAGGGTCTTCATTCCTCAAG

TTTGAAACATTTGTCACTGAGTTGTGTAACACCTTATGCAAATGTCATATCGACAACGTA

CAAGAGATATCCAAGGACTATAAGAGACAATTAAACCTTAACGGACAAGATGTGTGTTGA
