# Supplementary material for: Methyl Salicylate Level Increase in Flax after Fusarium oxysporum Infection Is Associated with Phenylpropanoid Pathway Activation
Source: Front Plant Sci. 2017 Jan 20;7:1951. doi: 10.3389/fpls.2016.01951 (PMC5247452; doi:10.3389/fpls.2016.01951)
Supplement: Supplementary file 4 [file Image1.pdf]

A

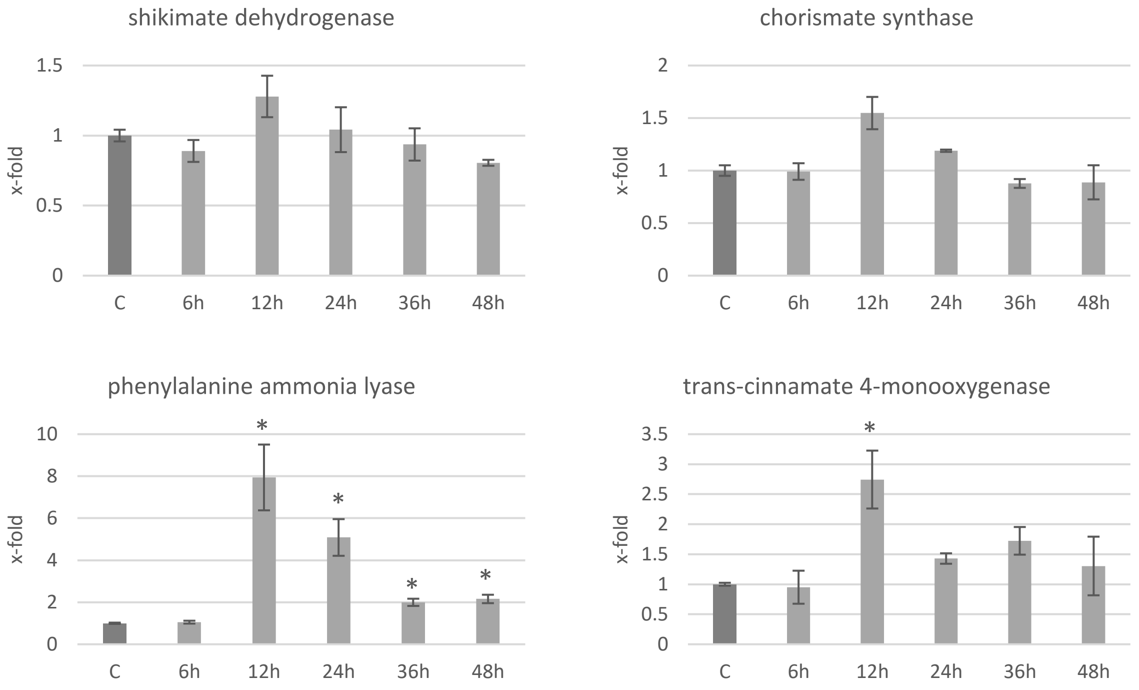

shikimate dehydrogenase
chorismate synthase
phenylalanine ammonia lyase
trans-cinnamate 4-monooxygenase
x-fold
C
6h
12h
24h
36h
48h
*

B

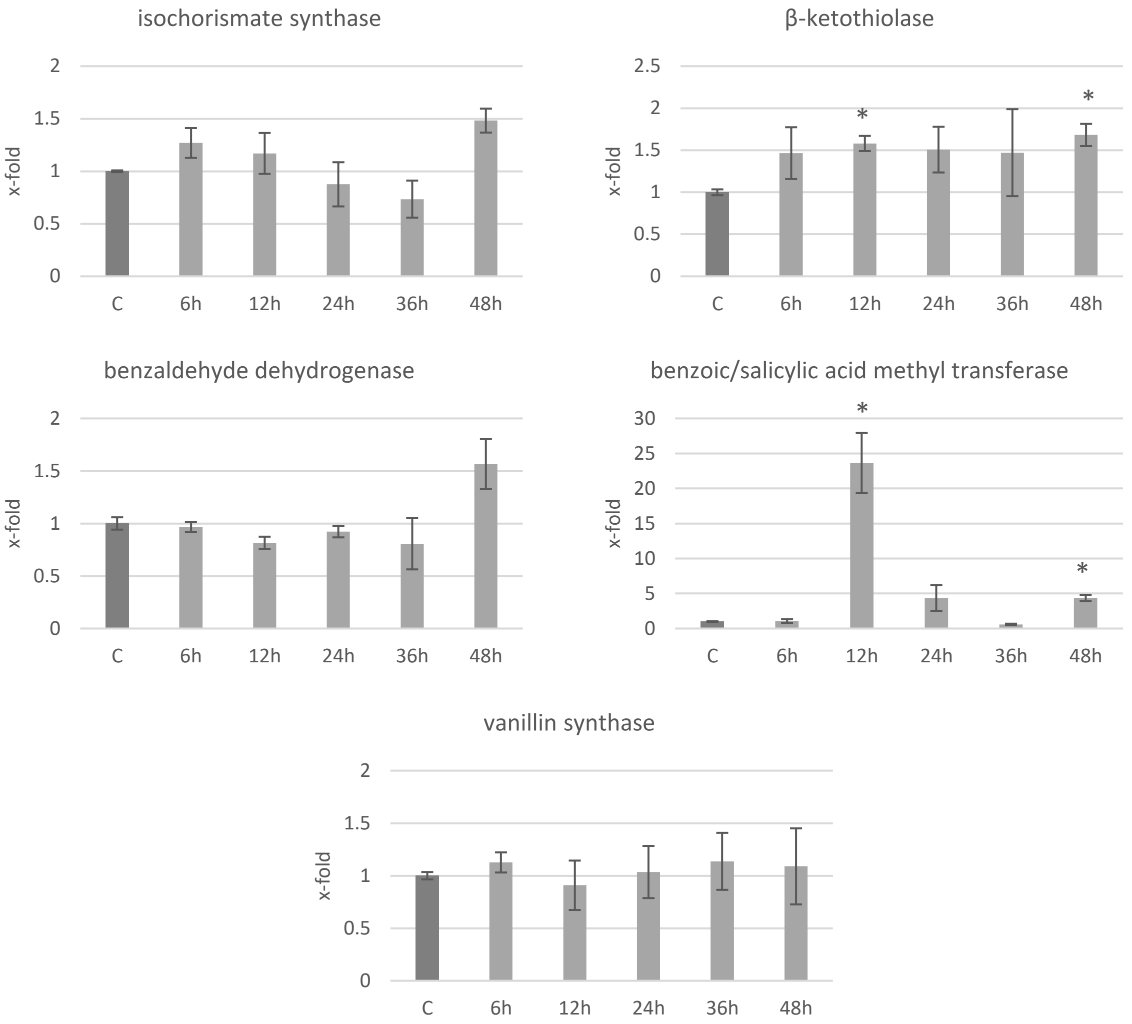
isochorismate synthase
β-ketothiolase
benzaldehyde dehydrogenase
benzoic/salicylic acid methyl transferase
vanillin synthase
x-fold
C
6h
12h
24h
36h
48h
*

C

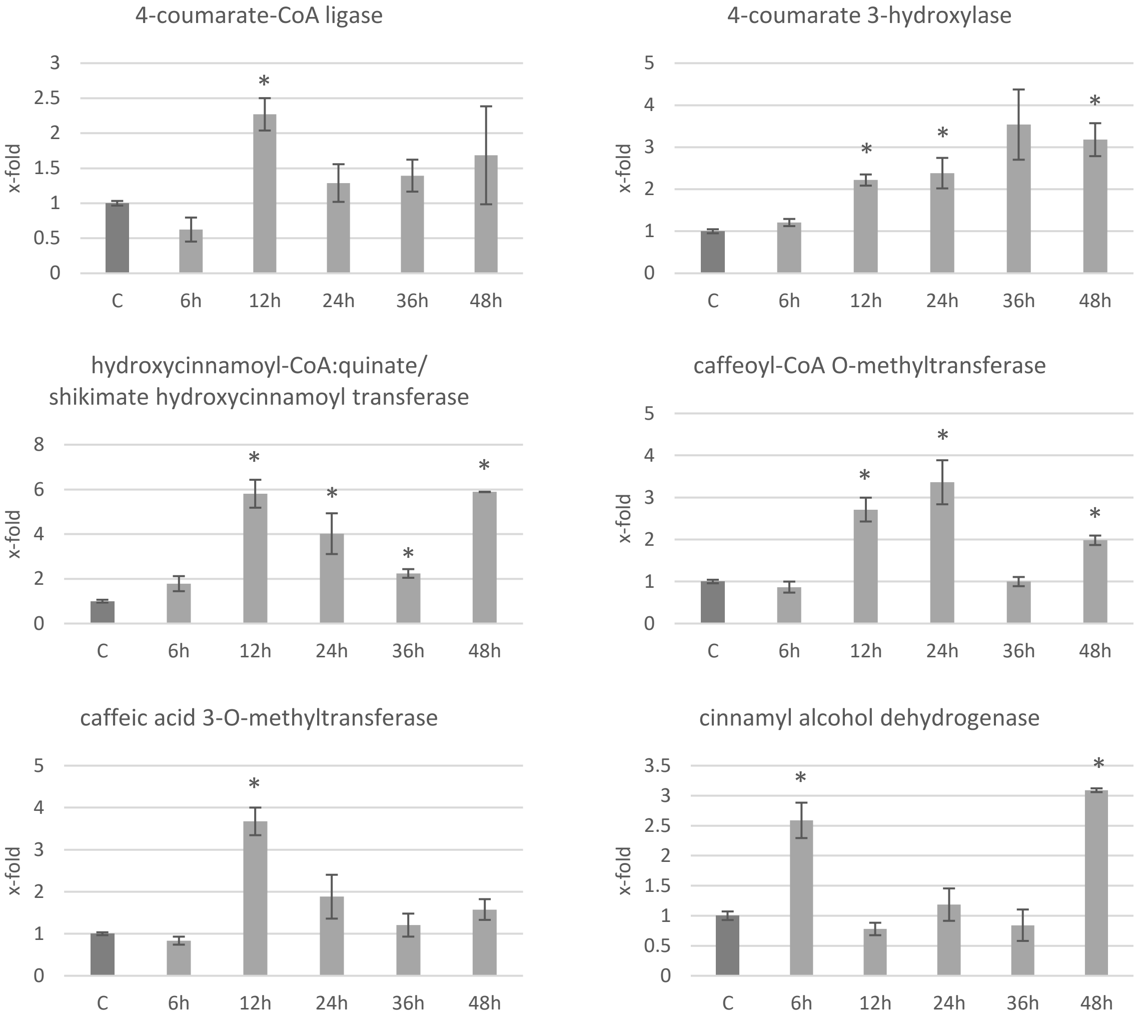

Supplementary Fig. 1. Levels of transcript of genes of the core route of the shikimate/phenylpropanoid pathway (A), the benzoate derivative biosynthesis route of the shikimate/phenylpropanoid pathway (B) and the hydroxycinnamic acid/lignin biosynthesis route of the shikimate/phenylpropanoid pathway (C) measured at 6, 12, 24, 36 and 48 hours after *F. oxysporum* infection relatively to control (marked C) (relative quantification, RQ=1). Actin was used as the reference gene. The results are presented as means ± standard deviations of three independent repeats. Changes statistically significant at $p<0.05$ are marked with asterisks.
